# Interrogating the *anti-*Insertion of Alkynes into Gold(III)

**DOI:** 10.1021/jacsau.5c00056

**Published:** 2025-02-26

**Authors:** Jaime Martín, Johannes Schörgenhumer, Cristina Nevado

**Affiliations:** Department of Chemistry, University of Zurich, Winterthurerstrasse 190, Zurich, CH 8057, Switzerland

**Keywords:** gold(III), hydrides, insertion reactions, alkynes, mechanisms

## Abstract

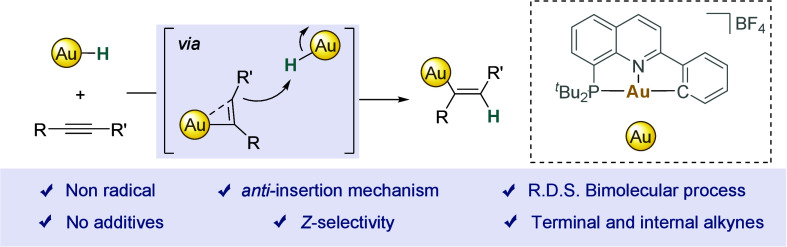

Alkyne hydrofunctionalizations are a powerful strategy to efficiently build up structural complexity. The selectivity of these reactions is typically governed by the interaction between the alkyne and a metal-hydride, which commonly proceeds via a well-understood *syn*-insertion mechanism. In contrast, *anti*-insertions are far less common, with proposed mechanisms often extrapolated from literature precedents rather than grounded in direct experimental evidence. While gold complexes rank among the most efficient catalysts for such transformations, the mechanistic understanding of the key alkyne insertion step remains incomplete. In this study, we demonstrate that stable gold(III)-hydrides, featuring a (P^∧^N^∧^C) ligand, undergo selective insertion of alkynes to yield the corresponding *anti*-Markovnikov *Z*-vinyl complexes. A combination of control experiments, kinetic studies, and computational analyses reveals a nonradical, bimolecular insertion process, in which water plays a pivotal role by accelerating the reaction and potentially stabilizing a highly reactive, T-shaped gold(I) intermediate. Notably, this is the first demonstration of the insertion of both activated and unactivated terminal and internal alkynes into a gold(III)-hydride complex.

## Introduction

Homogeneous gold catalysis, encompassing both Lewis acid^1,2^ and redox-mediated gold(I)/gold(III) processes,^3,4^ has experienced a remarkable growth over the past two decades.^5−9^ Gold has been pivotal in the efficient hydrofunctionalization of unsaturated moieties, including alkenes and allenes.^1,2^ However, alkynes have achieved the highest efficiencies and broadest range of applications,^10−18^ due to the synergistic effect of gold’s relativistic properties and its unparalleled ability to form strong π-backbonding interactions with triple bonds.^19^ Surprisingly, despite the frequent proposal of gold-vinyl complexes in many transformations,^20−22^ the mechanistic understanding of the specific alkyne insertion step into gold remains underdeveloped.^23,24^ This stands in stark contrast to the extensive knowledge amassed for other transition metals in similar processes.^25−33^

Pioneering work by Sadighi and co-workers featured the insertion of internal alkynes into (NHC)gold(I)-fluoride and -hydride bonds to form the corresponding *Z*-configured gold(I)-vinyl complexes.^34,35^ A similar stereochemical outcome was also reported for the reaction of dimethyl acetylenedicarboxylate (DMAD) with a diphosphene gold(I)-hydride.^36^ In contrast, *syn*-insertions of alkynes have been uncovered as well by Bourissou^37−39^ and Yamashita^40^ using gold(I)-silyl and -boryl complexes, respectively.

**Figure 1:**
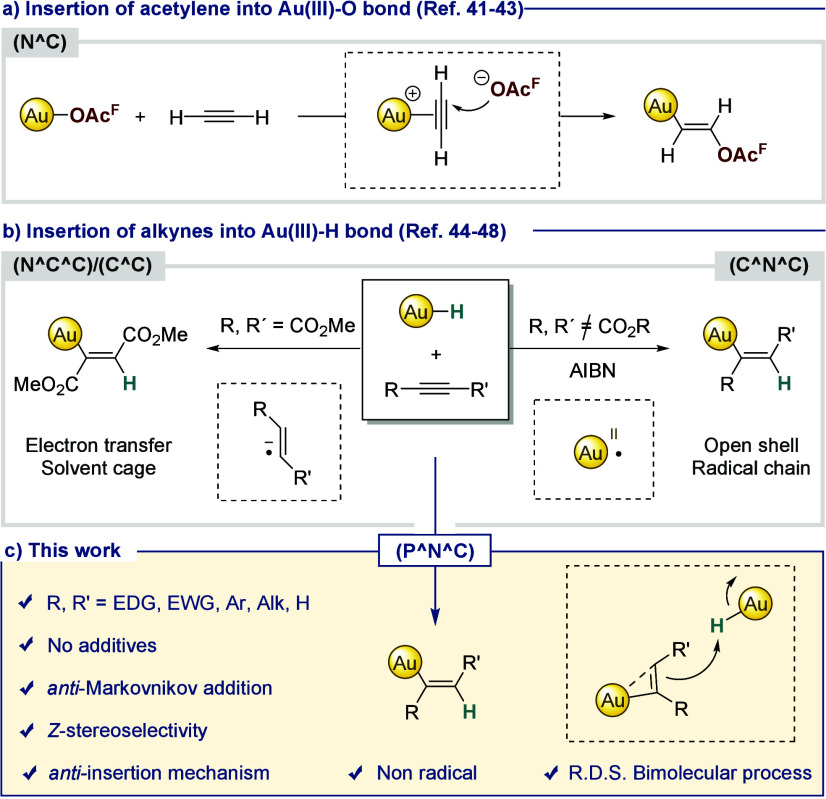
a) Insertion of acetylene into (N^∧^C)Au(III)–O bonds. b) Insertion of alkynes into Au(III)–H with (N^∧^C^∧^C), (C^∧^N^∧^C), and (C^∧^C) ligands via radical mechanisms. c) This work: Insertion of alkynes into Au(III)–H bonds with (P^∧^N^∧^C) ligands via a nonradical bimolecular mechanism.

For gold(III) species, seminal studies by Tilset and co-workers on the catalytic functionalization of acetylene revealed the formation of *Z*-vinyl gold(III) complexes after the insertion of acetylene into a gold(III)–OAc^F^ bond (OAc^F^ = OCOCF_3_).^41−43^ The reaction proceeded through a two-step process involving ligand substitution facilitated by the labile nature of the OAc^F^ group, followed by nucleophilic *anti*-addition (Figure 1a). On the other hand, Bochmann and Rocchigiani have studied the insertion of alkynes into gold(III)-hydrides. (C^∧^N^∧^C)-stabilized complexes could insert both terminal and internal unactivated alkynes (R, R′ ≠ CO_2_R) in the presence of azobis(isobutyronitrile) (AIBN), delivering *Z*-vinyl gold species.^44^ Control experiments and DFT calculations strongly suggest a radical-initiated, outer-sphere mechanism operative in these reactions (Figure 1b, right).^45,46^

In contrast, gold(III)-hydrides featuring (N^∧^C^∧^C) and (C^∧^C) ligands were able to insert, exclusively, DMAD to give the corresponding *Z*-vinyl products.^47,48^ An electron-transfer mechanism via an alkyne radical anion, followed by H abstraction and Au–C bond formation, was proposed to explain the observed *Z* stereochemistry, also justifying the need for alkynes with low reduction potentials (*E*_red_ = −0.8 V for DMAD) (Figure 1b, left).^48^

In our pursuit to characterize and study key elementary steps in gold(III) chemistry,^49−53^ we recently reported a novel class of (P^∧^N^∧^C) pincer ligands to stabilize gold(III)-hydrides under very mild conditions.^54^ Here, we describe that (P^∧^N^∧^C)gold(III)-hydrides are able to insert, for the first time, both terminal as well as internal, activated and unactivated alkynes, yielding the corresponding *anti*-Markovnikov addition, *Z*-vinyl configured complexes in the absence of external additives (Figure 1c). Despite the apparent simplicity of this two-component reaction, control experiments together with kinetic and computational studies suggest a bimolecular, nonradical process underlying the *anti*-insertion step. Moreover, we unravel the key role of water in accelerating and potentially generating a T-shaped short-lived gold(I) intermediate at the heart of these and likely other gold-mediated *anti*-insertion reactions.

## Results and Discussion

### Insertion of Alkynes into the Au–H Bond: Reactivity and Scope

**Figure 2:**
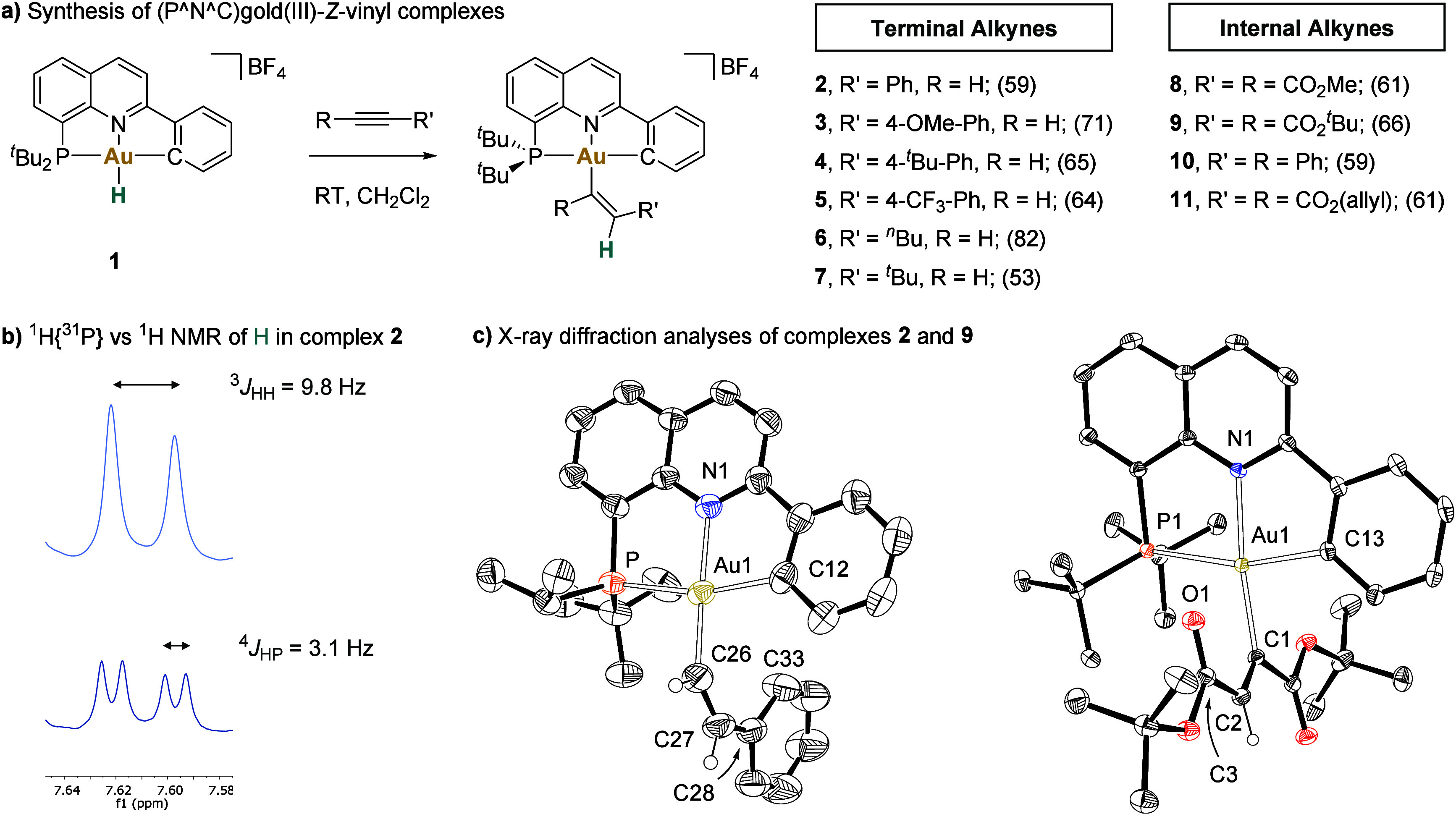
a) Reactivity of **1** with terminal and internal alkynes. Isolated yields are provided in brackets.^56^ b) Comparison between ^1^H{^31^P} (top) and ^1^H (bottom) NMR spectra in CD_2_Cl_2_ of one vinyl signal for compound **2**. c) ORTEP representation of **2** and **9** with 50% probability ellipsoids. Counter-anion, solvent molecule, and hydrogen atoms (except H26 and H27 in **2** and H2 in **9**) are omitted for clarity. Selected distances (Å) in **2**: Au1–P 2.3806(10), Au1–N1 2.053(4), Au1–C12 2.055(4), Au1–C26 2.014(5), C26–C27 1.329(7); and in **9**: Au1–P1 2.4049(6), Au1–N1 2.053(2), Au1–C13 2.055(2), Au1–C1 2.024(2), C1–C2 1.335(4). Selected angles (deg) in **2**: C12–Au1–P 164.33(12), C26–Au1–N1 173.37(16), Au1–C26–C27 131.8(4); and in **9**: C13–Au1–P1 164.02(8), C1–Au1–N1 171.28(9), Au1–C1–C2 122.82(19). Selected torsion angles (deg) in **2**: P1–Au1–C26–C27 97.7(5), C26–C27–C28–C29 20.4(8); and in **9**: P1–Au1–C1–C2 −95.7(2), and O1–C3–C2–C1 −7.5(4).

Despite their nonhydridic character, (P^∧^N^∧^C)gold(III)-hydrides react with allenes to yield 1,2-insertion products.^54^ Here, we set out to investigate the reactivity of gold(III)-hydride **1** with 1 equiv of phenylacetylene in dichloromethane at room temperature. We were delighted to observe the immediate formation, with *anti*-Markovnikov regioselectivity, of gold(III)-vinyl complex **2** in 59% isolated yield (Figure 2a). The ^1^H NMR spectrum exhibits a doublet of doublets at 7.61 ppm (^3^*J*_HH_ = 9.8 Hz, ^4^*J*_HP_ = 3.1 Hz), which is consistent with a *cis* disposition of the two H atoms in the double bond (Figure 2b). In addition, the ^*t*^Bu_2_ substituents of the phosphine become inequivalent (the CH_3_ groups display two doublets at 1.52 and 1.22 ppm), suggesting that the alkenyl group is not coplanar with respect to the (P^∧^N^∧^C)Au moiety. Single crystals of **2** suitable for X-ray diffraction analysis were obtained by slow vapor diffusion of diethyl ether into a solution of the compound in dichloromethane at low temperature.^55^ The crystal structure confirmed the *Z*-configuration of the double bond (Figure 2c, left). Further, the vinyl group adopts a perpendicular disposition with respect to the coordination plane of gold (torsion angle P–Au1–C26–C27: 97.7(5)°), and the phenyl group is almost coplanar to the double bond (torsion angle C26–C27–C28–C33: 20.4(8)°). Different solvents such as dichloromethane, acetone, and toluene proved to be compatible in the reaction of **1** with phenylacetylene, resulting in complete conversion to complex **2**. Additionally, the use of different counterions (BF_4_, B_Ar_^F^, and PF_6_) had no effect on the reaction outcome. These control experiments indicate that neither the solvent nor the counterion influences the final stereochemistry of the gold-vinyl product.^56^

The insertion of various terminal alkynes into the gold(III)–hydrogen bond was subsequently explored (Figure 2a). Under the same reaction conditions, phenylacetylene derivatives substituted with electron-donating (−OMe, −^*t*^Bu) and electron-withdrawing groups (−CF_3_) in the *para* position of the aromatic ring yielded complexes **3**–**5** in good yields (64–71%). Other terminal alkynes such as 1-hexyne and ^*t*^Bu-acetylene also furnished the corresponding vinyl gold species **6** and **7**, although the latter required heating for 24 h at 323 K, likely due to steric hindrance imposed by the ^*t*^Bu group. According to the recorded spectroscopic data, all gold(III)-vinyl complexes displayed a *Z*-configuration.

The compatibility of the reaction with internal alkynes was also investigated. Treatment of **1** with DMAD and di-*tert*-butyl acetylenedicarboxylate (DTAD) generated the corresponding insertion gold complexes (**8** and **9**) in 61% and 66% yield, respectively, after 24 h at room temperature. Even the less activated diphenyl acetylene reacted to furnish the vinyl complex **10** in 59% isolated yield. Single crystals of **9** suitable for X-ray diffraction analysis were obtained by slow vapor diffusion of pentane into a solution of the compound in dichloromethane at low temperature.^55^ The crystal structure confirmed the *trans* disposition of the two carboxylate substituents, and thus the exclusive formation of the *Z*-isomer, also with internal alkynes (Figure 2c, right). As in the case of **2**, the vinyl group appears to be perpendicular to the coordination plane of gold (torsion angle P1–Au1–C1–C2: −95.7(2)°). Here, the carboxylate groups appear coplanar to the π-system, thus suggesting π delocalization across the vinyl moiety (torsion angle, O1–C3–C2–C1: −7.5(4)°). These results represent the first time that both electronically biased and unbiased terminal and internal alkynes can be inserted into gold-hydrides devoid of external additives.^35,36,44,47,48^

### Mechanistic Investigations

Our efforts then turned to investigate the mechanism underlying these transformations. While the *syn*-insertion of alkynes into metal hydrides is commonly proposed to occur via a concerted process, an *anti*-insertion can be explained through a variety of mechanisms. Such proposals range from *syn*-insertion followed by rapid isomerization, participation of η^1^-vinylidene intermediates, concerted pathways, or the involvement of radicals.^57−67^

#### Radical Trapping Experiments

The reaction of **1** with phenylacetylene was investigated using various radical probes.^56^ First, the addition of 2,2,6,6-tetramethylpiperidinyloxy (TEMPO) or butylated hydroxytoluene (BHT) did not significantly affect the formation of **2** (eq 1). These results contrast with the behavior observed for (C^∧^N^∧^C)Au-H, which not only requires a radical initiator (AIBN) to promote the insertion but also shuts down the reaction in the presence of TEMPO.^44^ Given that some Au–H complexes have been reported to be light sensitive,^68^ trace amounts of radical species might be generated during the reaction. To exclude this possibility, we carried out the insertion of phenylacetylene in the dark, finding equally productive conditions for the formation of **2**. Furthermore, the reaction of **1** with phenylacetylene in the presence of BHT-*d*_1_ or ethylbenzene-*d*_10_ produced *Z-*vinyl-gold product **2** with no incorporation of deuterium (eq 2). Finally, a radical clock experiment was conducted employing diallyl acetylenedicarboxylate (DAAD). The reaction afforded the *Z*-vinyl complex **11** in 61% isolated yield after 24 h at room temperature, and no cyclization product was observed (eq 3). These results strongly suggest that neither a radical chain mechanism nor open-shell species are operative under our reaction conditions.
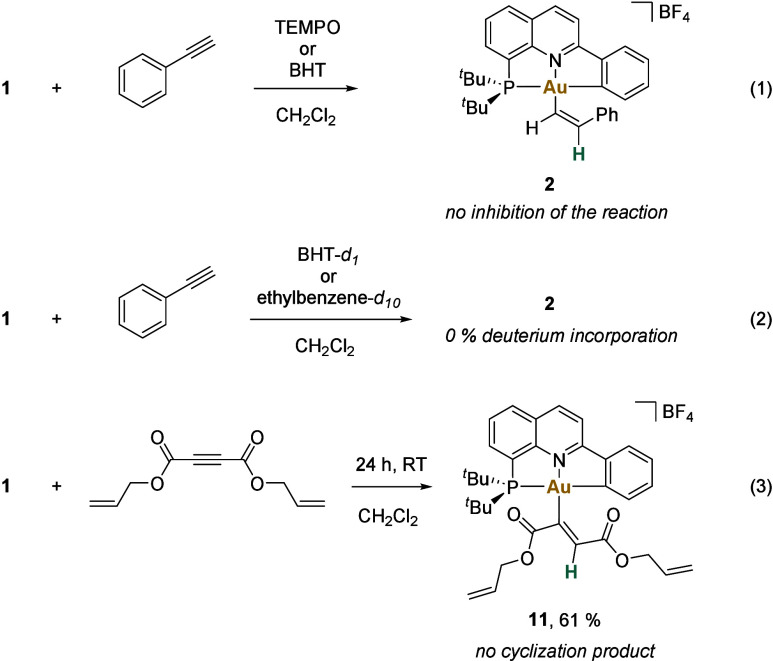


#### Deuterium-Labeling Experiments

**Figure 3:**
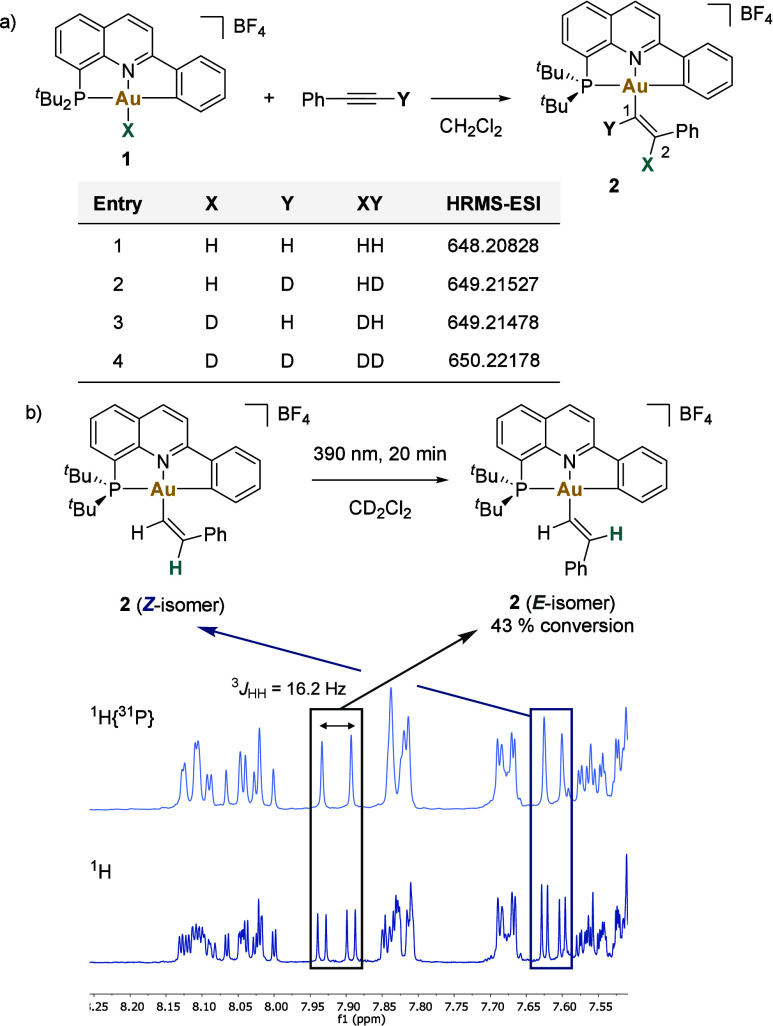
a) Deuterium-labeling experiments. b) Photoisomerization of **2** after illumination at 390 nm for 20 min in CD_2_Cl_2_. Comparison of the ^1^H{^31^P} (top) and ^1^H (bottom) NMR spectra.

Reactions were carried out between gold-hydride **1** and its deuteride counterpart **1-D**, and phenylacetylene and phenylacetylene-*d*_1_ (Figure 3a). These experiments furnished compounds **2**, **2-HD**, **2-DH**, and **2-DD** (characterized by HRMS and NMR spectroscopy). These results clearly showcase that the C1–Y bond of the vinyl system stems exclusively from the alkyne precursor, whereas C2–X accommodates the H or D atom delivered by the initial gold(III) complex.^56^

#### *E*/*Z* Isomerization

A plausible explanation for the formation of the *anti*-addition products could involve a classical *syn-*insertion reaction followed by a rapid *E/Z* isomerization process, potentially mediated by either a zwitterionic species or a metallacyclopropene intermediate.^69−71^ However, while the feasibility of *syn*-insertion of alkynes has been demonstrated in gold(I) chemistry,^37−40^ such a reaction pathway is not favored for gold(III), which lacks available coordination sites.^72^

The conversion of the *Z*-vinyl-gold compounds to their *E*-isomers can be achieved by illuminating the samples with UV light.^56^ Following a 20 min exposure of (*Z*)-**2** to 390 nm light, a mixture of *Z/E* (57/43) was observed (Figure 3b). Analysis of the mixture by ^1^H NMR spectroscopy revealed a new set of signals featuring a doublet of doublets at 7.91 ppm (^3^*J*_HH_ = 16.2 Hz, ^4^*J*_HP_ = 4.7 Hz), typical for *trans* H–H coupling. Notably, this *Z/E* ratio remained unchanged over time, indicating that a facile *Z/E* isomerization is not occurring. Similar behavior was observed in photoisomerization experiments with compounds **8** and **9**. Moreover, no evolution between isomers was observed in the presence of **1**, indicating that under the reaction conditions, complex **1** does not promote a *Z/E* interconversion.^56^ These experiments suggest that the *Z*-isomer is the primary product resulting from the insertion of alkynes into the Au–H bond, and no prior *syn*-insertion followed by an *E/Z* isomerization process takes place.

#### Kinetics

**Figure 4:**
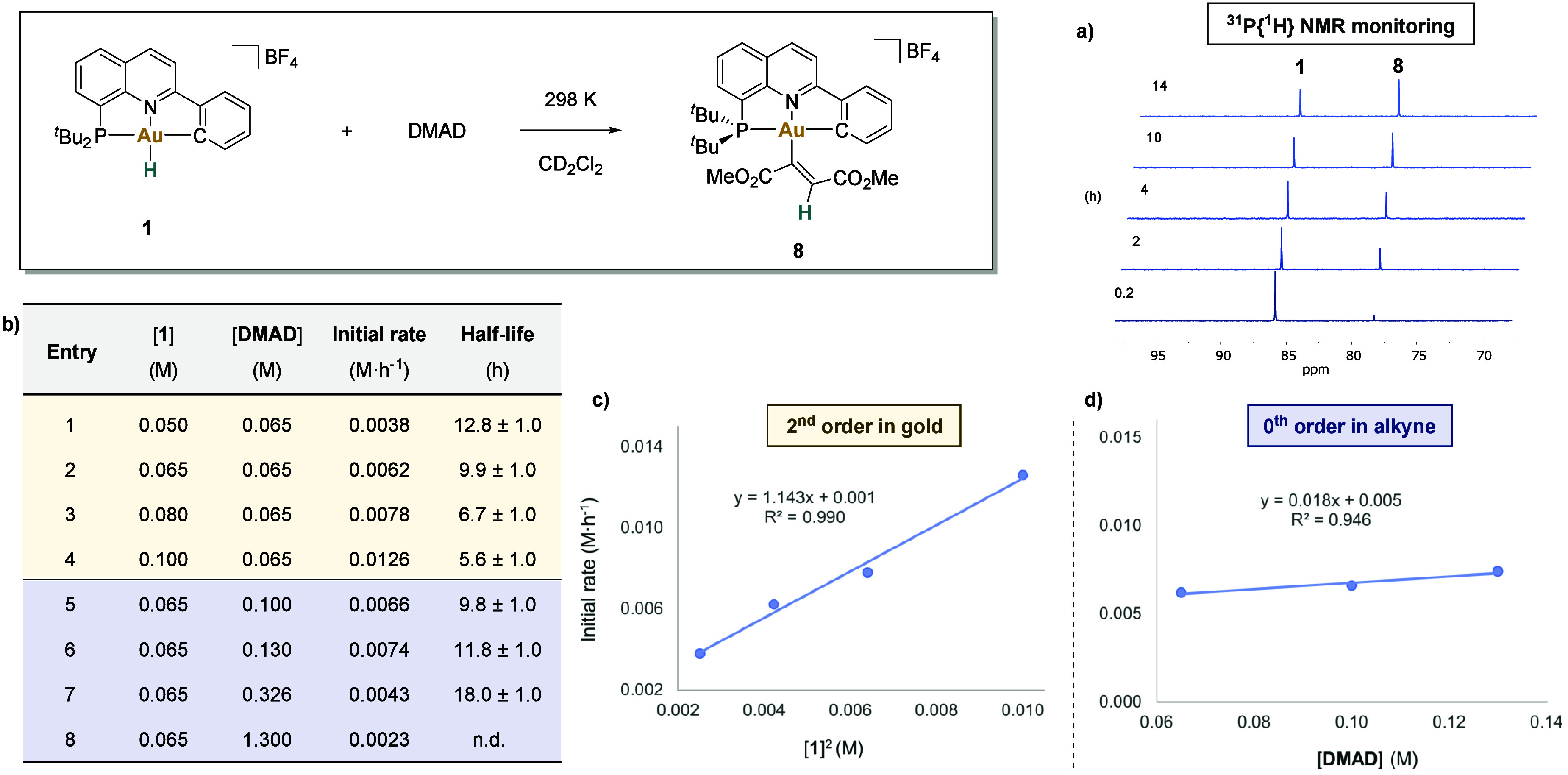
Kinetic study. a) Reaction of **1** (0.065 M) with 1 equiv of DMAD monitored by ^31^P{^1^H} NMR (161.99 MHz, in CD_2_Cl_2_) at 298 K over time. b) Kinetic data for the reaction of **1** with DMAD. c) Plot of initial rates vs [**1**]^2^, using 0.065 M of DMAD (table in Figure 4b, entries 1–4). d) Plot of initial rates vs [DMAD], using 0.065 M of **1** (table in Figure 4b, entries 2, 5, 6).^56^

To get a deeper insight into the reaction mechanism, the insertion reaction was monitored using ^31^P{^1^H} NMR spectroscopy.^56^ Due to the rather fast reaction observed for terminal alkynes, we selected DMAD as the alkyne of choice for these experiments. Figure 4a illustrates the monitoring of the insertion reaction by ^31^P{^1^H} NMR spectroscopy, showing the consumption of **1** and the formation of the *Z*-isomer of compound **8** over time. No other intermediates were detected.

Next, the kinetics of the insertion reaction were studied. Experiments were carried out in CD_2_Cl_2_, under an argon atmosphere, in the dark, at 298 K.^56^ Initial rates (table in Figure 4b) revealed a second-order dependence on gold (Figure 4c). On the other hand, a zero-order dependence in DMAD (0.065–0.130 M) was found (Figure 4d). These data suggest that the interaction of the alkyne with the metal is faster than the rate-determining step. Interestingly, using an excess of DMAD, the reaction rate is significantly reduced, resulting in longer half-life values (table in Figure 4b, entries 7, 8), which compromises the kinetic study under pseudo-first-order conditions. In addition, Eyring analysis was performed^56^ providing the following thermodynamic parameters for the reaction: Δ*H*^⧧^ = 15 ± 1 kcal·mol^–1^, Δ*S*^⧧^ = −25 ± 3 cal·mol^–1^·K^–1^, and Δ*G*_298 K_^^⧧^^ = 22 ± 1 kcal·mol^–1^. Notably, the negative entropic value suggests an associative mechanism, in which two partners combine to form a single activated complex. The energy values are in the range of the ones calculated by DFT (*vide infra*).

#### Effect of Water

**Figure 5:**
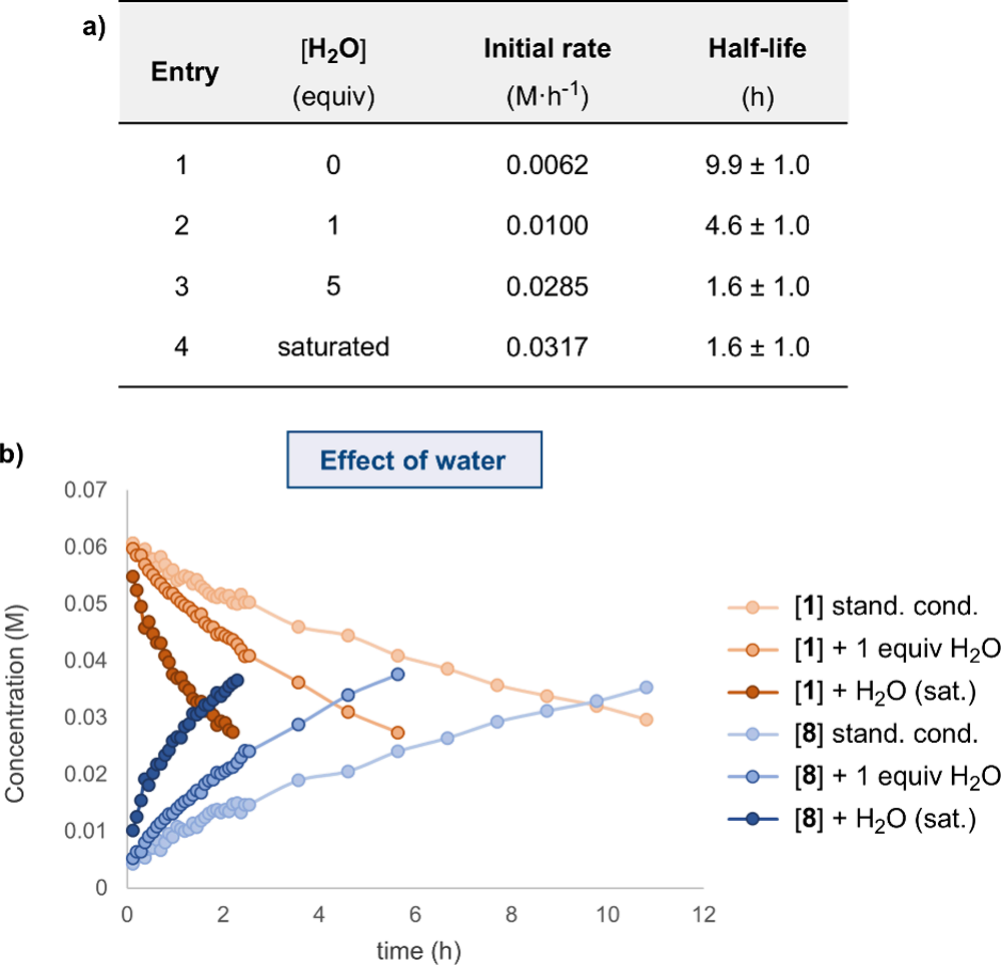
a) Kinetic data for the reaction of **1** (0.065 M) with DMAD (0.065 M) in CD_2_Cl_2_ in the presence of water.^73^ b) Partial concentrations of **1** (orange) and **8** (blue) under standard conditions (entry 1), with 1 equiv of water (entry 2), and CD_2_Cl_2_ saturated with water (entry 4). Entry 3 of the table is not included for the sake of clarity.

During our investigations, we observed that the presence of moisture had an impact on the kinetics. To quantify this effect, we performed experiments by adding known amounts of water to the equimolar reaction of **1** with DMAD and tracking the concentrations of **1** and **8** over time. As a result, we observed that the addition of small amounts of water in dichloromethane (table in Figure 5a) significantly increased the reaction rate and reduced the half-life values (Figure 5b).^56^

#### Mechanistic Proposal

Based on the results of this study, a concerted mechanism involving a four-membered *syn*-addition intermediate followed by rapid isomerization can be ruled out, as no isomerization between the *Z-* and *E*-isomers was observed under the reaction conditions. Furthermore, control experiments, including those outlined in eqs 1–3, helped to exclude the possibility of a radical mechanism operating in these transformations. Kinetic analysis revealed a second-order dependence on gold and a zero-order dependence on alkyne, suggesting a bimolecular process in which two gold molecules interact in the rate-determining step, in line with the negative entropy obtained via Eyring analysis (Δ*S*^⧧^ = −25 ± 3 cal·mol^–1^·K^–1^).

**Figure 6:**
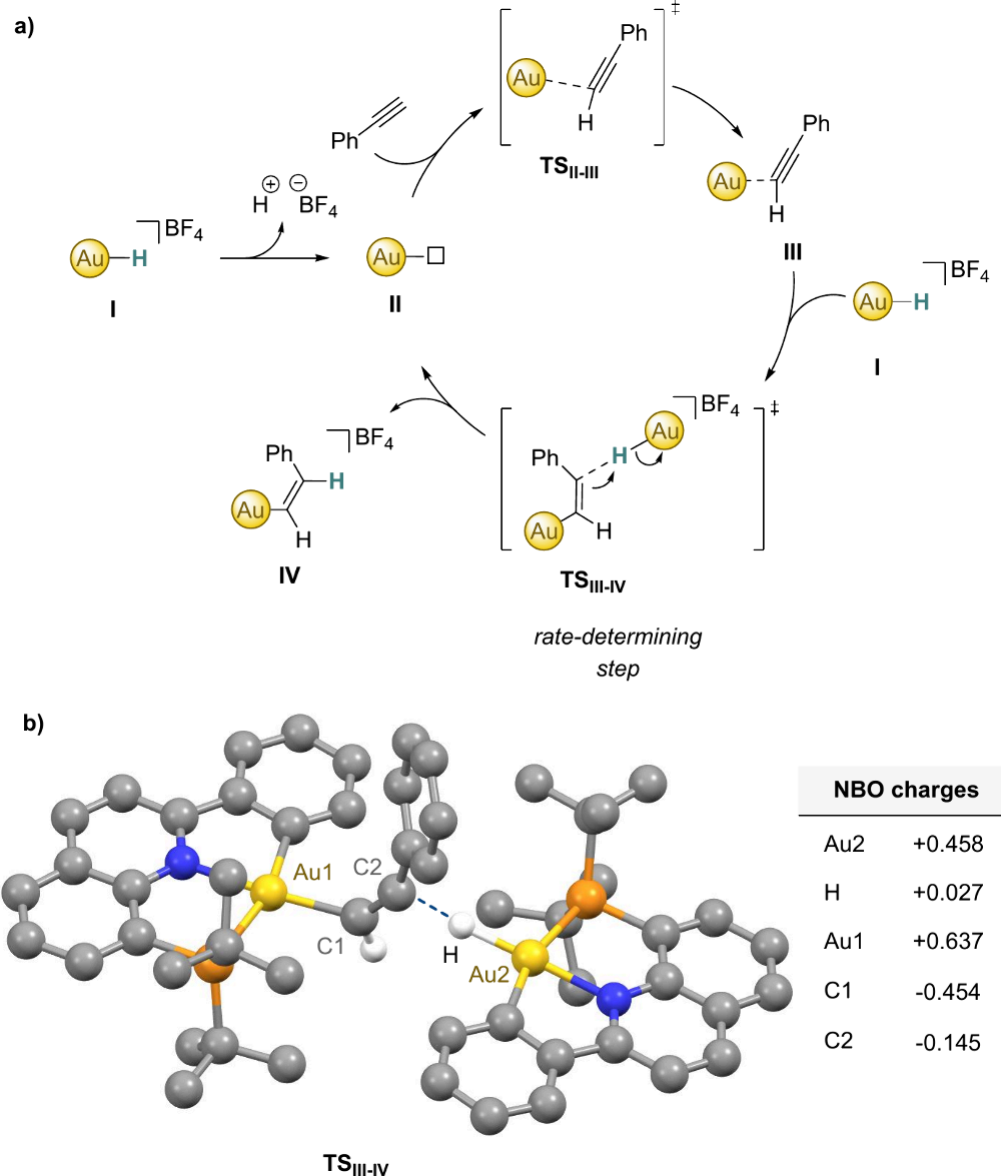
a) Proposed ionic mechanism for the insertion reaction of alkynes into the Au–H bond. b) Optimized geometry of the key bimolecular species **TS**_**III–IV**_ involved in the rate-determining step, computed at the PBEPBE/DEF2TZVP level of theory. Selected distances (Å): Au1–C1 2.076, C1–C2 1.300, C2–H 1.634, Au2–H 1.673.^56^

Our proposal involves the initial formation of an *anti*-Markovnikov vinyl gold complex (**III**) through hydride displacement from **I**,^74−76^ potentially facilitated by the presence of a molecule of water via a short-lived intermediate **II**, which according to DFT calculations can be formally considered a T-shaped gold(I) complex (Figure 6a).^77^ While the notion of water abstracting the nonhydridic Au-hydride to form a hydronium cation (H_3_O^+^) seems speculative at first,^78−82^ a single such event would allow the reaction to continue until the complete consumption of **I**. In this scenario, the observed acceleration of the overall process upon the addition of extra water also seems justified. In fact, a control experiment involving Au–H in the presence of D_2_O resulted in a mixture of Au–D and Au–H, demonstrating the exchange process between water and Au–H (see Supporting Information). Once gold intermediate **III** is formed, a second molecule of complex **I** is responsible for the *anti*-addition of the hydrogen atom, in line with the observed second order in gold. Such a bimolecular process leads to the formation of the final *Z*-vinyl species **IV** and another molecule of gold complex **II**. According to DFT calculations,^56^ intermediate **III** formally features a gold(I) metal center and a substantial electron density at the alkyne, thus explaining the abstraction of the hydrogen from another molecule of compound **I**, which *de facto* becomes a “proton donor”.^83^ The observed zero-order dependence on the initial concentration of the alkyne could be explained by the faster coordination of the alkyne to **II** compared to the interaction of the two gold units. Interestingly, the reaction rate is significantly reduced when the reaction is carried out using an excess of DMAD, highlighting that the alkyne can affect the rate-limiting step by preventing two gold units from coming into close proximity. In contrast, when the concentration of alkyne and gold are similar, the two gold units can approach each other more easily. Alternative pathways to initiate the reaction, such as a bimolecular reaction between two gold-hydrides resulting in the formation of a gold dihydride (for example via decoordination of one of the donor atoms of the (P^∧^N^∧^C) ligand) and intermediate **II**, cannot be ruled out at this stage but are deemed less likely since they did not account for the role of water and experimental evidence for the formation of a gold dihydride could not be gathered.^56^

A DFT analysis of this transformation enabled the characterization of the key bimolecular Au–H addition step.^56^ This step involves a transition state featuring the *anti*-addition of a Au–H molecule to an alkyne, which is coordinated to a second gold unit, with an activation barrier of 16.5 kcal mol^–1^ (Figure 6b).^56^ In this **TS**_**III–IV**_, both Au complexes are situated in a plane solely connected by interactions around the alkyne coordinated perpendicularly. In this arrangement, the alkyne is found to be coordinated to the first gold center at a distance of 2.076 Å (Au1–C1), whereas the second gold molecule is found at 1.634 Å from the H of the Au2–H bond to carbon C2 of the alkyne. The Au2–H bond is elongated to 1.673 Å as compared to the distance of 1.576 Å calculated for complex **1**.^54^ The geometry of the bimolecular transition structure offers an explanation for the reaction’s stereoselectivity for the *Z*-isomer as the steric hindrance posed by the (P^∧^N^∧^C)-Au-alkene complex prevents the Au–H from approaching via any other trajectory.

The analysis of natural bond orbital (NBO) partial charges reveals a significantly positively charged Au center (+0.458) for the Au2–H unit and an almost neutral hydrogen (+0.027), in line with our previous results for Au–H complexes with (P^∧^N^∧^C) ligands.^54^ On the alkyne-Au part, a higher positive partial charge (+0.637) is observed at the metal, which is connected to a strongly negatively charged terminal carbon atom C1 of the alkyne (−0.454). Likewise, C2 of the alkyne is found to bear a negative partial charge (−0.145), fostering a proton transfer from the Au–H, as proposed based on experimental findings. This view is consistent with the formal oxidation of the metal center upon release of the vinyl adduct **IV** and the regeneration of T-shaped gold(I) intermediate **II** that inserts the next alkyne moiety.

## Conclusions

The insertion of alkynes into a (P^∧^N^∧^C)gold(III)-hydride yields *Z*-configured gold-vinyl complexes with *anti*-Markovnikov regioselectivity. While this two-component reaction has been extensively studied with other metal-hydrides, we demonstrate for the first time with gold that it proceeds with both terminal and internal, electronically biased and unbiased alkynes, without the need for external additives. Our investigations ruled out several commonly proposed pathways for *anti-*addition reactions, including radical mechanisms, *syn-*addition followed by isomerization, and mononuclear processes. Instead, our findings suggest a bimolecular mechanism as the rate-determining step. The controlled introduction of water significantly accelerated the reaction, drastically reducing half-life values and pointing to the possible formation of a T-shaped short-lived, gold(I) intermediate that may mediate this and potentially other *anti-*insertion reactions.

## Methods

### General Procedure for the Synthesis of Gold Complexes (**2**–**11**)

A solution of (P^∧^N^∧^C)gold(III)-hydride **1** (1 equiv) in 1 mL of dichloromethane was treated with the corresponding alkyne (1 equiv) at room temperature. After completion of the reaction (ca. 15 min for terminal alkynes and ca. 24 h for internal alkynes), the mixture was concentrated under reduced pressure. The solid was washed with diethyl ether and pentane and subsequently dried under vacuum to deliver the corresponding (P^∧^N^∧^C)gold(III)-vinyl complex.

## Supporting Information Available

(General procedures, synthesis and characterization of the new compounds, mechanistic investigations, NMR spectra, X-ray diffraction analyses, and DFT calculations PDF)
X-ray crystallographic data for **2** (CIF)
X-ray crystallographic data for **9** (CIF)

## Supplementary Material

au5c00056_si_001.pdf

au5c00056_si_002.cif

au5c00056_si_003.cif
