# Supplementary material for: Interrogating the anti-Insertion of Alkynes into Gold(III)
Source: JACS Au. 2025 Feb 26;5(3):1439–47. doi: 10.1021/jacsau.5c00056 (PMC11938007; doi:10.1021/jacsau.5c00056)
Supplement: Supplementary file 1 — au5c00056_si_001.pdf [file au5c00056_si_001.pdf]

Supporting Information

Interrogating the *anti*-Insertion of Alkynes into Gold(III)

*Jaime Martín, Johannes Schörgenhumer, and Cristina Nevado**

Department of Chemistry, University of Zurich, Winterthurerstrasse 190, Zurich, CH 8057, Switzerland

*Corresponding author e-mail address: cristina.nevado@chem.uzh.ch

Contents	Page
1. General information and experimental methods	S2
2. Synthesis and characterization of new compounds	S3
3. Mechanistic investigations	S10
3.1 Effect of counterion and solvent	S10
3.2 Radical trapping experiments	S12
3.3 Isomerization experiments	S15
3.4 Kinetics	S21
3.5 Calculation of thermodynamic activation parameters	S26
3.6 Exchange between Au-H and water	S27
3.7 Effect of water on the kinetics	S28
4. NMR spectra	S31
5. X-ray diffraction analyses	S55
6. DFT calculations	S59
7. References	S100

1. General information and experimental methods

Unless otherwise stated, reactions were performed under nitrogen or argon atmosphere using Schlenk techniques and gloveboxes. Commercial chemicals were used as received. Complex **1**,[1] diallyl acetylenedicarboxylate (DAAD)[2] and 2,6-di-*tert*-butyl-4-methyl-1-deuteriophenol (BHT-d_1)[3] were prepared according to reported procedures. Phenylacetylene-d_1 was synthesized by reacting phenylacetylene with 1 equiv of nBuLi in THF at -78 ºC, followed by quenching with D_2O. The product exhibited >99% of deuteration, as confirmed by its ^{1}H NMR spectrum. Flash column chromatography was performed over silica gel (230-400 mesh). NMR spectra (^{1}H, ^{2}H, ^{13}C, ^{19}F, ^{31}P and 2D experiments) were recorded on either AV2 400, AV2 500 or Avance Neo 500 MHz Bruker spectrometers. Chemical shifts are given in ppm. ^{1}H and ^{13}C NMR spectra are referenced to residual solvent peaks.[4] ^{2}H, ^{19}F and ^{31}P NMR spectra are referenced to TMS-d_{12}, $CFCl_3$ and 85% H_3PO_4, respectively. Multiplicities are abbreviated: singlet (s), doublet (d), triplet (t), quartet (q), septuplet (sept), multiplet (m), and broad (br). Constant couplings are given in Hz. High resolution electrospray ionization mass spectrometry (HR-ESI-MS) were measured in *QExactive* or *timsTOF* instruments. *Dionex Ultimate 3000* UHPLC system (*ThermoFischer Scientifics*, Germering, Germany) connected to a *QExactive* MS with a heated ESI source (*ThermoFisher Scientific*, Bremen, Germany); onflow injection of 1 μL sample (*c* = ca. 50 μg mL^{-1} in the indicated solvent) with an *XRS* auto-sampler (*CTC*, Zwingen, Switzerland); flow rate 120 μL min^{-1}; ESI: spray voltage 3.0 kV, capillary temperature 280 °C, sheath gas 30 L min^{-1}, aux gas 8 L min^{-1}, s-lens RF level 55.0, aux gas temperature 250 °C (N_2); full scan MS in the alternating (+)/(−)-ESI mode; mass ranges 80–1'200 *m/z*, 133–2'000 *m/z*, or 200–3'000 *m/z* at 70'000 resolution (full width half-maximum); automatic gain control (AGC) target of $3.00 \cdot 10^6$; maximum allowed ion transfer time (IT) 30 ms; mass calibration to <2 ppm accuracy with *Pierce*® ESI calibration solutions. (*ThermoFisher Scientific*, Rockford, USA); lock masses: ubiquitous erucamide (*m/z* 338.34174, (+)-ESI) and palmitic acid (*m/z* 255.23295, (−)-ESI). *TimsTOF Pro* TIMS-QTOF-MS instrument (*Bruker Daltonics* GmbH, Bremen, Germany). The samples were dissolved in (e.g. MeOH) at a concentration of ca. 50 µg ml^{-1} and analyzed via continuous flow injection (2 µL min^{-1}). The mass spectrometer was operated in the positive (or negative) electrospray ionization mode at 4'000 V (-4'000 V) capillary voltage and -500 V (500 V) endplate offset with a N_2 nebulizer pressure of 0.4 bar and a dry gas flow of 4 l min^{-1} at 180 °C. Mass spectra were acquired in a mass range from *m/z* 50 to 2'000 at ca. 20'000 resolution (*m/z* 622) and at 1.0 Hz rate. The mass analyzer was calibrated between *m/z* 118 and 2'721 using an *Agilent* ESI-L low concentration tuning mix solution (*Agilent*, USA) at a resolution of 20'000 giving a mass accuracy below 2 ppm. All solvent used were purchased in best LC-MS quality. Infrared spectra were recorded on a JASCO FT/IR-4100 spectrometer. Single crystal X-ray diffraction data were collected at 160.0(1) K on a Rigaku Oxford Diffraction XtaLAB Synergy-S dual source diffractometer[5]: Kappa-axis four circle goniometer with a HyPix 6000 (**2**) or a Dectris Pilatus3 R 200K (**9**) hybrid pixel area detector and Mo (λ = 0.71073 Å) PhotonJet microfocus X-ray source.

Safety Statement: no uncommon hazards are noted.

2. Synthesis and characterization of new compounds

General procedure for the synthesis of complexes (2-11). A solution of (P^N^C)gold(III)-hydride **1** (1 equiv) in 1 mL of dichloromethane was treated with the corresponding alkyne (1 equiv) at room temperature. After completion of the reaction (ca. 15 min for terminal alkynes, ca. 24 h for internal alkynes), the mixture was concentrated under reduced pressure and the resulting crude was treated with diethyl ether to afford the corresponding (P^N^C)gold(III)-vinyl complex as a yellow solid, which was washed with diethyl ether and pentane, and subsequently dried under vacuum.

(P^N^C)Au-D (1-D). Compound **1-D** was synthesized by stirring a solution of **1** (40 mg, 0.063 mmol) in 2 mL methanol-d_1. After 30 minutes, 10 mL of Et_2O was added to afford a pale yellow solid, which was washed with Et_2O and pentane, and dried under vacuum. Yield: 53 mg (44%). ^{1}H NMR (400.13 MHz, CD_2Cl_2, 298 K): δ 8.92 (dd, $^3J_{HH}$ = 8.9, 1H), 8.40-8.34 (m, 3H), 8.12-8.08 (m, 1H), 8.05-8.01 (m, 2H), 7.56-7.54 (m, 2H), 1.52 (d, $^3J_{HP}$ = 16.9, 18H). ^{2}H NMR (61.42 MHz, CD_2Cl_2, 298 K): δ -5.45 (br). ^{13}C{^{1}H} NMR (100.62 MHz, CD_2Cl_2, 298 K): δ 166.8 (d, $^3J_{CP}$ = 5.6), 162.5 (d, $^2J_{CP}$ = 110.9), 150.8 (d, $^2J_{CP}$ = 11.5), 149.1 (s), 144.2 (s), 141.4 (d, $^2J_{CP}$ = 1.2), 139.8 (d, $^3J_{CP}$ = 3.0), 134.8 (d, $^4J_{CP}$ = 8.4), 134.2 (d, $^4J_{CP}$ = 2.1), 130.4 (d, $^3J_{CP}$ = 6.9), 130.1 (d, $^3J_{CP}$ = 6.7), 129.0 (d, $^1J_{CP}$ = 38.9), 128.9 (s), 128.8 (s), 120.0 (s), 38.5 (d, $^1J_{CP}$ = 17.8), 30.1 (d, $^2J_{CP}$ = 4.6). ^{31}P{^{1}H} NMR (161.99 MHz, CD_2Cl_2, 298 K): δ 85.7 (br). (+)-HR-ESI-MS (electrospray, m/z): calcd for $C_{23}H_{27}DNAuP$ $[M]^+$, 547.16823; found, 547.16742.

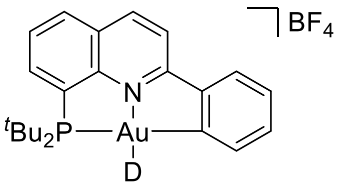

(P^N^C)Au-(*Z*)-(C(H)=C(H)Ph) (2). Compound **2** was synthesized following the general procedure using **1** (20.0 mg, 0.032 mmol) and phenylacetylene (3.5 µL, 0.032 mmol). Yield: 13.8 mg (59%). ^{1}H NMR (400.13 MHz, CD_2Cl_2, 298 K): δ 8.94 (dd, $^3J_{HH}$ = 8.8, $^5J_{HP}$ = 1.4, 1H), 8.43-8.37 (m, 3H), 8.11 (ddd, $^3J_{HH}$ = 7.5, $^4J_{HH}$ = 3.6, $^4J_{HP}$ = 1.7, 1H), 8.01 (ddd, $^3J_{HH}$ = 7.8, $^3J_{HH}$ = 7.6, $^4J_{HP}$ = 1.7, 1H), 7.83-7.79 (m, 1H), 7.74 (dd, $^3J_{HH}$ = 9.7, $^4J_{HP}$ = 1.8, 1H), 7.62-7.58 (m, 2H), 7.54-7.43 (m, 3H), 6.75-6.71 (m, 2H), 3.69 (s, 3H), 1.53 (d, $^3J_{HP}$ = 16.4, 9H), 1.26 (d, $^3J_{HP}$ = 16.7, 9H). ^{13}C{^{1}H} NMR (100.62 MHz, CD_2Cl_2, 298 K): δ 166.4 (d, $^3J_{CP}$ = 5.9), 161.7 (d, $^2J_{CP}$ = 113.7), 150.5 (d, $^2J_{CP}$ = 10.9), 148.3 (s), 144.3 (s), 141.6 (d, $^2J_{CP}$ = 1.2), 138.6 (s), 134.3 (d, $^4J_{CP}$ = 2.1), 134.1 (d, $^4J_{CP}$ = 8.0), 134.0 (d, $^2J_{CP}$ = 1.3), 132.6 (d, $^3J_{CP}$ = 2.9), 131.5 (d, ^3J = 8.8), 130.4 (d, $^3J_{CP}$ = 6.5), 129.9 (d, $^3J_{CP}$ = 6.5), 129.3 (s), 129.0 (s), 128.9 (d, $^4J_{CP}$ = 5.6), 128.8 (d, $^1J_{CP}$ =

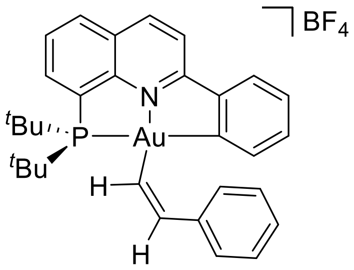

35.9), 128.2 (s), 127.9 (s), 120.0 (s), 39.3 (d, $^1J_{CP}$ = 15.6), 39.0 (d, $^1J_{CP}$ = 15.2), 29.8 (d, $^2J_{CP}$ = 3.9), 29.4 (d, $^2J_{CP}$ = 4.2). ^{31}P{^{1}H} NMR (161.99 MHz, CD_2Cl_2, 298 K): δ 75.4 (s, *P*). (+)-HR-ESI-MS (electrospray, m/z): calcd for $C_{31}H_{34}NAuP$ $[M]^+$, 648.20889; found, 648.20828. Single crystals of **2** suitable for X-ray diffraction analysis were obtained by slow vapor diffusion of diethyl ether into a solution of the compound in dichloromethane at low temperature.

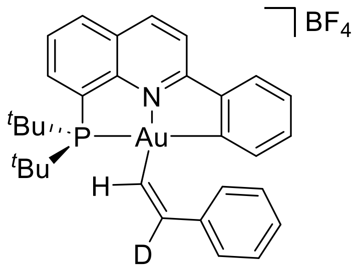

(P^N^C)Au-(*Z*)-(C(H)=C(D)Ph) (2-HD). Compound **2-HD** was synthesized following the general procedure using **1-D** (10.0 mg, 0.016 mmol) and phenylacetylene (8.6 µL, 0.079 mmol). Yield: 4 mg (35%). ^{1}H NMR (400.13 MHz, CD_2Cl_2, 298 K): δ 8.93 (dd, $^3J_{HH}$ = 8.8, $^5J_{HP}$ = 1.3, 1H), 8.42-8.38 (m, 3H), 8.11-8.08 (m, 1H), 8.03-7.99 (m, 1H), 7.84-7.80 (m, 1H), 7.68-7.65 (m, 2H), 7.60 (d, $^4J_{HP}$ = 3.1, 1H), 7.55-7.47 (m, 2H), 7.22-7.18 (m, 3H), 1.52 (d, $^3J_{HP}$ = 16.5, 9H), 1.22 (d, $^3J_{HP}$ = 16.5, 9H). ^{31}P{^{1}H} NMR (161.99 MHz, CD_2Cl_2, 298 K): δ 75.3 (s, *P*). (+)-HR-ESI-MS (electrospray, m/z): calcd for $C_{31}H_{33}DNAuP$ $[M]^+$, 649.21517; found, 649.21527.

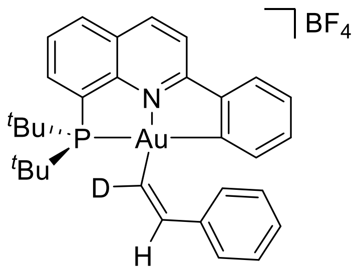

(P^N^C)Au-(*Z*)-(C(H)=C(D)Ph) (2-DH). Compound **2-DH** was synthesized following the general procedure using **1** (10.0 mg, 0.016 mmol) and phenylacetylene-d_1 (8.6 µL, 0.079 mmol). Yield: 4 mg (35%). ^{1}H NMR (400.13 MHz, CD_2Cl_2, 298 K): δ 8.93 (d, $^3J_{HH}$ = 8.8, 1H), 8.42-8.36 (m, 3H), 8.11-8.09 (m, 1H), 8.03-7.99 (m, 1H), 7.84-7.80 (m, 2H), 7.68-7.65 (m, 2H), 7.55-7.46 (m, 2H), 7.21-7.18 (m, 3H), 1.52 (d, $^3J_{HP}$ = 16.5, 9H), 1.22 (d, $^3J_{HP}$ = 16.5, 9H). ^{31}P{^{1}H} NMR (161.99 MHz, CD_2Cl_2, 298 K): δ 75.4 (s, *P*). (+)-HR-ESI-MS (electrospray, m/z): calcd for $C_{31}H_{33}DNAuP$ $[M]^+$, 649.21517; found, 649.21478.

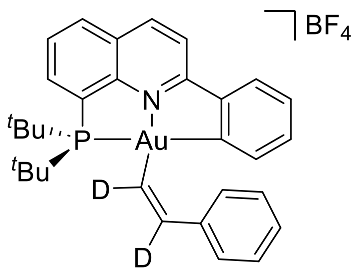

(P^N^C)Au-(*Z*)-(C(D)=C(D)Ph) (2-DD). Compound **2-DD** was synthesized following the general procedure using **1-D** (10.0 mg, 0.016 mmol) and phenylacetylene-d_1 (8.6 µL, 0.079 mmol). Yield: 4 mg (35%). ^{1}H NMR (400.13 MHz, CD_2Cl_2, 298 K): δ 8.93 (d, $^3J_{HH}$ = 8.8, 1H), 8.41-8.38 (m, 3H), 8.11-8.08 (m, 1H), 8.03-7.99 (m, 1H), 7.84-7.80 (m, 1H), 7.68-7.65 (m, 2H), 7.54-7.48 (m, 2H), 7.22-7.17 (m, 3H), 1.52 (d, $^3J_{HP}$ = 16.7, 9H), 1.22 (d, $^3J_{HP}$ = 16.7, 9H). ^{31}P{^{1}H} NMR (161.99 MHz, CD_2Cl_2, 298 K): δ 75.3 (s, *P*). (+)-

HR-ESI-MS (electrospray, m/z): calcd for $C_{31}H_{32}D_2NAuP$ $[M]^+$, 650.22144; found, 650.22178.

(P^N^C)Au-(*Z*)-(C(H)=C(H)C_6H_4OMe) (3). Compound **3** was synthesized following the general procedure using **1** (19.6 mg, 0.031 mmol) and 4-(methoxy)phenylacetylene (4 µL, 0.031 mmol). Yield: 17.7 mg (71%). ^{1}H NMR (400.13 MHz, CD_2Cl_2, 298 K): δ 8.94 (dd, $^3J_{HH}$ = 8.8, $^5J_{HP}$ = 1.4, 1H), 8.43-8.37 (m, 3H), 8.11 (ddd, $^3J_{HH}$ = 7.5, $^4J_{HH}$ = 3.6, $^4J_{HP}$ = 1.7, 1H), 8.01 (ddd, $^3J_{HH}$ = 7.8, $^3J_{HH}$ = 7.6, $^4J_{HP}$ = 1.7, 1H), 7.83-7.79 (m, 1H), 7.74 (dd, $^3J_{HH}$ = 9.7, $^4J_{HP}$ = 1.8, 1H), 7.62-7.58 (m, 2H), 7.54-7.43 (m, 3H), 6.75-6.71 (m, 2H), 3.69 (s, 3H), 1.53 (d, $^3J_{HP}$ = 16.4, 9H), 1.26 (d, $^3J_{HP}$ = 16.7, 9H). ^{13}C{^{1}H} NMR (100.62 MHz, CD_2Cl_2, 298 K): δ 166.3 (d, $^3J_{CP}$ = 6.0), 161.8 (d, $^2J_{CP}$ = 114.3), 159.7 (s), 150.5 (d, $^2J_{CP}$ = 10.9), 148.3 (s), 144.2 (s), 141.7 (d, $^2J_{CP}$ = 1.4), 134.3 (d, $^4J_{CP}$ = 2.1), 134.1 (d, overlapped), 134.0 (s), 131.9 (d, $^3J_{CP}$ = 2.8), 131.1 (s), 130.4 (d, $^3J_{CP}$ = 6.6), 129.8 (d, $^3J_{CP}$ = 6.5), 129.2 (s), 129.2 (s), 129.1 (d, $^4J_{CP}$ = 8.9), 128.9 (d, $^1J_{CP}$ = 35.7), 128.8 (d, $^4J_{CP}$ = 5.4), 119.9 (s), 114.3 (s), 55.4 (s), 39.3 (d, $^1J_{CP}$ = 15.8), 39.0 (d, $^1J_{CP}$ = 15.1), 29.8 (d, $^2J_{CP}$ = 3.9), 29.4 (d, $^2J_{CP}$ = 4.2). ^{31}P{^{1}H} NMR (161.99 MHz, CD_2Cl_2, 298 K): δ 75.1 (s, *P*). (+)-HR-ESI-MS (electrospray, m/z): calcd for $C_{32}H_{36}ONAuP$ $[M]^+$, 678.21945; found, 678.22016.

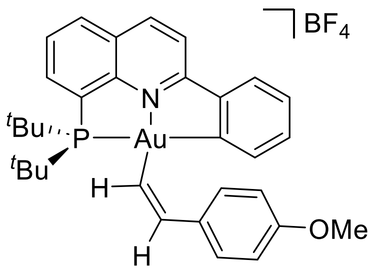

(P^N^C)Au-(*Z*)-(C(H)=C(H)C_6H_4^{t}Bu) (4). Compound **4** was synthesized following the general procedure using **1** (19.6 mg, 0.031 mmol) and 4-(*tert*-butyl)phenylacetylene (5.1 µL, 0.031 mmol). Yield: 16.0 mg (65%). ^{1}H NMR (500.30 MHz, CD_2Cl_2, 298 K): δ 8.95 (d, $^3J_{HH}$ = 8.9, 1H), 8.42 (d, $^3J_{HH}$ = 8.9, 1H), 8.40-8.36 (m, 2H), 8.14-8.11 (m, 1H), 8.01 (ddd, $^3J_{HH}$ = 7.8, $^3J_{HH}$ = 7.7, $^4J_{HP}$ = 1.0, 1H), 7.86 (dd, $^3J_{HP}$ = 7.5, $^3J_{HH}$ = 7.2, 1H), 7.79 (d, $^3J_{HH}$ = 9.8, 1H), 7.58 (d, $^3J_{HH}$ = 8.2, 2H), 7.55-7.50 (m, 3H), 7.22 (d, $^3J_{HH}$ = 8.3, 2H), 1.52 (d, $^3J_{HP}$ = 16.4, 9H), 1.19 (s, 9H), 1.19 (d, $^3J_{HP}$ = 16.4, 9H). ^{13}C{^{1}H} NMR (125.81 MHz, CD_2Cl_2, 298 K): δ 166.3 (d, $^3J_{CP}$ = 6.0, C^9), 161.9 (d, $^2J_{CP}$ = 114.6, C^{15}), 151.6 (s, C^{23}), 150.5 (d, $^2J_{CP}$ = 10.9), 148.3 (s), 144.3 (s), 141.6 (d, $^2J_{CP}$ = 1.2), 136.1 (s), 134.3 (d, $^4J_{CP}$ = 2.1), 134.2-134.2 (m), 132.3 (d, $^3J_{CP}$ = 2.7), 130.9 (d, ^3J = 8.7), 130.5 (d, $^3J_{CP}$ = 6.5), 129.9 (d, $^3J_{CP}$ = 6.5), 129.3 (s), 129.0 (d, $^1J_{CP}$ = 35.9), 128.8 (d, $^4J_{CP}$ = 5.5), 127.6 (s), 125.9 (s), 120.0 (s), 39.3 (d, $^1J_{CP}$ = 15.6), 39.0 (d, $^1J_{CP}$ = 14.9), 34.9 (s), 31.2 (s), 29.8 (d, $^2J_{CP}$ = 4.0), 29.4 (d, $^2J_{CP}$ = 4.2). ^{31}P{^{1}H} NMR (161.99 MHz, CD_2Cl_2, 298 K): δ 75.2 (s, *P*). (+)-HR-ESI-MS (electrospray, m/z): calcd for $C_{35}H_{42}NAuP$ $[M]^+$, 704.27149; found, 704.27274.

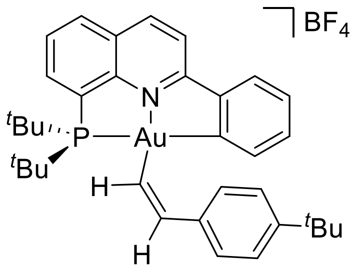

(P^N^C)Au-(*Z*)-(C(H)=C(H)$C_6H_4CF_3$) (5). Compound **5** was synthesized following the general procedure using **1** (19.6 mg, 0.031 mmol) and 4-(trifluoromethyl)phenylacetylene (5.1 µL, 0.031 mmol). Yield: 16.0 mg (64%). ^{1}H NMR (500.13 MHz, CD_2Cl_2, 298 K): δ 8.97 (d, $^3J_{HH}$ = 8.8, $^5J_{HP}$ = 1.3, 1H), 8.43 (d, $^3J_{HH}$ = 9.0, 1H), 8.42-8.38 (m, 2H), 8.12 (ddd, $^3J_{HH}$ = 7.7, $^4J_{HH}$ = 3.6, $^4J_{HP}$ = 1.3, 1H), 8.03 (ddd, $^3J_{HH}$ = 7.8, $^3J_{HH}$ = 7.7, $^4J_{HP}$ = 1.4, 1H), 7.88 (dd, $^3J_{HH}$ = 10.1, $^4J_{HP}$ = 1.5, 1H), 7.82-7.76 (m, 4H), 7.54 (dd, $^3J_{HH}$ = 7.5, $^3J_{HH}$ = 7.2, 1H), 7.50-7.46 (m, 3H), 1.53 (d, $^3J_{HP}$ = 16.5, 9H), 1.22 (d, $^3J_{HP}$ = 16.5, 9H). ^{13}C{^{1}H} NMR (100.62 MHz, CD_2Cl_2, 298 K): δ 166.5 (d, $^3J_{CP}$ = 6.0), 161.4 (d, $^2J_{CP}$ = 112.4), 150.6 (d, $^2J_{CP}$ = 10.9), 148.3 (s), 144.5 (s), 142.3 (s), 141.7 (d, $^2J_{CP}$ = 1.1), 134.6 (d, $^2J_{CP}$ = 2.7), 134.5 (d, $^4J_{CP}$ = 2.2), 134.3 (d, $^4J_{CP}$ = 7.9), 133.9 (d, $^3J_{CP}$ = 1.0), 131.6 (d, $^3J_{CP}$ = 2.7), 130.5 (d, $^3J_{CP}$ = 6.6), 130.0 (d, $^3J_{CP}$ = 6.6), 129.7 (q, $^2J_{CF}$ = 32.6), 129.5 (s), 129.1 (d, $^4J_{CP}$ = 5.3), 128.6 (d, $^1J_{CP}$ = 36.2), 128.2 (s), 125.9 (q, $^3J_{CF}$ = 3.9), 124.5 (q, $^1J_{CF}$ = 272.3), 120.1 (s), 39.5 (d, $^1J_{CP}$ = 15.6), 39.0 (d, $^1J_{CP}$ = 15.2), 29.8 (d, $^2J_{CP}$ = 3.9), 29.5 (d, $^2J_{CP}$ = 4.1). ^{19}F{^{1}H} NMR (470.71 MHz, CD_2Cl_2, 298 K): δ -63.2 (s, C*F*$_3$), -153.0 (br, B*F*$_4$). ^{31}P{^{1}H} NMR (202.52 MHz, CD_2Cl_2, 298 K): δ 76.1 (s, *P*). (+)-HR-ESI-MS (electrospray, m/z): calcd for $C_{32}H_{33}NAuF_3P$ $[M]^+$, 716.19627; found, 716.19491.

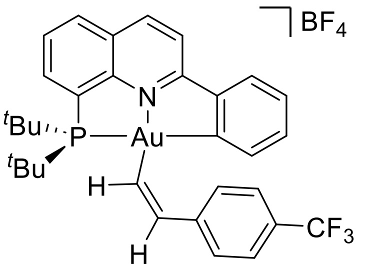

(P^N^C)Au-(*Z*)-(C(H)=C(H)^{n}Bu) (6). Compound **6** was synthesized following the general procedure using **1** (19.6 mg, 0.031 mmol) and 1-hexyne (3.6 µL, 0.031 mmol). Yield: 18.3 mg (82%). ^{1}H NMR (400.13 MHz, CD_2Cl_2, 298 K): δ 8.89 (dd, $^3J_{HH}$ = 8.8, $^5J_{HP}$ = 1.3, 1H), 8.42-8.34 (m, 3H), 8.07 (ddd, $^3J_{HH}$ = 7.5, $^5J_{HP}$ = 3.5, $^4J_{HH}$ = 1.7, 1H), 8.01 (ddd, $^3J_{HH}$ = 8.0, $^3J_{HH}$ = 7.6, $^4J_{HP}$ = 1.7, 1H), 7.87 (ddd, $^4J_{HP}$ = 7.7, $^3J_{HH}$ = 7.5, $^4J_{HH}$ = 1.3, 1H), 7.60-7.51 (m, 2H), 7.20 (ddt, $^3J_{HH}$ = 8.1, $^3J_{HP}$ = 5.6, $^4J_{HH}$ = 1.3, 1H), 6.73 (dtd, $^3J_{HH}$ = 7.6, $^3J_{HH}$ = 6.9, $^4J_{HP}$ = 1.8, 1H), 2.11 (m, 2H), 1.52 (d, $^3J_{HP}$ = 16.5, 9H), 1.51 (d, $^3J_{HP}$ = 16.4, 9H), 1.24 (s, 4H), 0.82 (t, $^3J_{HH}$ = 7.3, 3H). ^{13}C{^{1}H} NMR (100.62 MHz, CD_2Cl_2, 298 K): δ 166.0 (d, $^3J_{CP}$ = 6.1), 161.7 (d, $^2J_{CP}$ = 116.0), 150.4 (d, $^2J_{CP}$ = 10.9), 148.5 (s), 143.9 (s), 141.4 (d, $^3J_{CP}$ = 1.6), 134.3 (d, $^4J_{CP}$ = 2.2), 134.3 (d, $^3J_{CP}$ = 1.0), 134.2 (d, $^3J_{CP}$ = 2.2), 133.7 (d, $^4J_{CP}$ = 7.9), 131.3 (d, $^2J_{CP}$ = 8.4), 130.3 (d, $^3J_{CP}$ = 6.6), 129.8 (d, $^3J_{CP}$ = 6.5), 129.2 (s), 129.0 (d, $^1J_{CP}$ = 36.0), 128.7 (d, $^4J_{CP}$ = 5.5), 119.8 (s), 39.2 (d, $^1J_{CP}$ = 15.6), 38.4 (d, $^4J_{CP}$ = 1.0), 31.2 (s), 29.9 (d, $^2J_{CP}$ = 4.1), 29.8 (d, $^2J_{CP}$ = 4.0). 23.0 (s), 14.1 (s). ^{31}P{^{1}H} NMR (161.99 MHz, CD_2Cl_2, 298 K): δ 72.8 (s, *P*). (+)-HR-ESI-MS (electrospray, m/z): calcd for $C_{29}H_{38}NAuP$ $[M]^+$, 628.24019; found, 628.23953.

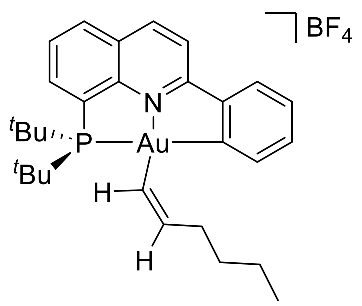

(P^N^C)Au-(*Z*)-(C(H)=C(H)^{t}Bu) (7). Compound **7** was synthesized following the general procedure using **1** (19.6 mg, 0.031 mmol) and 3,3-dimethyl-1-butyne (3.8 μL, 0.031 mmol) heating at 50 ºC. Yield: 11.7 mg (53%). ^{1}H NMR (400.13 MHz, CD_2Cl_2, 298 K): δ 8.89 (dd, $^3J_{HH}$ = 8.8, $^5J_{HP}$ = 1.2, 1H), 8.41 (dd, $^3J_{HH}$ = 7.6, $^3J_{HP}$ = 7.5, 1H), 8.38-8.34 (m, 2H), 8.07-8.04 (m, 1H), 8.02 (ddd, $^3J_{HH}$ = 7.8, $^3J_{HH}$ = 7.6, $^4J_{HP}$ = 1.5, 1H), 7.94-7.90 (m, 1H), 7.59-7.51 (m, 2H), 7.19 (dd, $^3J_{HH}$ = 9.8, $^3J_{HP}$ = 5.3, 1H), 6.70 (dd, $^3J_{HH}$ = 9.8, $^4J_{HP}$ = 2.0, 1H), 1.57 (d, $^3J_{HP}$ = 16.5, 9H), 1.50 (d, $^3J_{HP}$ = 16.3, 9H), 1.14 (s, 9H). ^{13}C{^{1}H} NMR (100.62 MHz, CD_2Cl_2, 298 K): δ 166.1 (d, $^3J_{CP}$ = 6.2), 163.2 (d, $^2J_{CP}$ = 117.2), 150.4 (d, $^2J_{CP}$ = 11.1), 147.9 (s), 143.9 (s), 143.3 (d, $^3J_{CP}$ = 2.2), 141.4 (d, $^3J_{CP}$ = 1.7), 135.0 (s), 134.2 (d, $^4J_{CP}$ = 2.1), 133.7 (d, $^4J_{CP}$ = 7.9), 130.3 (d, $^3J_{CP}$ = 6.5), 129.8 (d, $^3J_{CP}$ = 6.4), 129.2 (s), 129.1 (d, $^1J_{CP}$ = 35.4), 128.7 (d, $^4J_{CP}$ = 5.5), 123.0 (d, $^2J_{CP}$ = 8.2), 119.8 (s), 39.4 (d, $^1J_{CP}$ = 15.8), 39.0 (d, $^1J_{CP}$ = 14.3), 34.1 (s), 30.3 (d, $^2J_{CP}$ = 4.0), 29.8 (s), 29.7 (d, $^2J_{CP}$ = 4.0). ^{31}P{^{1}H} NMR (161.99 MHz, CD_2Cl_2, 298 K): δ 73.3 (s, *P*). (+)-HR-ESI-MS (electrospray, m/z): calcd for $C_{29}H_{38}NAuP$ $[M]^+$, 628.24019; found, 628.23958.

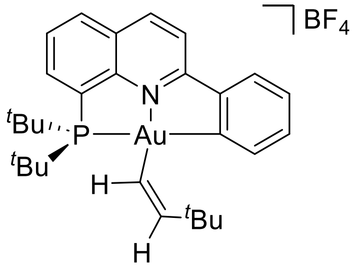

(P^N^C)Au-(*Z*)-(C(CO_2Me)=C(H)CO_2Me) (8). Compound **8** was synthesized following the general procedure using **1** (19.6 mg, 0.031 mmol) and dimethyl acetylenedicarboxylate (3.8 μL, 0.031 mmol). Yield: 14.6 mg (61%). ^{1}H NMR (400.13 MHz, CD_2Cl_2, 298 K): δ 8.93 (dd, $^3J_{HH}$ = 8.8, $^5J_{HP}$ = 1.4, 1H), 8.42 (ddd, $^3J_{HP}$ = 7.7, $^3J_{HH}$ = 7.6, $^4J_{HH}$ = 1.0, 1H), 8.40-8.38 (m, 1H), 8.36 (d, $^3J_{HH}$ = 8.9, 1H), 8.06-8.02 (m, 2H), 7.87 (d, $^4J_{HP}$ = 2.7, 1H), 7.55-7.46 (m, 2H), 7.36-7.32 (m, 1H), 3.85 (s, 3H), 3.67 (s, 3H), 1.45 (d, $^3J_{HP}$ = 16.6, 9H), 1.45 (d, $^3J_{HP}$ = 16.5, 9H). ^{13}C{^{1}H} NMR (100.62 MHz, CD_2Cl_2, 298 K): δ 168.9 (s), 168.9 (s), 167.3 (d, $^3J_{CP}$ = 6.2), 162.0 (d, $^2J_{CP}$ = 111.5), 155.2 (d, $^2J_{CP}$ = 8.9), 150.7 (d, $^2J_{CP}$ = 11.1), 147.4 (s), 144.5 (s), 141.5 (s), 134.5 (s), 134.4 (d, $^4J_{CP}$ = 7.8), 132.3 (s), 132.2 (d, $^3J_{CP}$ = 2.8), 130.2 (d, $^3J_{CP}$ = 6.7), 130.0 (d, $^3J_{CP}$ = 6.5), 129.3 (s), 129.0 (d, $^4J_{CP}$ = 5.1), 128.5 (d, $^1J_{CP}$ = 36.8), 120.0 (s), 53.5 (s, overlapped), 52.8 (s), 40.1 (d, $^1J_{CP}$ = 14.2), 39.2 (d, $^1J_{CP}$ = 15.6), 29.6 (d, $^2J_{CP}$ = 3.9), 29.3 (d, $^2J_{CP}$ = 4.1). ^{31}P{^{1}H} NMR (161.99 MHz, CD_2Cl_2, 298 K): δ 78.2 (s, *P*). (+)-HR-ESI-MS (electrospray, m/z): calcd for $C_{29}H_{34}O_4NAuP$ $[M]^+$, 688.18855; found, 688.18735.

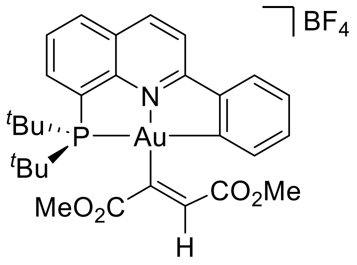

(P^N^C)Au-(*Z*)-(C($CO_2{}^t$Bu)=C(H)$CO_2{}^t$Bu) (9). Compound **9** was synthesized following

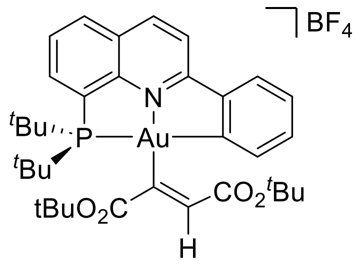

the general procedure using **1** (19.6 mg, 0.031 mmol) and di-*tert*-butyl acetylenedicarboxylate (7.0 mg, 0.031 mmol). Yield: 17.5 mg (66%). ^{1}H NMR (400.13 MHz, CD_2Cl_2, 298 K): δ 8.91 (dd, $^3J_{HH}$ = 8.9, $^5J_{HP}$ = 1.4, 1H), 8.42 (ddd, $^3J_{HP}$ = 7.6, $^3J_{HH}$ = 7.5, $^4J_{HH}$ = 1.0, 1H), 8.38-8.34 (m, 2H), 8.05-8.01 (m, 2H), 7.66 (d, $^4J_{HP}$ = 2.6, 1H), 7.53-7.47 (m, 2H), 7.38-7.34 (m, 1H), 1.48 (d, $^3J_{HP}$ = 16.4, 9H), 1.48 (s, 9H), 1.47 (d, $^3J_{HP}$ = 16.4, 9H), 1.36 (s, 9H). ^{13}C{^{1}H} NMR (100.62 MHz, CD_2Cl_2, 298 K): δ 167.8 (s), 167.8 (s), 167.2 (d, $^3J_{CP}$ = 6.2), 162.2 (d, $^2J_{CP}$ = 112.5), 156.5 (d, $^2J_{CP}$ = 9.1), 150.7 (d, $^2J_{CP}$ = 11.2), 147.4 (s), 144.3 (s), 141.3 (d, $^2J_{CP}$ = 1.4), 134.3 (d, $^4J_{CP}$ = 2.2), 134.2 (d, $^4J_{CP}$ = 8.0), 133.1 (d, $^3J_{CP}$ = 2.8), 132.3 (d, ^3J = 1.2), 130.2 (d, $^3J_{CP}$ = 6.8), 129.9 (d, $^3J_{CP}$ = 6.3), 128.8 (d, $^1J_{CP}$ = 36.3), 129.1 (s), 128.7 (d, $^4J_{CP}$ = 5.2), 120.0 (s), 83.8 (s), 82.4 (s), 40.1 (d, $^1J_{CP}$ = 15.6), 39.1 (d, $^1J_{CP}$ = 15.2), 29.6 (d, $^2J_{CP}$ = 4.4), 29.6 (d, $^2J_{CP}$ = 4.2), 28.1 (s), 28.1 (s). ^{31}P{^{1}H} NMR (161.99 MHz, CD_2Cl_2, 298 K): δ 76.9 (s, *P*). (+)-HR-ESI-MS (electrospray, m/z): calcd for $C_{35}H_{46}O_4NAuP$ $[M]^+$, 772.28301; found, 772.28238. Single crystals of **9** suitable for X-ray diffraction analysis were obtained by slow vapor diffusion of pentane into a solution of the compound in dichloromethane at low temperature.

(P^N^C)Au-(*Z*)-(C(CO_2Me)=C(H)CO_2Me) (10). Compound **10** was synthesized

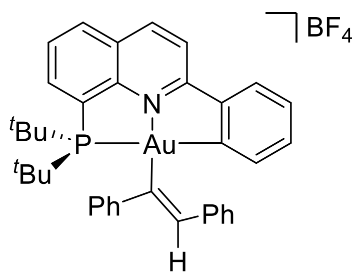

following the general procedure using **1** (20.0 mg, 0.032 mmol) and diphenyl acetylenedicarboxylate (5.6 mg, 0.032 mmol). Yield: 15.2 mg (59%). ^{1}H NMR (400.13 MHz, CD_2Cl_2, 298 K): δ 8.98 (dd, $^3J_{HH}$ = 8.8, $^5J_{HP}$ = 1.3, 1H), 8.48 (d, $^3J_{HH}$ = 8.9, 1H), 8.38 (d, $^3J_{HH}$ = 8.1), 8.31 (ddd, $^3J_{HH}$ = 7.6, $^3J_{HP}$ = 7.5, $^4J_{HH}$ = 1.0, 1H), 8.19 (ddd, $^3J_{HH}$ = 7.9, $^5J_{HP}$ = 3.6, $^4J_{HH}$ = 1.4, 1H), 7.98 (ddd, $^3J_{HH}$ = 7.8, $^3J_{HH}$ = 6.4, $^5J_{HP}$ = 1.4, 1H), 7.94 (ddd, $^3J_{HH}$ = 7.8, $^4J_{HP}$ = 6.0, $^4J_{HH}$ = 1.2, 1H), 7.82 (d, $^4J_{HP}$ = 1.7, 1H), 7.77-7.74 (m, 2H), 7.66-7.63 (m, 2H), 7.59 (dd, $^3J_{HH}$ = 7.7, $^3J_{HH}$ = 7.5, 1H), 7.53-7.48 (m, 1H), 7.39-7.29 (m, 3H), 7.21-7.12 (m, 3H), 1.21 (d, $^3J_{HP}$ = 16.5, 9H), 1.03 (d, $^3J_{HP}$ = 16.3, 9H). ^{13}C{^{1}H} NMR (100.62 MHz, CD_2Cl_2, 298 K): δ 166.3 (d, $^3J_{CP}$ = 6.2), 160.8 (d, $^2J_{CP}$ = 113.4), 150.4 (d, $^2J_{CP}$ = 10.5), 149.7 (d, $^2J_{CP}$ = 7.7), 148.0 (s), 145.2 (d, $^4J_{CP}$ = 1.0), 144.4 (s), 141.6 (d, $^2J_{CP}$ = 1.5), 139.2 (s), 135.3 (d, $^3J_{CP}$ = 1.0), 134.5 (d, $^4J_{CP}$ = 7.9), 134.2 (d, $^4J_{CP}$ = 2.1), 131.1 (d, $^3J_{CP}$ = 2.7), 130.4 (d, $^3J_{CP}$ = 6.2), 129.7 (d, $^3J_{CP}$ = 6.4), 129.5 (s), 129.3 (s), 129.2 (s), 129.2 (d overlapped, $^1J_{CP}$ = 36), 129.1 (s), 129.1 (s), 128.6 (s), 128.4 (s), 128.1 (s), 120.0 (s), 39.0 (d, $^1J_{CP}$ = 15.0), 38.7 (d, $^1J_{CP}$ = 13.9), 29.4 (d, $^2J_{CP}$ = 4.2), 28.9 (d, $^2J_{CP}$ = 4.2). ^{31}P{^{1}H}

NMR (161.99 MHz, CD_2Cl_2, 298 K): δ 75.8 (s, *P*). (+)-HR-ESI-MS (electrospray, m/z): calcd for $C_{37}H_{38}NAuP$ $[M]^+$, 724.24019; found, 740.23994.

(P^N^C)Au-(*Z*)-(C(allyl)=C(H)allyl) (11). Compound **11** was synthesized following the general procedure using **1** (19.6 mg, 0.031 mmol) and diallyl acetylenedicarboxylate (6.0 mg, 0.031 mmol). Yield: 15.4 mg (61%). ^1H NMR (400.13 MHz, CD_2Cl_2, 298 K): δ 8.92 (dd, $^3J_{HH}$ = 8.8, $^5J_{HP}$ = 1.3, 1H), 8.43-8.37 (m, 2H), 8.35 (d, $^3J_{HH}$ = 8.8, 1H), 8.05-8.02 (m, 2H), 7.91 (d, $^4J_{HP}$ = 2.6, 1H), 7.56-7.47 (m, 2H), 7.36 (ddd, $^4J_{HP}$ = 7.7, $^3J_{HH}$ = 7.4, $^4J_{HH}$ = 1.3, 1H), 5.99-5.78 (m, 2H), 5.34-5.18 (m, 4H), 4.85-4.49 (m, 4H),1.45 (d, $^3J_{HP}$ = 16.7, 9H), 1.44 (d, $^3J_{HP}$ = 16.5, 9H). $^{13}C\{^1H\}$ NMR (125.81 MHz, CD_2Cl_2, 298 K): δ 168.2 (s), 168.1 (s), 167.4 (d, $^3J_{CP}$ = 6.1), 162.0 (d, $^2J_{CP}$ = 111.8), 155.8 (d, $^2J_{CP}$ = 9.1), 150.8 (d, $^2J_{CP}$ = 11.1), 147.4 (s), 144.5 (s), 141.5 (d, $^2J_{CP}$ = 1.3), 134.5 (s), 134.5 (d, $^4J_{CP}$ = 7.9), 132.4 (s), 132.3 (d, $^3J_{CP}$ = 3.1), 131.8 (s), 131.7 (s), 130.3 (d, $^3J_{CP}$ = 6.8), 130.0 (d, $^3J_{CP}$ = 6.5), 129.4 (s), 129.0 (d, $^4J_{CP}$ = 5.3), 128.6 (d, $^1J_{CP}$ = 36.6), 120.0 (s), 119.8 (s), 119.1 (s), 67.5 (s), 66.5 (s), 40.1 (d, $^1J_{CP}$ = 14.4), 39.3 (d, $^1J_{CP}$ = 15.5), 29.6 (d, $^2J_{CP}$ = 3.9), 29.5 (d, $^2J_{CP}$ = 4.4). $^{31}P\{^1H\}$ NMR (161.99 MHz, CD_2Cl_2, 298 K): δ 78.3 (s, *P*). (+)-HR-ESI-MS (electrospray, m/z): calcd for $C_{33}H_{38}O_4NAuP$ $[M]^+$, 740.21985; found, 740.22009.

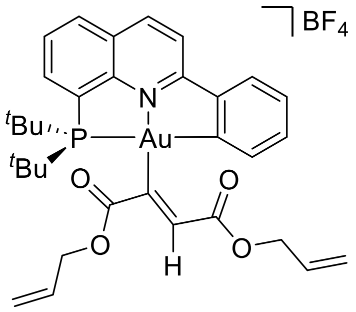

3. Mechanistic investigations

3.1 Effect of counterion and solvent

Control experiments were conducted to determine the effect of the solvent and the counterion in the selectivity of the reaction.

The reaction of **1** with phenylacetylene in acetone-*d*6 showed complete conversion to **2** (Figure S1a). Unfortunately, the cationic nature of **1** limits its solubility in less polar solvents. To address this issue, counterion exchange was performed to replace BF_4 by B_{Ar}^F and PF_6. As expected, both **1-B_{Ar}^F** and **1-PF_6** reacted with phenylacetylene in dichloromethane to yield **2-B_{Ar}^F** and **2-PF_6**, respectively (Figure S2). Then, other solvents were tested. Compound **1-B_{Ar}^F** becomes only partially soluble in less polar solvents such as toluene-*d*8. Nevertheless, after the addition of phenylacetylene, the insertion reaction occurred immediately, with the product **2-B_{Ar}^F** being fully soluble in toluene (Figure S1b).

These experiments exclusively produced the *Z*-isomer, indicating that neither the solvent nor the counterion plays a role in the final stereoselectivity of the gold-vinyl products.

<u>Solvents</u>. The insertion reaction was explored in other solvents than dichloromethane.

- *Acetone.* Phenylacetylene (4.3 μL, 0.039 mmol) was added to an NMR tube containing a solution of **1** (5 mg; 0.0079 mmol) in 0.5 mL of acetone-*d*6. After 15 minutes, the $^{31}P\{^1H\}$ NMR spectrum showed complete consumption of **1** and clean formation of **2** (Figure S1a).
- *Toluene.* Phenylacetylene (0.8 μL, 0.007 mmol) was added to an NMR tube containing a suspension of **1-B_{Ar}^F** (10 mg; 0.007 mmol) in 0.5 mL of toluene-*d*8. After 15 minutes, the suspension was transformed into a clear solution and the $^{31}P\{^1H\}$ NMR spectrum showed complete consumption of **1-B_{Ar}^F** and clean formation of **2-B_{Ar}^F** (Figure S1b).

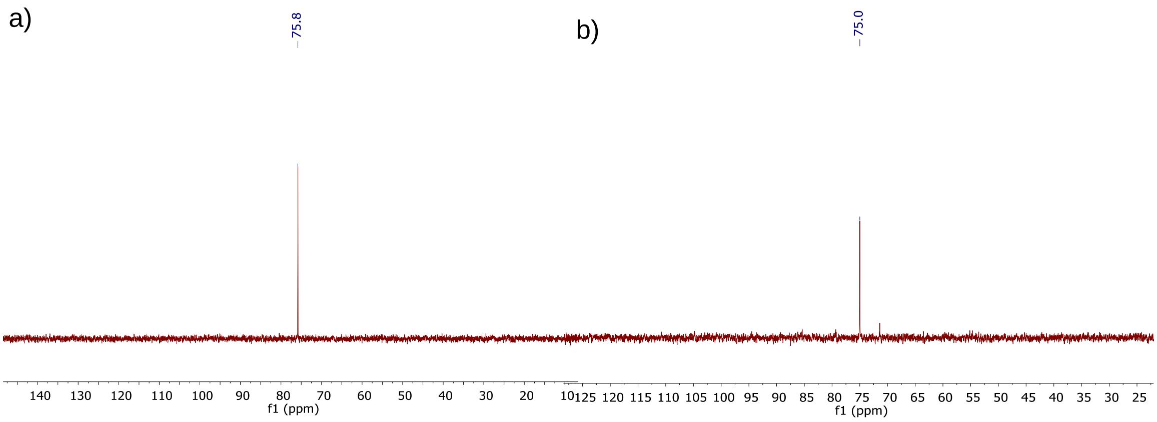

Figure S1. $^{31}P\{^1H\}$ NMR spectra (161.99 MHz) showing the conversion of **1** into **2** in a) acetone-*d*6, b) toluene-*d*8. (using **1-B_{Ar}^F**).

Counterions. To explore the effect of other counterions in the insertion reaction, BF_4 was replaced with B_{Ar}^F and PF_6. using NaB_{Ar}^F (B_{Ar}^F = Tetrakis(3,5-bis(trifluoromethyl)phenyl)borate) and KPF_6, respectively.

- For the reaction of **1-B_{Ar}^F**, phenylacetylene (0.8 µL, 0.007 mmol) was added to an NMR tube containing a solution of **1-B_{Ar}^F** (10 mg; 0.007 mmol) in 0.5 mL of CD_2Cl_2. After 15 minutes, the $^{31}P\{^1H\}$ NMR spectrum showed the complete consumption of starting material and the clean formation of **2-B_{Ar}^F** (Figure S2a).
- For the reaction of **1-PF_6**, phenylacetylene (1.5 µL, 0.015 mmol) was added to an NMR tube containing a solution of **1-PF_6** (10 mg; 0.015 mmol) in 0.5 mL of CD_2Cl_2. After 15 minutes, the $^{31}P\{^1H\}$ NMR spectrum showed the complete consumption of starting material and the clean formation of **2-PF_6** (Figure S2b).

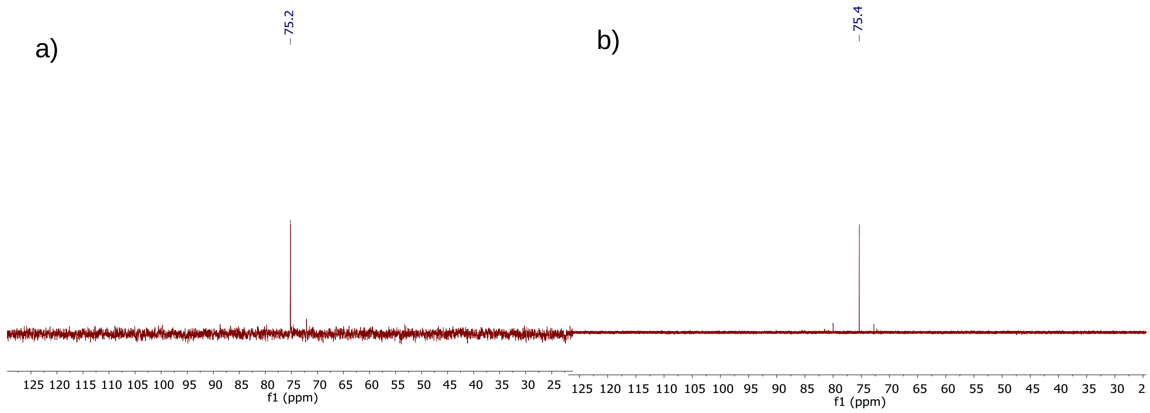

Figure S2. $^{31}P\{^1H\}$ NMR spectra (161.99 MHz, in CD_2Cl_2) showing the conversion of a) **1-B_{Ar}^F** into **2-B_{Ar}^F**, b) **1-PF_6** into **2-PF_6**.

3.2 Radical trapping experiments

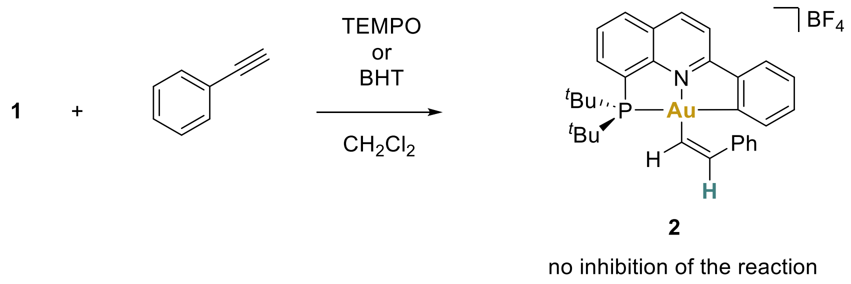

Scheme S1. Radical trapping experiments in presence of TEMPO or BHT.

TEMPO. 2,2,6,6-Tetramethylpiperidinyloxy (TEMPO) (4.8 mg, 0.031 mmol) was added to an NMR tube containing a solution of **1** (19.6 mg; 0.031 mmol) in 0.5 mL of CD_2Cl_2. Subsequently, phenylacetylene (3.4 μL, 0.031 mmol) was added. After 30 minutes, the $^{31}P\{^1H\}$ NMR spectrum showed complete consumption of **1** and predominant formation of **2**, along with some unidentified minor species (Figure S3). It has been reported that TEMPO can react with certain metal-hydrides to form a metal-TEMPO adduct, accompanied by elimination of molecular hydrogen.[6] Although we were unable to identify such species in the reaction mixture, the formation of compound **2** suggests that TEMPO does not inhibit the insertion reaction with alkynes.

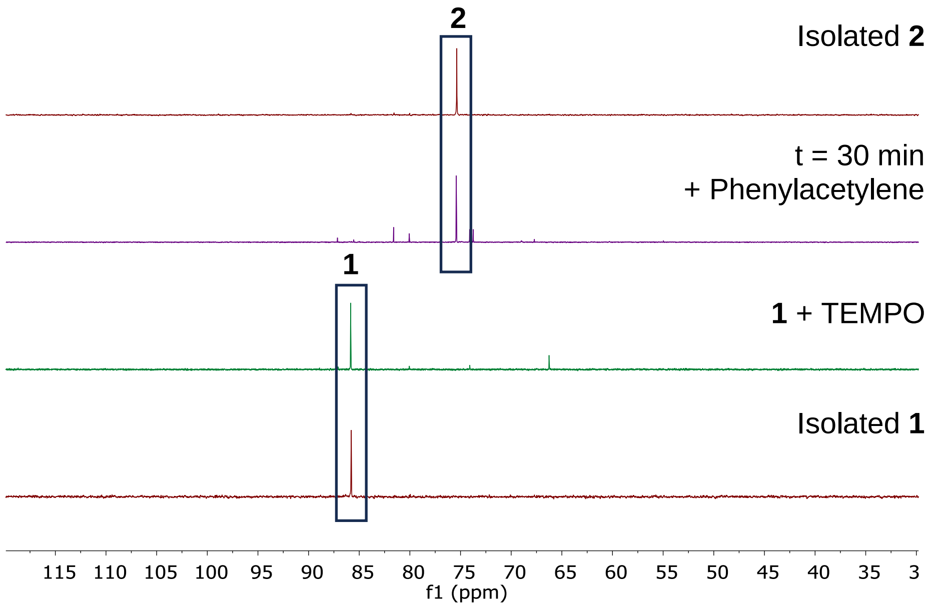

Figure S3. Stacked $^{31}P\{^1H\}$ NMR spectra (161.99 MHz, in CD_2Cl_2) showing the conversion of **1** into **2** after addition of phenylacetylene in presence of TEMPO.

BHT. Butylated hydroxytoluene (BHT) (6.8 mg, 0.031 mmol) was added to an NMR tube containing a solution of **1** (19.6 mg; 0.031 mmol) in 0.5 mL of CD_2Cl_2. Subsequently, phenylacetylene (3.4 µL, 0.031 mmol) was added. After 30 minutes, the $^{31}P\{^1H\}$ NMR spectrum showed complete consumption of **1** and formation of **2** (Figure S4). As in the experiment with TEMPO, BHT did not significantly affect the formation of gold-vinyl species.

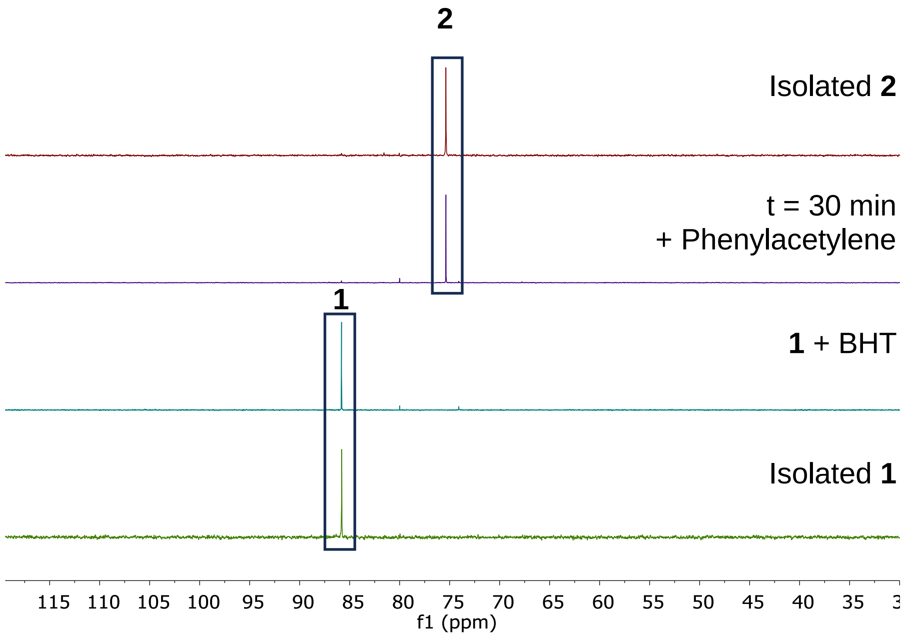

Figure S4. Stacked $^{31}P\{^1H\}$ NMR spectra (161.99 MHz, in CD_2Cl_2) showing the conversion of **1** into **2** after addition of phenylacetylene in presence of BHT.

BHT-*d*₁ and ethylbenzene-*d*₁₀. In order to rule out the presence of vinyl radical intermediates, the insertion reaction was carried out in the presence of molecules with low bond dissociation energy (BDE) for the carbon-deuterium bond, such as BHT-d_1 and ethylbenzene-d_{10} (Scheme S2), that have been reported to participate as substrates in hydrogen atom abstraction (HAA) reactions.[7]

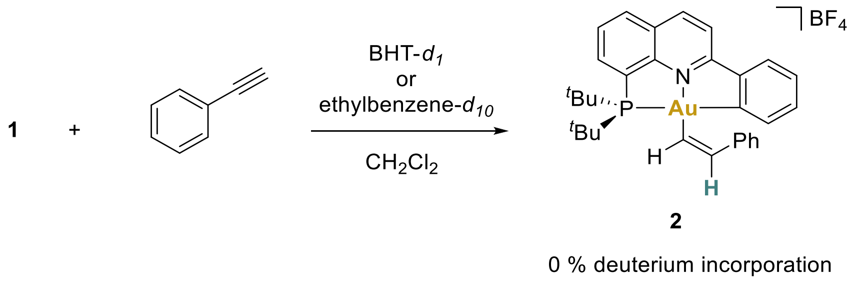

Scheme S2. Reaction of **1** with phenylacetylene in presence of BHT-d_1 and ethylbenzene-d_{10}.

- BHT-d_1 (6.8 mg, 0.031 mmol) was added to an NMR tube containing a solution of **1** (19.6 mg; 0.031 mmol) in 0.5 mL of CD_2Cl_2. Subsequently, phenylacetylene (3.4 μL, 0.031 mmol) was added. The vinyl complex **2** was isolated as described in the experimental section.
- Ethylbenzene-d_{10} (3.8 μL, 0.031 mmol) was added to an NMR tube containing a solution of **1** (19.6 mg; 0.031 mmol) in 0.5 mL of CD_2Cl_2. Subsequently, phenylacetylene (3.4 μL, 0.031 mmol) was added. The vinyl complex **2** was isolated as described in the experimental section.

In both cases, the ^{1}H NMR spectra displayed consistent integration of all signals, indicating non detectable incorporation of deuterium.

Dark conditions. To further exclude the presence of radical species the insertion reaction was conducted in absence of any source of light. Phenylacetylene (3.4 μL, 0.031 mmol) was added to an NMR tube covered with aluminium foil containing a solution of **1** (19.6 mg; 0.031 mmol) in 0.5 mL of CD_2Cl_2. After 15 minutes, the $^{31}P\{^1H\}$ NMR spectrum showed complete consumption of **1** and clean formation of **2**.

Radical clock experiment. The reaction of **1** with diallyl acetylenedicarboxylate (DAAD) was carried out as described in the experimental section and complex **11** was isolated accordingly. No cyclized product was observed (Scheme 3).

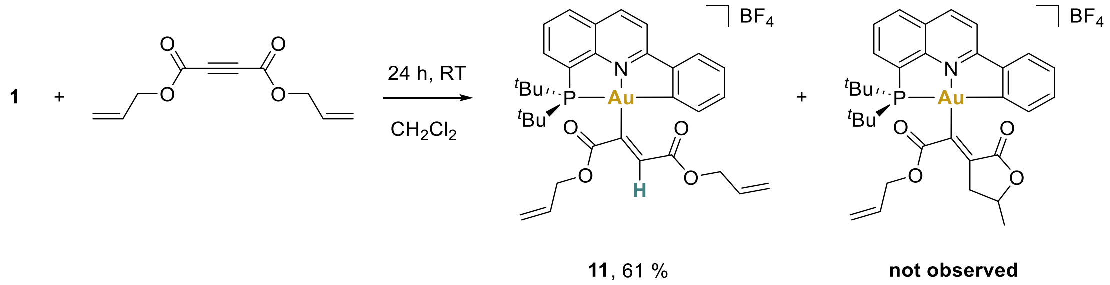

Scheme S3. Reaction of **1** with DAAD.

3.3 Isomerization experiments

Isomerization of compound 2

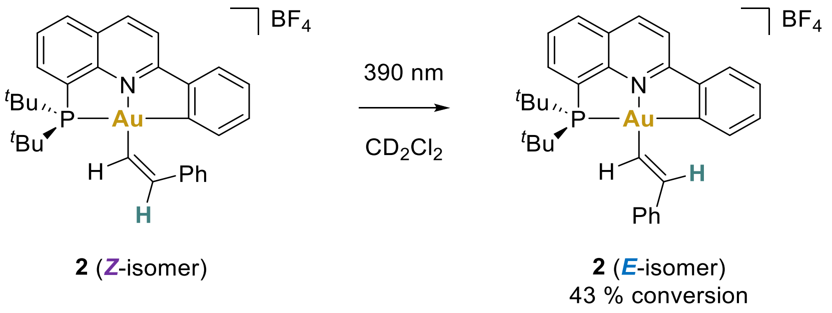

Scheme S4. Isomerization of compound **2** under blue light.

The photoisomerization of compound **2** (Scheme S4) was monitored by $^{31}P\{^{1}H\}$ NMR spectroscopy. A solution of **2-*Z*** (22.8 mg, 0.031 mmol) in 0.5 mL of CD_2Cl_2 was placed in an NMR tube and illuminated with a Kessil lamp at 390 nm over time. After 20 min of illumination, a mixture of isomers *Z/E* (57/43) was observed. Prolonged exposure to this light resulted in the activation of the solvent, leading to the formation of [Au]-Cl species, yet the *Z/E* ratio remained unchanged. Conversely, in the absence of light, a sample containing the mixture of isomers *Z/E* (57/43) exhibited no change over time, maintaining the same ratio of isomers (Figure S5-6).

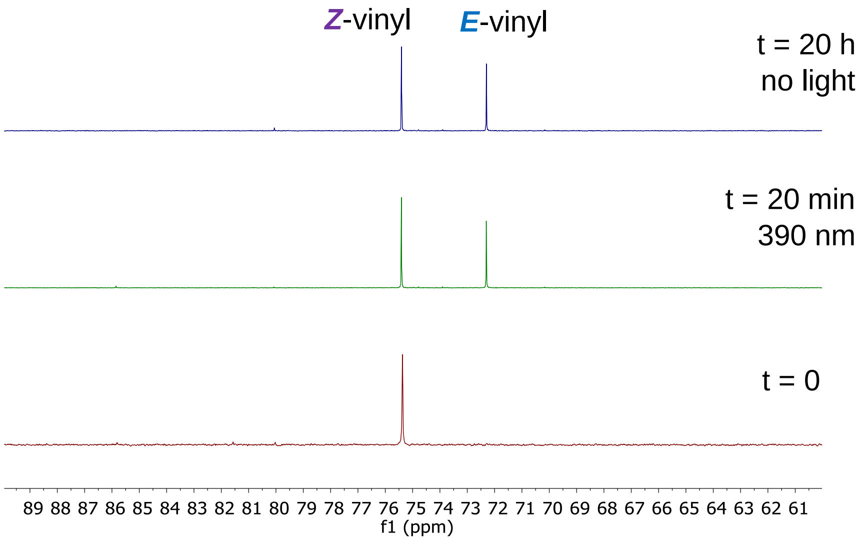

Figure S5. Stacked $^{31}P\{^{1}H\}$ NMR spectra (161.99 MHz, in CD_2Cl_2) showing the isomerization of compound **2** after illumination at 390 nm for 20 min, and the evolution in absence of light for 20 h.

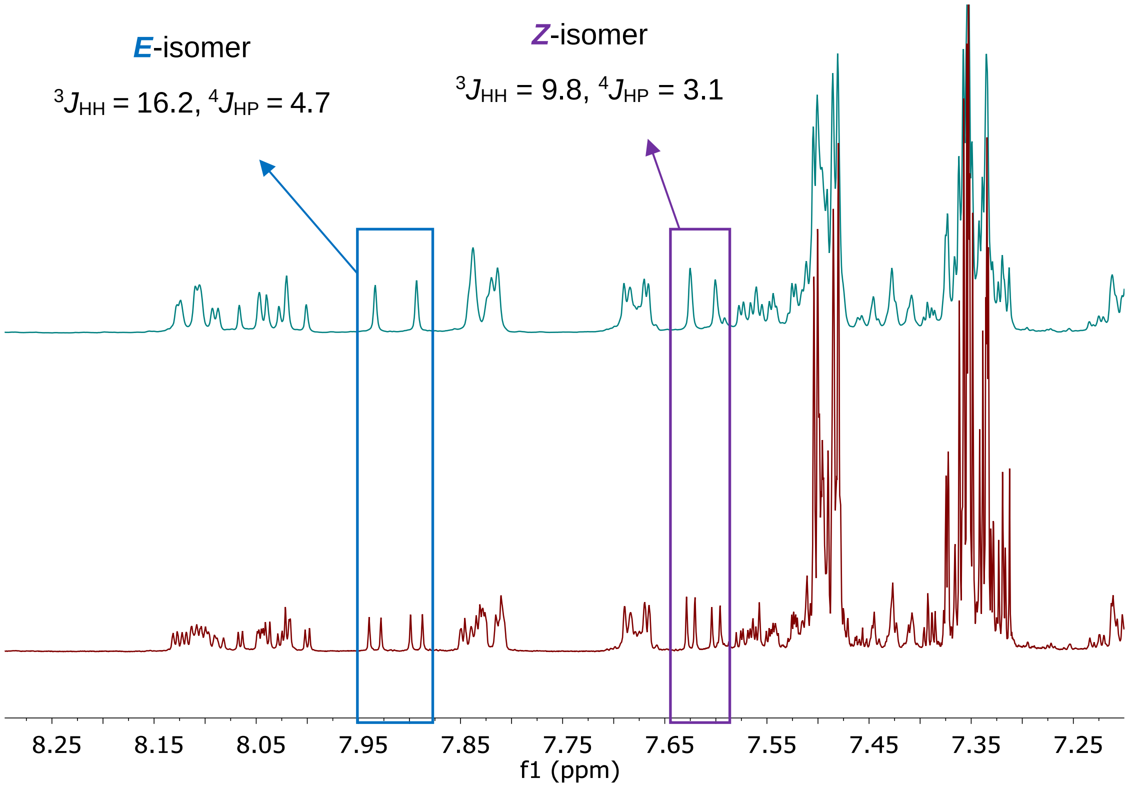

Figure S6. Comparison between $^{1}H\{^{31}P\}$ (top) and ^{1}H (bottom) NMR spectra (400.13 MHz, in CD_2Cl_2) showing the isomerization of compound **2** after illumination at 390 nm for 20 min.

Addition of 2-*Z* isomer to a known mixture of isomers. A solution of a mixture of isomers **2-*Z/E*** (53/47) (6 mg, 0.008 mmol) in 0.5 mL of CD_2Cl_2 was placed in an NMR tube and **2-Z** (6 mg, 0.008 mmol) was added to the mixture. The mixture of isomers *Z/E* reaction changed to (79/21) and remain stable over time, according to $^{31}P\{^{1}H\}$ NMR spectroscopy. Later, the mixture was illuminated with a Kessil lamp at 390 nm. After 20 min, the original ratio of isomers *Z/E* (56/44) was observed.

Isomerization of compound 8

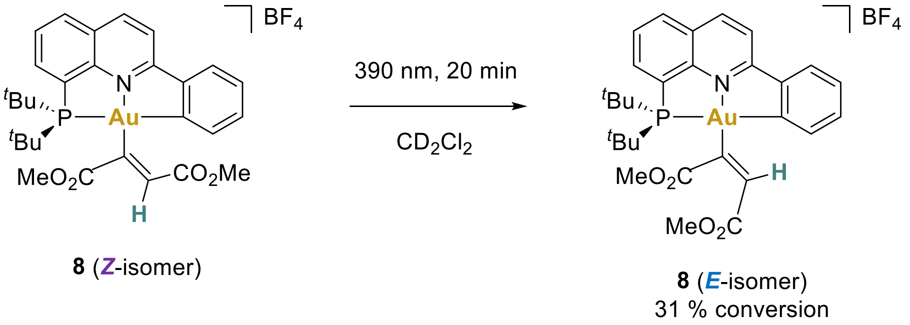

Scheme S5. Isomerization of compound **8** under blue light.

The photoisomerization of compound **8** (Scheme S5) was monitored by $^{31}P\{^1H\}$ NMR spectroscopy. A solution of **8-*Z*** (5 mg, 0.06 mmol) in 0.5 mL of CD_2Cl_2 was placed in an NMR tube and illuminated with a Kessil lamp at 390 nm. After 20 min of illumination, a mixture of isomers *Z/E* (69/31) was observed. Prolonged exposure to this light resulted in the activation of the solvent, leading to the formation of [Au]-Cl species, yet the *Z/E* ratio remained unchanged. In the absence of light, the ratio *Z/E* is maintained over time (Figure S7-8). Under the same irradiation conditions as with compound **2**, significantly less conversion to the *E*-isomer is achieved, possibly due to steric hindrance caused by the CO_2R substituents.

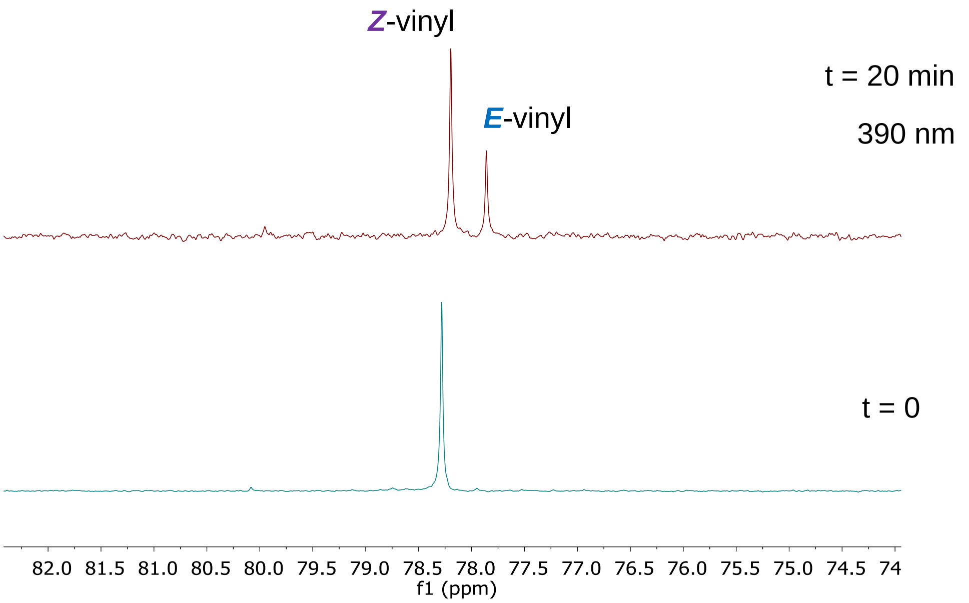

Figure S7. Stacked $^{31}P\{^1H\}$ NMR spectra (161.99 MHz, in CD_2Cl_2) showing the isomerization of compound **8** after illumination at 390 nm for 20 min.

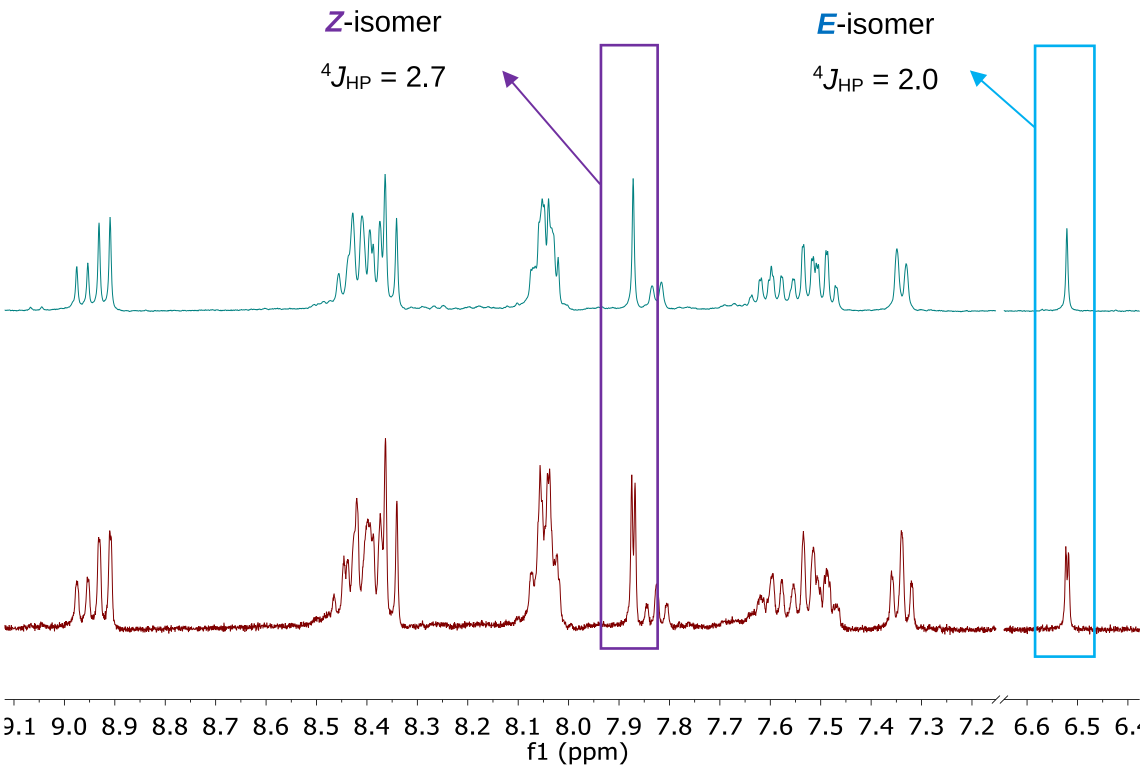

Figure S8. Comparison between $^1H\{^{31}P\}$ (top) and ^1H (bottom) NMR spectra (400.13 MHz, in CD_2Cl_2) showing the isomerization of compound **8** after illumination at 390 nm for 20 min.

Isomerization of compound 9

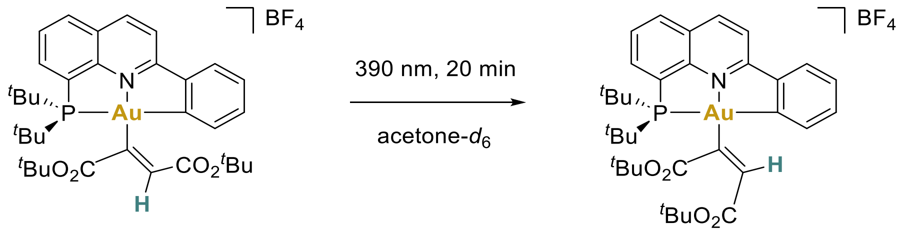

Scheme S6. Isomerization of compound **9** under blue light.

The photoisomerization of compound **9** (Scheme S6) was monitored by $^{31}P\{^1H\}$ NMR spectroscopy. A solution of **9-*Z*** (22.8 mg, 0.031 mmol) in 0.5 mL of acetone-d_6 was placed in an NMR tube and illuminated with a Kessil lamp at 390 nm. After 20 min of illumination, a mixture of isomers *Z/E* (73/27) was observed. Prolonged exposure to this light resulted in the activation of the solvent, leading to the formation of [Au]-Cl species, yet the *Z/E* ratio remained unchanged. In the absence of light, the ratio *Z/E* is maintained over time (Figure S9-10). Under the same irradiation conditions as with compound **2**, significantly less conversion to the *E*-isomer is achieved, possibly due to steric hindrance

caused by the CO_2R substituents. Additionally, the isomerization process is unaffected by the change of solvent.

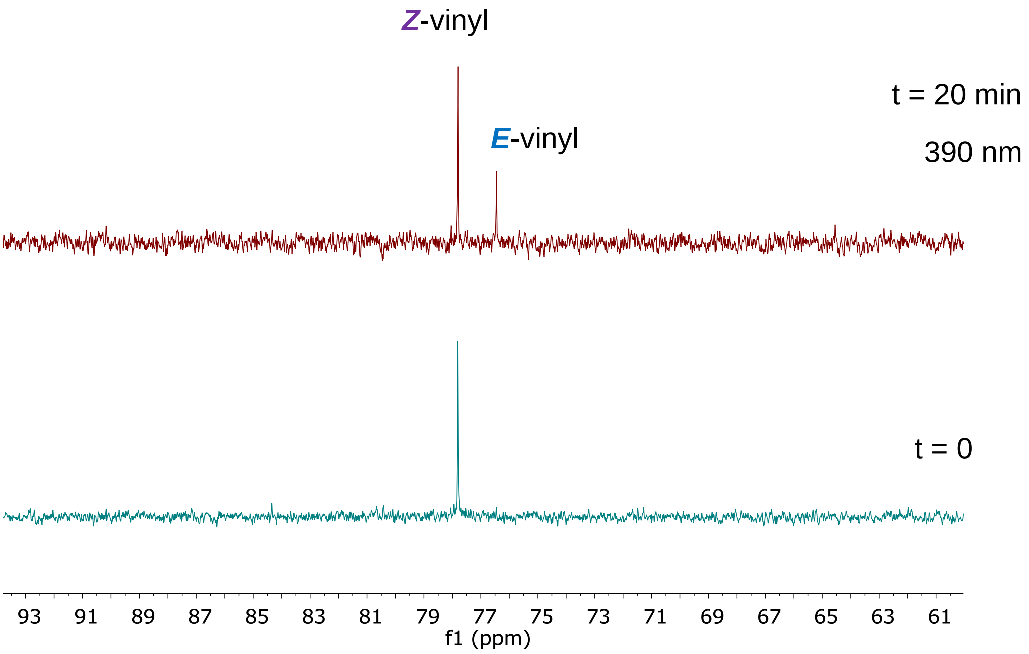

Figure S9. Stacked $^{31}P\{^1H\}$ NMR spectra (161.99 MHz, in acetone-d_6) showing the isomerization of compound **9** after illumination at 390 nm for 20 min.

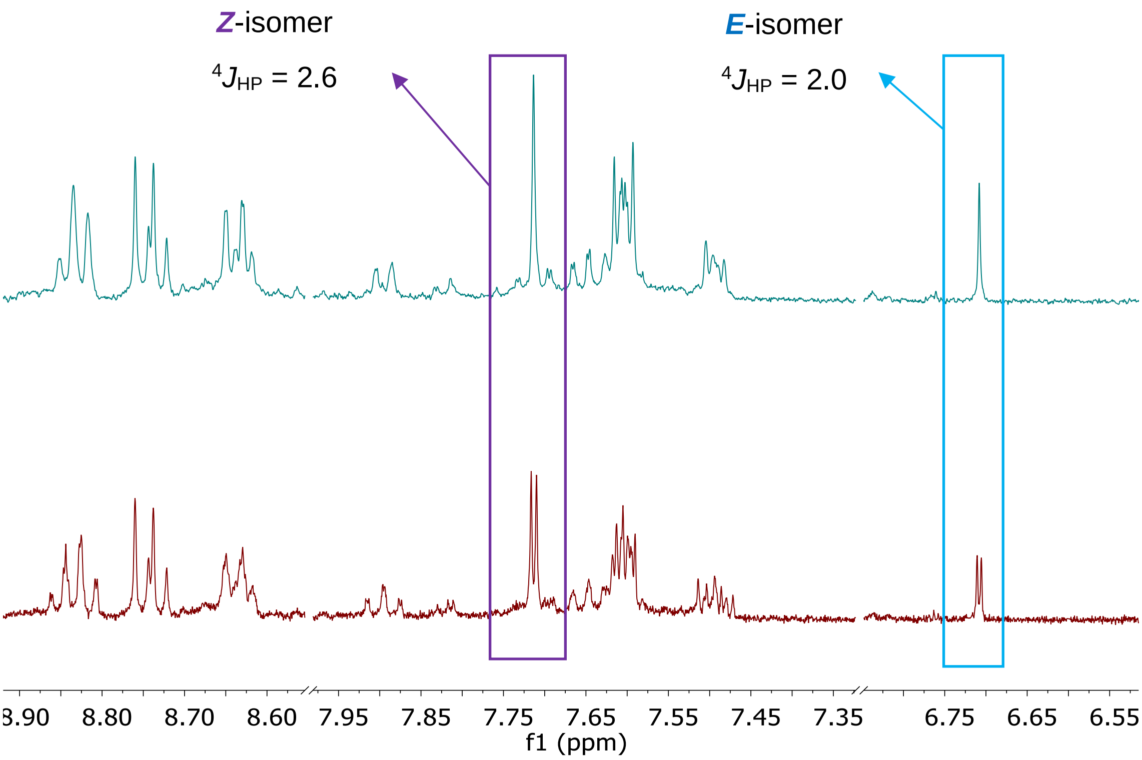

Figure S10. Comparison between $^1H\{^{31}P\}$ (top) and ^1H (bottom) NMR spectra (400.13 MHz, in acetone-d_6) showing the isomerization of compound **9** after illumination at 390 nm for 20 min.

Addition of complex 1 to known 8-*Z*/*E* isomer mixtures

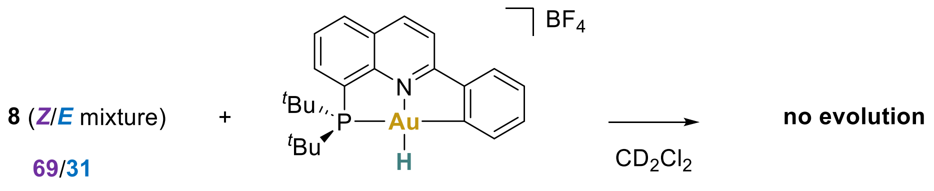

Scheme S7. Control experiment of a known isomer-mixture of **8** in presence of **1**.

A *Z*/*E* mixture 69/31 of complex **8** (5 mg, 0.006 mmol) and 0.5 equiv of complex **1** (2 mg, 0.003 mmol) were placed in a NMR tube, and 0.5 mL of CD_2Cl_2 was added to dissolve the mixture (Scheme S7). The sample was analysed by NMR spectroscopy, and the ratio of isomers was observed to remain unchanged (Figure S11).

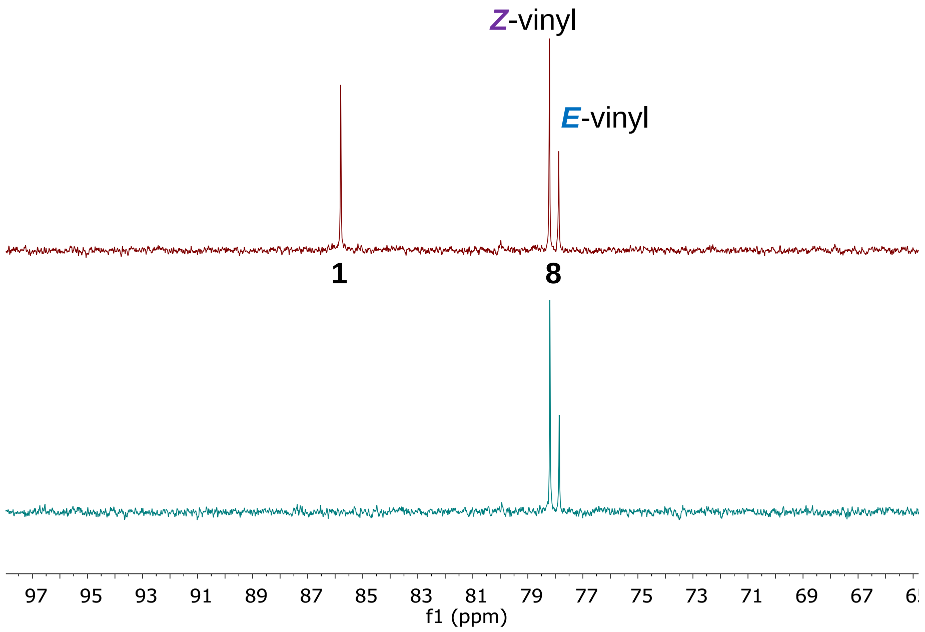

Figure S11. Stacked $^{31}P\{^1H\}$ NMR spectra (161.99 MHz, in CD_2Cl_2), showing that a mixture of isomers *Z*/*E* of complex **8** remain unchanged after addition of complex **1**.

3.4 Kinetics

General procedure. The insertion reaction of complex **1** with DMAD was tracked by $^{31}P\{^{1}H\}$ NMR spectroscopy at 298 K (Scheme S8). The samples were prepared in a glovebox under Argon atmosphere. With a precision syringe, 0.5 mL of a stock solution of DMAD in DCM-d_2 was added to an NMR tube containing the required amount of **1** and a capillary tube with a solution of PPh_3 in DMSO-d_8 as internal standard. NMR spectra were recorded periodically to show the formation of compound **8** as a function of the time (See Figure S12 for an example). The parameters of the $^{31}P\{^{1}H\}$ NMR were modified to allow the integration of the signals: pulse program (zgig30), $d_1 \geq 5T_1$ (d_1 = 14 s).

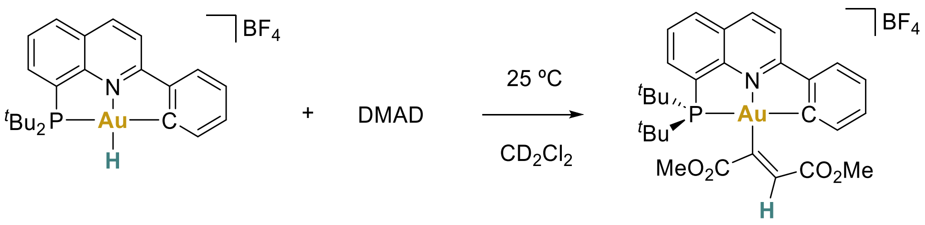

1 **8**

Scheme S8. Reaction of **1** with DMAD. Kinetic study.

Order in gold. The rate dependence on gold concentration was investigated at 298 K maintaining a constant concentration of DMAD (0.065 M). Initial rates at ca. 10-15 % conversion were measured across a range of concentrations of **1**, spanning from 0.050 M to 0.100 M (Table S1, Figure S13).

Table S1. Kinetic data for the insertion reaction of **1** with DMAD, with variable concentrations of **1**.

**Entry**	**[1]** (M)	**[DMAD]** (M)	**rate** [**8**]/dt ($M \cdot h^{-1}$)	**Half-life** (h)
**1**	0.050	0.065	0.0038	12.8±1.0
**2**	0.065	0.065	0.0062	9.9±1.0
**3**	0.080	0.065	0.0078	6.7±1.0
**4**	0.100	0.065	0.0126	5.6±1.0

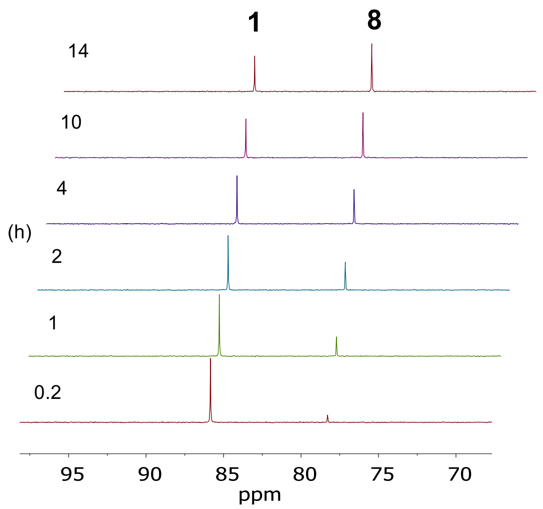

Figure S12. $^{31}P\{^{1}H\}$ NMR spectra (161.99 MHz, in CD_2Cl_2) for the transformation of **1** into **8** at 298 K under the conditions of entry 2 in Table S1. No other intermediates are detected by NMR.

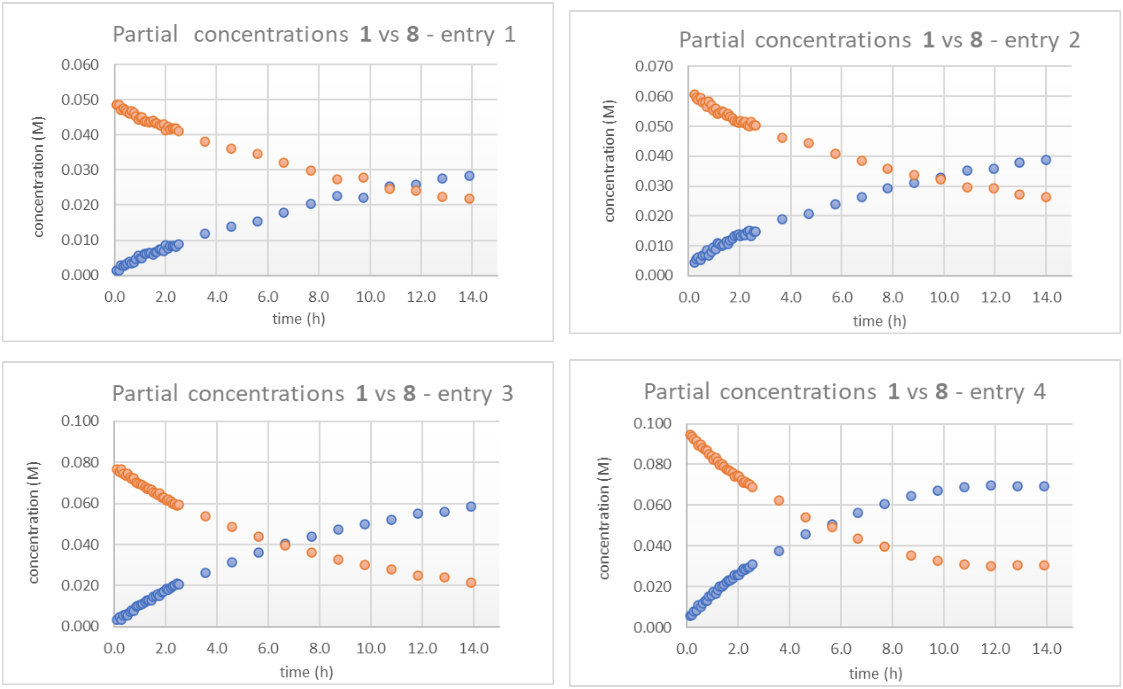

Figure S13. Partial concentrations of **1** (orange) vs **8** (blue) for the insertion reaction under the conditions of Table S1.

The plot of ln(rate) against ln[**1**] yields a straight line with a slope of 1.7, suggesting a second-order dependence on gold concentration (Figure S14).

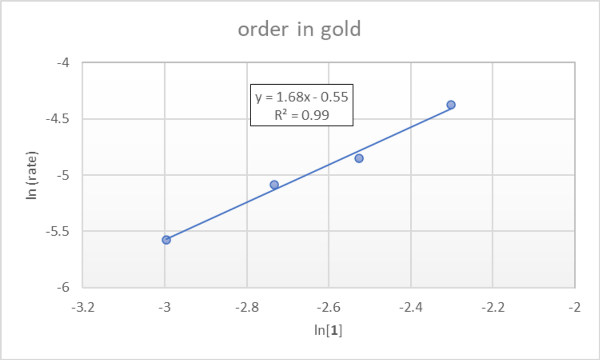

Figure S14. Plot of ln(d[**8**]/dt) as a function of ln[**1**] in DCM-d_2 at 298 K, for a constant [DMAD] of 0.065 M.

The plot d[**8**]/dt as a function of [**1**]^2 fits with a second-order equation (R^2 = 0.99), while a first-order plot provides a non-linear fitting (Figure S15).

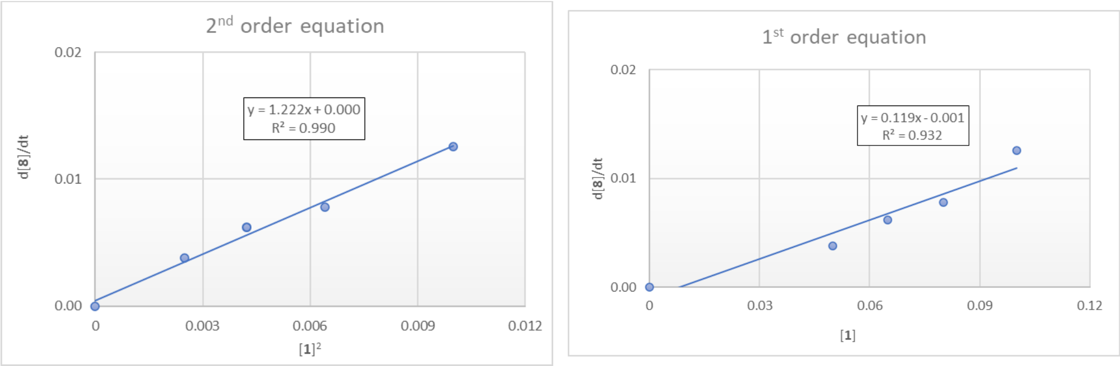

Figure S15. Plot of d[**8**]/dt as a function of [**1**]^2 (2nd order equation) and as a function of [**1**] (1st order equation) in DCM-d_2 at 298 K, for a constant [DMAD] of 0.065 M.

In addition, it is worth noting that the half-life value increases when [**1**] decreases (Table S1), which is as well consistent with the 2nd order dependence on gold.

Order in alkyne. Similarly, the rate dependence on alkyne concentration was investigated at 298 K maintaining a constant concentration of **1** (0.065 M). Initial rates at ca. 10-15 % conversion were measured across a range of concentrations of DMAD, spanning from 0.065 M to 1.300 M (Table S2, Figure S16).

Table S2. Kinetic data for the insertion reaction of **1** with DMAD, with variable concentrations of DMAD.

**Entry**	**[1]** (M)	**[DMAD]** (M)	**rate** [**8**]/dt (M·h^{-1})	**Half-life** (h)
**1**	0.065	0.065	0.0062	9.9±1.0
**2**	0.065	0.100	0.0066	9.8±1.0
**3**	0.065	0.130	0.0074	11.8±1.0
**4**	0.065	0.326	0.0043	18.0±1.0
**5**	0.065	1.300	0.0023	n.d.

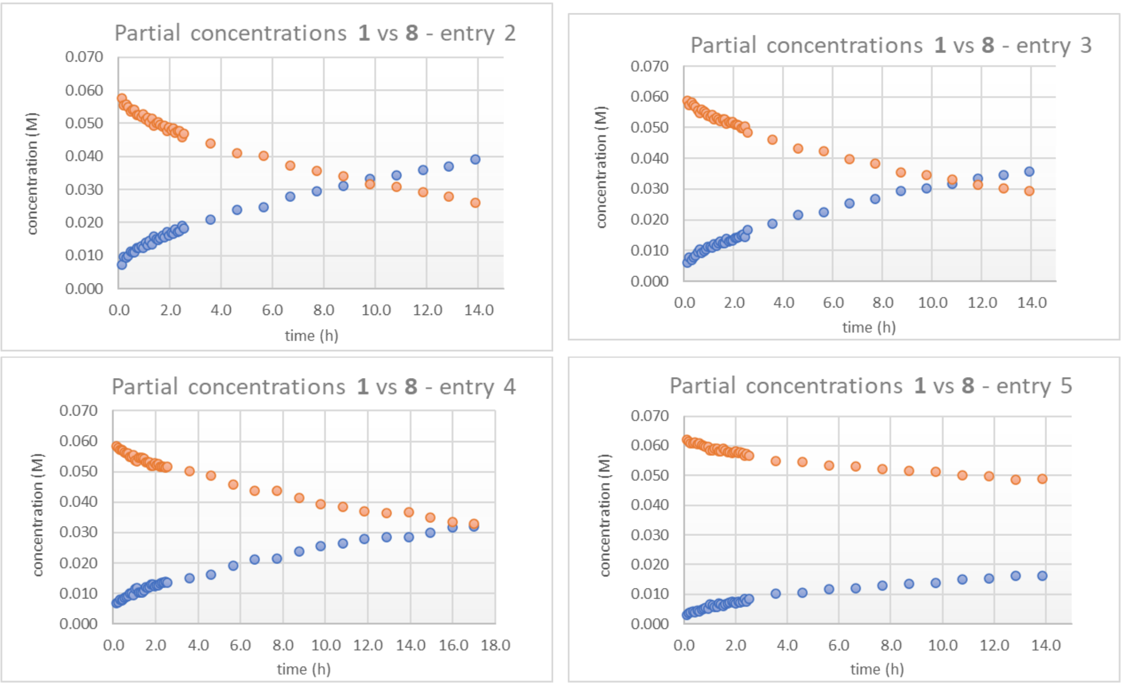

Figure S16. Partial concentrations of **1** (orange) vs **8** (blue) for the insertion reaction under the conditions of Table S2.

The initial rates for entries 1-3 in Table **S2** remain similar within the range of concentrations studied, indicating that the rate is independent on the [DMAD]$_0$. The plot ln(rate) against ln[**8**] yields a straight line with a slope parallel to the x-axis, suggesting a zero-order dependence on alkyne concentration (Figure S17).

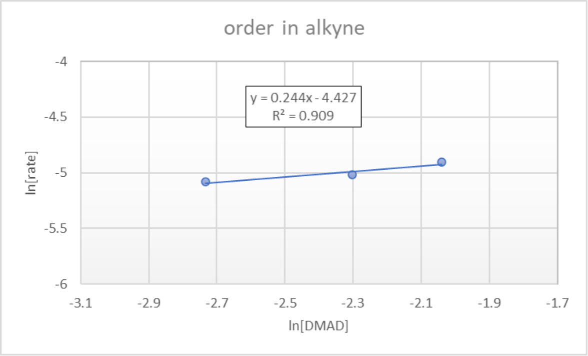

Figure S17. Plot of lnd[**8**]/dt as a function of [DMAD] in DCM-d_2 at 298 K, for a constant [**1**] of 0.065 M.

Interestingly, it was observed that using 5-fold excess of DMAD (Table S2, entry 4) significantly reduced the reaction rate, resulting in a half-life of 18 hours. Furthermore, when 20 equiv of DMAD were used (Table S2, entry 5) even lower initial rate is observed with non-determined half-life along 14 h of experiment. This outcome, unfortunately, prevents the study of kinetics under pseudo-first-order conditions. An inhibition of the reaction does not appear to occur according to the reaction profiles of entries 4 and 5 (Figure S16). A possible explanation for this behaviour is that at high concentrations, the alkyne affects to the rate-limiting step by preventing two gold units from coming into close proximity. In contrast, when the concentration of alkyne and gold are similar, the two gold units can approach each other more easily.

Kinetic isotope effect (KIE). The insertion reaction of complex **1-D** (0.065 M) with DMAD (0.065 M) was tracked by $^{31}P\{^1H\}$ NMR spectroscopy at 298 K. The initial rate of this experiment was 0.0035 $M \cdot h^{-1}$. Comparison of this result with the initial rates obtained for the non-deuterated complex **1** yields a KIE value of 1.8.

3.5 Calculation of thermodynamic activation parameters

The activation parameters were calculated using the Eying-Polanyi and Gibbs-Helmholtz equations:

$$\ln\frac{k}{T} = \frac{-\Delta H^{\ddagger}}{RT} + \ln\frac{k_B}{h} + \frac{\Delta S^{\ddagger}}{R}$$

$$\Delta G^{\ddagger}(T) = \Delta H^{\ddagger} - T\cdot\Delta S^{\ddagger}$$

k = reaction rate constant

$\Delta H^{\ddagger}$ = Enthalpy of activation

$\Delta S^{\ddagger}$ = Entropy of activation

$\Delta G^{\ddagger}$ = Gibbs energy of activation

T = temperature

R = gas constant = 1.987 $cal\cdot K^{-1}\cdot mol^{-1}$

k_B = Boltzmann constant = $3.29762\cdot 10^{-24}$ $cal\cdot K^{-1}$

h = Planck constant = $4.39529\cdot 10^{-38}$ $cal\cdot h$

Table S3. Kinetic data for the insertion reaction of **1** with DMAD at different temperatures.

**Entry**	**[1]** (M)	**[DMAD]** (M)	**T** (K)	**rate** [**8**]/dt ($M\cdot h^{-1}$)	**k** ($M^{-1}\cdot h^{-1}$)
**1**	0.065	0.065	298	0.0062	1.4675
**2**	0.065	0.065	308	0.0156	3.6923
**3**	0.065	0.065	318	0.0314	7.4320

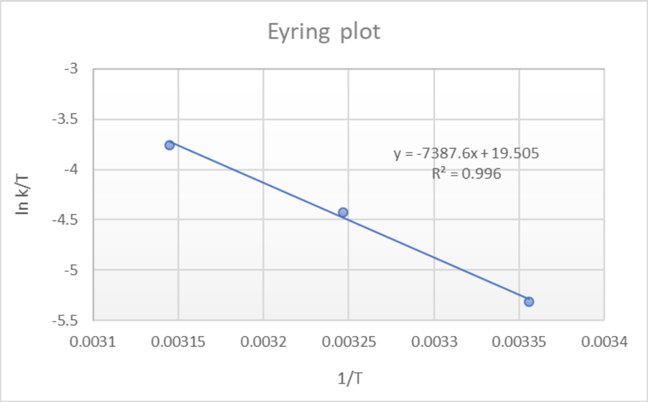

Figure S18. Eyring Plot for the insertion reaction of **1** (0.065 M) with DMAD (0.065 M).

$\Delta H^{\ddagger}$ = 15 ± 1 $kcal\cdot mol^{-1}$

$\Delta S^{\ddagger}$ = -25 ± 3 $cal\cdot mol^{-1}\cdot K^{-1}$

$\Delta G^{\ddagger}_{298K}$ = 22 ± 1 $kcal\cdot mol^{-1}$

3.6 Exchange between Au-H and water

As a control experiment, we monitored the evolution of compound **1** in CD_2Cl_2 saturated with D_2O by NMR spectroscopy (Scheme S9). Compound **1** is partially transformed into Au-deuteride species (Figure S19a, b), reaching an H/D ratio of 58/42 after 4.5 h, which remains stable over time (Figure S19c). Only trace amounts of Au-Cl species were detected after extended periods and upon exposure to light.

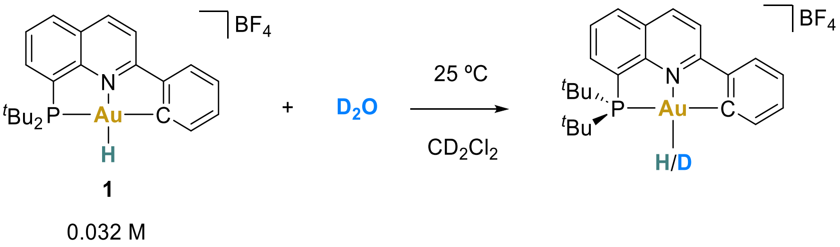

Scheme S9. Reaction of **1** with D_2O in CD_2Cl_2.

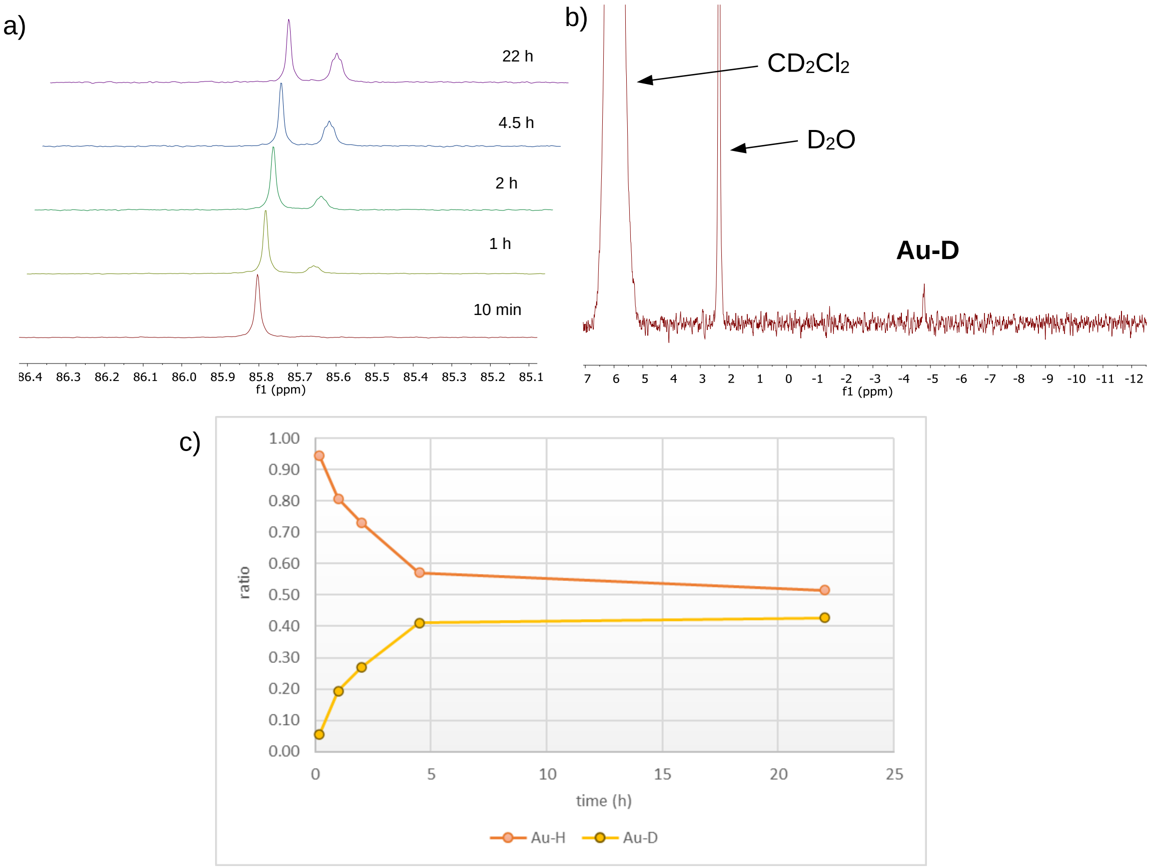

Figure S19. a) Stacked $^{31}P\{^1H\}$ NMR spectra (161.99 MHz, in CD_2Cl_2 saturated with D_2O) showing the transformation of **1** into **1-D** at 298 K. b) ^2H NMR spectrum (61.42 MHz, in CD_2Cl_2 saturated with D_2O) showing the signal of the deuterated hydride in the high field region. c) Partial concentrations of **1** (orange) vs **1-D** (yellow) under the conditions of Figure S19a.

3.7 Effect of water on the kinetics

During our investigations, we observed that the presence of moisture affects the kinetics of the reaction. To quantify this effect, we performed experiments by adding known amounts of water to the equimolar reaction of **1** (0.065 M) + DMAD (0.065 M) (Scheme S10) and tracking the concentrations of **1** and **8** over time (Table S4, Figure S20). The reactions were prepared as described at the beginning of section 3.4. The stock solutions of DMAD in CD_2Cl_2 were prepared with the required amount of water in each case.

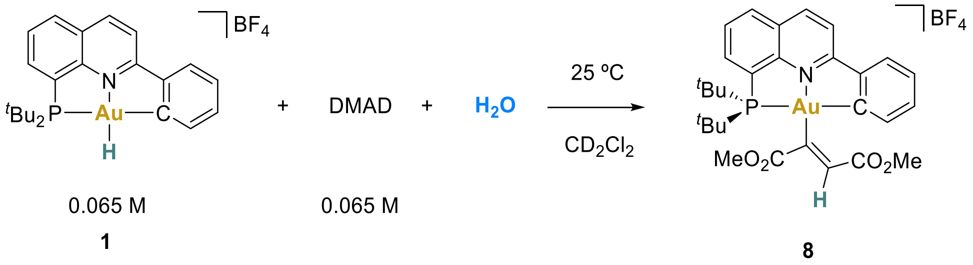

Scheme S10. Reaction of **1** with DMAD in the presence of H_2O. Kinetic study.

Table S4. Kinetic data for the insertion reaction of **1** with DMAD in presence of water.

**Entry**	**[H$_2$O]** (equiv)	**rate** [**8**]/dt (M·h^{-1})	**Half-life** (h)	**Ratio** ***Z/E***
**1**	-	0.0062	9.9 ± 1.0	-
**2**	1	0.0100	4.6 ± 1.0	-
**3**	5	0.0285	1.6 ± 0.1	97/3
**4**	saturated	0.0317	1.6 ± 0.1	96/4

The addition of small amounts of water in dichloromethane significantly increases the reaction rate and reduces the half-life values (Table S4). Similarly, we observed a significant decrease on the reaction rate when using freshly distilled alkyne and freshly prepared dry DCM-d_2 under strict exclusion of moisture, suggesting that trace amounts of water present under standard conditions are required for the reaction to proceed.

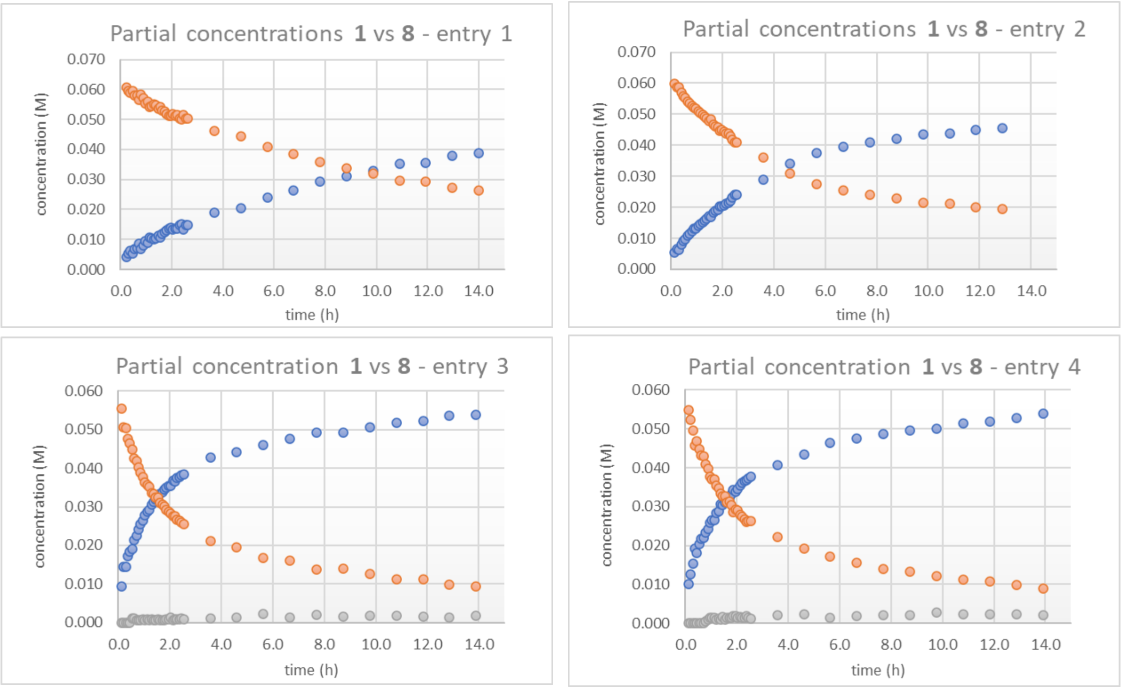

Figure S20. Partial concentrations of **1** (orange) vs **8** (blue) for the insertion reaction under the conditions of Table S4. Entries 3 and 4 include the partial concentrations of **8-*E*** (grey).

Interestingly, a careful evaluation of the NMR spectra at the end of the kinetic experiment in entries 3 and 4 revealed the formation of trace amounts of the gold-vinyl complex **8-*E*** (<5%). The *Z/E* ratio remains unchanged over time, suggesting that water does not promote isomerization between *Z* and *E* isomers. On the other hand, this experiment indicates that when the concentration of water is sufficient, a second mechanism should facilitate the formation of the *E* isomer.

To determine the origin of isomer *E*, we repeated the experiment from entry 4 in Table S4 using CD_2Cl_2 saturated with D_2O and tracked the concentration profiles of the species involved. We carried out the insertion reaction under equimolar conditions using CD_2Cl_2 saturated with D_2O (Scheme S11). As expected from previous control experiments (Figures S19), compound **1** is partially transformed to **1-D** during the insertion reaction with DMAD (Figure S21 a, b). Complex **8** was obtained as a mixture of *Z/E* isomers (96/4), consistent with the presence of sufficient H_2O. The formation of isomer **8-*E*** under these conditions indicates that the source of hydrogen in the vinyl gold complex is not H_2O but rather the gold-hydride **1**.

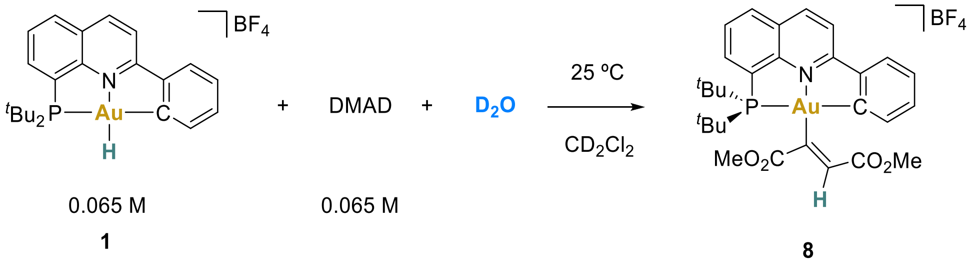

Scheme S11. Reaction of **1** with DMAD in the presence of D_2O.

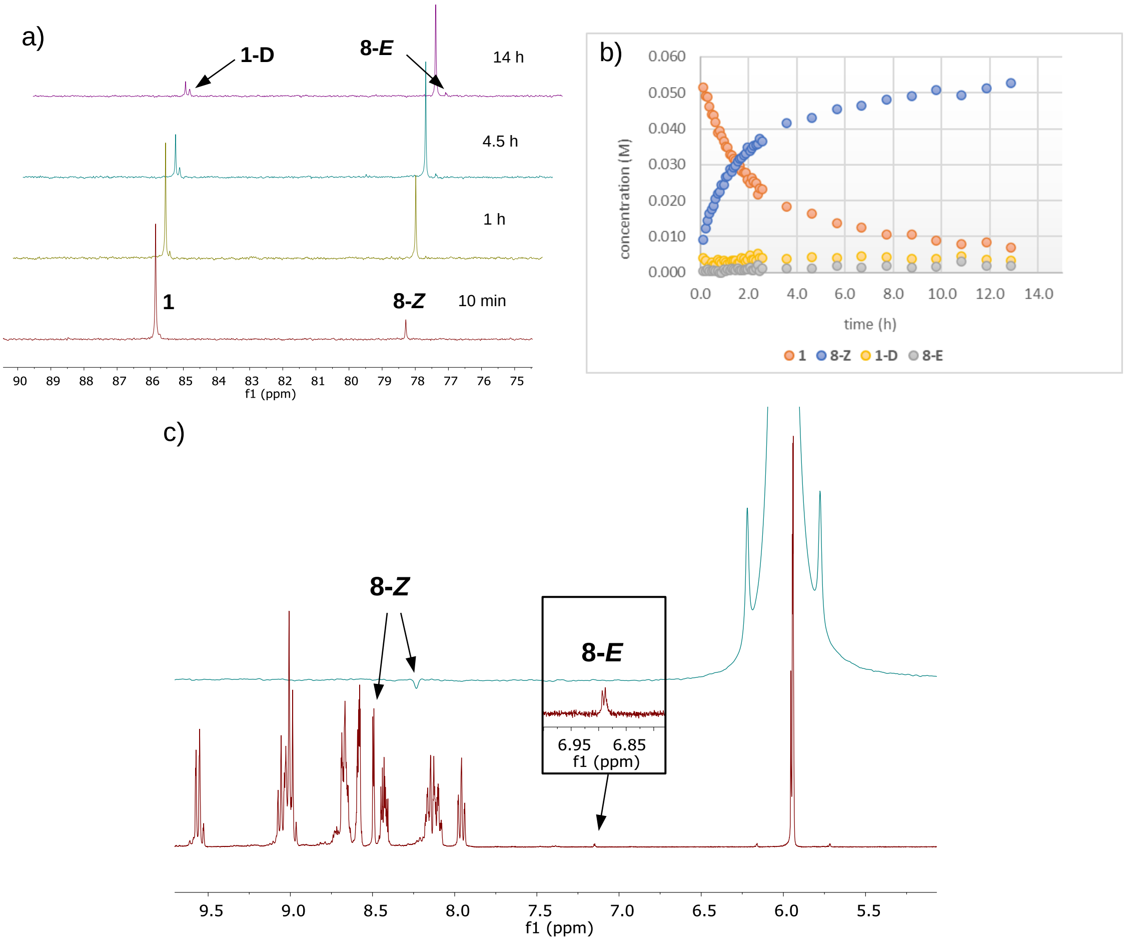

Figure S21. a) Stacked $^{31}P\{^1H\}$ NMR spectra (161.99 MHz, in CD_2Cl_2 saturated with D_2O) showing the transformation of **1** into **1-D**, **8-*Z***, and **8-*E*** at 298 K. b) Partial concentrations of **1** (orange), **8-*Z*** (blue), **1-D** (yellow), and **8-*E*** (grey). c) Comparison of ^1H (bottom) vs ^2H (top) NMR spectra in CD_2Cl_2 saturated with D_2O after 14 h, showing the vinylic signal of **8-*Z*** and **8-*E***.

Additionally, due to the formation of significant amounts of **1-D** (see Figure S19), the formation of deuterated **8** is expected and confirmed by a ^2H NMR experiment after 14 h, which showed a signal corresponding to deuterated **8-*Z*** (Figure S21, c).

Finally, the reaction rate of **1-D** (0.065 M) with DMAD (0.065 M) in CD_2Cl_2 saturated with D_2O was 0.0205 $M{\cdot}h^{-1}$ is significantly lower than the reaction rate of **1** (0.065 M) with DMAD (0.065 M) in CD_2Cl_2 saturated with H_2O (0.0317 $M{\cdot}h^{-1}$, entry 4).

4. NMR Spectra

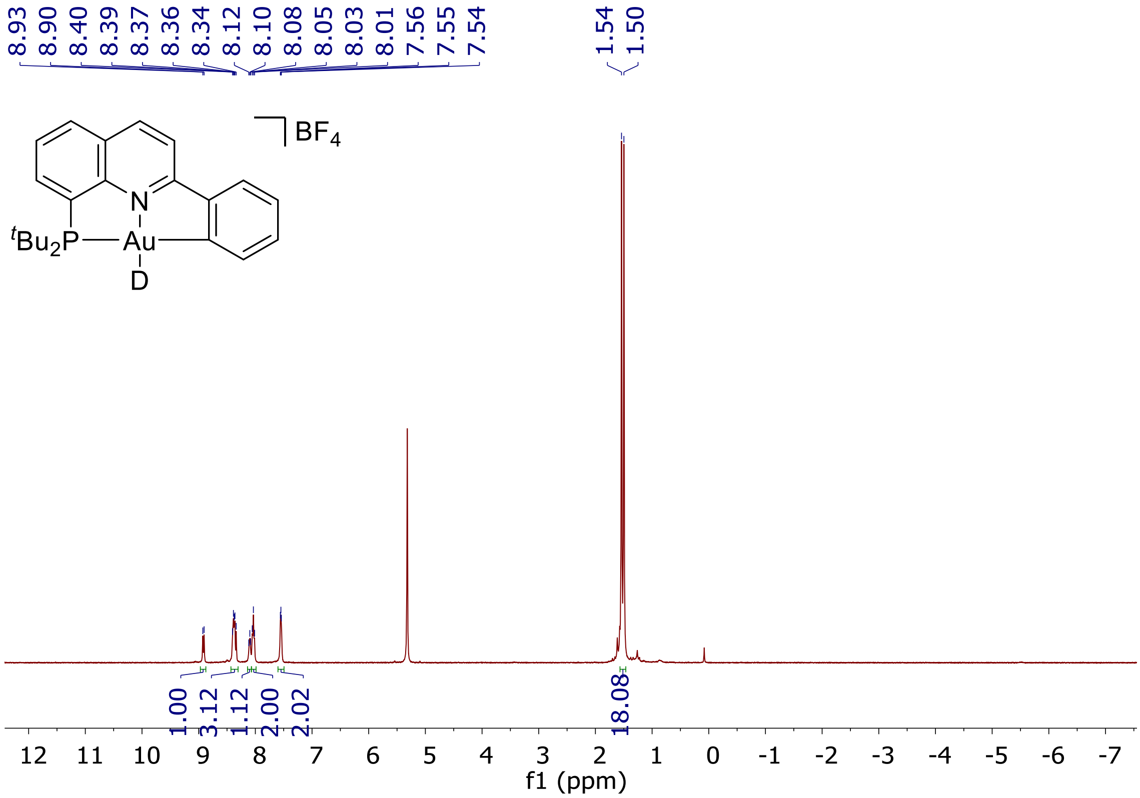

Figure S22. ^{1}H NMR (400.13 MHz, CD_2Cl_2, 298 K) spectrum of compound **1-D**.

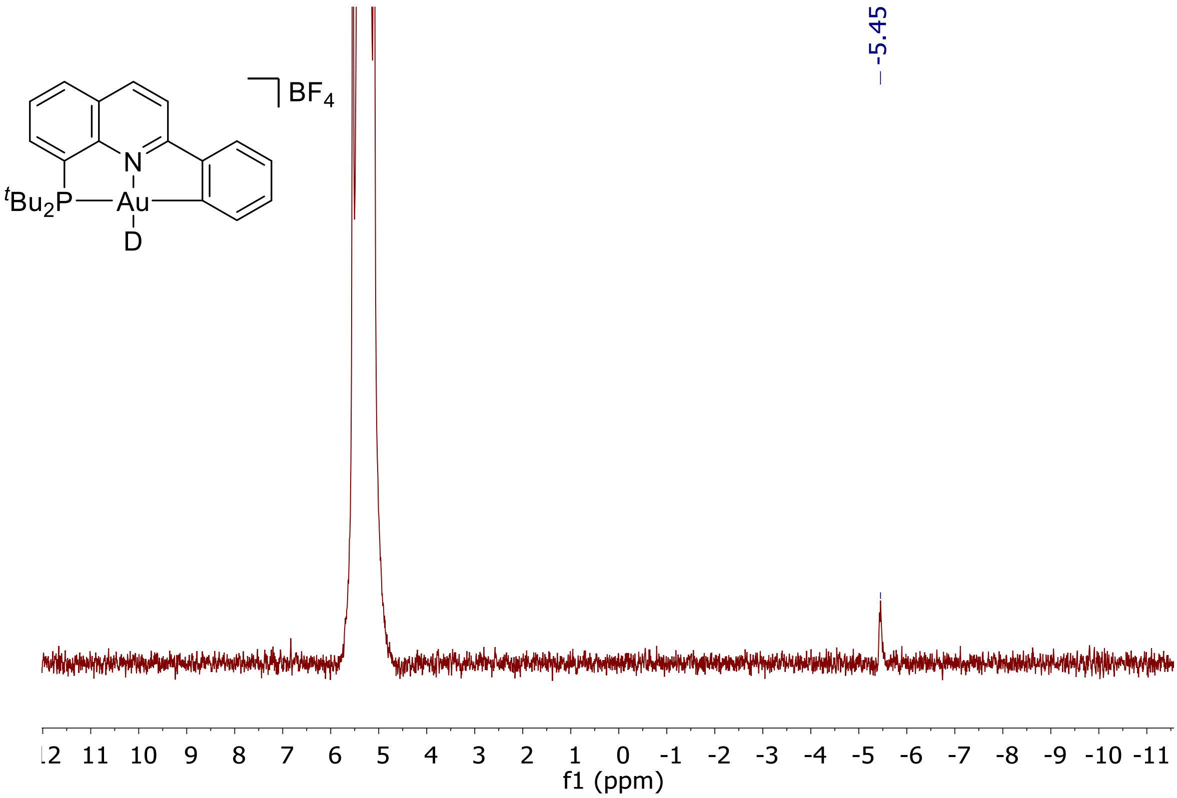

Figure S23. ^{2}H NMR (61.42 MHz, CD_2Cl_2, 298 K) spectrum of compound **1-D**.

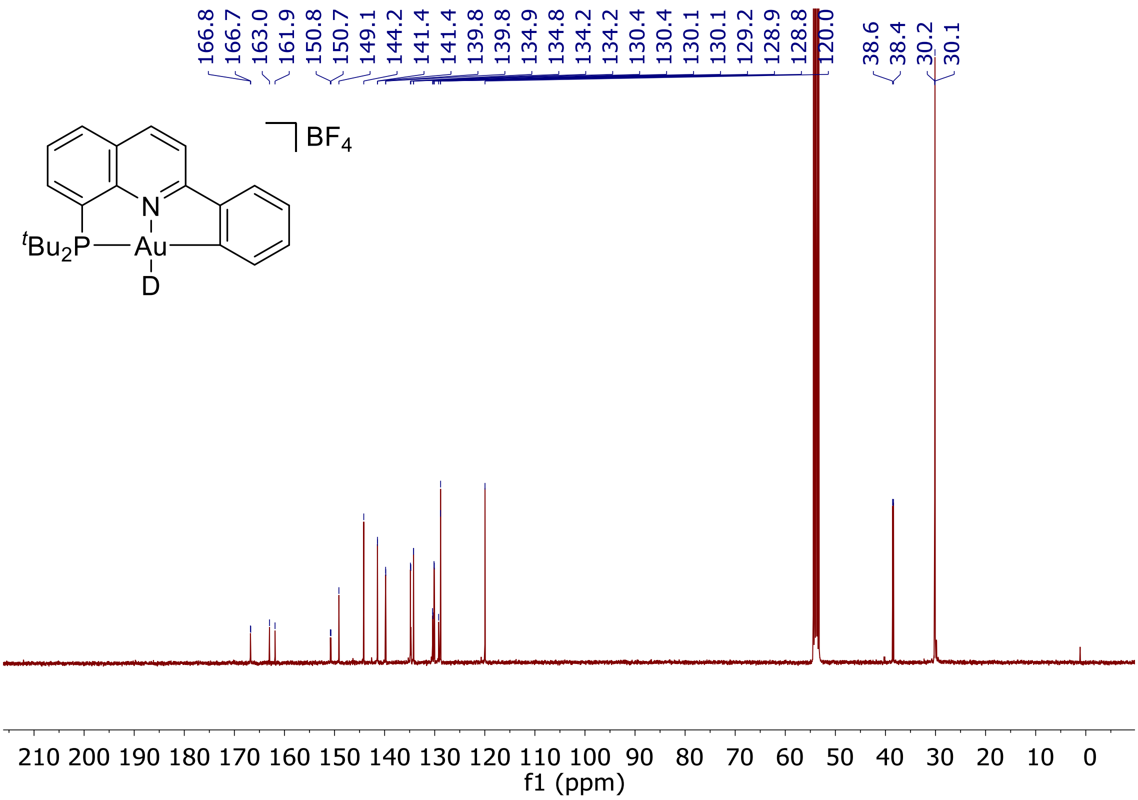

Figure S24. $^{13}C\{^1H\}$ NMR (100.62 MHz, CD_2Cl_2, 298 K) spectrum of compound **1-D**.

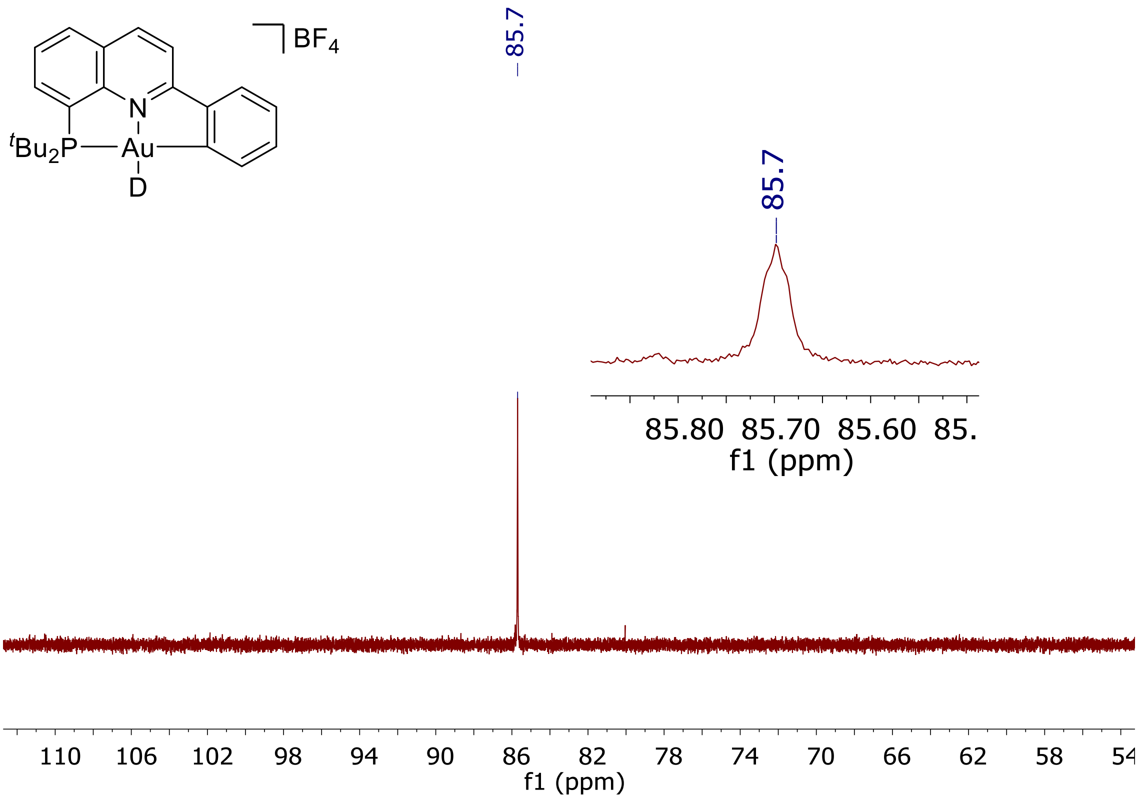

Figure S25. $^{31}P\{^1H\}$ NMR (161.99 MHz, CD_2Cl_2, 298 K) spectrum of compound **1-D**.

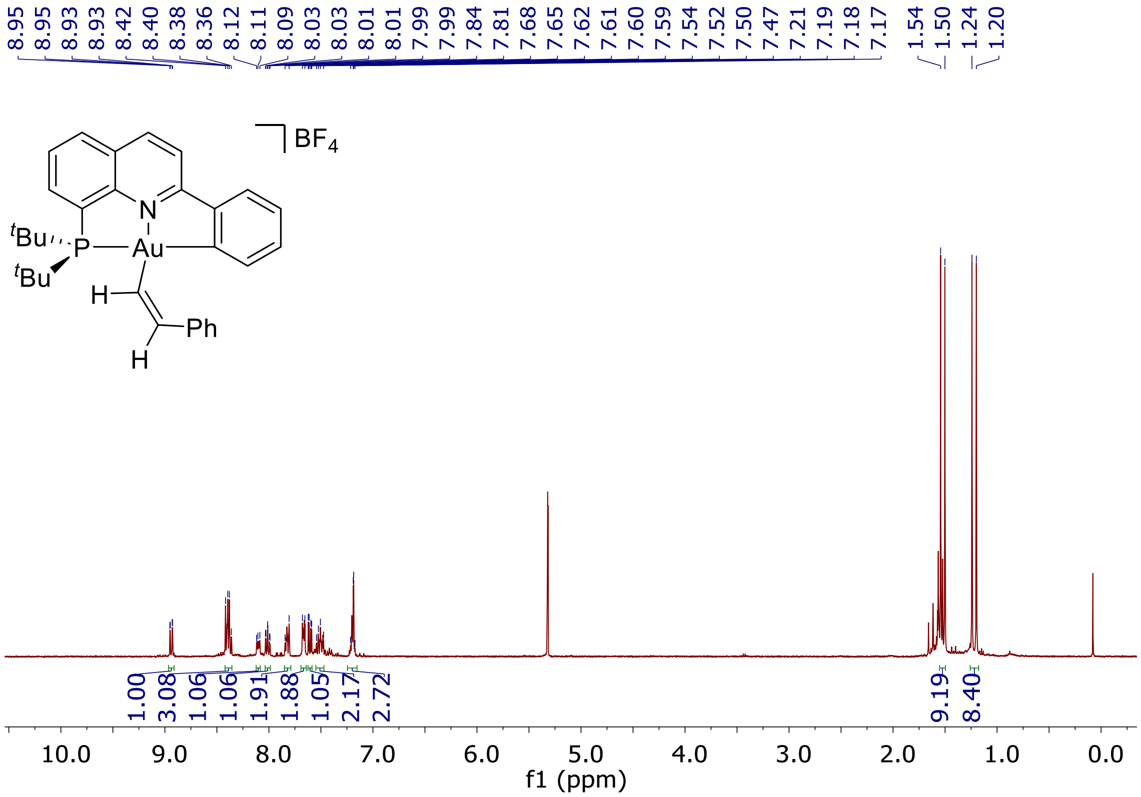

Figure S26. ^{1}H NMR (400.13 MHz, CD_2Cl_2, 298 K) spectrum of compound **2**.

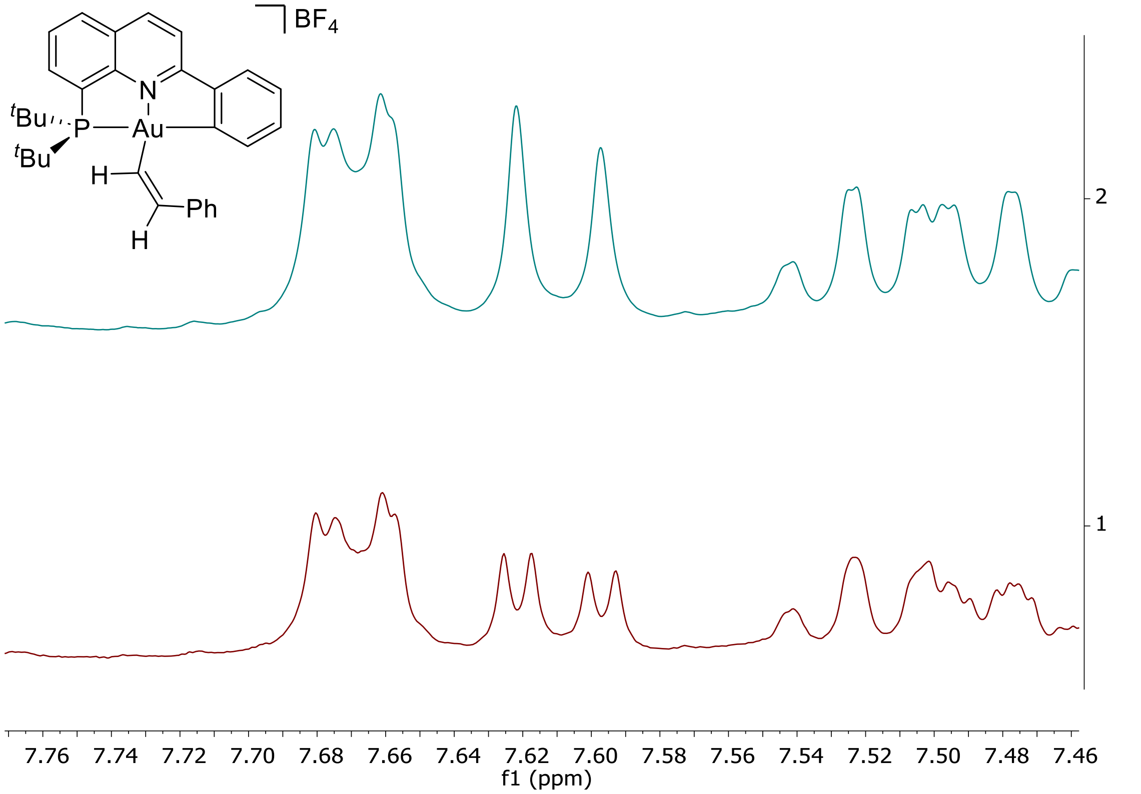

Figure S27. Comparison between ^{1}H (bottom) and ^{1}H{^{31}P} (top) NMR spectra of the vinyl signal in compound **2**.

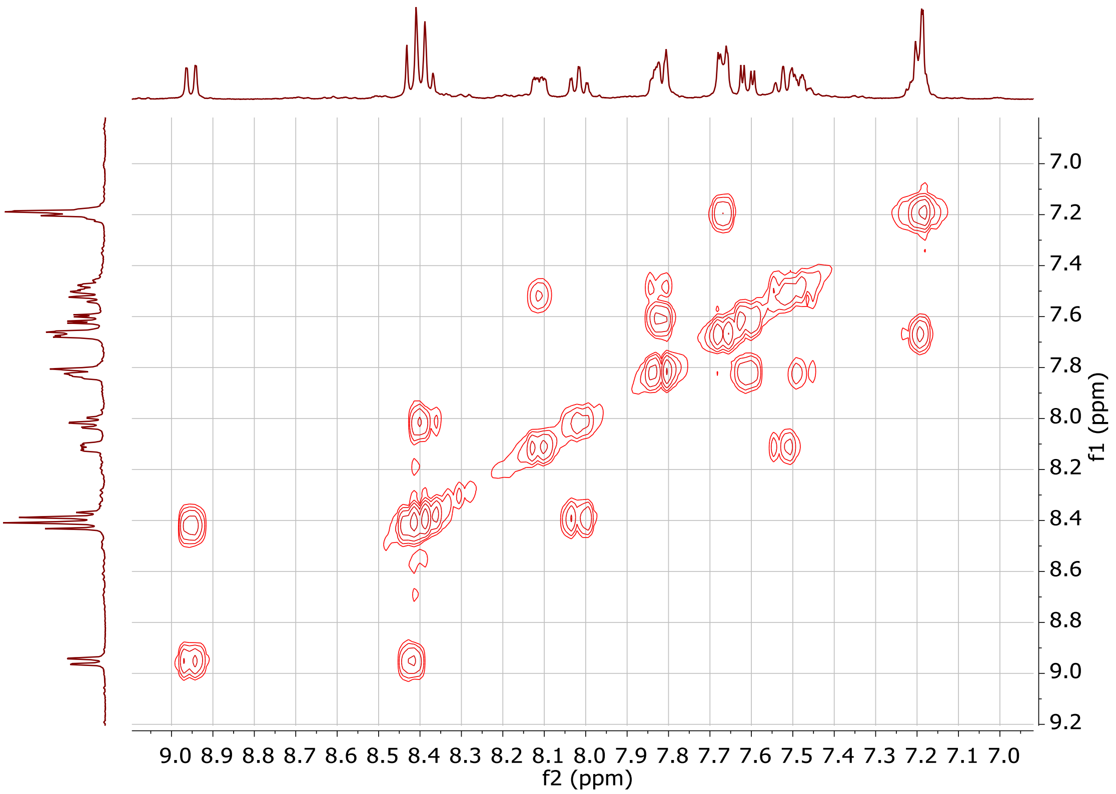

Figure S28. ^{1}H-^{1}H COSY NMR spectra of the compound **2**. Expansion of the aromatic region.

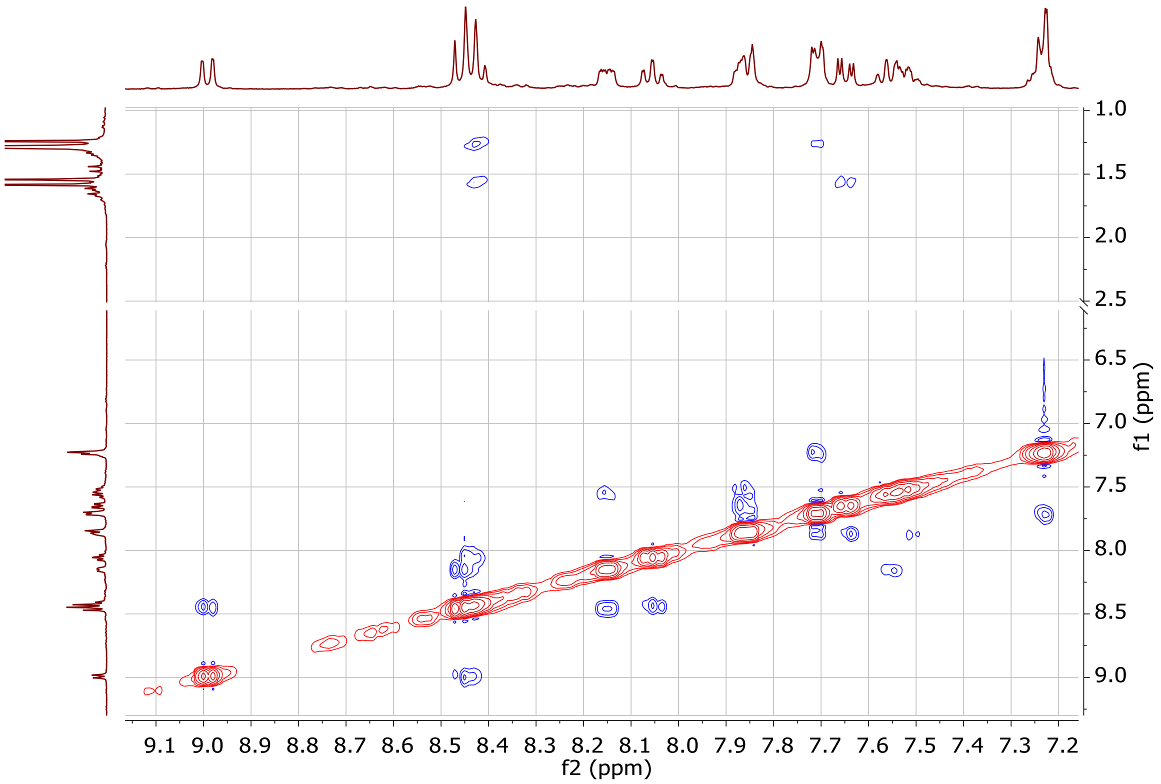

Figure S29. ^{1}H-^{1}H NOESY NMR spectra of the compound **2**. Expansion of the aromatic region.

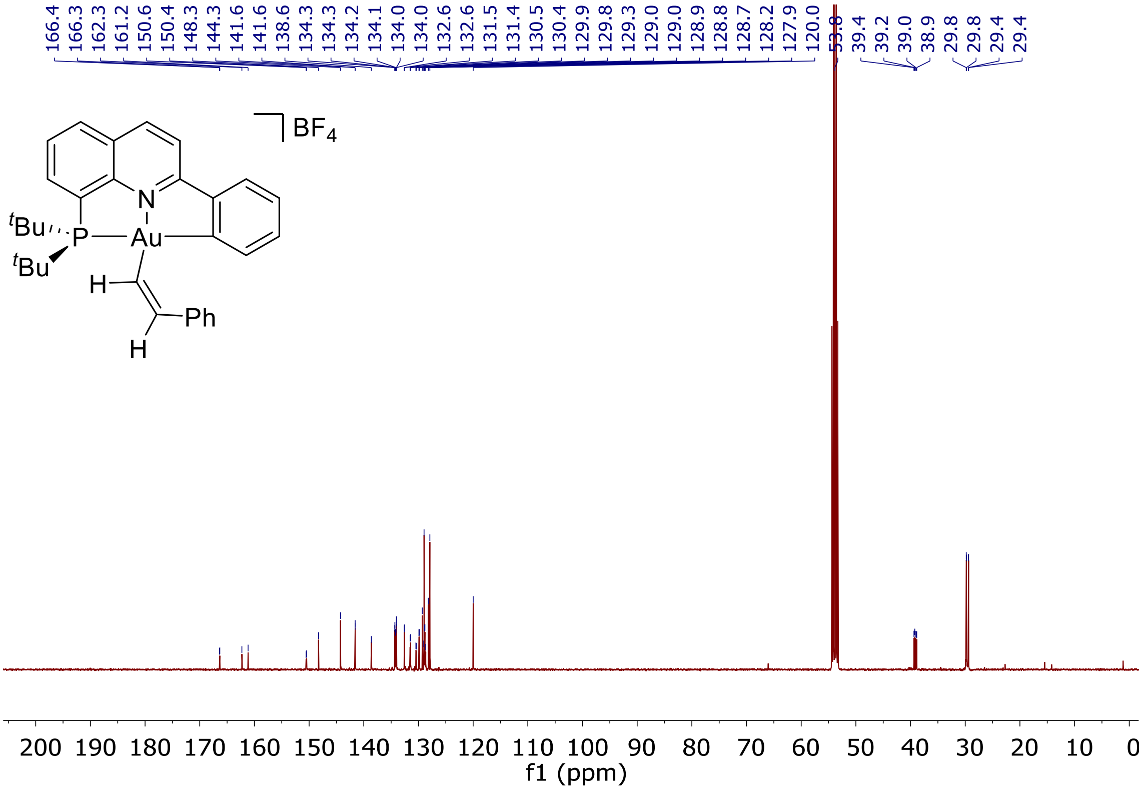

Figure S30. $^{13}C\{^{1}H\}$ NMR (100.62 MHz, CD_2Cl_2, 298 K) spectrum of compound **2**.

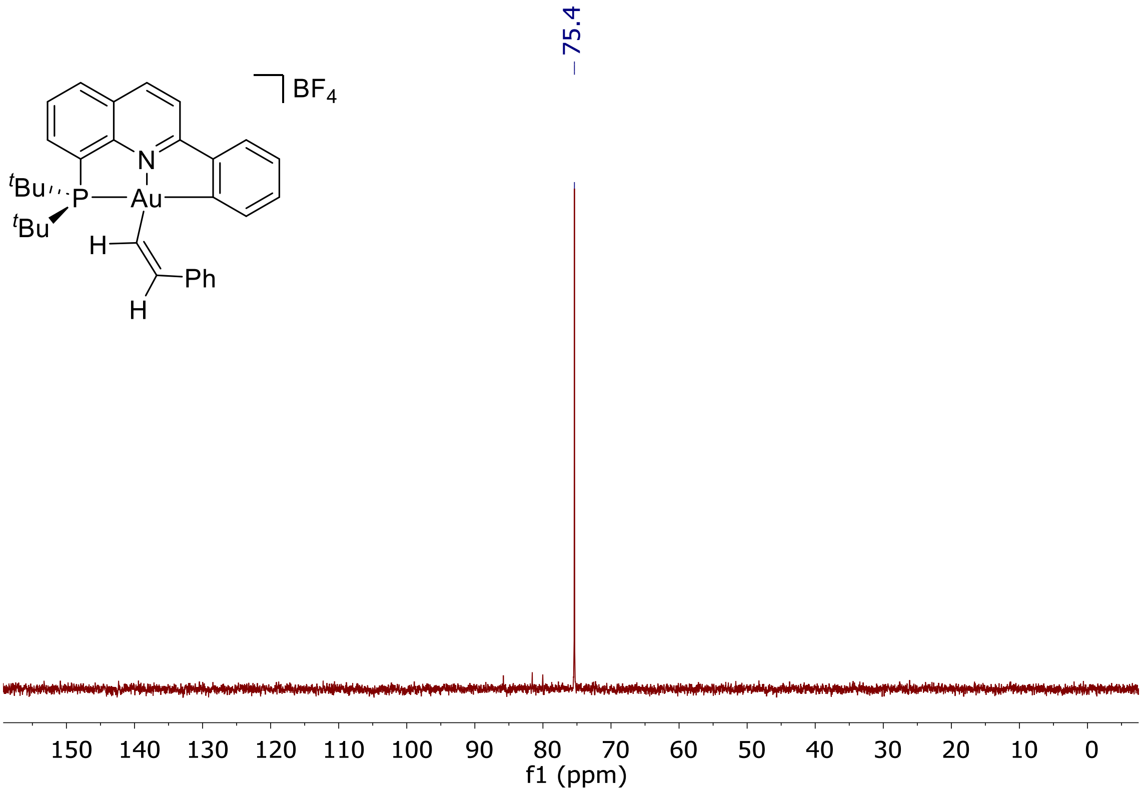

Figure S31. $^{31}P\{^{1}H\}$ NMR (161.99 MHz, CD_2Cl_2, 298 K) spectrum of compound **2**.

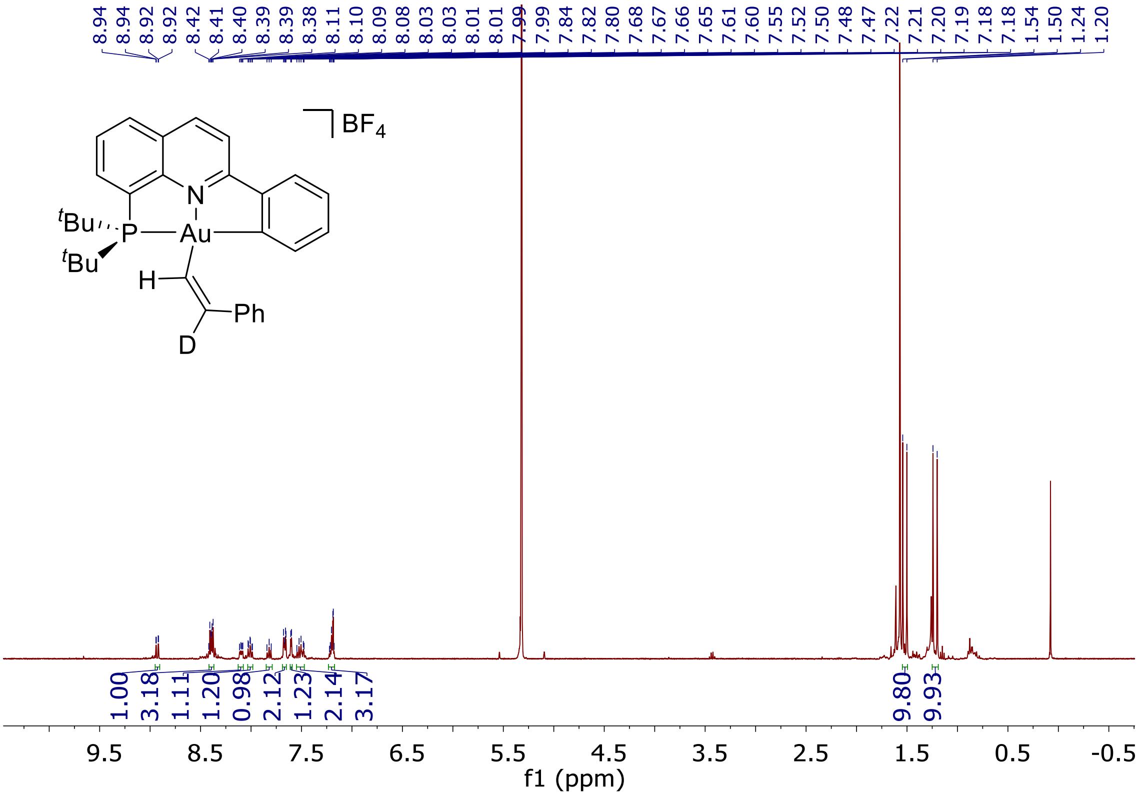

Figure S32. ^{1}H NMR (400.13 MHz, CD_2Cl_2, 298 K) spectrum of compound **2-HD**. The spectrum contains residual water (1.60).

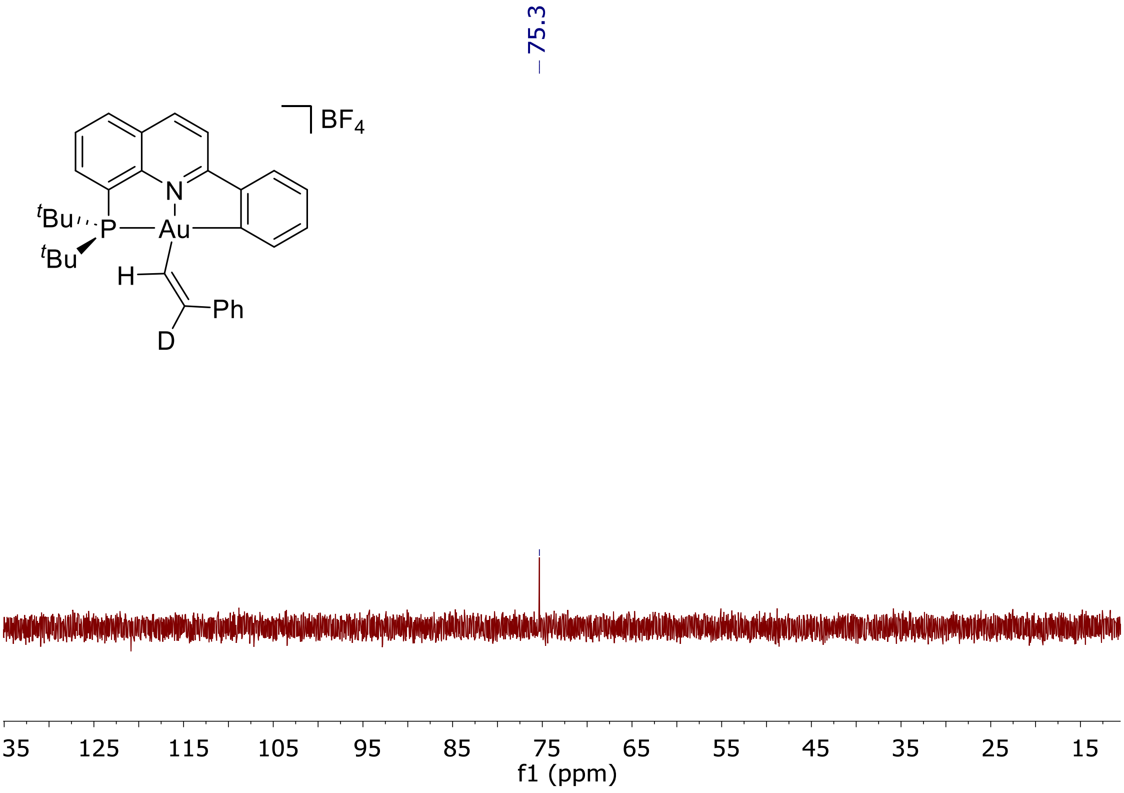

Figure S33. $^{31}P\{^{1}H\}$ NMR (161.99 MHz, CD_2Cl_2, 298 K) spectrum of compound **2-HD**.

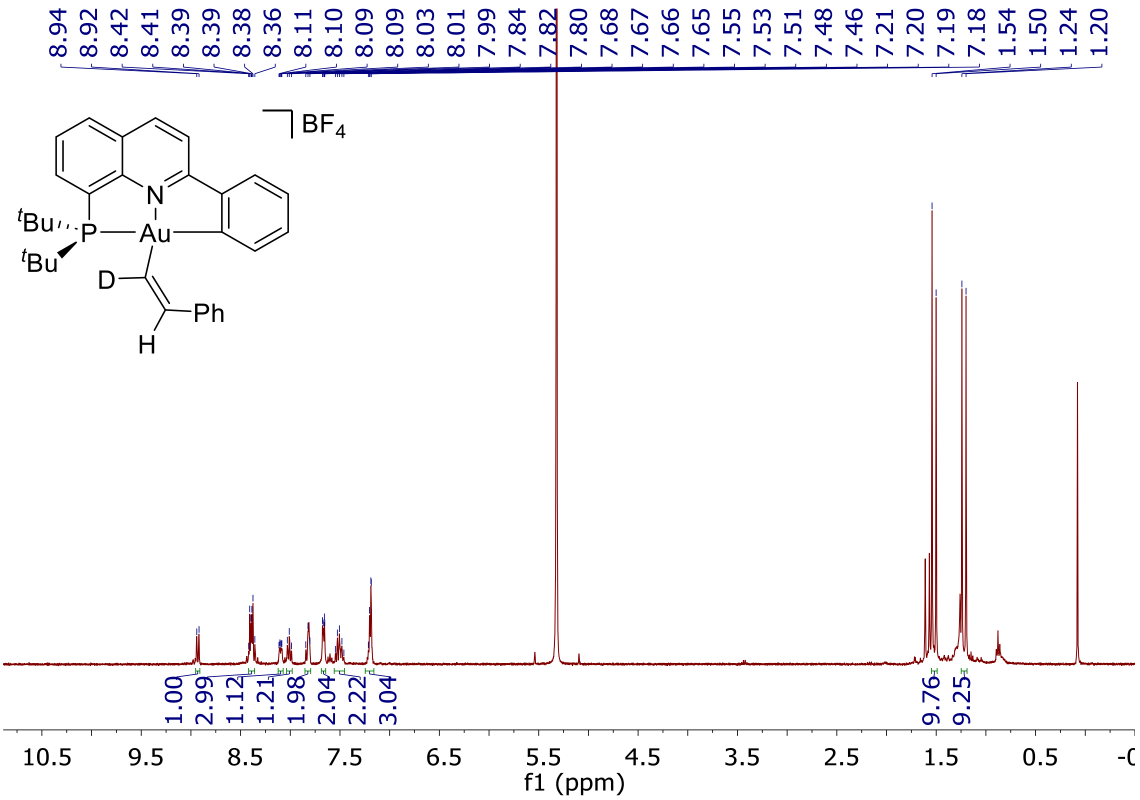

Figure S34. ^{1}H NMR (400.13 MHz, CD_2Cl_2, 298 K) spectrum of compound **2-DH**. The spectrum contains residual water (1.60).

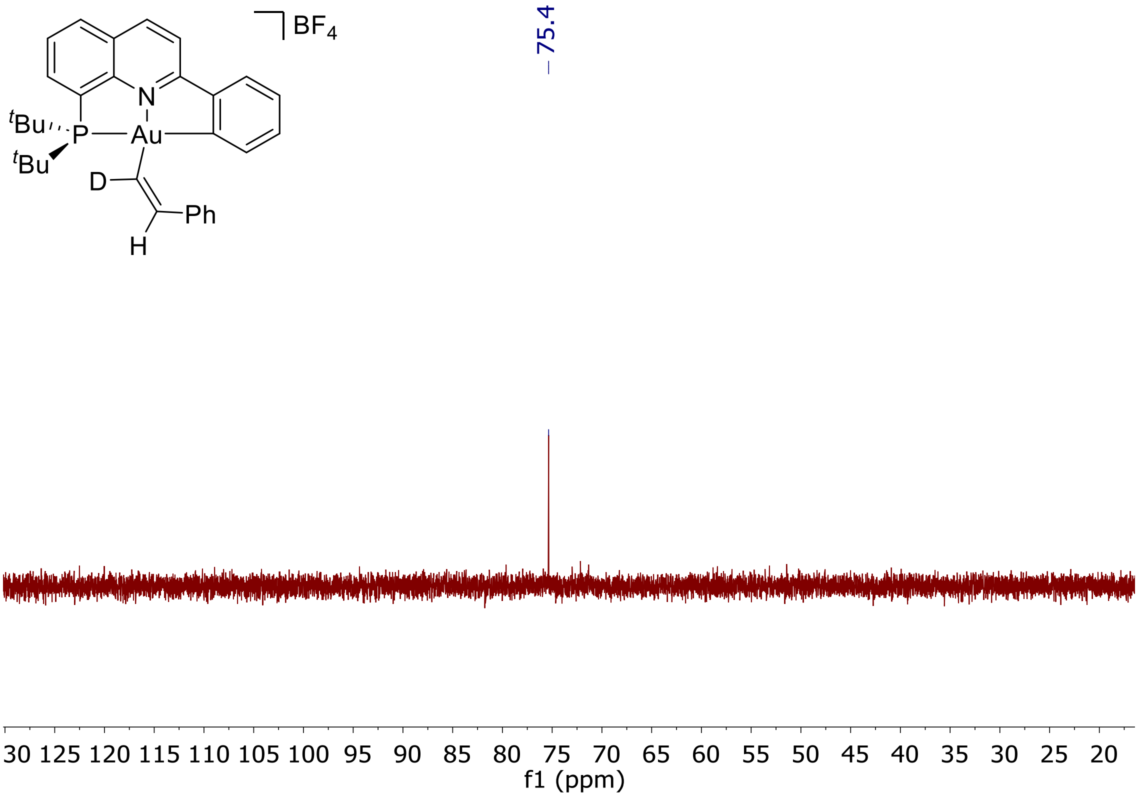

Figure S35. $^{31}P\{^{1}H\}$ NMR (161.99 MHz, CD_2Cl_2, 298 K) spectrum of compound **2-DH**.

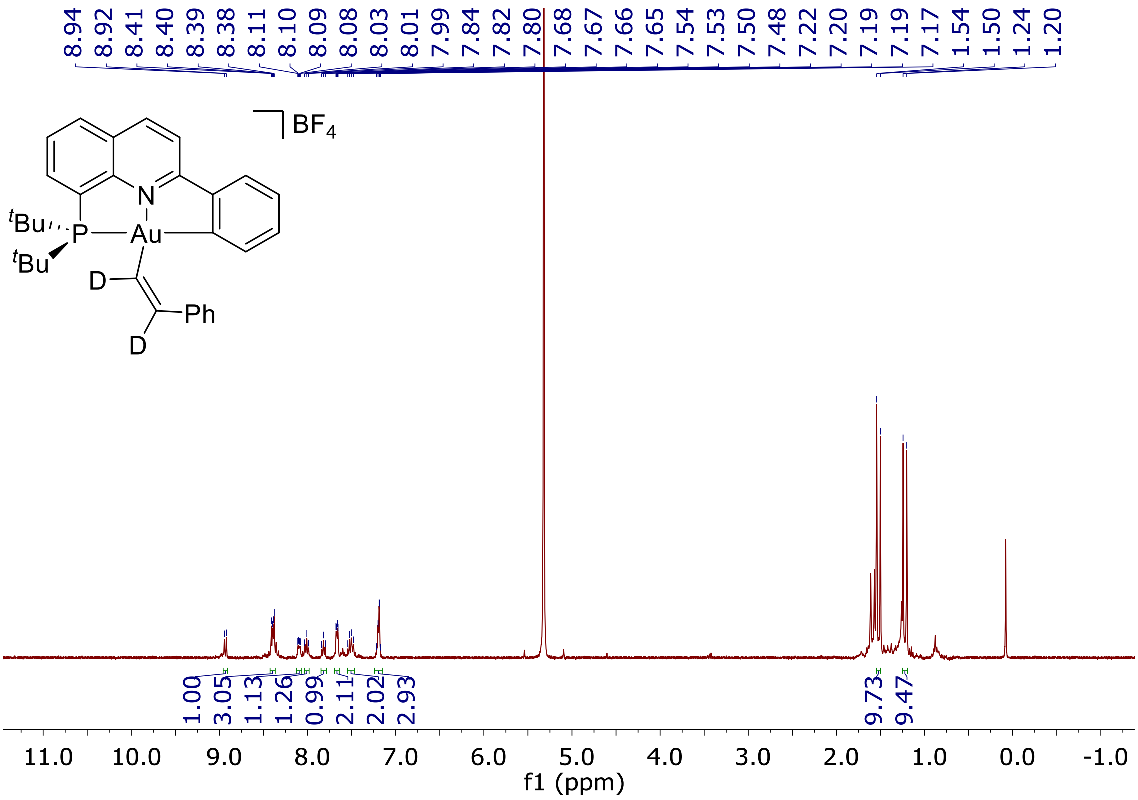

Figure S36. ^{1}H NMR (400.13 MHz, CD_2Cl_2, 298 K) spectrum of compound **2-DD**.

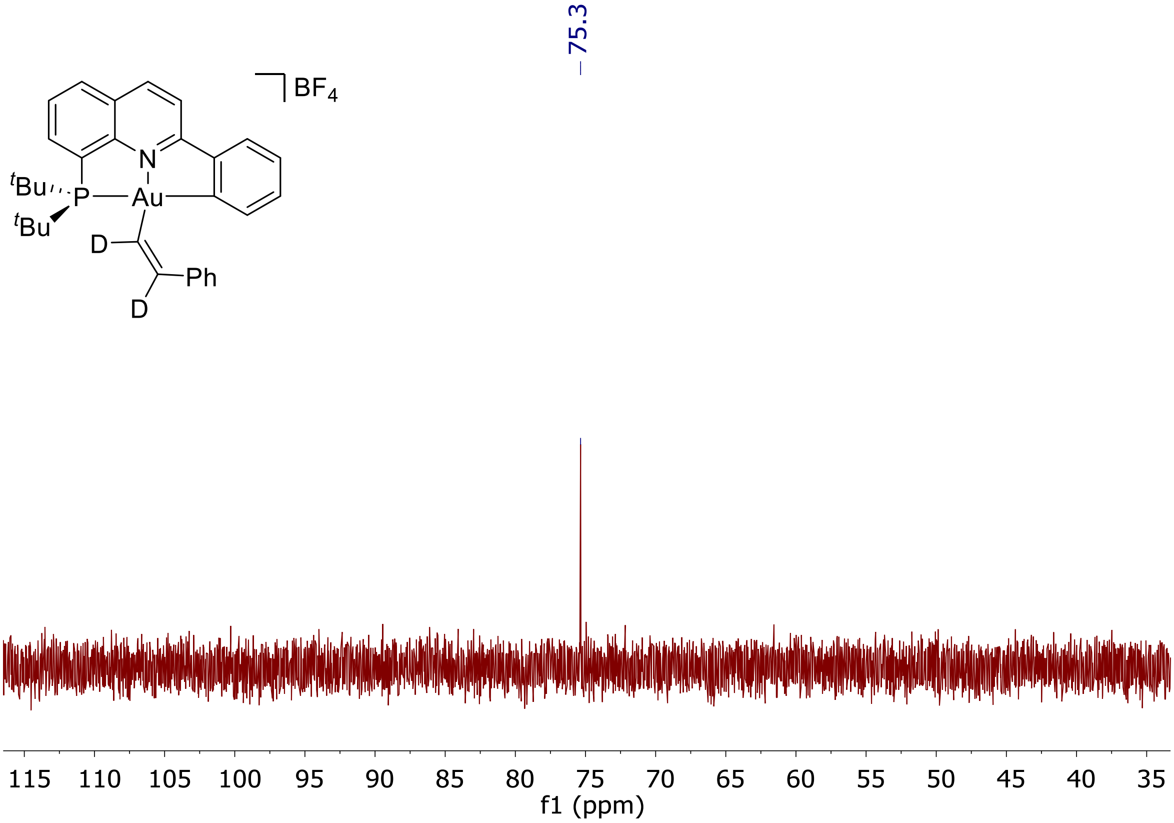

Figure S37. $^{31}P\{^{1}H\}$ NMR (161.99 MHz, CD_2Cl_2, 298 K) spectrum of compound **2-DD**.

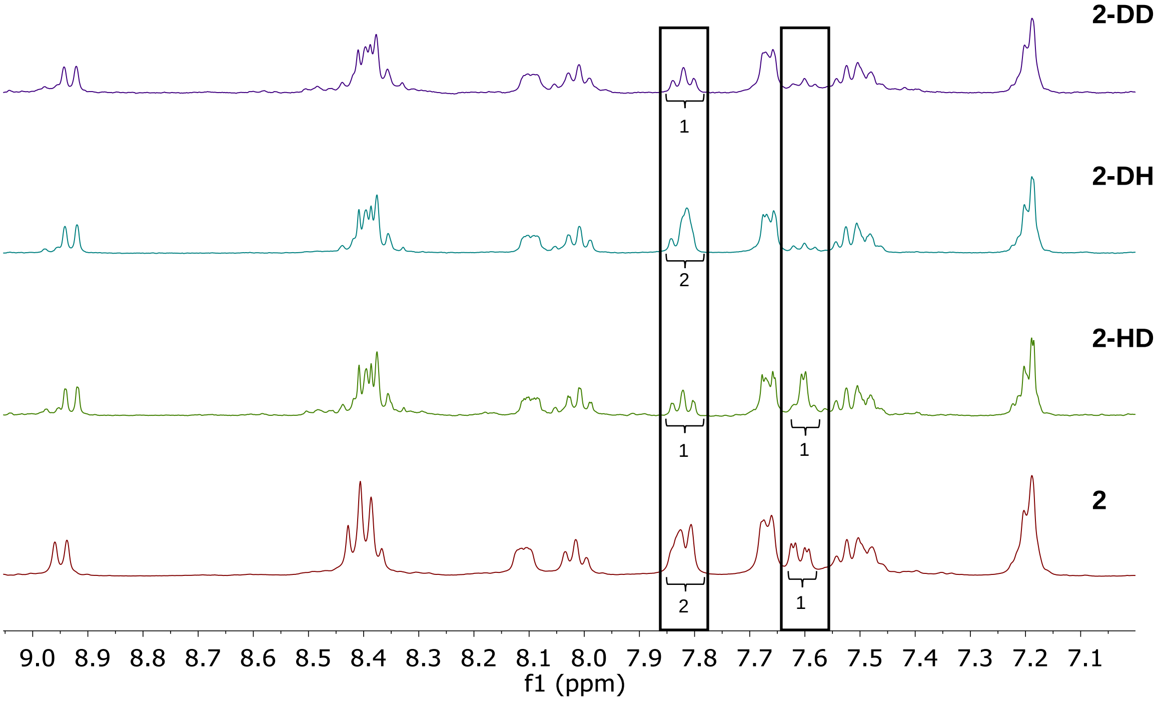

Figure S38. ^{1}H NMR spectra of the vinyl signals in compounds **2**, **2-HD**, **2-DH** and **2-DD**.

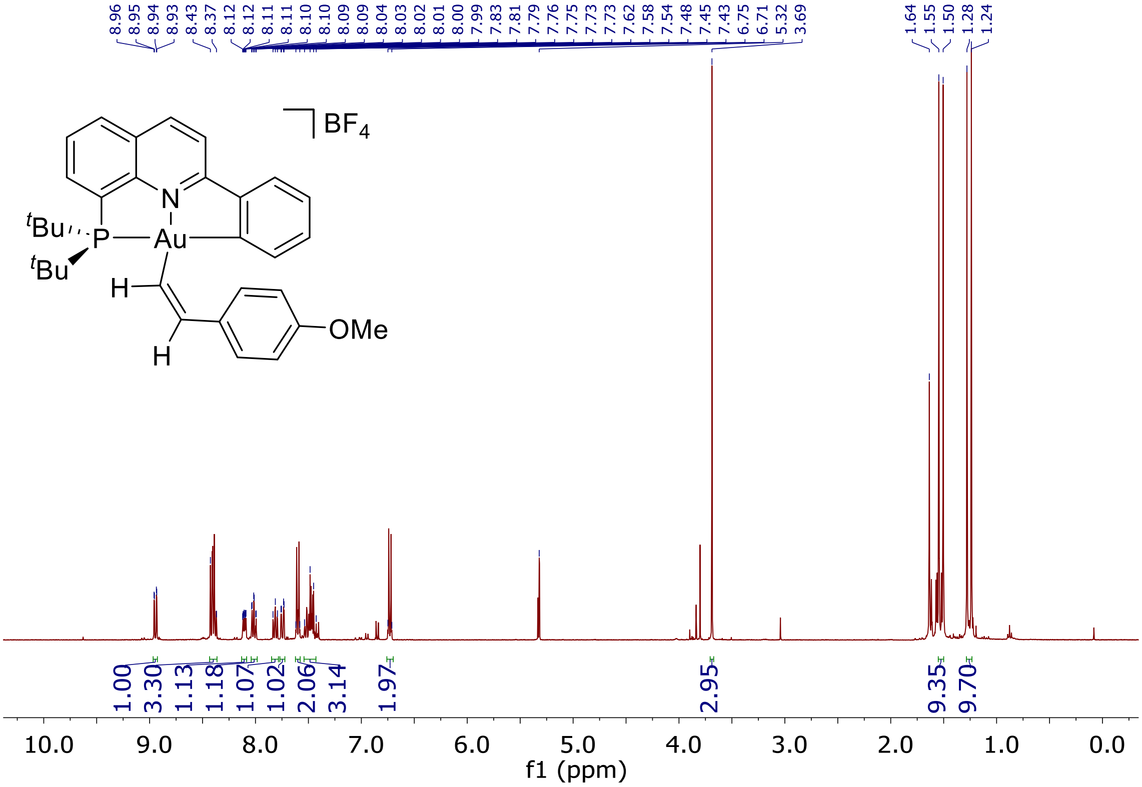

Figure S39. ^{1}H NMR (400.13 MHz, CD_2Cl_2, 298 K) spectrum of compound **3**. The ^{1}H NMR spectrum contains residual water (1.60).

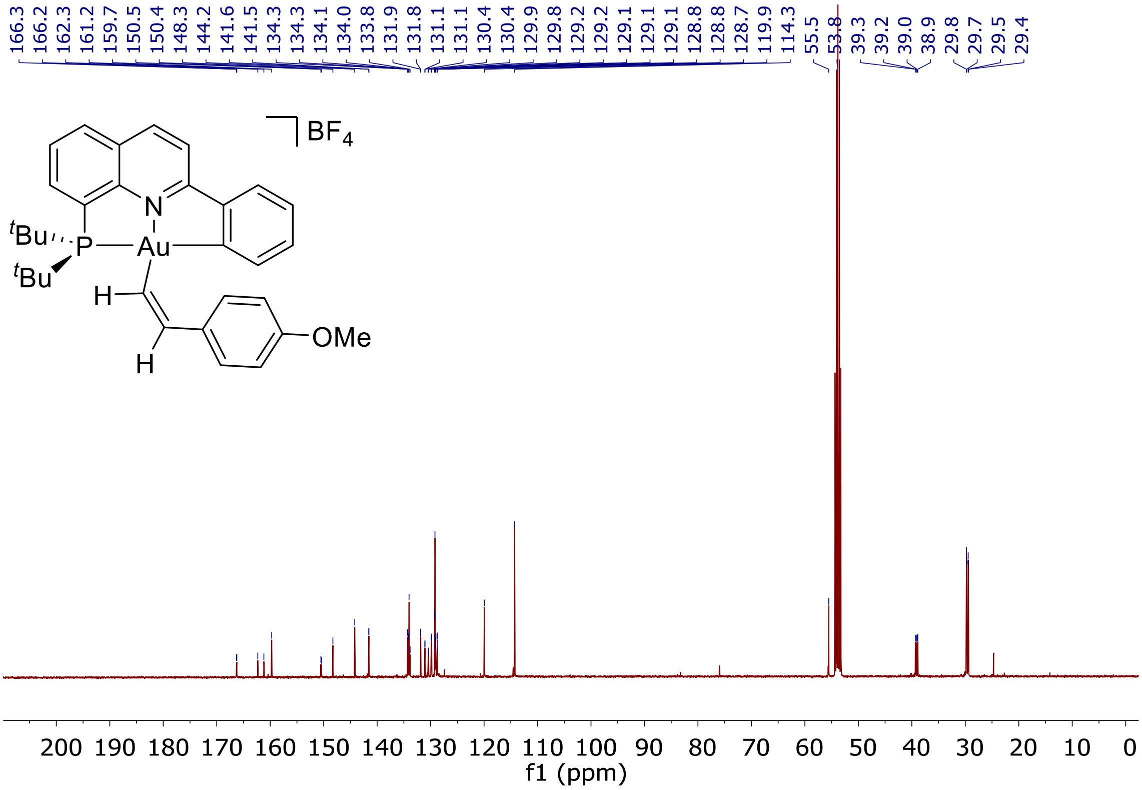

Figure S40. $^{13}C\{^1H\}$ NMR (100.62 MHz, CD_2Cl_2, 298 K) spectrum of compound **3**.

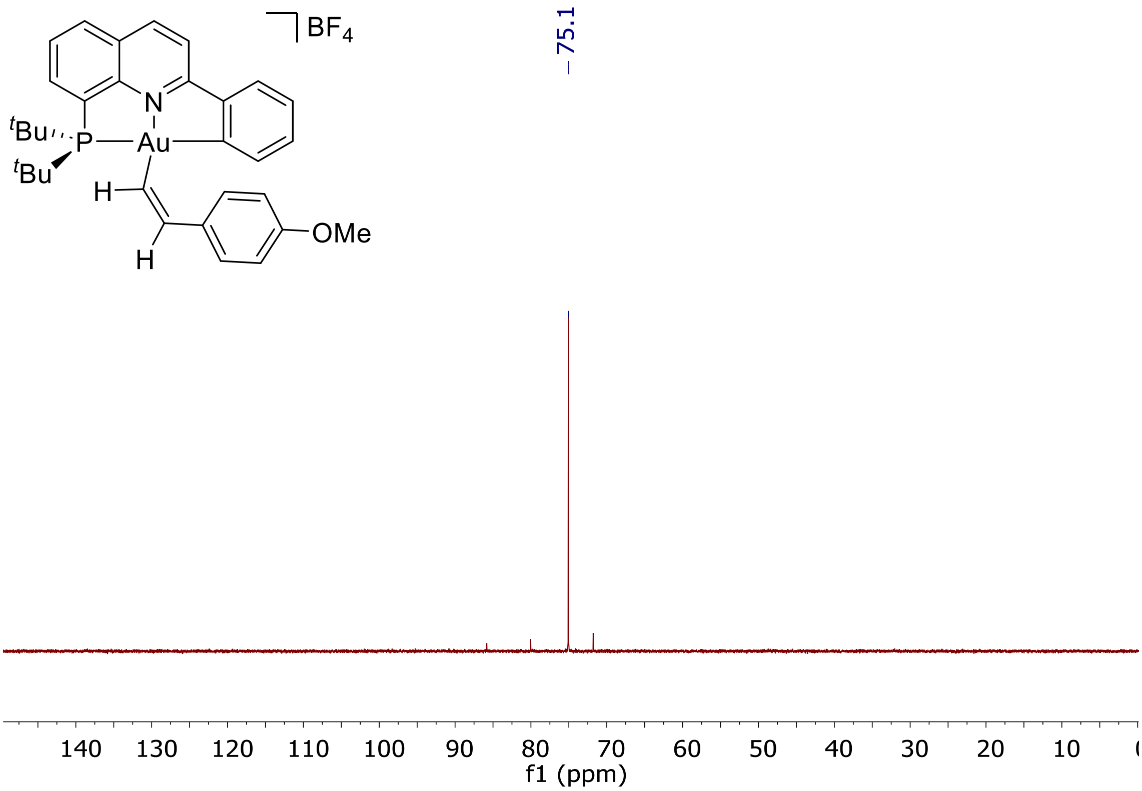

Figure S41. $^{31}P\{^1H\}$ NMR (161.99 MHz, CD_2Cl_2, 298 K) spectrum of compound **3**.

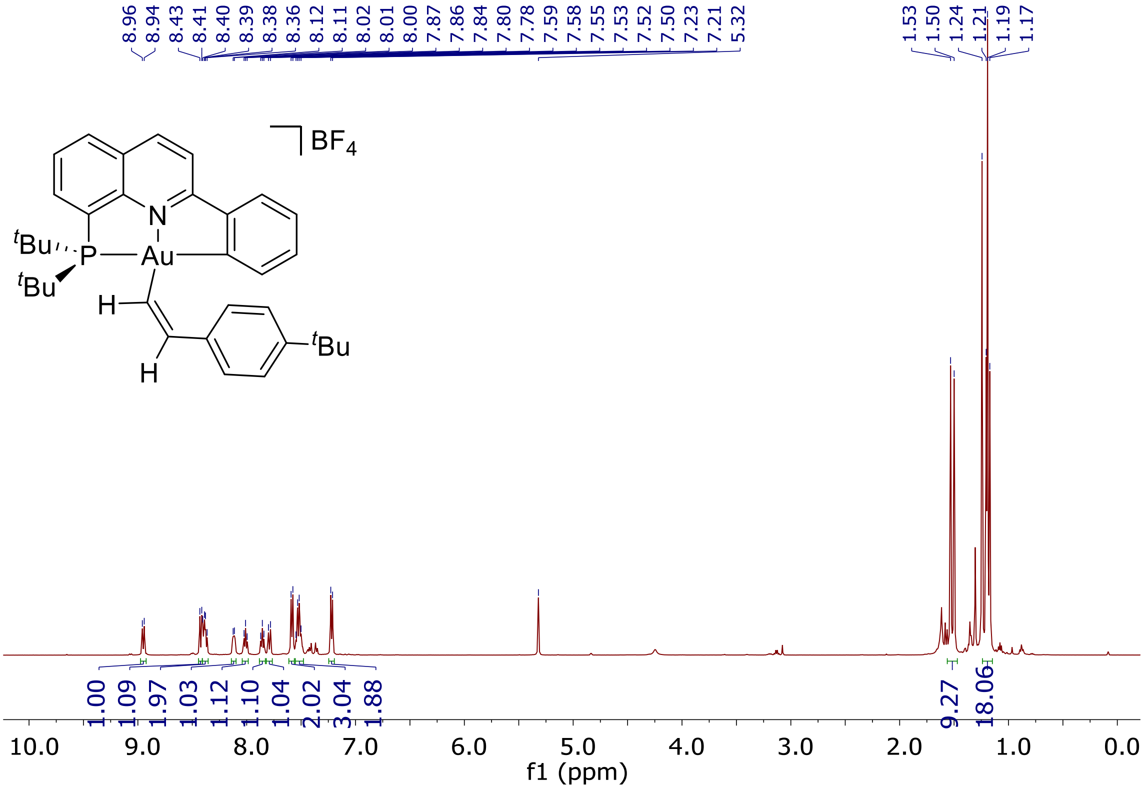

Figure S42. ^{1}H NMR (500.30 MHz, CD_2Cl_2, 298 K) spectrum of compound **4**.

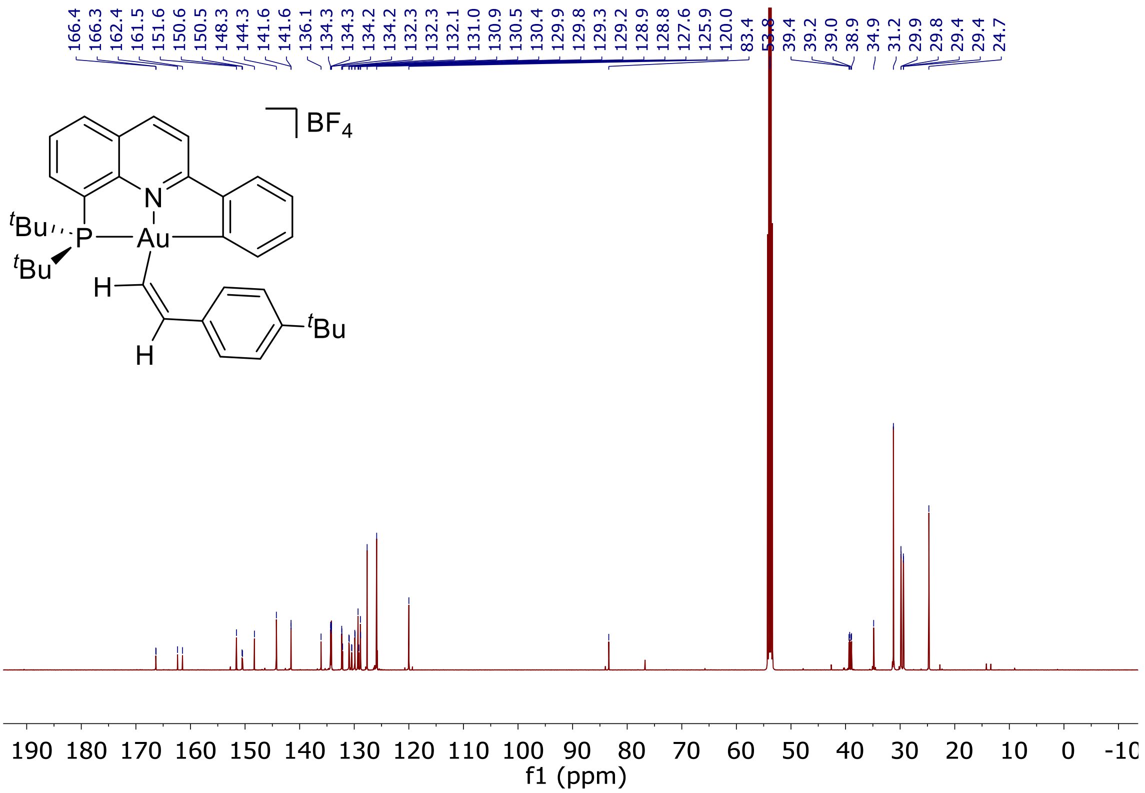

Figure S43. $^{13}C\{^{1}H\}$ NMR (125.81 MHz, CD_2Cl_2, 298 K) spectrum of compound **4**. Impurities of $O(B_2pin_2)$ at 83.3 and 24.7 ppm.

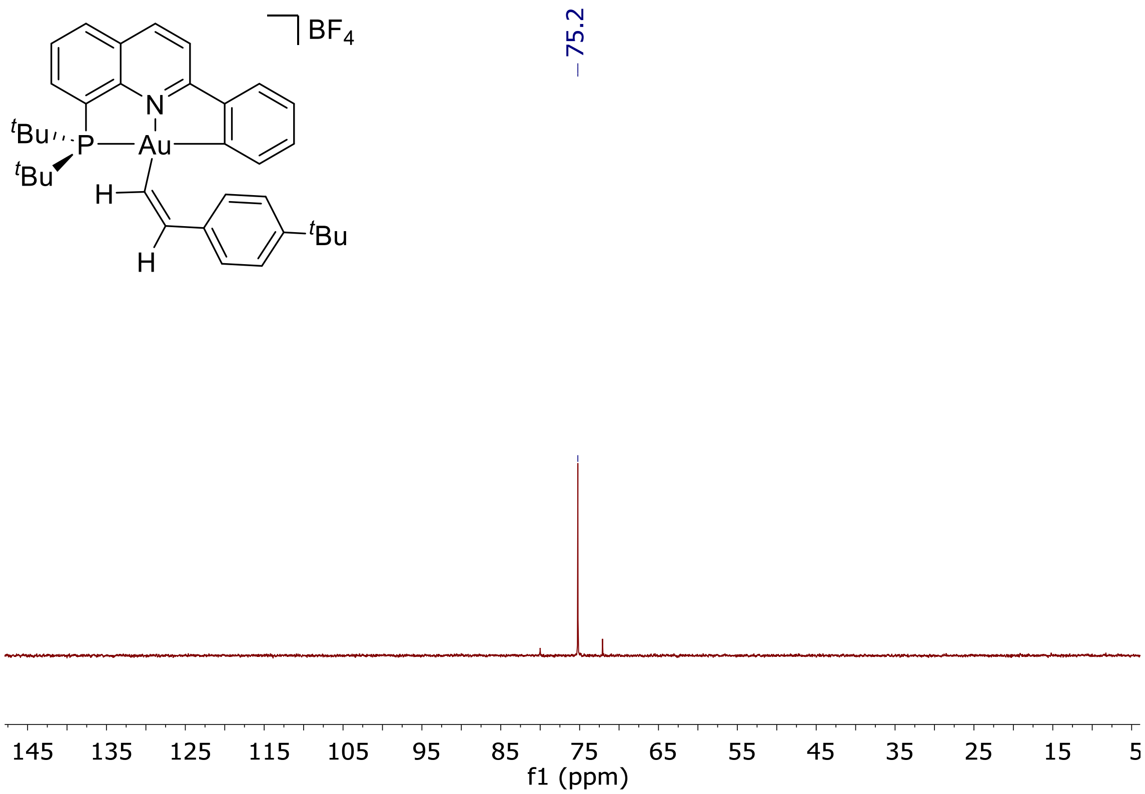

Figure S44. $^{31}P\{^{1}H\}$ NMR (161.99 MHz, CD_2Cl_2, 298 K) spectrum of compound **4**.

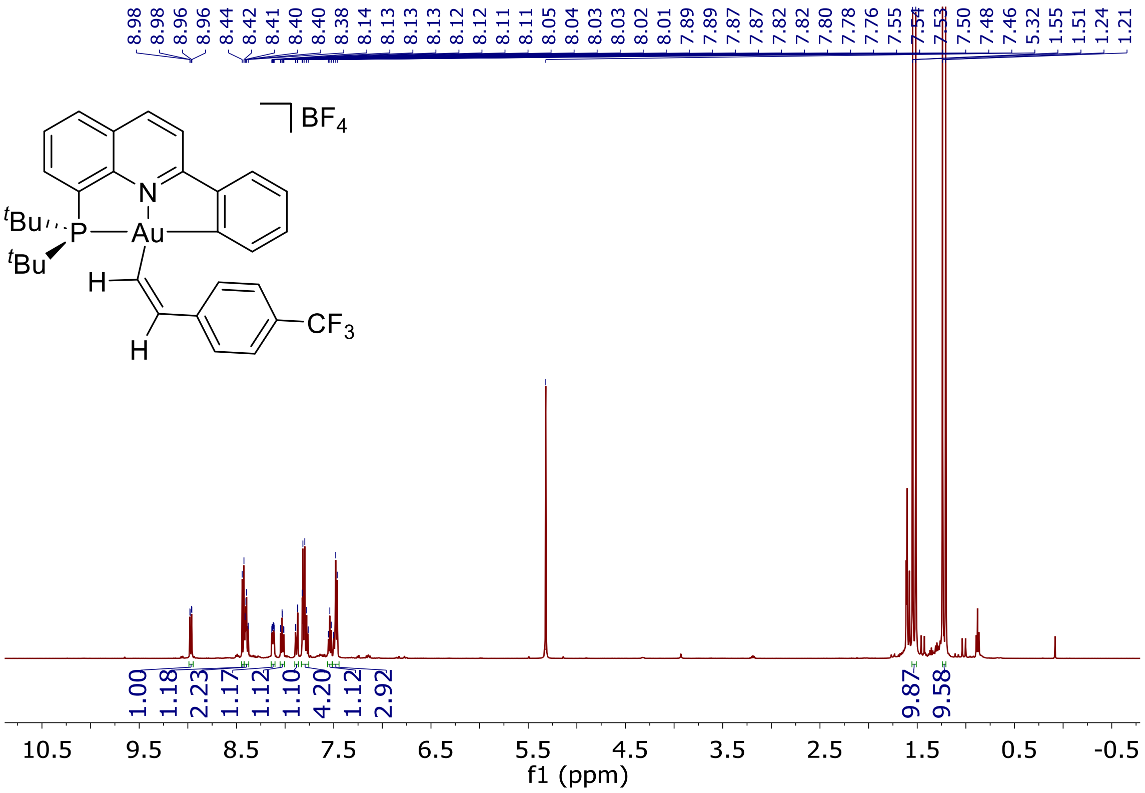

Figure S45. ^{1}H NMR (500.13 MHz, CD_2Cl_2, 298 K) spectrum of compound **5**.

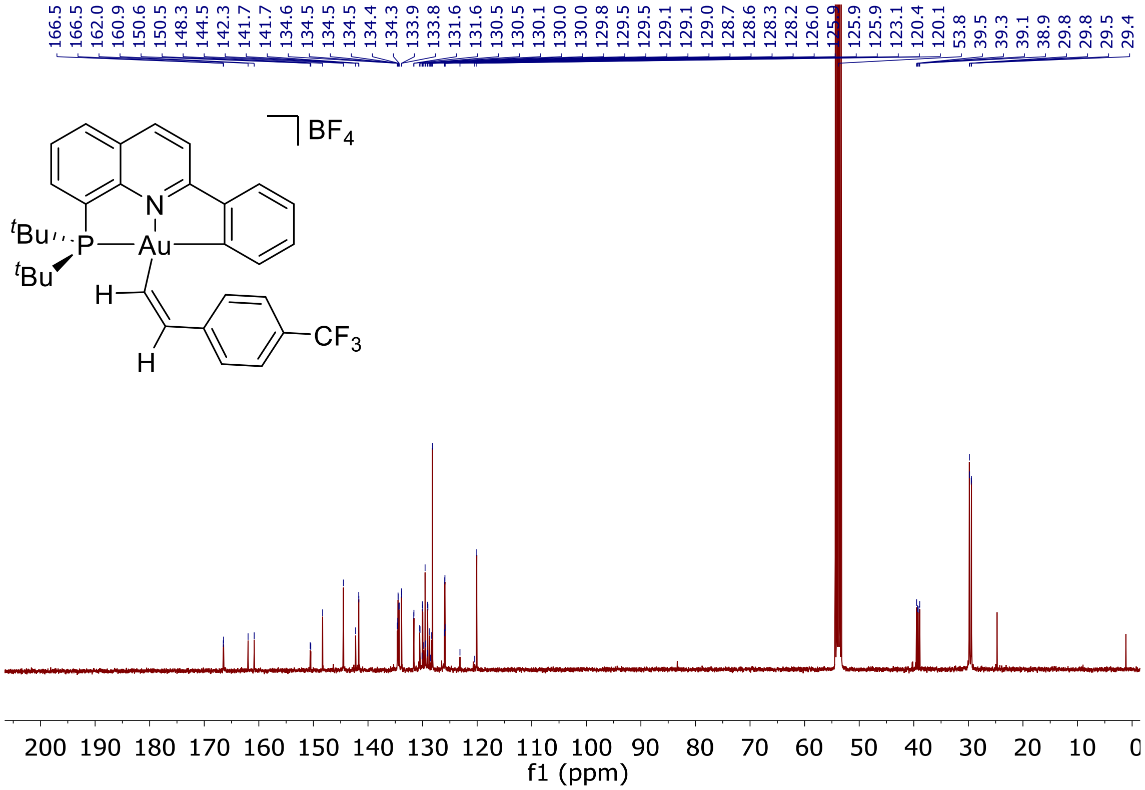

Figure S46. $^{13}C\{^{1}H\}$ NMR (125.77 MHz, CD_2Cl_2, 298 K) spectrum of compound **5**. Impurities of pentane at 34.5, 22.7 and 14.2 ppm.

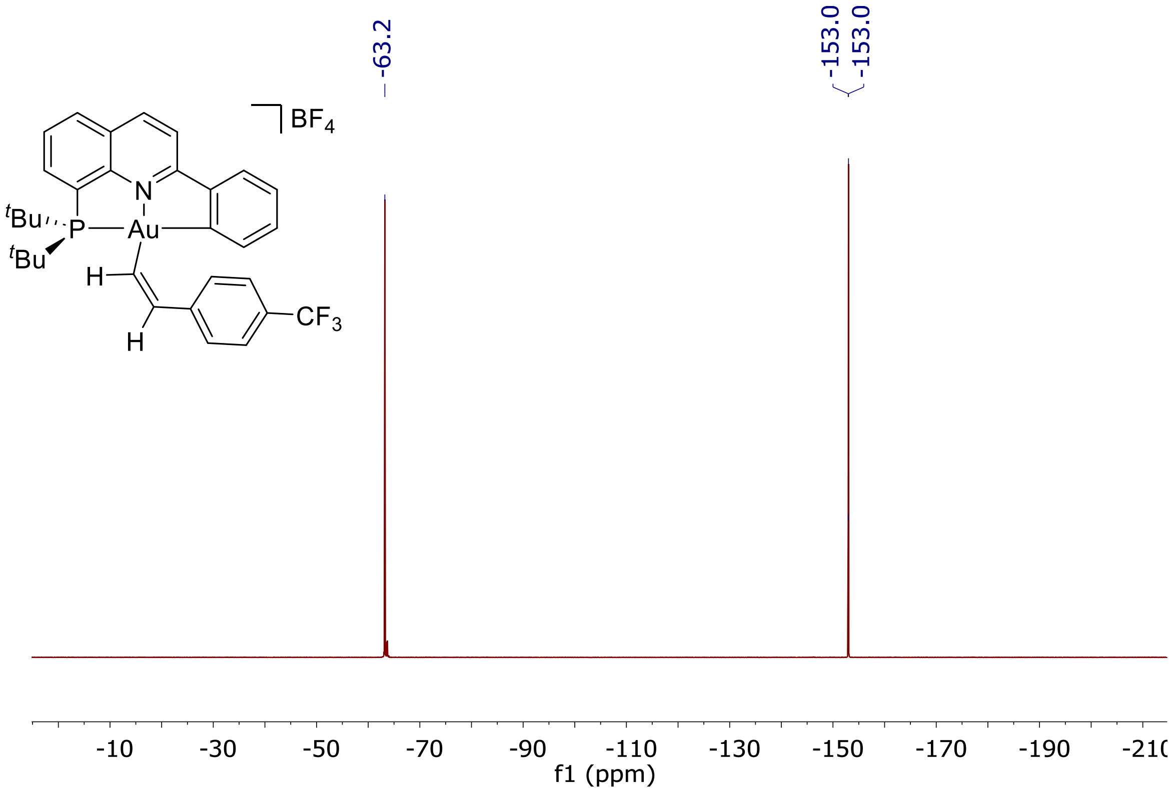

Figure S47. $^{19}F\{^{1}H\}$ NMR (470.71 MHz, CD_2Cl_2, 298 K) spectrum of compound **5**.

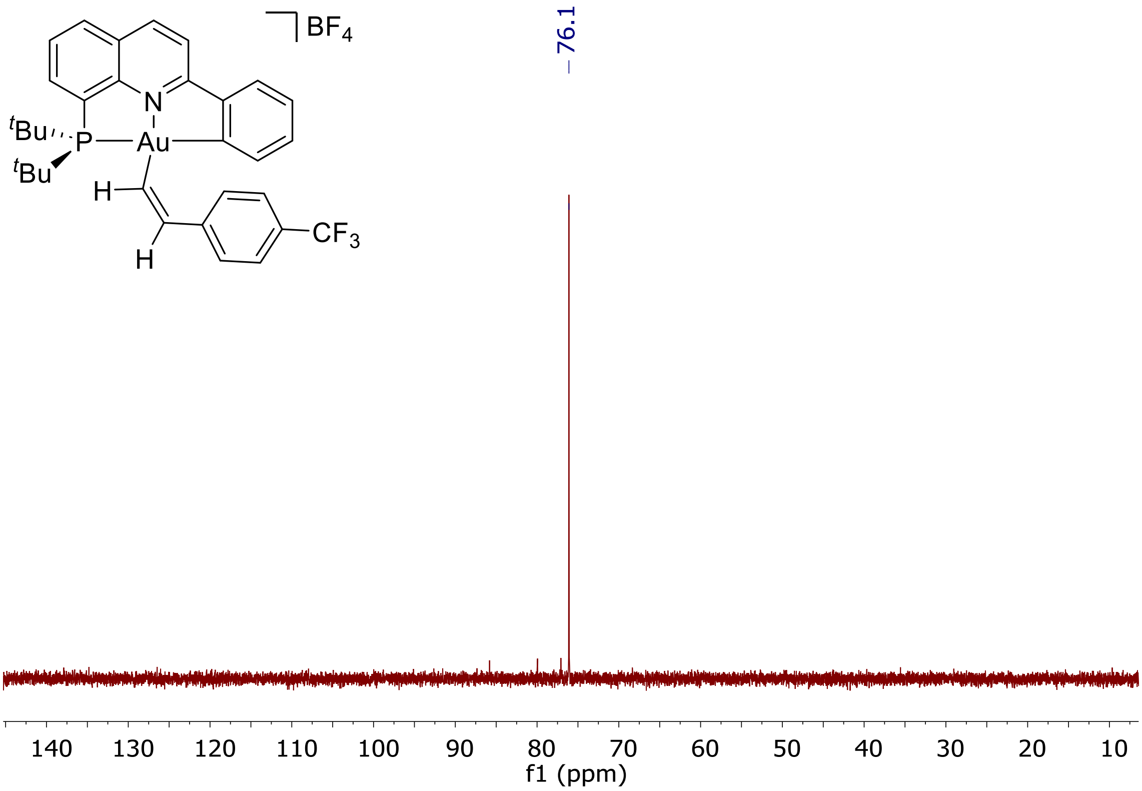

Figure S48. $^{31}P\{^{1}H\}$ NMR (202.52 MHz, CD_2Cl_2, 298 K) spectrum of compound **5**.

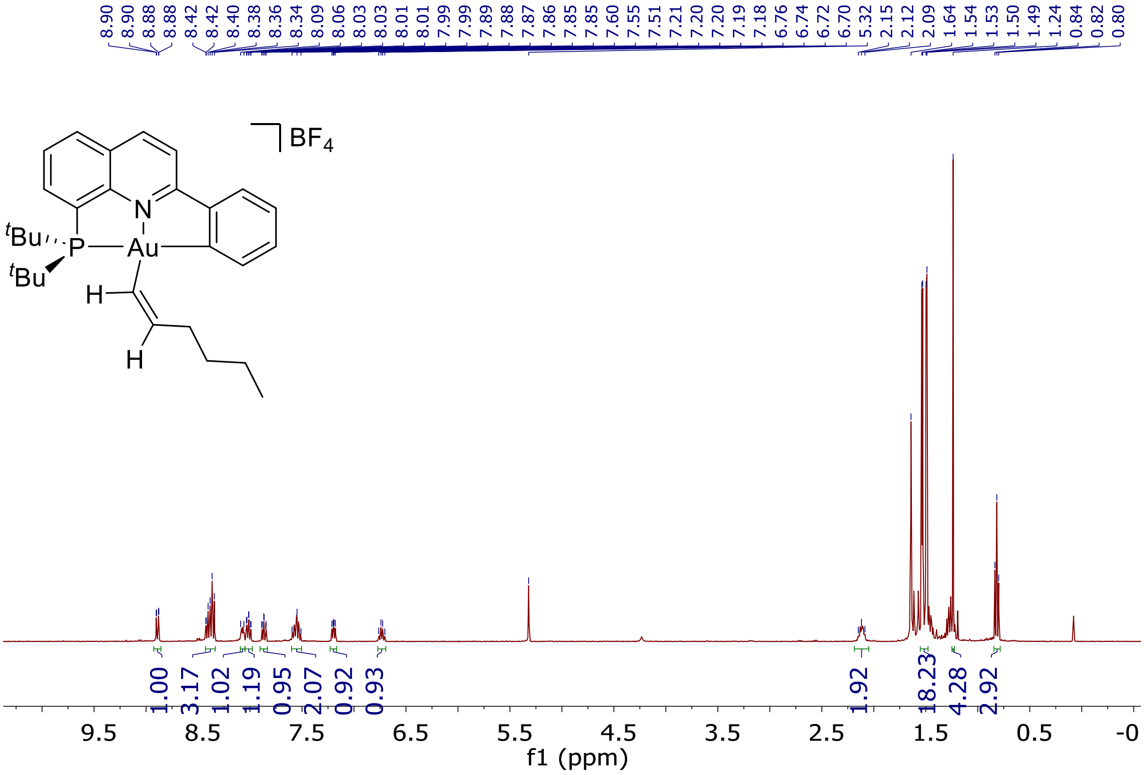

Figure S49. ^{1}H NMR (400.13 MHz, CD_2Cl_2, 298 K) spectrum of compound **6**. Residual water at 1.64 ppm.

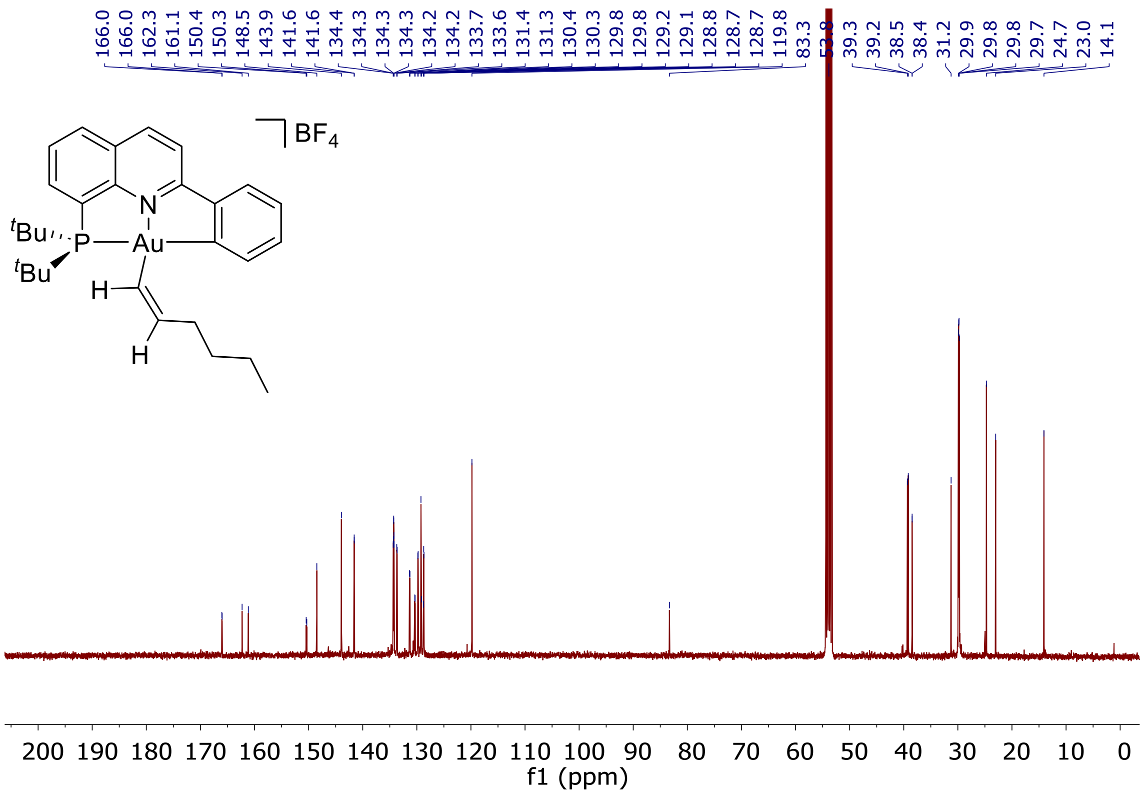

Figure S50. $^{13}C\{^{1}H\}$ NMR (100.62 MHz, CD_2Cl_2, 298 K) spectrum of compound **6**. Traces of $O(B_2pin_2)$ can be detected at 83.3 and 24.7 ppm in ^{13}C NMR.

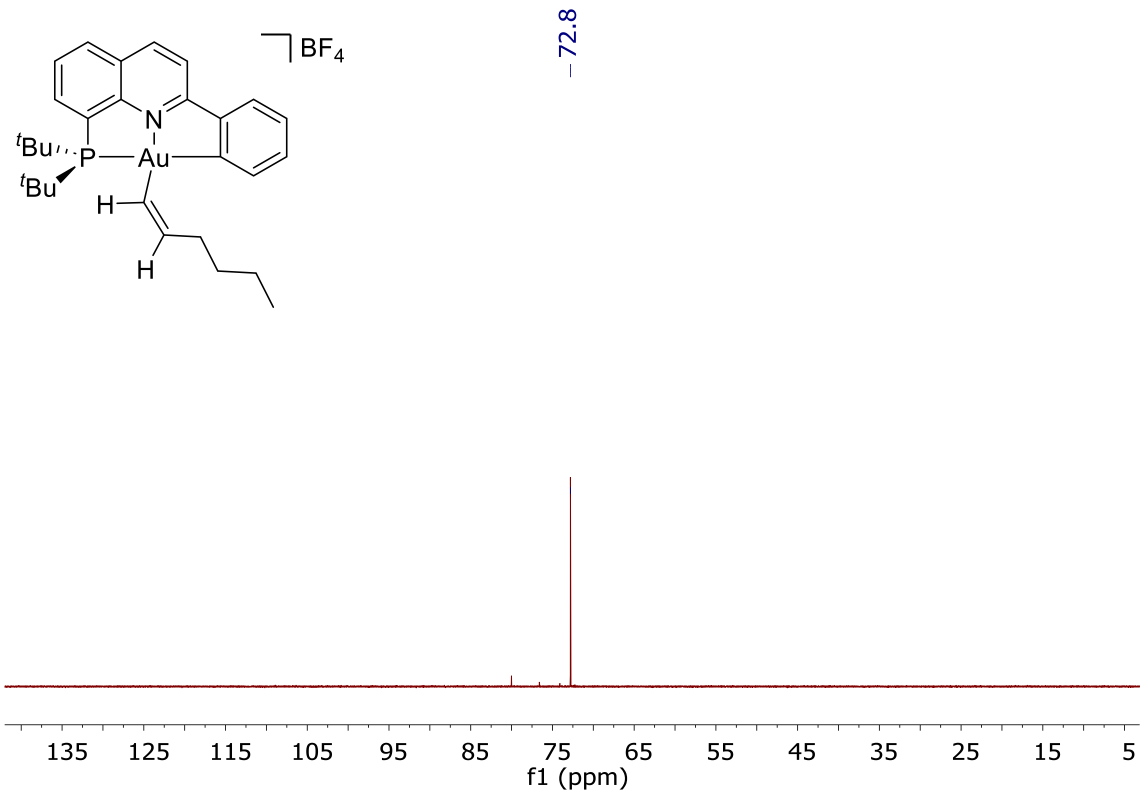

Figure S51. $^{31}P\{^{1}H\}$ NMR (161.99 MHz, CD_2Cl_2, 298 K) spectrum of compound **6**.

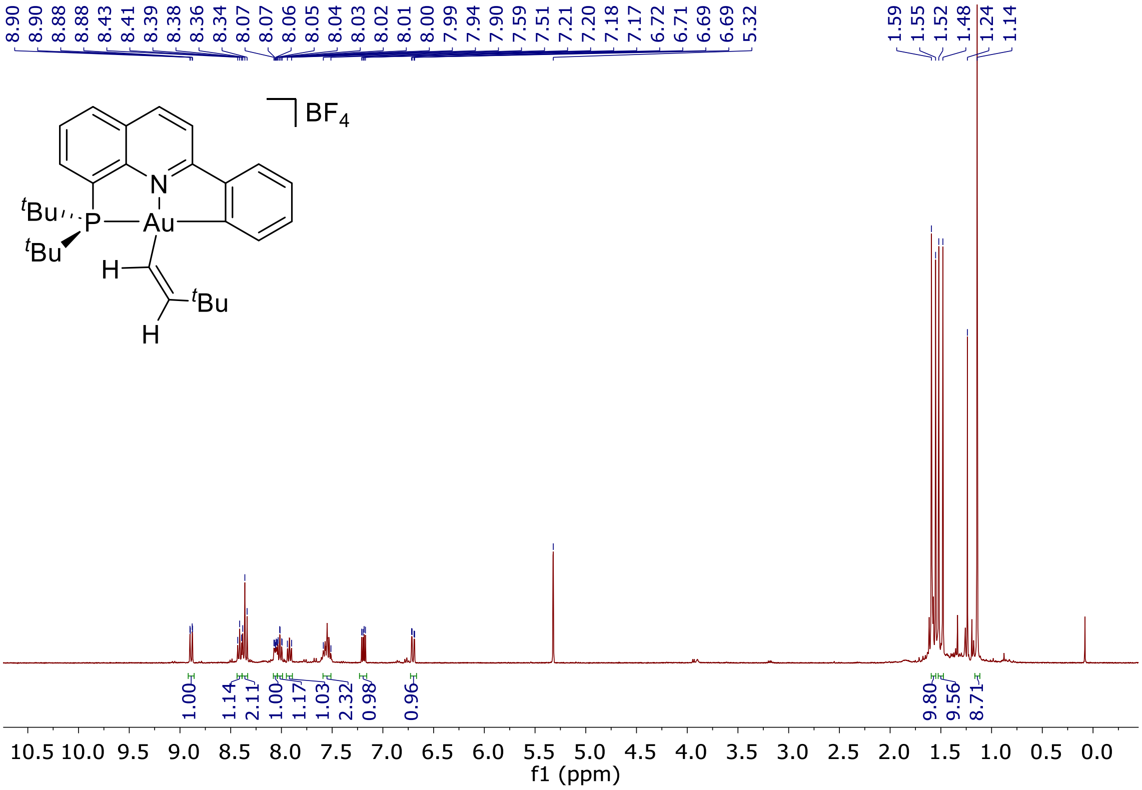

Figure S52. ^{1}H NMR (400.13 MHz, CD_2Cl_2, 298 K) spectrum of compound **7**.

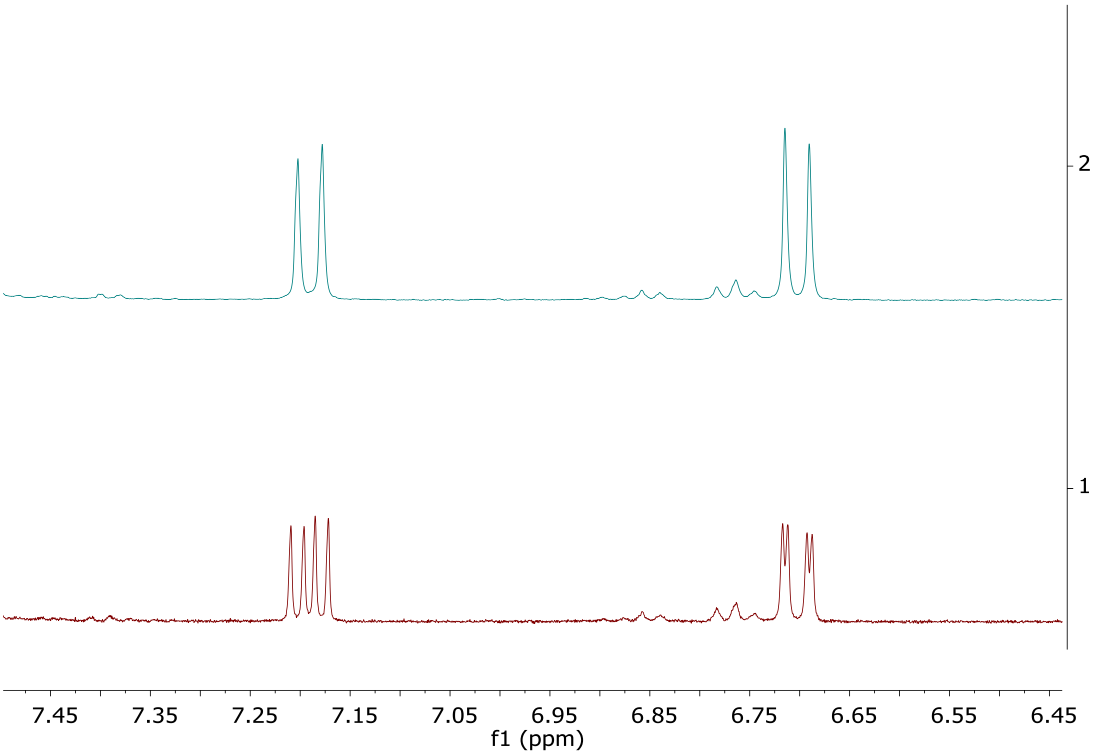

Figure S53. Comparison between ^{1}H (bottom) and $^{1}H\{^{31}P\}$ (top) NMR (400.13 MHz, CD_2Cl_2, 298 K) spectra of the vinylic signals in compound **7**.

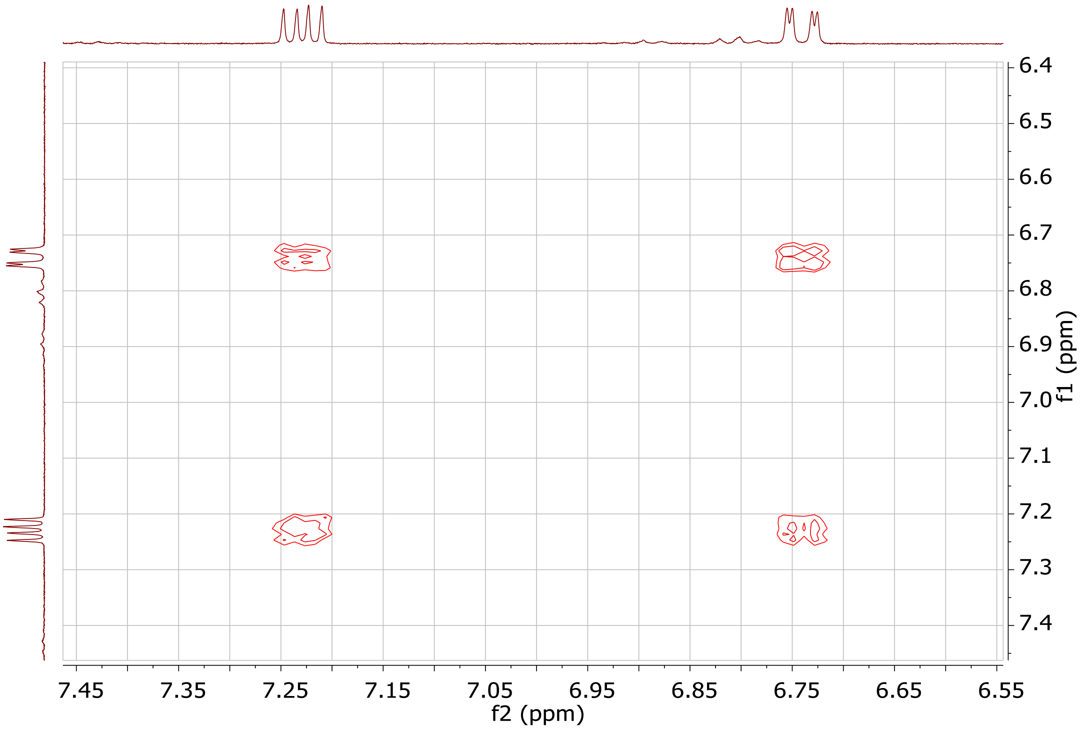

Figure S54. ^{1}H-^{1}H COSY NMR (400.13 MHz, CD_2Cl_2, 298 K) spectra of the compound **7**. Expansion of the aromatic region.

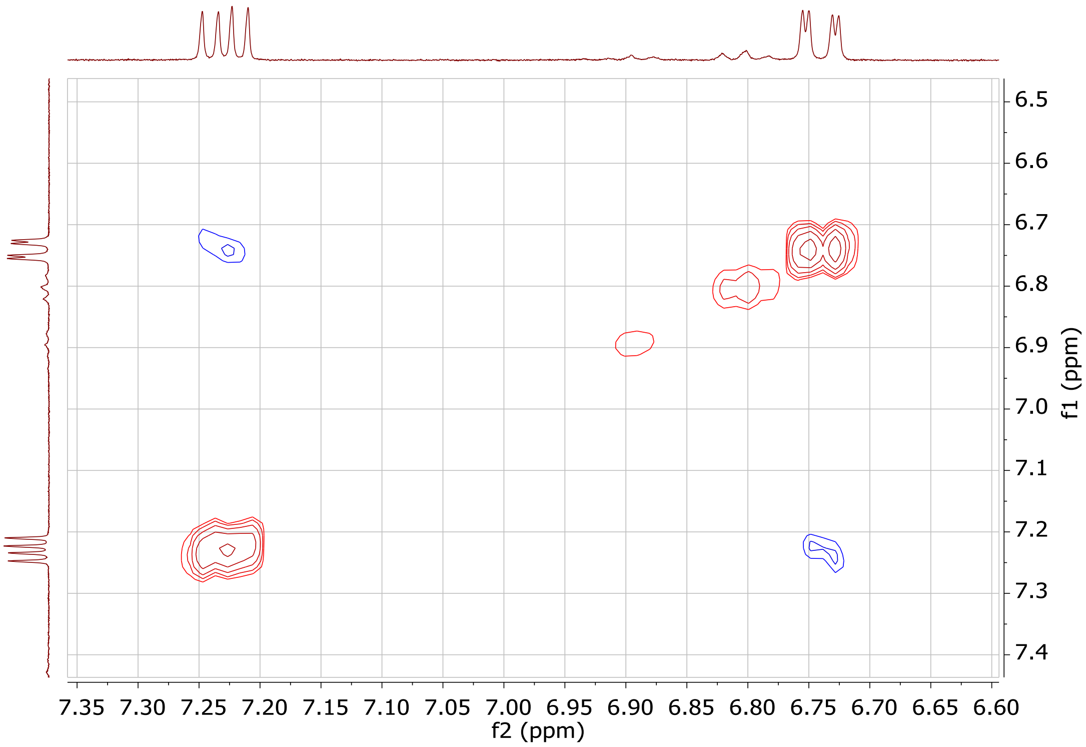

Figure S55. ^{1}H-^{1}H NOESY NMR (400.13 MHz, CD_2Cl_2, 298 K) spectra of the compound **7**. Expansion of the aromatic region.

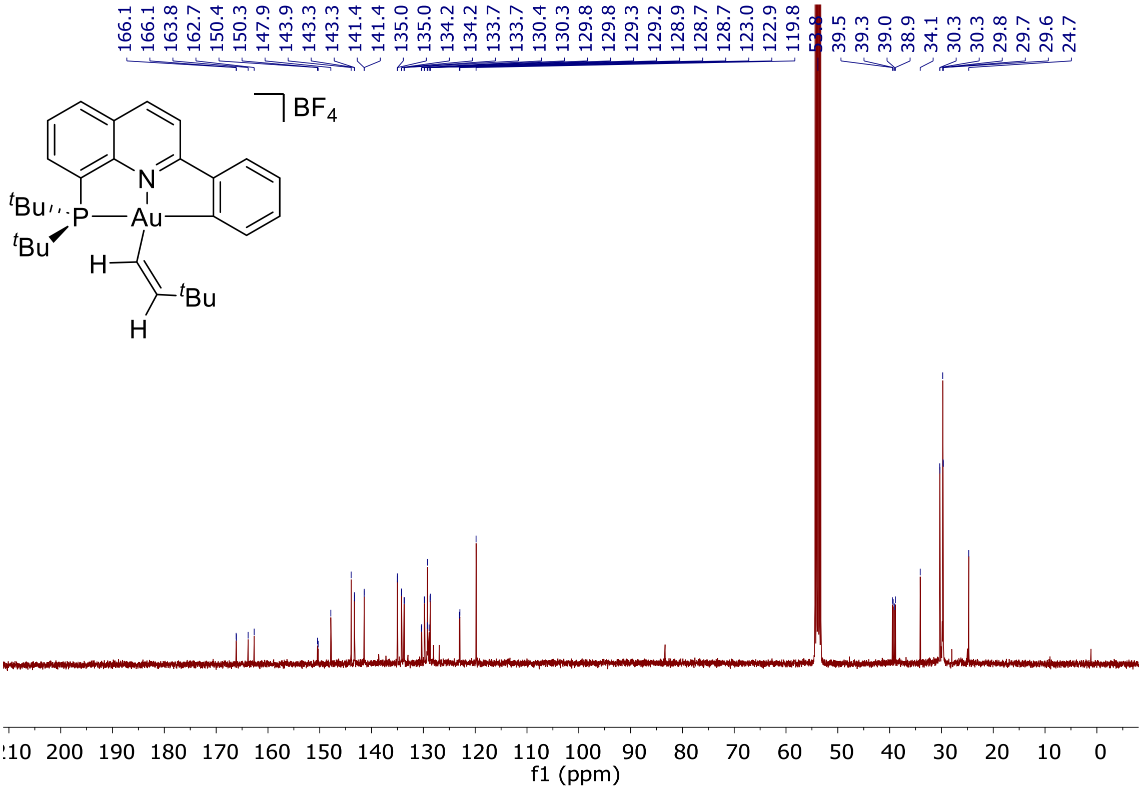

Figure S56. $^{13}C\{^1H\}$ NMR (100.62 MHz, CD_2Cl_2, 298 K) spectrum of compound **7**.

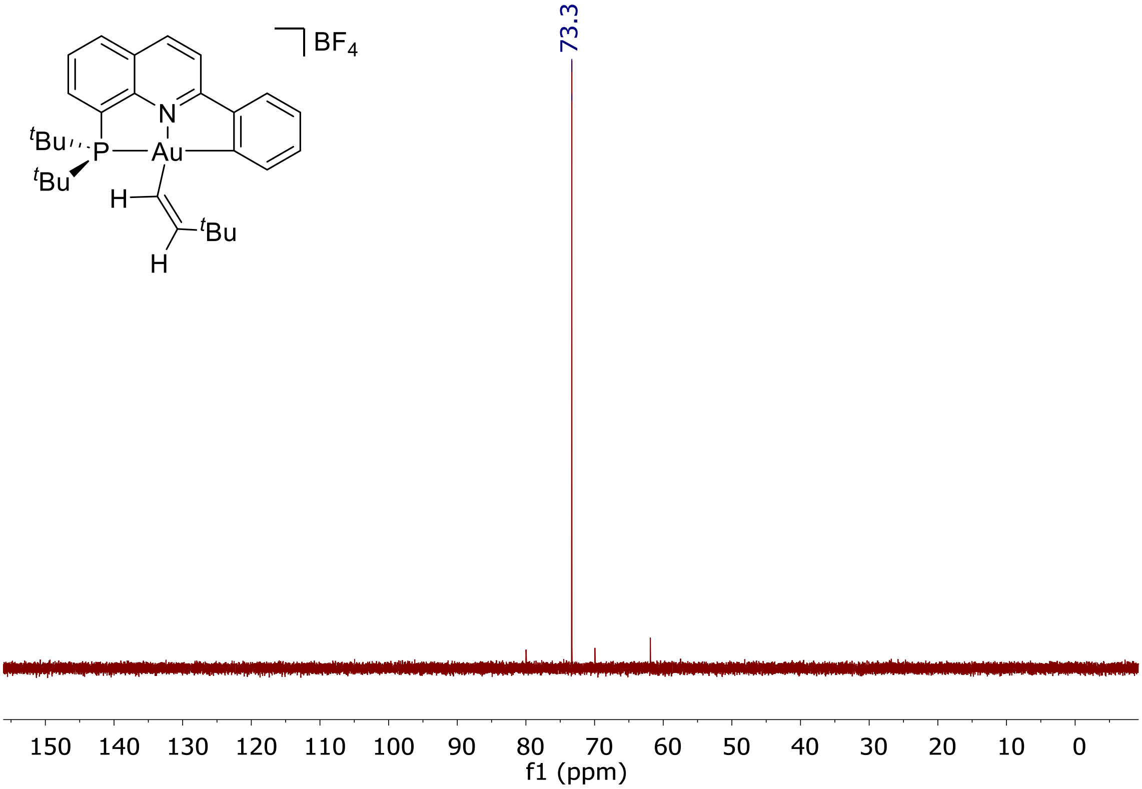

Figure S57. $^{31}P\{^1H\}$ NMR (161.99 MHz, CD_2Cl_2, 298 K) spectrum of compound **7**.

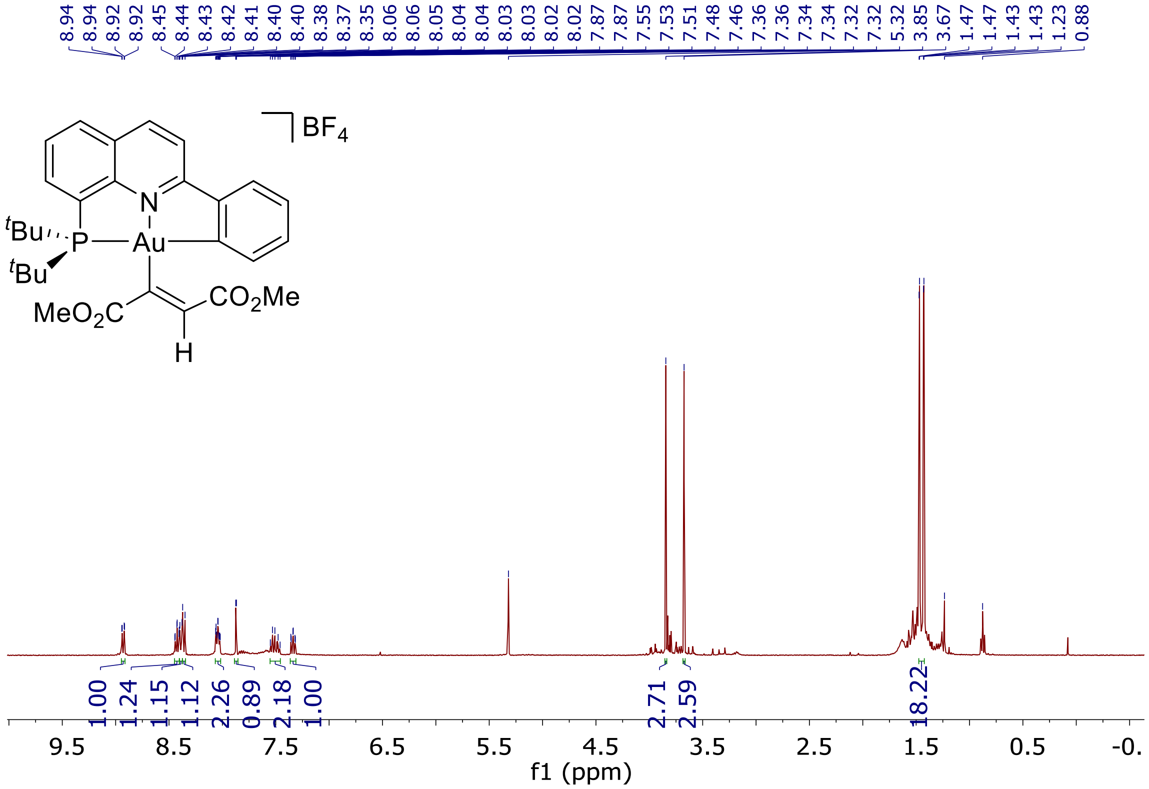

Figure S58. ^{1}H NMR (400.13 MHz, CD_2Cl_2, 298 K) spectrum of compound **8**. Residual pentane in the ^{1}H NMR spectrum (1.23, 0.88).

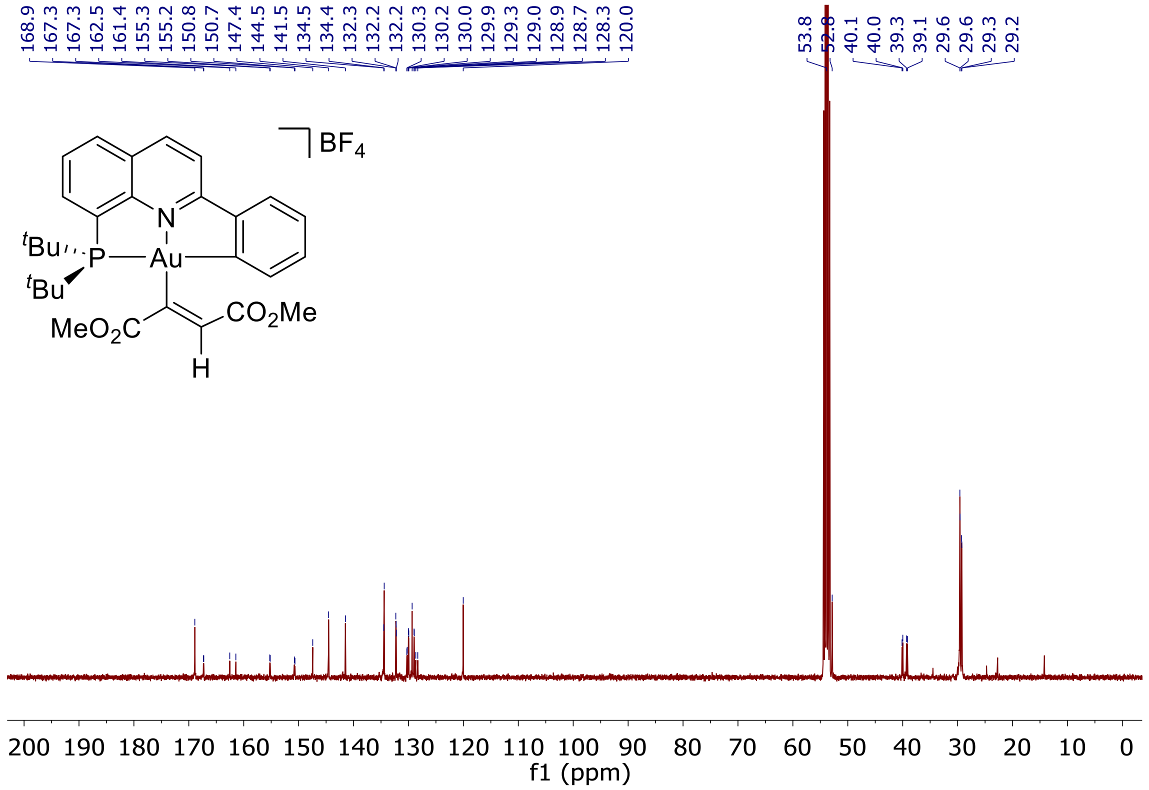

Figure S59. $^{13}C\{^{1}H\}$ NMR (100.62 MHz, CD_2Cl_2, 298 K) spectrum of compound **8**.

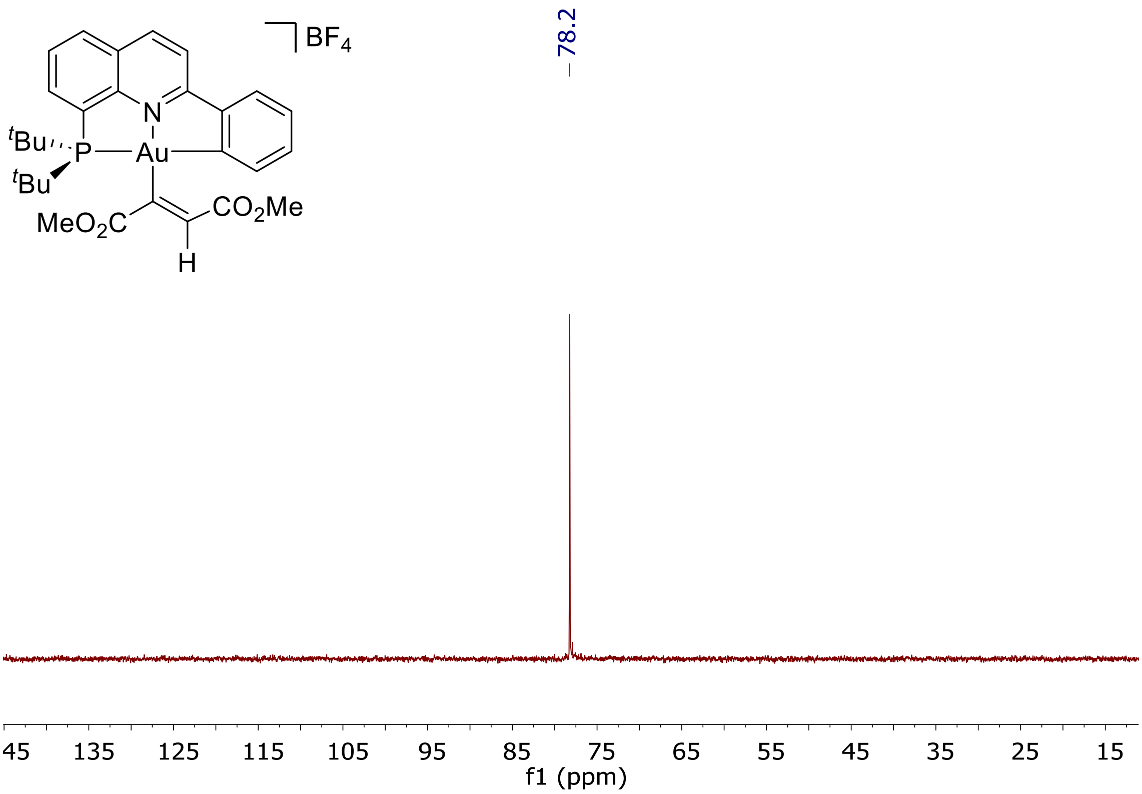

Figure S60. $^{31}P\{^{1}H\}$ NMR (161.99 MHz, CD_2Cl_2, 298 K) spectrum of compound **8**.

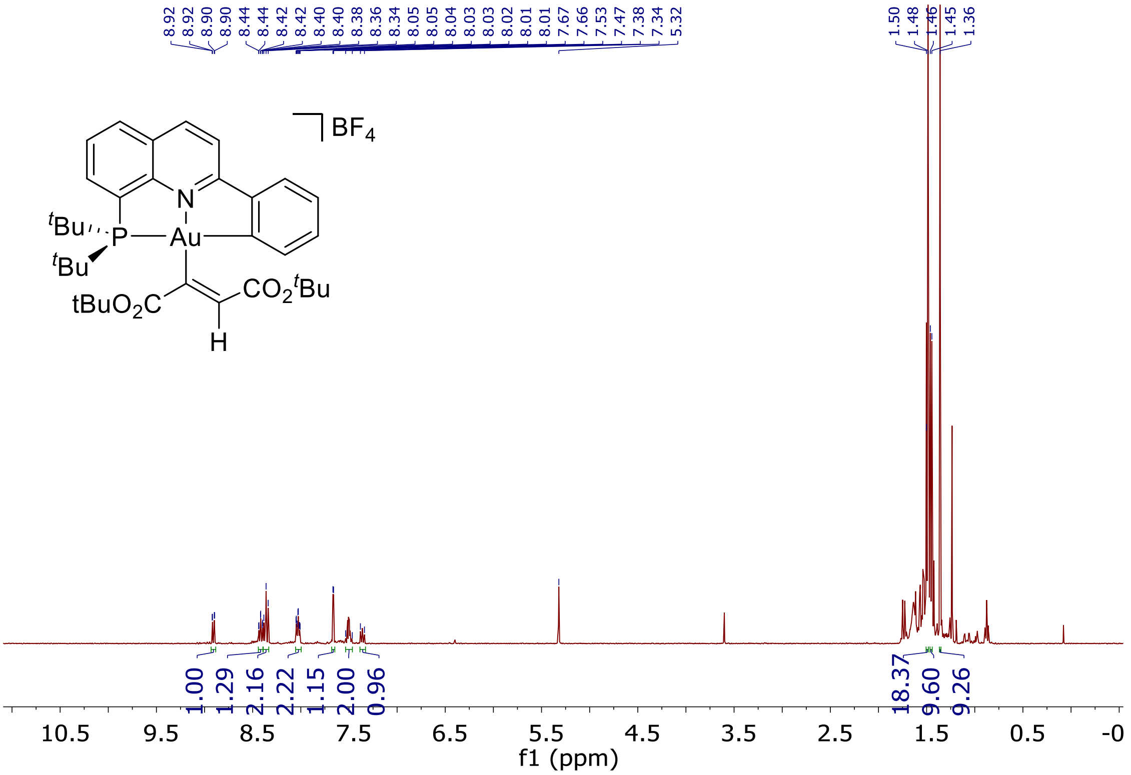

Figure S61. ^{1}H NMR (400.13 MHz, CD_2Cl_2, 298 K) spectrum of compound **9**.

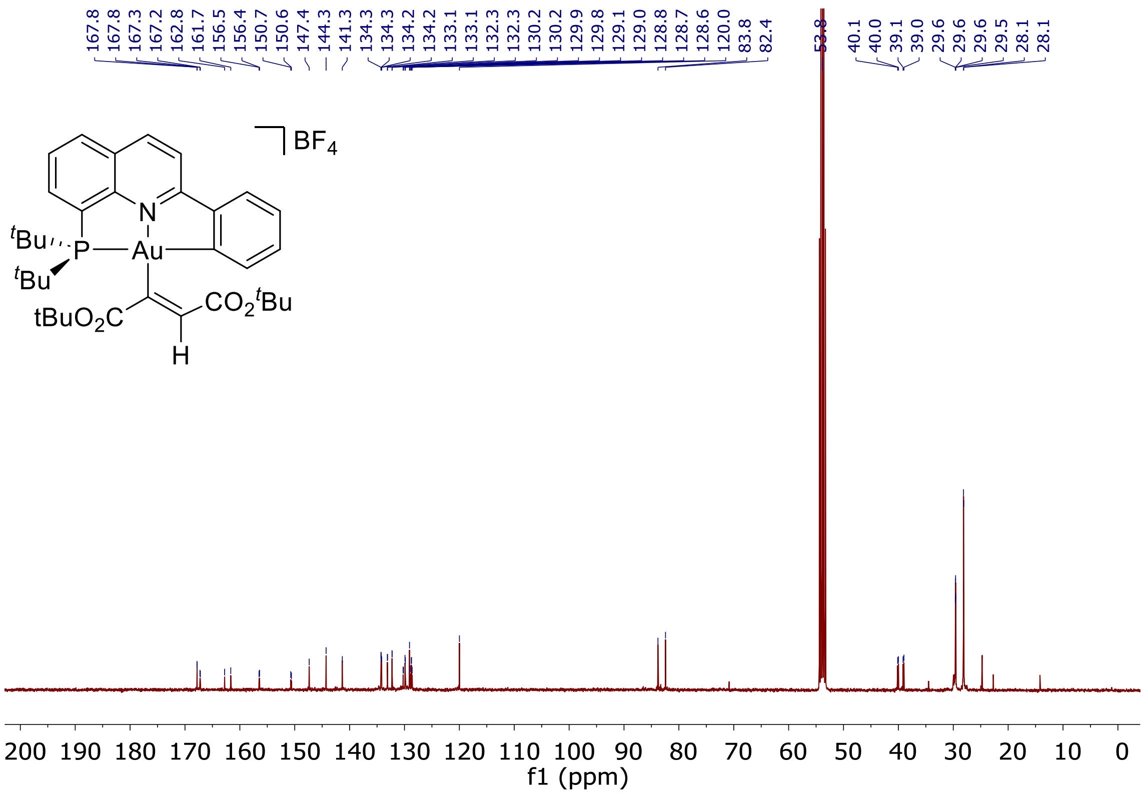

Figure S62. $^{13}C\{^{1}H\}$ NMR (100.62 MHz, CD_2Cl_2, 298 K) spectrum of compound **9**.

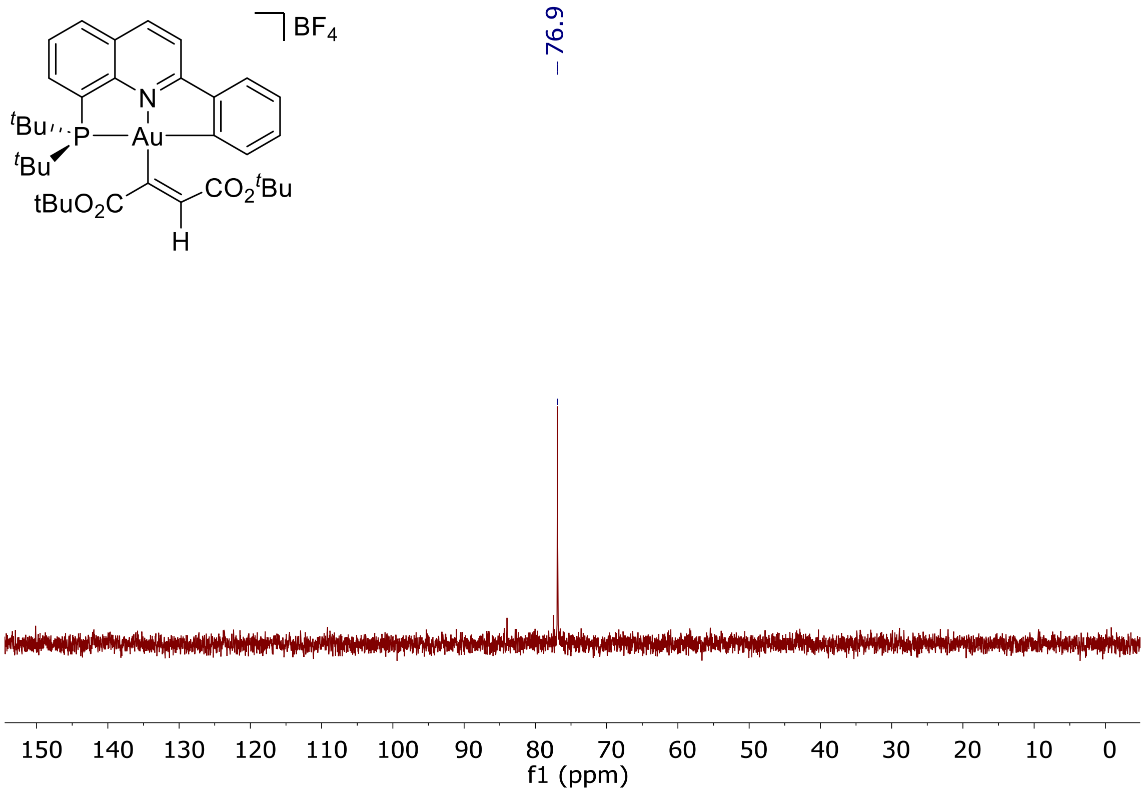

Figure S63. $^{31}P\{^{1}H\}$ NMR (161.99 MHz, CD_2Cl_2, 298 K) spectrum of compound **9**.

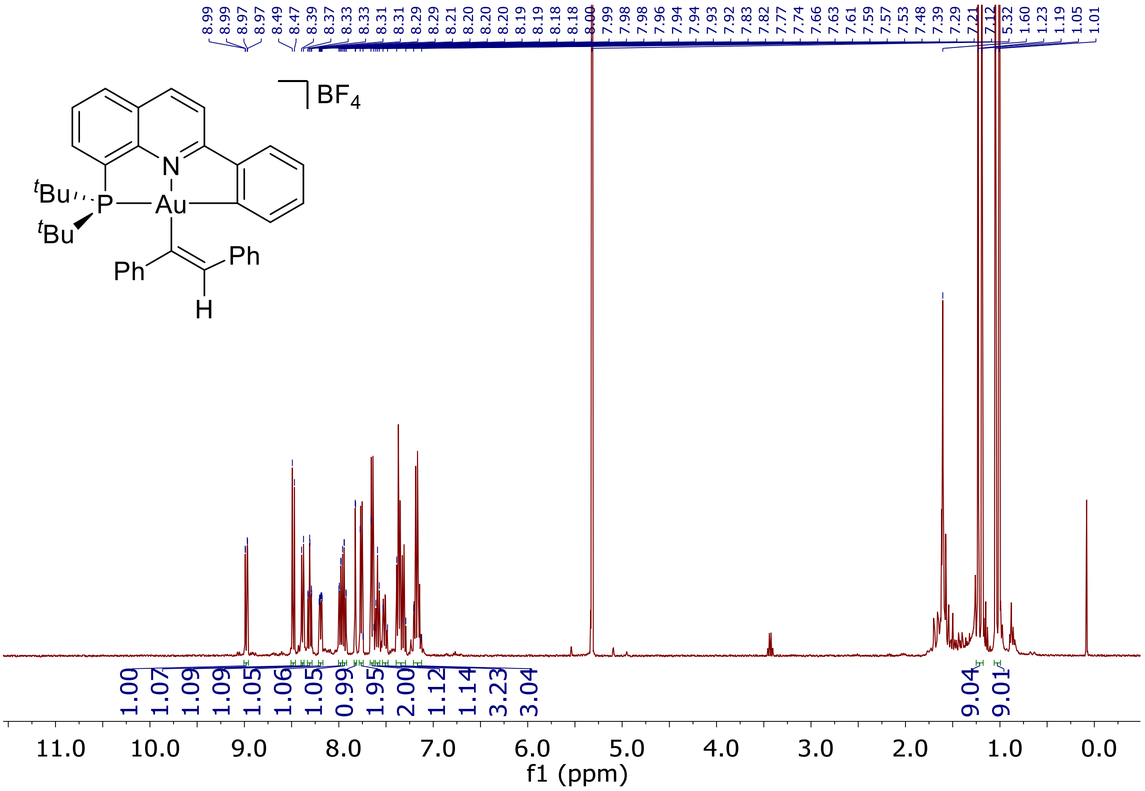

Figure S64. ^{1}H NMR (400.13 MHz, CD_2Cl_2, 298 K) spectrum of compound **10**. The ^{1}H NMR spectrum contains residual diethyl ether (3.42) and water (1.60).

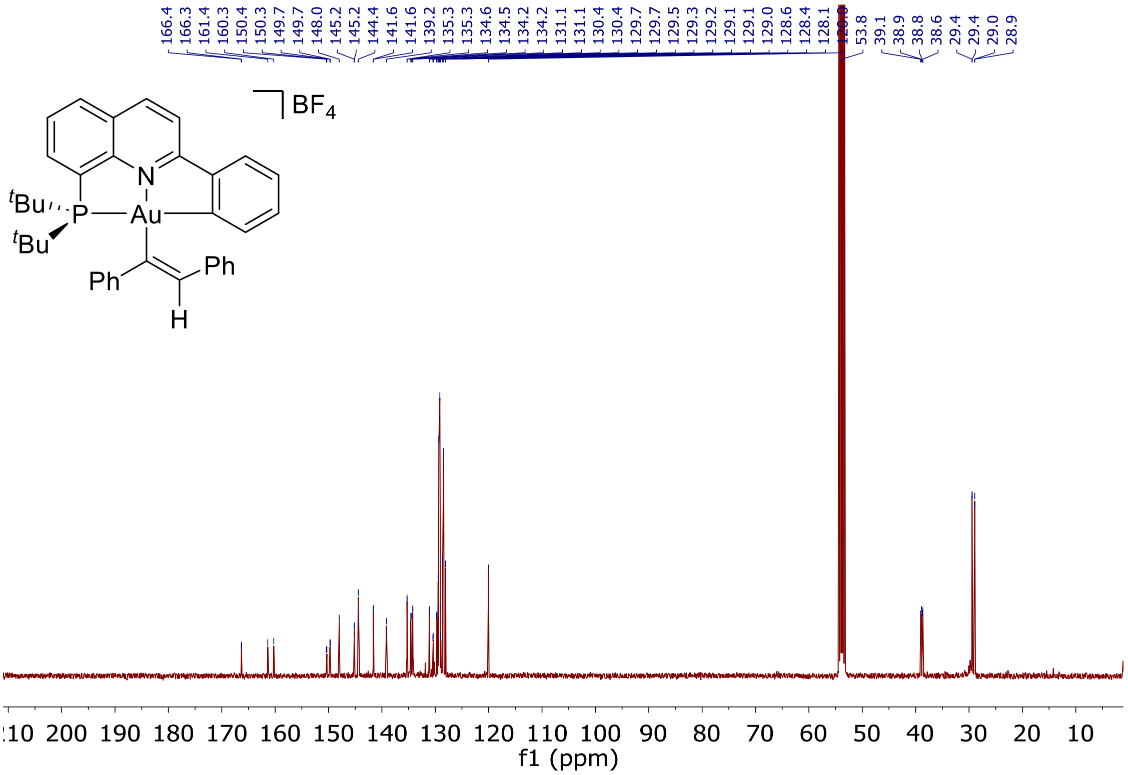

Figure S65. $^{13}C\{^{1}H\}$ NMR (100.62 MHz, CD_2Cl_2, 298 K) spectrum of compound **10**.

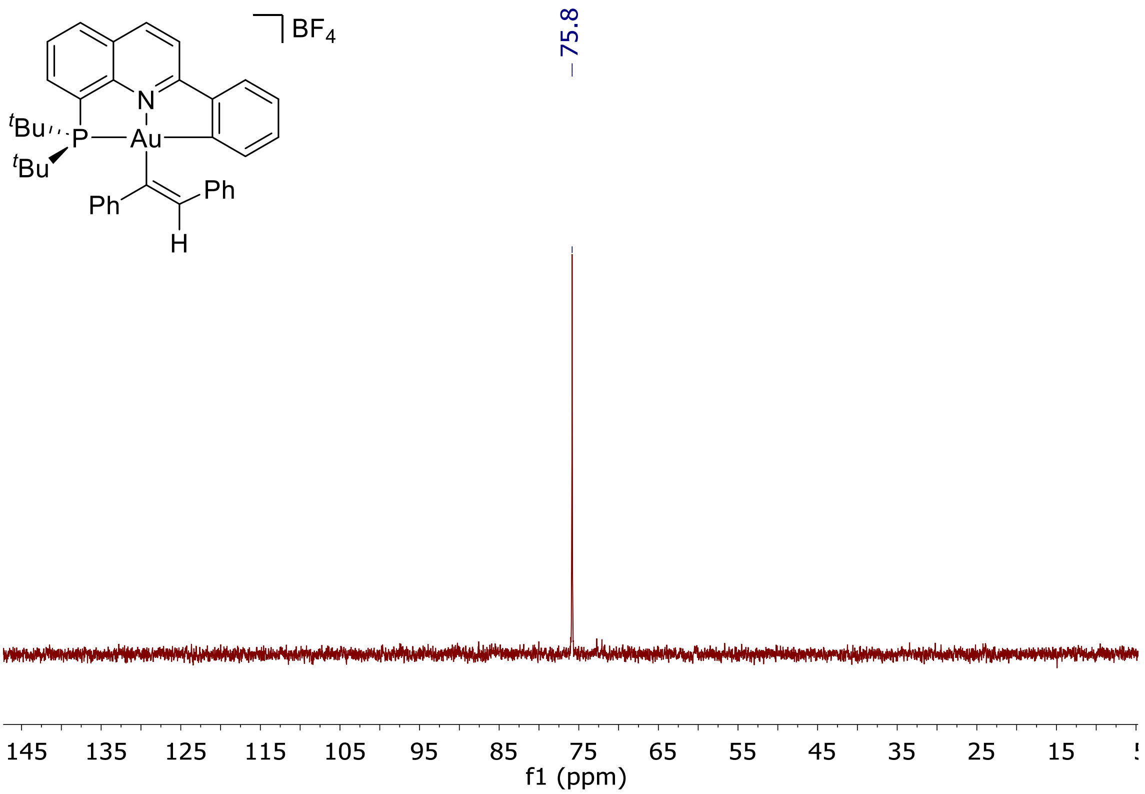

Figure S66. $^{31}P\{^{1}H\}$ NMR (161.99 MHz, CD_2Cl_2, 298 K) spectrum of compound **10**.

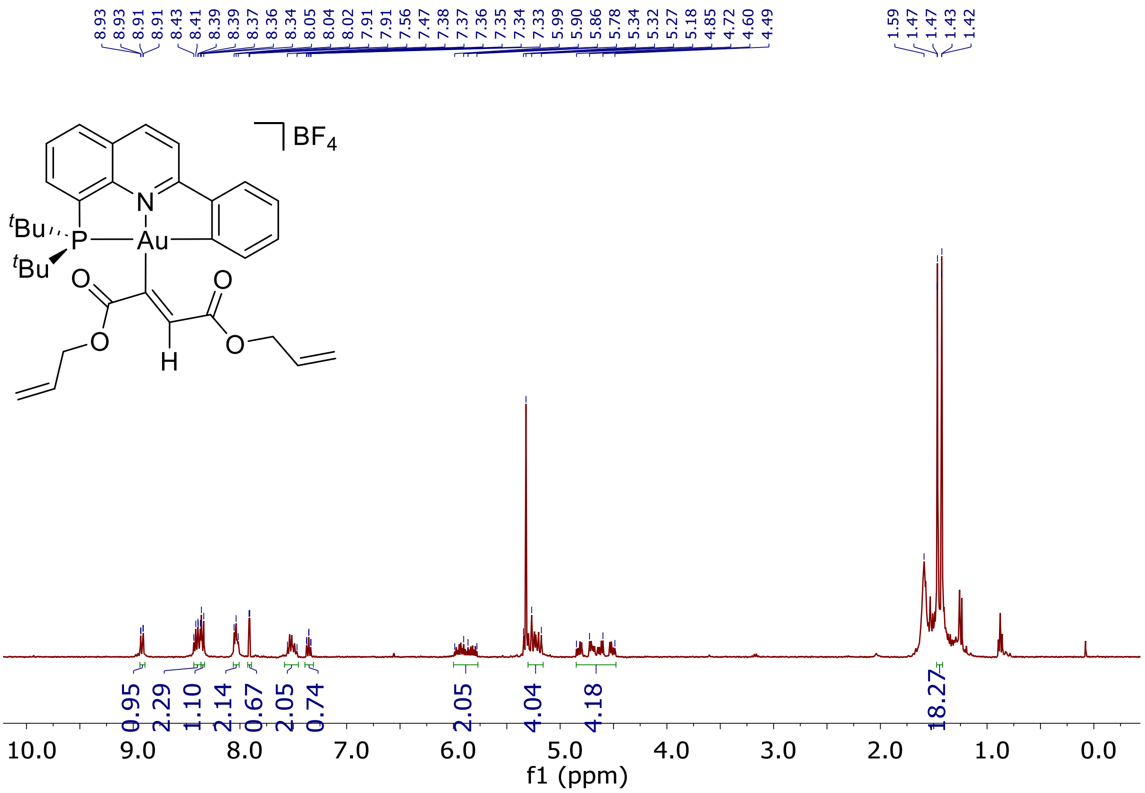

Figure S67. ^{1}H NMR (400.13 MHz, CD_2Cl_2, 298 K) spectrum of compound **11**. The ^{1}H NMR spectrum contains residual pentane (1.23, 0.88) and water (1.59).

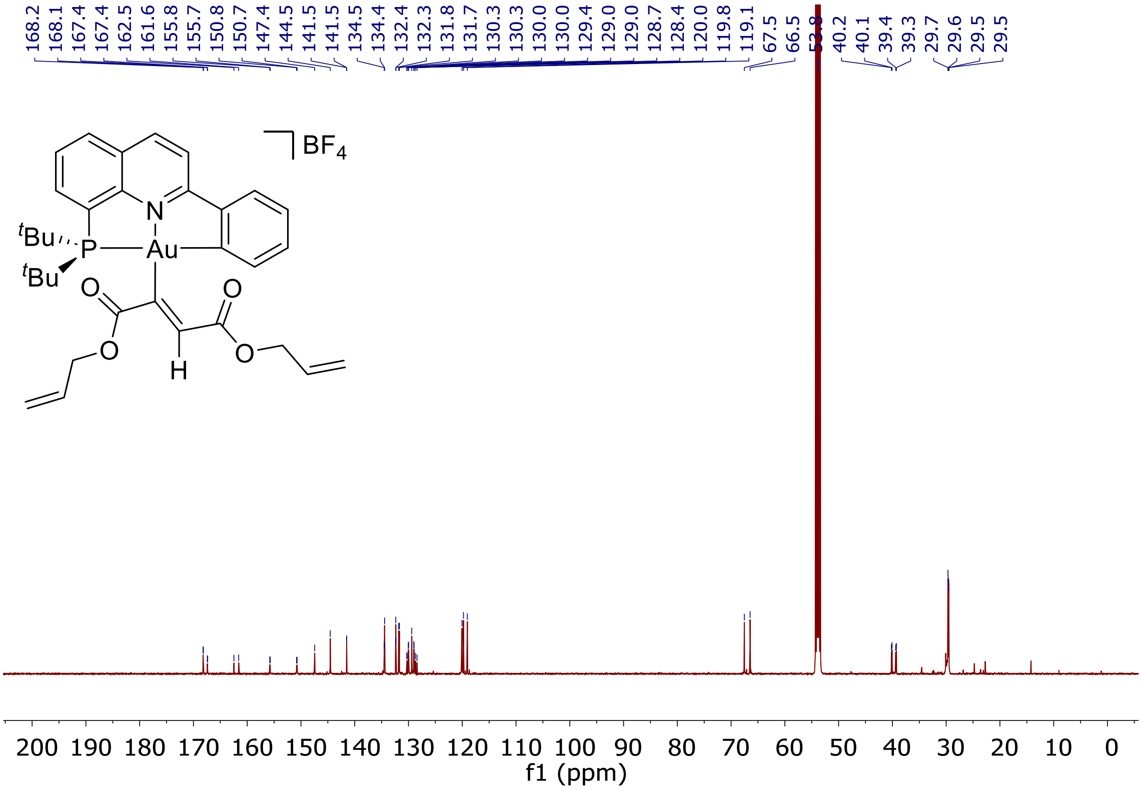

Figure S68. $^{13}C\{^{1}H\}$ NMR (125.81 MHz, CD_2Cl_2, 298 K) spectrum of compound **11**.

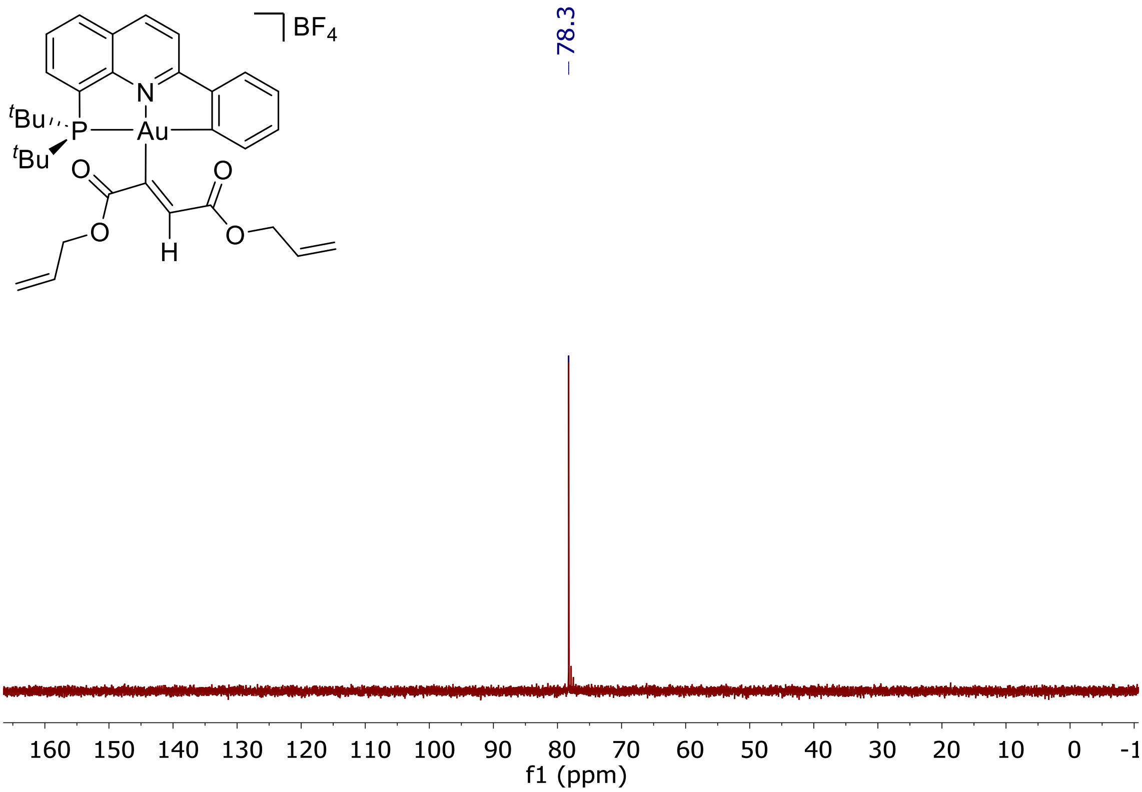

Figure S69. $^{31}P\{^{1}H\}$ NMR (161.99 MHz, CD_2Cl_2, 298 K) spectrum of compound **11**.

5. X-ray diffraction analyses

Complex 2 (CCDC 2374657)

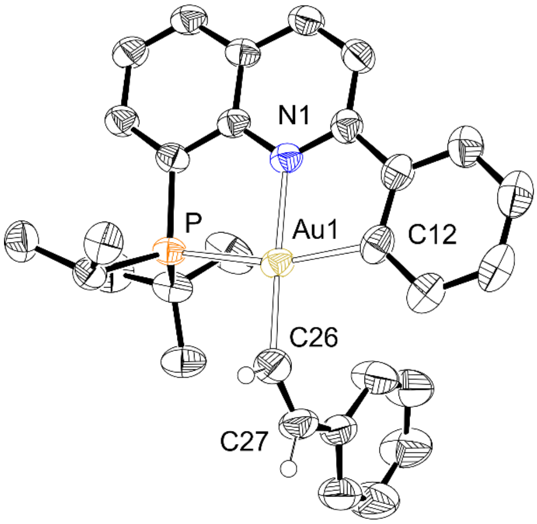

Table S5. Crystal data and structure refinement for complex **2**.

Identification code	MAR220906_339_DCM_Et2O_HP
Empirical formula	$C_{32}H_{36}AuBCl_2F_4NP$
Formula weight	820.26
Temperature/K	160.15
Crystal system	monoclinic
Space group	$P2_1/c$
a/Å	15.5233(11)
b/Å	13.0641(6)
c/Å	15.9995(6)
α/°	90
β/°	97.876(4)
γ/°	90
Volume/Å^3	3214.1(3)
Z	4
ρ_{calc}g/cm^3	1.695
μ/mm^{-1}	4.840
F(000)	1616.0

Crystal size/mm^3	0.46 × 0.15 × 0.09
Radiation	MoKα (λ = 0.71073)
2Θ range for data collection/°	4.092 to 67.838
Index ranges	-24 ≤ h ≤ 22, -20 ≤ k ≤ 19, -23 ≤ l ≤ 25
Reflections collected	85415
Independent reflections	11759 [R_{int} = 0.0542, R_{sigma} = 0.0390]
Data/restraints/parameters	11759/0/385
Goodness-of-fit on F^2	1.021
Final R indexes [I>=2σ (I)]	R_1 = 0.0476, wR_2 = 0.1090
Final R indexes [all data]	R_1 = 0.0731, wR_2 = 0.1183
Largest diff. peak/hole / e $Å^{-3}$	2.87/-0.91

Complex 9 (CCDC 2310071)

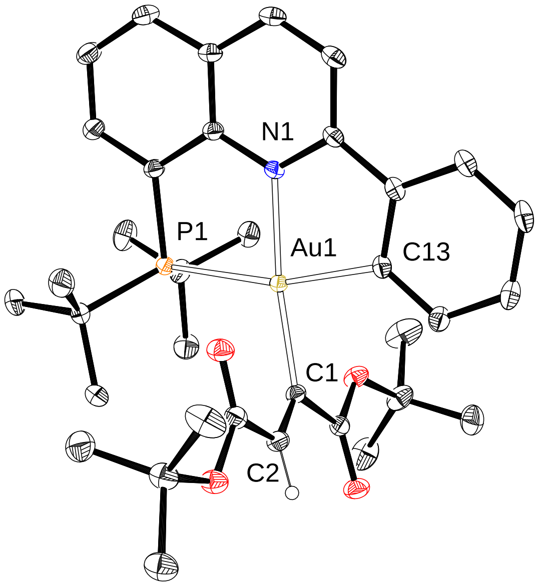

Table S6. Crystal data and structure refinement for complex **9**.

Identification code	Jaime12-10-22
Empirical formula	$C_{36}H_{48}AuBCl_2F_4NO_4P$
Formula weight	944.40
Temperature/K	160.0(1)
Crystal system	monoclinic
Space group	$P2_1/n$
a/Å	14.4022(2)
b/Å	18.8357(2)
c/Å	15.3468(2)
α/°	90
β/°	109.1000(10)
γ/°	90
Volume/Å^3	3934.02(9)
Z	4
ρ_{calc}g/cm^3	1.595
μ/mm^{-1}	3.973
F(000)	1888.0
Crystal size/mm^3	0.14 × 0.11 × 0.05

Radiation	Mo Kα (λ = 0.71073)
2Θ range for data collection/°	3.692 to 61.012
Index ranges	-20 ≤ h ≤ 20, -26 ≤ k ≤ 26, -21 ≤ l ≤ 21
Reflections collected	76466
Independent reflections	11994 [R_{int} = 0.0399, R_{sigma} = 0.0261]
Data/restraints/parameters	11994/196/500
Goodness-of-fit on F^2	1.045
Final R indexes [I>=2σ (I)]	R_1 = 0.0263, wR_2 = 0.0627
Final R indexes [all data]	R_1 = 0.0339, wR_2 = 0.0651
Largest diff. peak/hole / e $Å^{-3}$	1.31/-1.35

6. DFT Calculations

General information

DFT calculations were performed using Gaussian 16.[8] Gas-phase calculations were done using PBE[9] (PBEPBE in Gaussian) in combination with DEF2SVP.[10] Stationary points were confirmed by frequency and IRC calculations. Reoptimization, frequency calculations and NBO analyses were done using the same functional and employing DEF2TZVP[10] as a basis set. Using this basis set in Gaussian 16, for all heavy atoms (from Rb onwards), the effective core potential (ECP) of the DEF2TZVP definition is automatically applied, accounting for relativistic effects implicitly.

Unless stated otherwise, all energies stated in the following correspond to Gibbs free energies as obtained by the frequency calculations of the reoptimized structures.

For determining bond orders and formal oxidation states by LOBA calculations,[11] the MultiWFN package[12] was used.

Pathway investigations

For the DFT study, Au(III) hydride **1** and phenyl acetylene were chosen to investigate the formation of complex **2**. To facilitate the calculations, the BF_4 counterion was omitted in some of the model complexes.

In terms of overall energy balance, we found the final alkene product **2** at an energetic minimum (-16.9 kcal mol^{-1} relative to Au(III)-H complex **1**), rendering the whole alkyne insertion process exergonic. This in line with the reactivity observed and offers an explanation for the overall driving force of the reaction.

Our investigations of the reaction cycle start with T-shaped intermediate **II**, the reactive neutral complex after the proton transfer, to which a molecule of phenyl acetylene approaches to form in the intermediate **III**, which subsequently can react with Au-H molecule **I** via the bimolecular $\mathbf{TS_{III\text{-}IV}}$ to reach the final product and regenerate the reactive complex **II**.

The corresponding energy diagram is depicted in Figure S70. All energies for this pathway are given relative to that starting compound **II**.

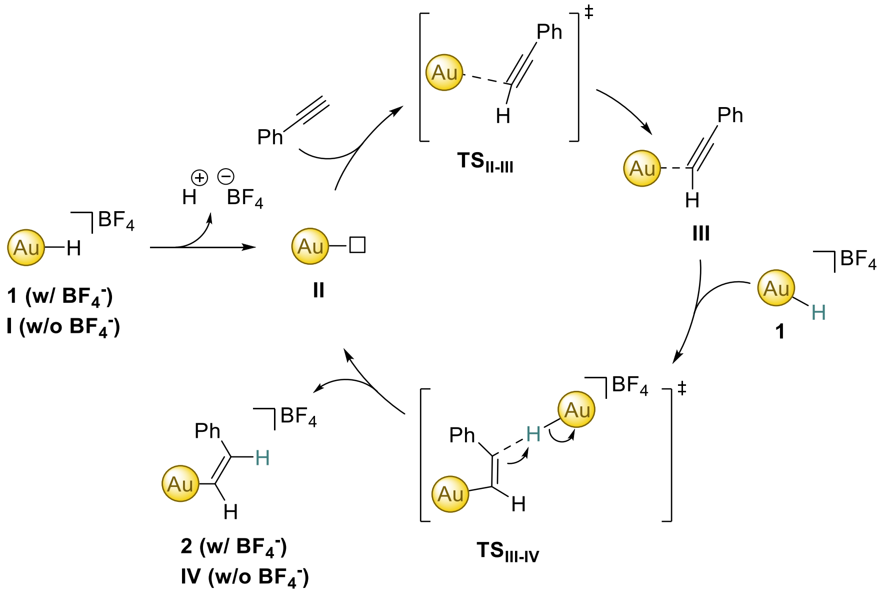

Scheme S12. Proposed pathway assuming deprotonation as initiation.

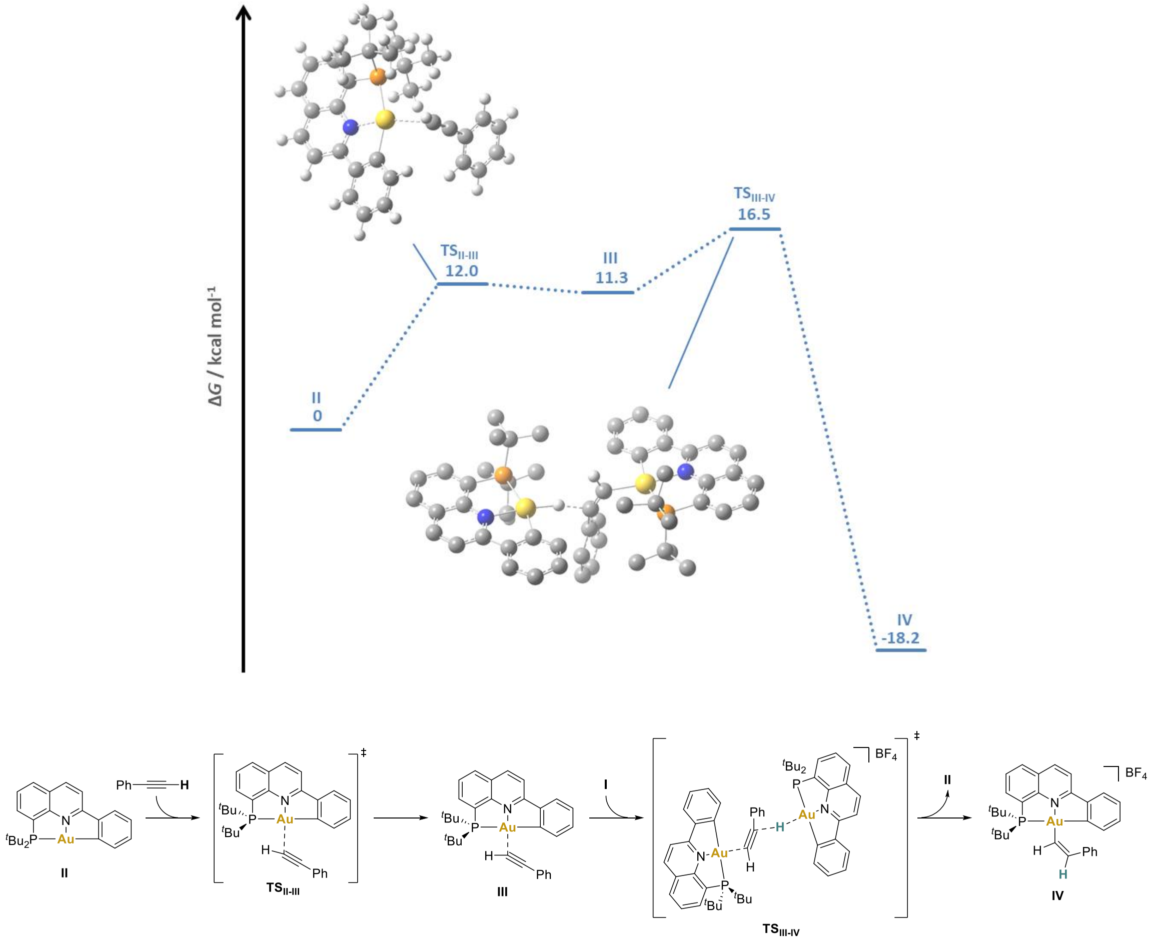

Figure S70. Energy diagram for the pathway initiated by proton transfer.

LOBA calculations for the neutral complex **II** confirmed a formal oxidation state of +I (threshold 33% to 74%). This T-shaped gold(I) species is locked in the same planar geometry by the tridentate ligand as **1** for which the formal oxidation state is +III according to LOBA. We located a TS geometry with a relatively low energetic barrier (**TS**$_{\textbf{II-III}}$, +12.0 kcal mol^{-1}) for the endergonic transition leading to the neutral complex **III** (+11.3 kcal mol^{-1}). While the bond order analysis for the coordinated phenyl acetylene reveals an NBO of 2 between the two carbon atoms, the bond length of 1.263 Å is between a C-C double and triple bond, somewhat closer to a double bond. The angles on the terminal side indicate a somewhat strained double bond character (C-C-H 135.7°, C-C-Au 117.4°), while the angle C-C-Ph still shows propensity to that of an alkyne (168.3°), which is coordinated to Au still at a significantly elevated distance (Au-C 2.237 Å, as compared to 2.026 Å in product **2**). Combined with the relatively high energy and a negative partial charge spread over the alkyne moiety (summed up NBO charges: -0.333), this indicates a reactive, nucleophilic complex **III**. A LOBA evaluation revealed a formal oxidation state of Au in **III** as +I (threshold 35% to 89%), which is in line with the loose coordination of the alkyne.

For the subsequent transition to the final product via the approach of the cationic Au(III)-H complex **I**, we found a feasibly low energetic activation barrier (**TS**$_{\textbf{III-IV}}$, +16.5 kcal mol^{-1}) which involves a formal reoxidation of the gold, leading to the stable anti-Markovnikov product **IV** (-18.2 kcal mol^{-1}) observed as main reaction product in the experimental studies while regenerating Au(I) intermediate **II**. The low calculated energy for the final product explains the overall driving force for the overall process.

Calculations of alternative pathways featuring a hydride or a hydrogen atom transfer

A classical hydride transfer to initiate the process has been explored as outlined in Scheme S13. Upon loss of hydride, a cationic intermediate **hydr-II** would thus coordinate phenyl acetylene. With this intermediate **hydr-III**, another molecule of Au-H **I** would react to result in the formation of the anti-Markovnikov insertion product **IV** and intermediate **hydr-II** to promote further reaction with the alkyne.

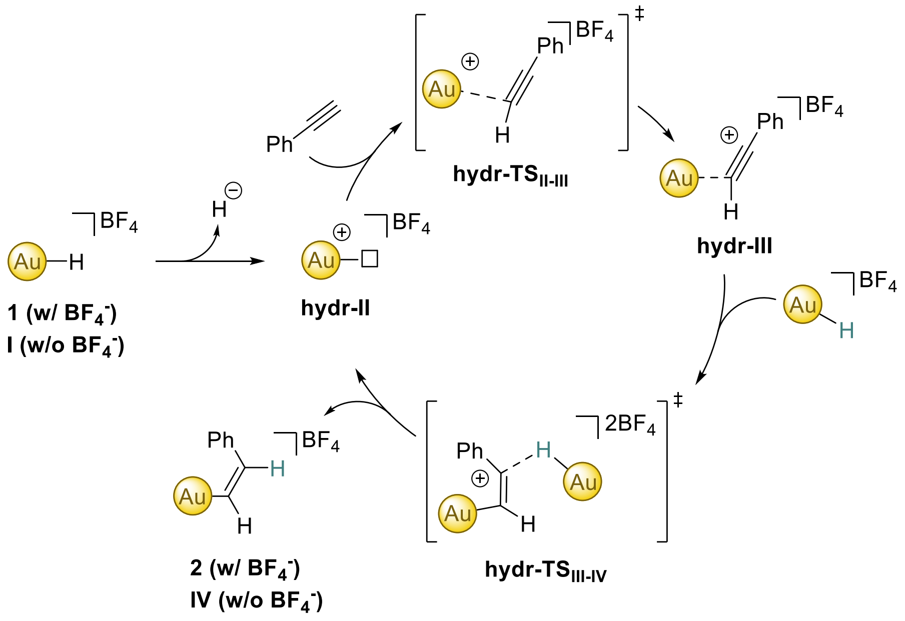

Scheme S13. Proposed reaction pathway assuming a hydride transfer.

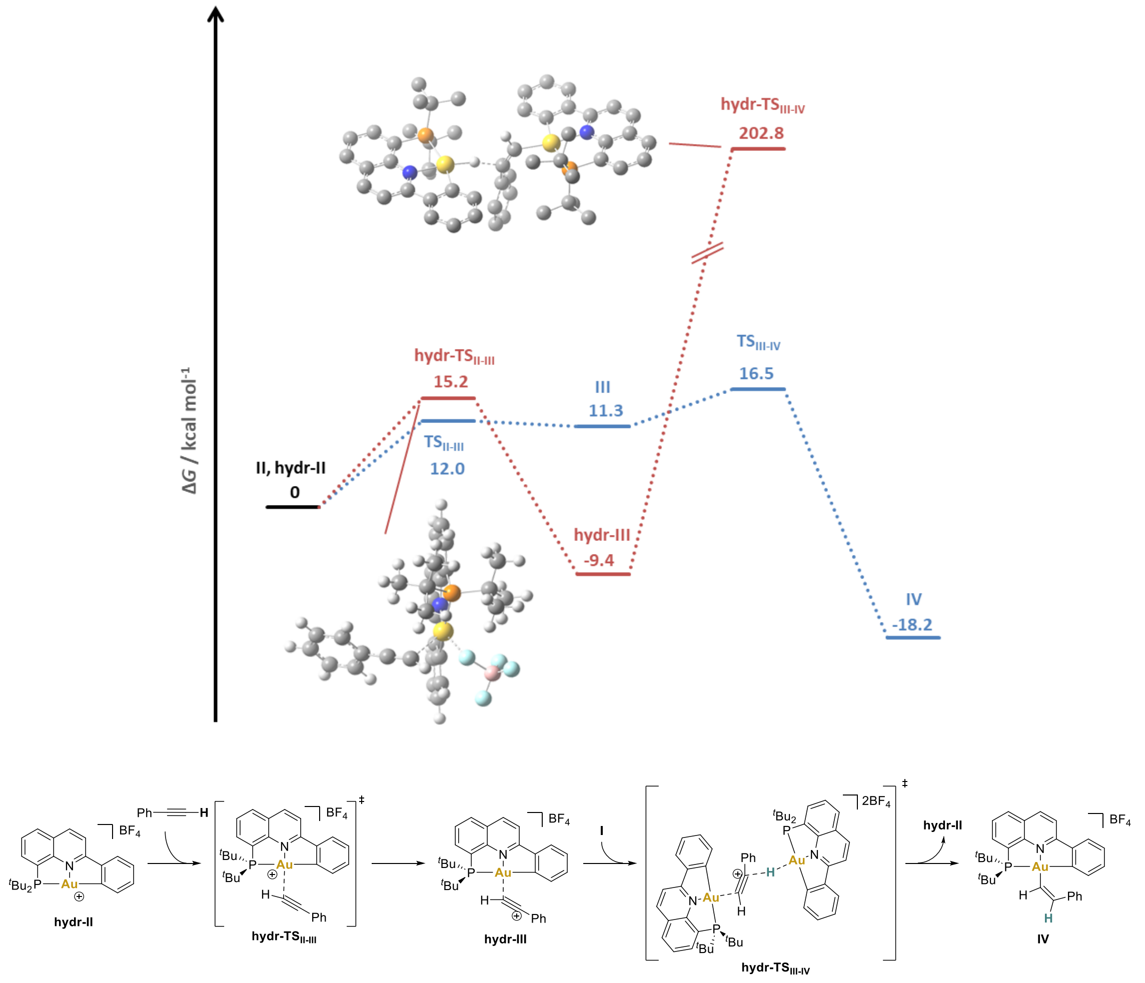

Figure S71. Energy diagram for the pathway initiated by hydride transfer (shown in red) as compared to the proposed and preferred pathway involving proton transfer (in blue).

No feasible transition structure for the initial hydride transfer could be found and thus, the following free energies were calculated relative to compound **hydr-II**. While we were able to locate a TS between **hydr-II** and **hydr-III** at a reasonable barrier (**hydr-TS$_{\text{II-III}}$**, +15.2 kcal mol^{-1}), the attempt to obtain a TS geometry for the hydride donation resulted in a prohibiting high barrier **hydr-TS$_{\text{III-IV}}$** (+202.8 kcal mol^{-1}). As this high barrier is incompatible with the applied reaction conditions (see energy diagram in Figure S71) and the Au(III) complex **1** features a protic behavior, a pathway involving a hydride transfer can be ruled out.

In parallel, a radical pathway was also attempted, however, no feasible structures involving an open-shell approach could be found. Hence, as suggested by experimental results, the DFT investigations propose to rule a radical mechanism for the alkyne insertion reaction.

Investigations for the deprotonation step

Since our initial studies on the Au-H complex **1** revealed a more protic reactivity,[1] we set our focus on exploring the pathway involving a deprotonation as initial step to give **II**. We found an interesting TS geometry (**TS$_{deprot}$**) for an intramolecular deprotonation, where the pyridine moiety of the ligand in complex **1** is decoordinated with the Au-H bond rotating out of the geometry plane towards the nitrogen, as shown in Figure S72. We found the free energy barrier for this transformation (+37.0 kcal mol^{-1}) to be quite high in context of the short reaction times experimentally observed for phenyl acetylene and we could not calculate any further pathway involving the intermediate **Int-PyH**. Nonetheless, these results could suggest a proton transfer as a feasible initiation step for the alkyne insertion.

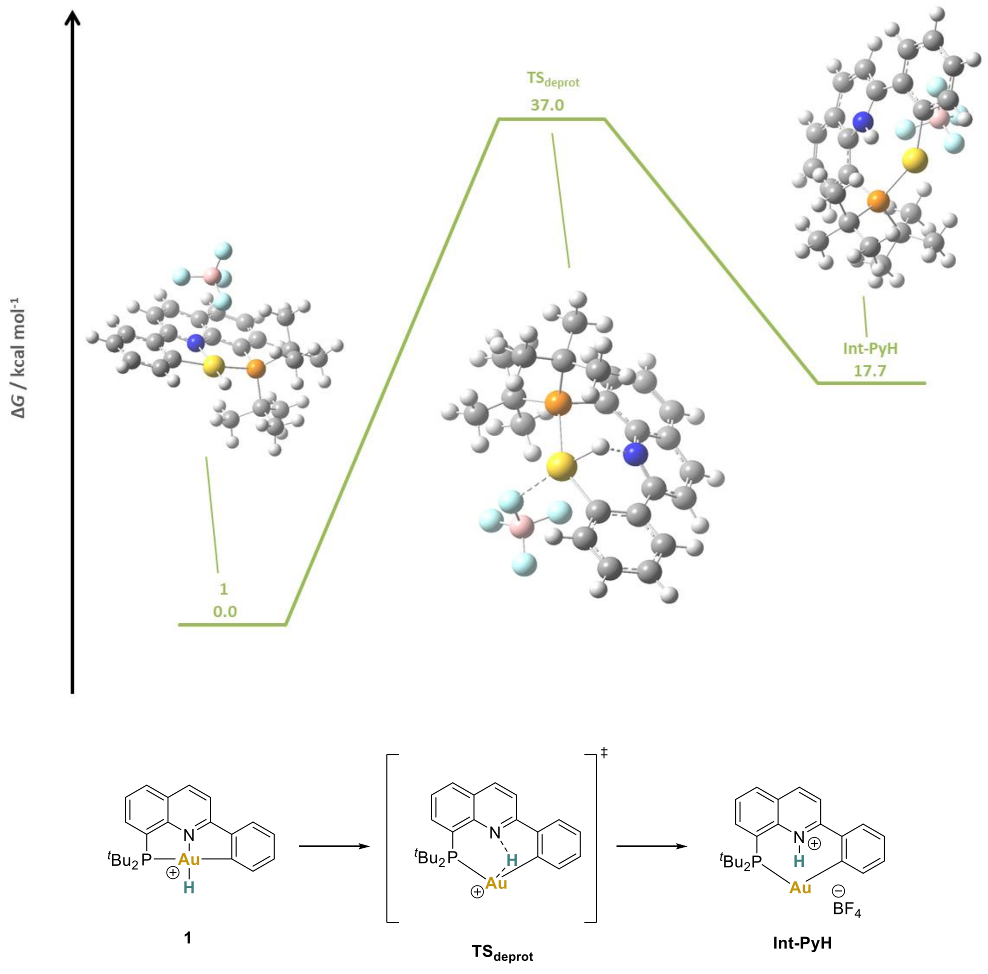

Figure S72. Calculated intramolecular deprotonation.

Several intermolecular options for the deprotonation: residual water, dichloromethane, the BF_4 anion were investigated as potential bases, as well as an intermolecular H-transfer to a second Au-H complex either to the gold center or the nitrogen atom of the pyridine moiety. Also, the involvement of a bimolecular Au complex bridged by one

hydrogen atom was explored in this regard. However, no feasible transition states could be located for these processes.

Given the experimental findings about the beneficial role of added water, we propose that this is needed to serve as a recipient for the proton transfer from the Au-H complex.

Despite having no computational evidence as to the fate of the initial proton transfer, the free energy for this deprotonation step can be derived from the overall reaction energy from **1** to product compound **2** (-16.9 kcal mol^{-1}) as compared to the reaction energy from **II** to compound **IV** (i.e. to the product without counterion; -18.2 kcal mol^{-1}). The approximate energy set-off the pathways described above (free energies always referenced to compound **II**) can thus be estimated at +1.3 kcal mol^{-1}.

TS analysis for the transition from III to IV

The TS for the rate-determining step backs the involvement of two Au-units as suggested by the kinetic experiments. The activation barrier of 16.5 kcal mol^{-1} relative to the neutral complex **II** after proton transfer is in line with our experimental observations.

In the TS geometry (Figure S73), both Au complexes are situated in a plane solely connected by interactions around the alkyne coordinated perpendicularly. In this arrangement, the alkyne is found to be coordinated to the gold center at a distance of 2.076 Å (Au1-C1), whereas the second gold unit is found at a distance of 1.634 Å from the H of the Au2-H bond to the carbon C2 of the alkyne intermediate. The Au2-H bond is elongated to 1.673 Å as compared to the distance of 1.576 Å calculated for complex **1**. The geometry of the bimolecular transition state offers an explanation for the reaction's stereoselectivity for the *Z*-isomer as the steric hindrance posed by the PNC-Au-alkene complex prevents the Au-H approaching from any other reaction trajectory. In contrast, other competent reagents for a proton transfer occupying a smaller volume may be able to approach from above the PNC-Au plane and hence deliver the opposite *E*-isomer of the product complex **IV**.

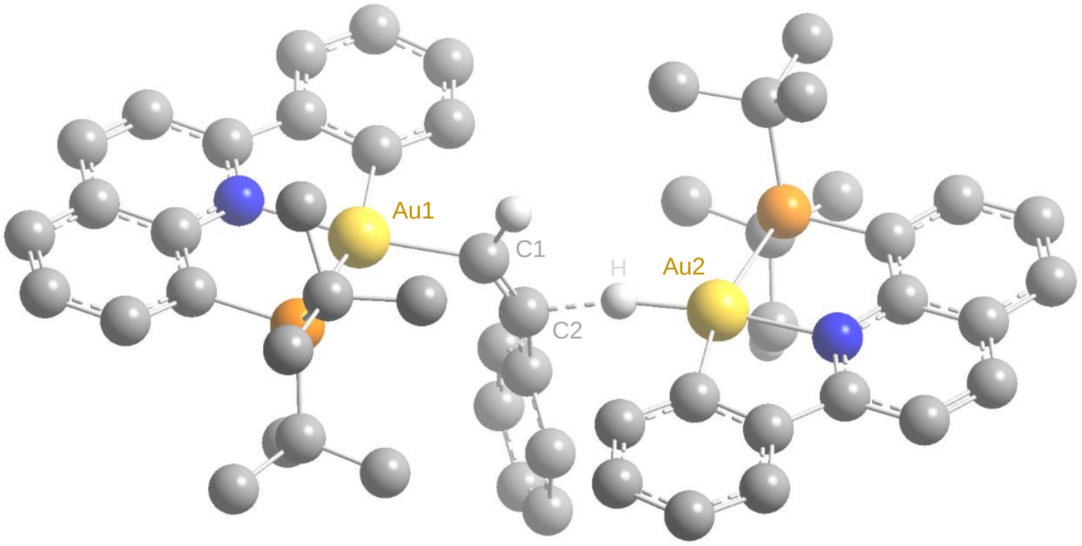

Figure S73. Computed transition state for the rate determining step (TS_{III-IV}), most hydrogens omitted for clarity. Selected distances (Å): Au1-C1 2.076, C1-C2 1.300, C2-H 1.634, Au2-H 1.673.

The analysis of NBO partial charges reveals a significantly positively charged Au center (+0.458) for the Au2-H unit and an almost neutral hydrogen (+0.027), which is in line with our previous results found for Au-H complexes with this PNC ligand class.[1] On the alkyne-Au part, a higher positive partial charge (+0.637) is observed at the metal, which is connected to a strongly negatively charged terminal carbon atom C1 of the alkyne (-0.454). Likewise, C2 of the alkene is found to bear a negative partial charge (-0.145), fostering a proton transfer from the Au-H, as proposed based on experimental findings.

Such an interaction can be seen in the MO analysis as shown in Figure S74, e.g. in the HOMO-1, in which a significant expansion of the bonding MO from the alkene to the Au-H complex can be seen.

a)

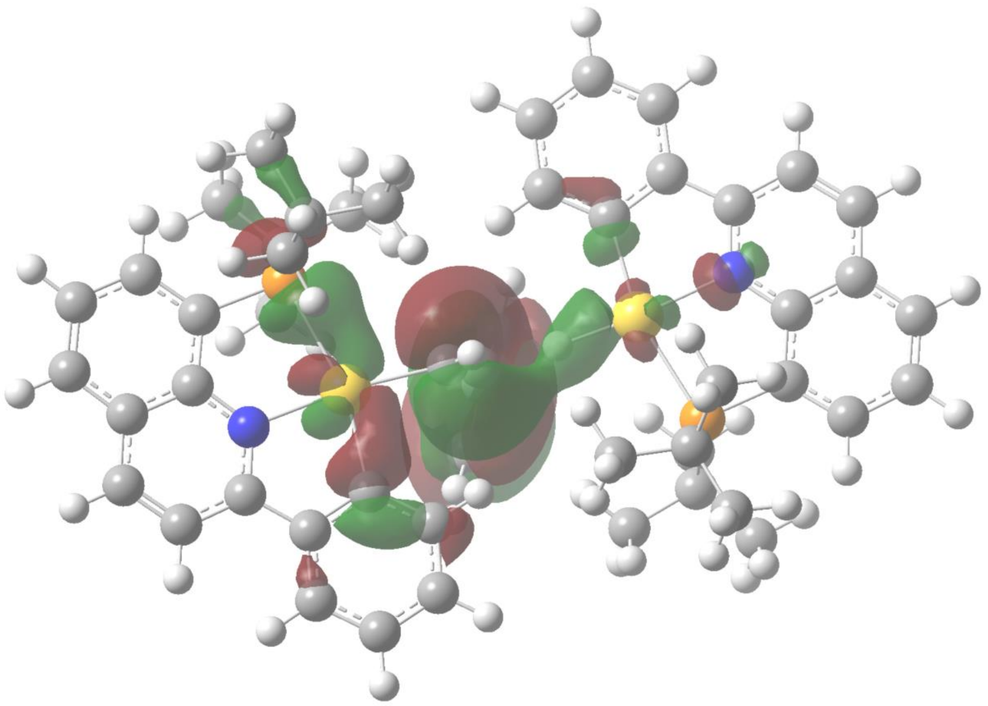

b)

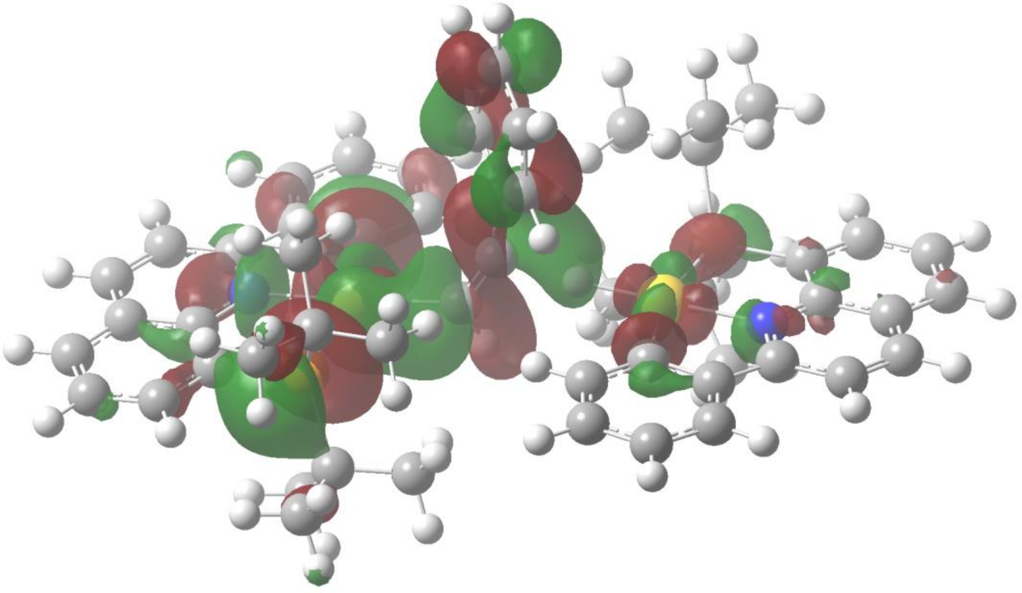

Figure S74. Molecular orbitals, i.e. a) HOMO-1 and b) LUMO+6 for the **TS$_{\mathbf{III\text{-}IV}}$**, showcasing the interaction from the alkene to the hydrogen from the Au-H complex (Isosurface value of 0.02 a.u.).

The transition from **III** to **IV** as well as the formation of complex **II** can be visualised by connecting the minima in the IRC data for the **TS$_{\mathbf{III\text{-}IV}}$**, as presented in Figure S75.

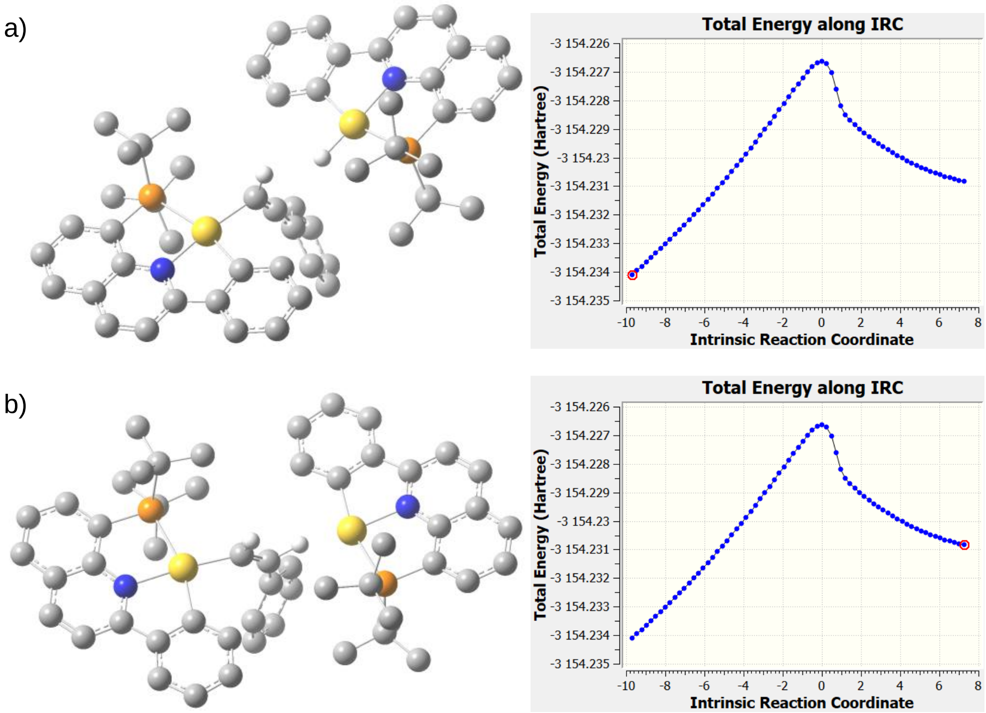

Figure S75. IRC pathway for TSIII-IV including the connected minima at start (a) and end or the reaction path (b). Irrelevant hydrogen atoms omitted for clarity.

Orbital analysis of complex 1

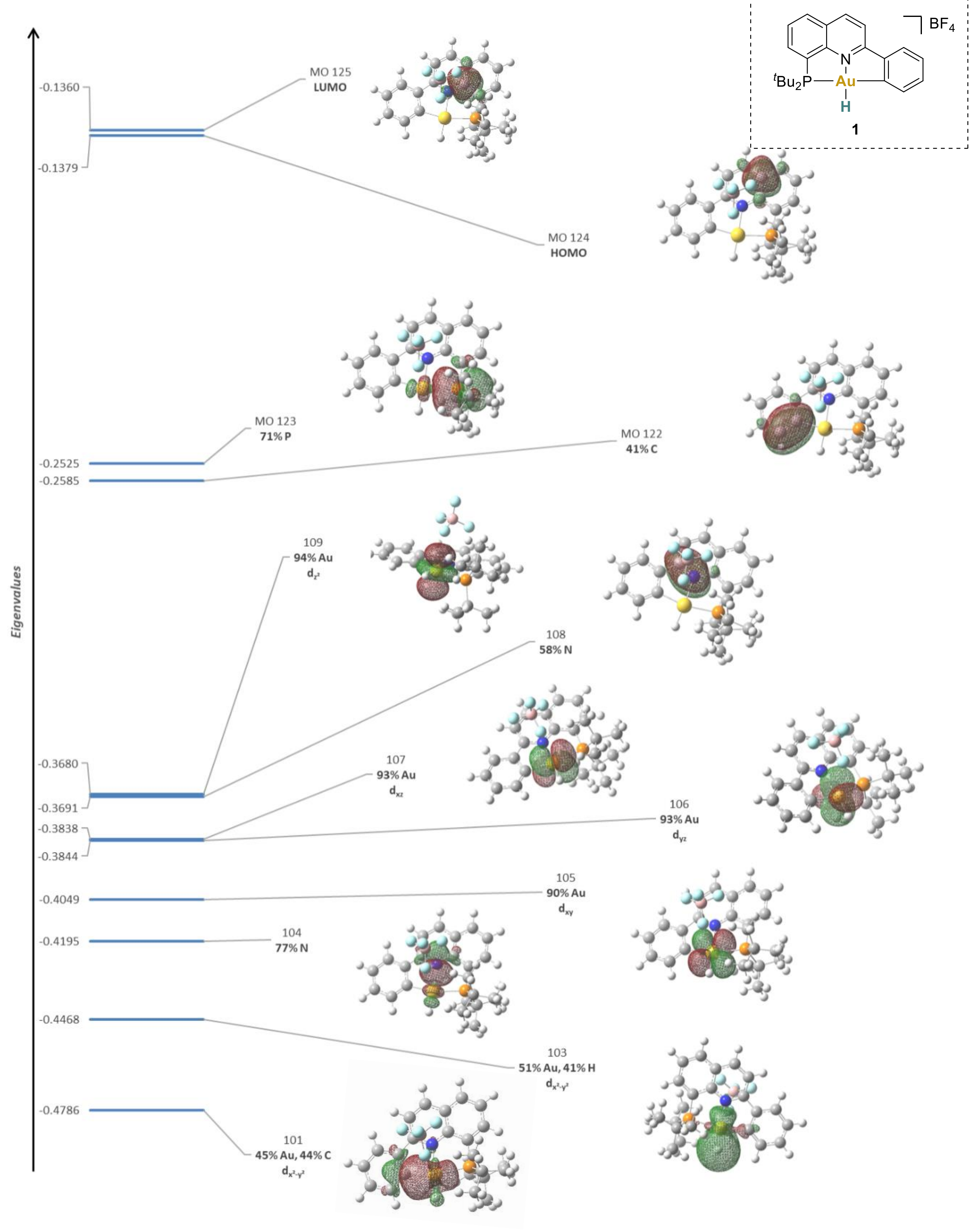

Figure S76. MO analysis of complex AuH **1**.

An analysis of the MOs in compound **1** revealed that the frontier orbitals (MO124(HOMO) and MO125(LUMO)) are not impacted significantly by neither the ligating atoms nor the gold atom center. As can be seen in Figure S76, the MOs with a high contribution of the ligating atoms, especially P and C, *i.e.* MO123 and MO122, were found significantly higher in energy than the MOs stemming from the gold, with the highest being the d_{z^2}

(MO109), and all MOs between them being located at the ligand, albeit not overlapping significantly with the ligating atoms.

Interestingly, the MOs with high N contribution (MO108 and MO104) were found in similar ranges as the MOs at Au.

Concerning the MO at the Au center, we found a reversed order for the MOs as compared to the MOs in a square planar complex, with the d_{z^2} (MO109) being the highest energy in our complex, followed by the d_{xz}, d_{yz} (MO106, MO107) and d_{xy} (MO105) and ultimately the $d_{x^2-y^2}$ being split into two MOs (MO101, MO103), one of them being the bonding orbital of the Au-H bond.

Given the stabilised MOs at the Au center in comparison to the high-energy MOs of the ligand, an inverted ligand-field could be confirmed. The stabilised electrons in the Au-centered MOs are in line with the observed protic character of complex **1**. The strong Au(I)-character of the PNC-Au T-shaped scaffold may offer an explanation for the loose coordination of the alkyne moiety in intermediate **III** and the comparably easy formal reoxidation proceeding via $\mathbf{TS_{III\text{-}IV}}$ of the proposed mechanism.

Exemplary calculation input

An exemplary header for a Gaussian 16 calculation file is depicted in Figure S77. In the example below, the input for a combined optimization and frequency calculation is shown for compound **I**. Since all atoms were calculated using the same basis set, the use of the keywords *gen pseudo=read* or *genecp* can be omitted, ECPs are applied by Gaussian 16 for all heavy atoms (Rb onwards) as defined in the basis set.

The coordinates are truncated in the example below. Full cartesian coordinates can be found below and are separately supplied as xyz-files.

```
%nprocshared=8
%mem=8GB
# opt freq pbepbe geom=connectivity def2tzvp

OF for cmpd I (Cplx 1 w/o BF4)

1 1
 C                  -2.59515200    2.31325600   -0.02766200
 C                  -1.53284600    1.41495600   -0.00188000
 C                  -0.20914300    1.95055000    0.00136200
 C                   0.01052900    3.36252700   -0.00530400
```

Figure S77. Header for a typical calculation file for the opt-freq job for compound **I**.

Coordinates of calculated structures

Au-H complex 1

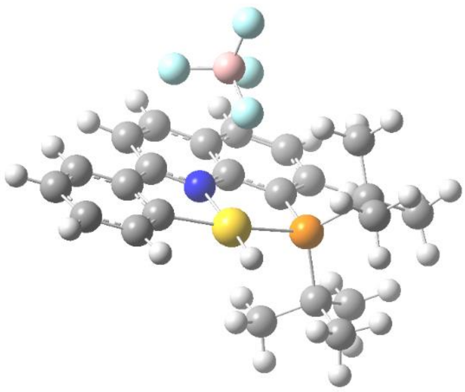

Electronic Energy (EE) = -1848.4064 Hartree
Zero-point Energy Correction = 0.452443 Hartree
Thermal Correction to Energy = 0.485729 Hartree
Thermal Correction to Enthalpy = 0.486673 Hartree
Thermal Correction to Free Energy = 0.387973 Hartree
EE + Zero-point Energy = -1847.9539 Hartree
EE + Thermal Energy Correction = -1847.9207 Hartree
EE + Thermal Enthalpy Correction = -1847.9197 Hartree
EE + Thermal Free Energy Correction = -1848.0184 Hartree

C	2.53655900	2.21627300	-1.00201200
C	1.59912900	1.21671500	-0.76719000
C	0.24466500	1.46435600	-1.14031500
C	-0.12857500	2.69963400	-1.74942800
C	0.86565500	3.67652800	-1.97408000
C	2.17530700	3.43931800	-1.60404100
C	-1.50047400	2.88617500	-2.06381900
C	-2.42575500	1.91131300	-1.78770700
C	-2.00980600	0.69481800	-1.18855300
H	3.57556000	2.07008100	-0.70596700
H	0.57843000	4.62786200	-2.42645500
H	2.93839700	4.20203800	-1.76408500
H	-1.81422100	3.83329200	-2.50756500
H	-3.48135000	2.07456400	-1.99465000
C	-2.87477900	-0.43479600	-0.83731100
C	-4.25568300	-0.44077400	-1.08784000
C	-2.27054300	-1.55745900	-0.20377300
C	-5.03106600	-1.54054600	-0.72828700
H	-4.73426200	0.42183000	-1.55393400
C	-3.05802800	-2.64914400	0.14960100
C	-4.43457600	-2.64234200	-0.11253200
H	-6.10492900	-1.53367400	-0.92019200
H	-2.61028200	-3.51163500	0.64365700
H	-5.04325700	-3.50193000	0.17576400
N	-0.70879700	0.52045600	-0.90816400
P	1.96974400	-0.39243000	0.05342600
C	2.77828700	-0.05285700	1.73940800

C	3.04753800	-1.37298100	-1.16813400
C	4.29872100	-0.62383300	-1.65098700
H	4.83919200	-1.27270900	-2.35912000
H	4.03869200	0.29791700	-2.18817500
H	4.99191600	-0.37537000	-0.83872900
C	2.15099800	-1.65805200	-2.38827300
H	1.26798900	-2.25490000	-2.11924600
H	1.81032300	-0.73212800	-2.87350600
H	2.73470900	-2.22866100	-3.12839400
C	3.44333500	-2.70678900	-0.51302200
H	3.93300700	-3.34070500	-1.26937900
H	4.15408400	-2.57269800	0.31353400
H	2.56644000	-3.25175300	-0.13525400
C	4.29025800	0.20825100	1.67481400
H	4.63687900	0.44336000	2.69381300
H	4.86069500	-0.66481600	1.32966900
H	4.54533600	1.06967000	1.04264800
C	2.06097000	1.16454000	2.34760000
H	2.28738800	2.09521500	1.81209600
H	0.96858500	1.04565000	2.37450200
H	2.41277400	1.27669800	3.38579600
C	2.50225500	-1.28202900	2.62774100
H	2.94082500	-2.20751300	2.23036900
H	2.94870500	-1.09741000	3.61753700
H	1.42460200	-1.43689700	2.76835000
Au	-0.25855800	-1.25596300	0.08822800
H	-0.02100400	-2.63321800	0.81665100
B	-1.69478900	2.04155500	2.03553600
F	-1.90923200	2.61056200	3.28789300
F	-0.73479100	2.79929800	1.29579900
F	-1.16638700	0.70528100	2.19037800
F	-2.89582300	1.96446700	1.28784100

Product complex 2

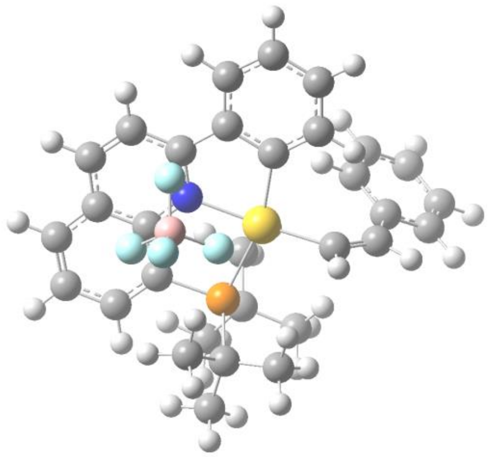

Electronic Energy (EE) = -2156.5586 Hartree
Zero-point Energy Correction = 0.564509 Hartree
Thermal Correction to Energy = 0.605195 Hartree
Thermal Correction to Enthalpy = 0.606139 Hartree
Thermal Correction to Free Energy = 0.489636 Hartree

EE + Zero-point Energy = -2155.9941 Hartree
EE + Thermal Energy Correction = -2155.9534 Hartree
EE + Thermal Enthalpy Correction = -2155.9525 Hartree
EE + Thermal Free Energy Correction = -2156.069 Hartree

Au	-0.39616000	0.36979500	-0.35090100
P	0.17080500	-1.98240200	-0.13651800
N	1.18819500	0.59615800	1.00381300
C	1.48040500	1.86897600	1.31832500
C	2.59609200	2.13991800	2.15085600
H	2.85134700	3.17108300	2.38549100
C	3.36446400	1.10567600	2.61944400
H	4.23567500	1.30452000	3.24699100
C	3.83512600	-1.33996800	2.69480800
H	4.70295400	-1.16230900	3.33316300
C	3.50925600	-2.61828900	2.28668300
H	4.11543400	-3.46857600	2.60161700
C	2.39940800	-2.83397600	1.44480800
H	2.18251000	-3.85250200	1.12408300
C	1.60002000	-1.78339700	1.00770700
C	1.93668500	-0.46281300	1.42350500
C	3.06251600	-0.23656100	2.27228900
C	0.58252900	2.87367700	0.74926800
C	-0.45400900	2.42187500	-0.11024600
C	-1.32052500	3.34280100	-0.68796500
H	-2.11352200	3.00277000	-1.35494300
C	-1.17305800	4.71038900	-0.42257800
H	-1.85571800	5.42566700	-0.88599200
C	-0.15582100	5.16130900	0.42075400
H	-0.03747700	6.22777700	0.61745600
C	0.71884400	4.24805700	1.00283700
H	1.52087700	4.61159700	1.64672300
C	-1.15050000	-2.93722600	0.84451500
C	-2.34577900	-3.20628400	-0.08528500
H	-2.69985200	-2.28915000	-0.57577300
H	-3.17729300	-3.60677400	0.51615100
H	-2.11046300	-3.95001300	-0.85881800
C	-1.59047200	-2.00092000	1.98510200
H	-0.75304100	-1.74084500	2.64886600
H	-2.34903200	-2.52129700	2.59154500
H	-2.04603900	-1.07558300	1.60905100
C	-0.65744700	-4.25041700	1.47219300
H	-0.30734300	-4.98066900	0.73385600
H	-1.50309100	-4.70752900	2.01122600
H	0.13969200	-4.07819300	2.20747400
C	0.79790100	-2.88201200	-1.69850900
C	-0.14213600	-2.50158800	-2.85747000
H	-1.19446100	-2.75003600	-2.66453800
H	0.17714000	-3.05608400	-3.75409800
H	-0.07033200	-1.43155500	-3.08645300
C	2.20297500	-2.34058500	-2.01083600
H	2.23420400	-1.24368800	-2.06285700
H	2.50085600	-2.73204300	-2.99698600
H	2.95148800	-2.66835800	-1.27856100
C	0.84892400	-4.41157500	-1.56585200

H	1.50152200	-4.74767600	-0.74896900
H	1.27210900	-4.81389700	-2.50024600
H	-0.14333800	-4.86409700	-1.43599200
C	-1.87269300	0.33586400	-1.73728100
H	-1.43998500	0.34253400	-2.74379800
C	-3.21591100	0.32218400	-1.64720600
H	-3.75058200	0.26309700	-2.60573800
C	-4.11479200	0.34755600	-0.48507300
C	-5.43688300	-0.11336100	-0.64997700
H	-5.74938400	-0.47940600	-1.63142800
C	-6.34272100	-0.11514600	0.40925400
H	-7.35829800	-0.48442500	0.25370000
C	-5.95504100	0.36383800	1.66341700
H	-6.66399800	0.37335800	2.49314400
C	-4.65578100	0.84819800	1.83970000
H	-4.34982100	1.24813400	2.80869600
C	-3.74729900	0.84061600	0.78174300
H	-2.74769900	1.25326900	0.92708000
B	3.31317100	1.34212300	-1.63229500
F	3.19836500	2.56922400	-0.93517900
F	1.99349600	0.93635200	-2.06616600
F	4.15334200	1.45760600	-2.73612500
F	3.79486400	0.34109800	-0.73374900

Cationic complex I (without BF_4)

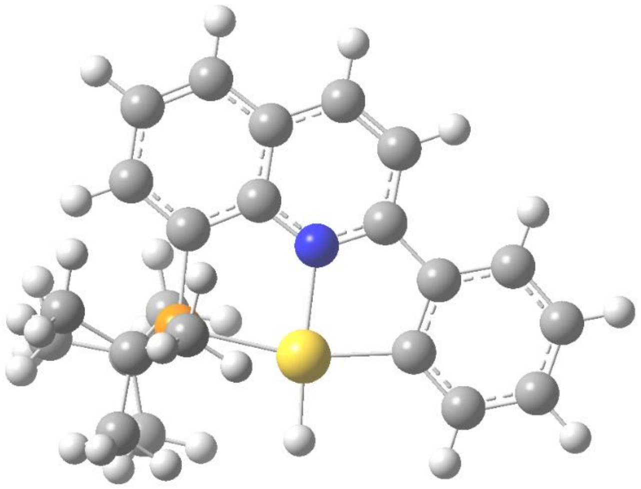

Electronic Energy (EE) = -1423.9913 Hartree
Zero-point Energy Correction = 0.438764 Hartree
Thermal Correction to Energy = 0.465504 Hartree
Thermal Correction to Enthalpy = 0.466448 Hartree
Thermal Correction to Free Energy = 0.384002 Hartree
EE + Zero-point Energy = -1423.5525 Hartree
EE + Thermal Energy Correction = -1423.5258 Hartree
EE + Thermal Enthalpy Correction = -1423.5248 Hartree
EE + Thermal Free Energy Correction = -1423.6073 Hartree

C	-2.59515200	2.31325600	-0.02766200
C	-1.53284600	1.41495600	-0.00188000

C	-0.20914300	1.95055000	0.00136200
C	0.01052900	3.36252700	-0.00530400
C	-1.10741800	4.22807400	-0.02386900
C	-2.38715500	3.70947900	-0.03885200
C	1.35749600	3.81606400	0.00106300
C	2.40501200	2.92546500	0.00728600
C	2.14438100	1.53001800	0.00545200
H	-3.62041700	1.94543300	-0.04114000
H	-0.94316400	5.30731600	-0.02983100
H	-3.24957400	4.37654500	-0.05851900
H	1.55109300	4.89087900	-0.00128600
H	3.43315100	3.28333600	0.01068500
C	3.14648300	0.46208300	0.00244500
C	4.52892400	0.71705500	0.00549300
C	2.67761600	-0.88500900	-0.00476000
C	5.43701800	-0.33905800	0.00150800
H	4.90373700	1.74199400	0.01120400
C	3.59834700	-1.92810400	-0.00847500
C	4.97427900	-1.65637000	-0.00554400
H	6.50804500	-0.13411300	0.00386800
H	3.25840600	-2.96370500	-0.01405000
H	5.68647700	-2.48351100	-0.00897300
N	0.86522200	1.11000300	0.00390500
P	-1.71454300	-0.42710400	0.00357900
C	-2.60136400	-0.92405200	-1.59887100
C	-2.57269200	-0.89646400	1.62740400
C	-3.87465300	-0.12719400	1.89803500
H	-4.30241300	-0.50192300	2.84111700
H	-3.69842000	0.94812300	2.03057900
H	-4.63207700	-0.27179400	1.11782500
C	-1.55708800	-0.56413500	2.73794400
H	-0.63361300	-1.15354800	2.64431900
H	-1.29422500	0.50350900	2.75513800
H	-2.01138900	-0.80784300	3.71085000
C	-2.84468700	-2.41033900	1.61989300
H	-3.16086400	-2.71105500	2.63058000
H	-3.65411000	-2.68485400	0.93047800
H	-1.94899200	-2.99436700	1.36506500
C	-4.10871700	-0.63222600	-1.59666100
H	-4.51507400	-0.90313800	-2.58374100
H	-4.64889200	-1.22855200	-0.84994100
H	-4.33704700	0.42983800	-1.43659500
C	-1.91430600	-0.13370600	-2.72839000
H	-2.10057700	0.94605800	-2.65897700
H	-0.82694200	-0.30218500	-2.74852500
H	-2.31755200	-0.48400500	-3.69090800
C	-2.36381900	-2.42791300	-1.83448200
H	-2.83100300	-3.05880300	-1.06867100
H	-2.81072500	-2.69902100	-2.80337400
H	-1.29373300	-2.67101500	-1.87843700
Au	0.61954200	-0.96426400	-0.00566500
H	0.54139600	-2.53594300	-0.01107700

BF_4 anion

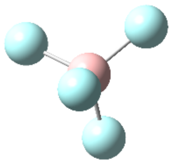

Electronic Energy (EE) = -424.30699 Hartree
Zero-point Energy Correction = 0.013605 Hartree
Thermal Correction to Energy = 0.018093 Hartree
Thermal Correction to Enthalpy = 0.019037 Hartree
Thermal Correction to Free Energy = -0.014111 Hartree
EE + Zero-point Energy = -424.29338 Hartree
EE + Thermal Energy Correction = -424.28889 Hartree
EE + Thermal Enthalpy Correction = -424.28795 Hartree
EE + Thermal Free Energy Correction = -424.3211 Hartree

B	0.00005500	0.00023200	-0.00002500
F	-0.17945900	-0.28528700	1.38094100
F	-1.18753900	0.58327100	-0.52143000
F	1.08372200	0.90683200	-0.15984000
F	0.28324500	-1.20494400	-0.69965700

Phenyl acetylene

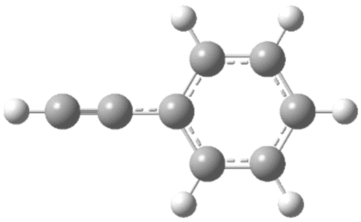

Electronic Energy (EE) = -308.09914 Hartree
Zero-point Energy Correction = 0.106084 Hartree
Thermal Correction to Energy = 0.112752 Hartree
Thermal Correction to Enthalpy = 0.113696 Hartree
Thermal Correction to Free Energy = 0.0755 Hartree
EE + Zero-point Energy = -307.99306 Hartree
EE + Thermal Energy Correction = -307.98639 Hartree
EE + Thermal Enthalpy Correction = -307.98544 Hartree
EE + Thermal Free Energy Correction = -308.02364 Hartree

C	-3.23618800	0.00000200	0.00001000
H	-4.30666600	0.00001000	0.00002900
C	-2.02134600	-0.00000700	-0.00001200
C	-0.59651700	0.00000500	-0.00000600
C	0.11989300	-1.21418300	-0.00000300
C	0.11989100	1.21418100	-0.00000400
C	1.51282700	-1.20899300	0.00000200
H	-0.43162800	-2.15516300	-0.00000700
C	1.51283400	1.20898900	0.00000200

H	-0.43161400	2.15517100	-0.00000700
C	2.21419300	0.00000200	0.00000500
H	2.05544100	-2.15601900	0.00000200
H	2.05543500	2.15602300	0.00000500
H	3.30551800	-0.00000800	0.00001000

II

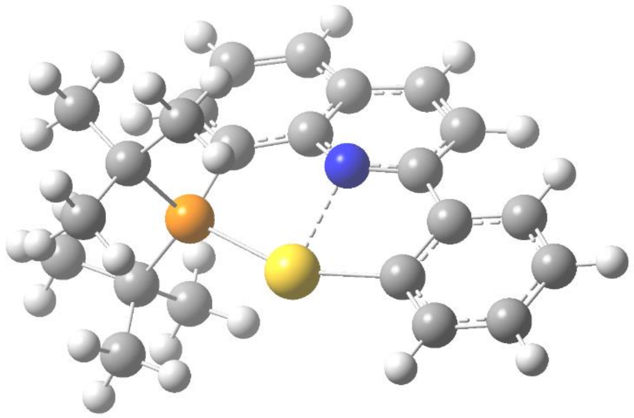

Electronic Energy (EE) = -1423.5704 Hartree
Zero-point Energy Correction = 0.427404 Hartree
Thermal Correction to Energy = 0.45437 Hartree
Thermal Correction to Enthalpy = 0.455314 Hartree
Thermal Correction to Free Energy = 0.371885 Hartree
EE + Zero-point Energy = -1423.143 Hartree
EE + Thermal Energy Correction = -1423.116 Hartree
EE + Thermal Enthalpy Correction = -1423.1151 Hartree
EE + Thermal Free Energy Correction = -1423.1985 Hartree

C	-2.57641500	2.31038300	-0.02565800
C	-1.49998800	1.44138400	0.11217200
C	-0.18928800	2.03284000	0.17098300
C	0.00119000	3.43746900	-0.02494500
C	-1.13498800	4.26874500	-0.15167000
C	-2.39974900	3.71065700	-0.12845800
C	1.35203800	3.88247700	-0.11768400
C	2.39772900	2.98496600	-0.07525600
C	2.12380400	1.60154500	0.15693400
H	-3.59296300	1.92143900	-0.07812600
H	-1.00437900	5.34569800	-0.28522700
H	-3.27994300	4.34858400	-0.22569200
H	1.54638300	4.94681600	-0.27230600
H	3.42042400	3.33129500	-0.22866600
C	3.11705200	0.49742300	0.11471500
C	4.48874300	0.78303200	0.24473400
C	2.67297600	-0.85291200	-0.10581600
C	5.44359200	-0.22756100	0.16266000
H	4.81650000	1.80765300	0.43720000
C	3.66727800	-1.84034100	-0.17989600
C	5.02955600	-1.54334300	-0.05475100
H	6.50334500	0.00974600	0.27304800

H	3.37227200	-2.87746300	-0.35044800
H	5.77066400	-2.34325200	-0.12907400
N	0.86819000	1.22560400	0.35872800
P	-1.63253100	-0.43024500	0.05056400
C	-2.74086800	-0.84130200	-1.45389200
C	-2.42201400	-0.89012800	1.72167200
C	-3.67795900	-0.09095900	2.09428400
H	-4.02708300	-0.42475900	3.08552300
H	-3.46882800	0.98466900	2.16718000
H	-4.50524100	-0.23990900	1.38882400
C	-1.32718300	-0.60384200	2.76788300
H	-0.41566000	-1.18581500	2.57008300
H	-1.05139500	0.46010000	2.79223600
H	-1.70850900	-0.87850900	3.76508600
C	-2.72990800	-2.39602700	1.72306900
H	-2.99182400	-2.70703500	2.74748800
H	-3.58183100	-2.64764800	1.07590100
H	-1.85924600	-2.98606200	1.40145300
C	-4.25058300	-0.60934600	-1.30629800
H	-4.74296400	-0.87620100	-2.25630800
H	-4.69312600	-1.24196800	-0.52496500
H	-4.50778800	0.43679000	-1.09546700
C	-2.19116700	-0.00503800	-2.62411600
H	-2.39825100	1.06675800	-2.50602600
H	-1.10410800	-0.13764100	-2.73432700
H	-2.66798200	-0.34549000	-3.55755100
C	-2.49380600	-2.32889000	-1.77608400
H	-2.88629800	-2.99812400	-0.99925600
H	-3.00559300	-2.57581300	-2.72081600
H	-1.42036200	-2.53698100	-1.89147100
Au	0.60676200	-1.11531400	-0.15930800

$TS_{II\text{-}III}$

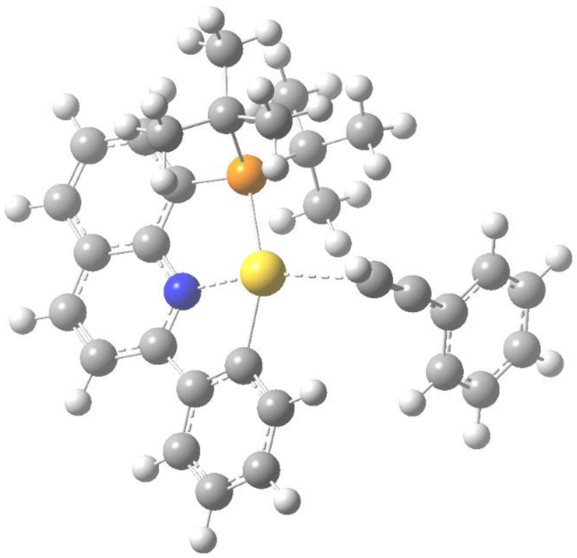

Electronic Energy (EE) = -1731.6682 Hartree
Zero-point Energy Correction = 0.533536 Hartree

Thermal Correction to Energy = 0.568554 Hartree
Thermal Correction to Enthalpy = 0.569498 Hartree
Thermal Correction to Free Energy = 0.465133 Hartree
EE + Zero-point Energy = -1731.1347 Hartree
EE + Thermal Energy Correction = -1731.0997 Hartree
EE + Thermal Enthalpy Correction = -1731.0987 Hartree
EE + Thermal Free Energy Correction = -1731.2031 Hartree

C	-3.72003000	-1.20735600	1.28428800
C	-2.55290700	-0.59351300	0.84262600
C	-2.40144500	0.80677100	1.12992700
C	-3.45891700	1.55455500	1.73887600
C	-4.61774600	0.87160100	2.17225700
C	-4.73064200	-0.49077800	1.96645200
C	-3.26867200	2.96406000	1.82706600
C	-2.13995300	3.55661200	1.30481000
C	-1.10873800	2.73744300	0.75174900
H	-3.89001100	-2.26588600	1.09076800
H	-5.42505800	1.43114100	2.65165300
H	-5.62401000	-1.02211100	2.29903800
H	-4.05501400	3.57516200	2.27704700
H	-2.04055400	4.64246600	1.31036500
C	0.11291400	3.22877100	0.07243800
C	0.53890100	4.55820400	0.25564700
C	0.82980200	2.35183000	-0.80919600
C	1.65252500	5.05285800	-0.41772300
H	0.00795000	5.21118100	0.95269600
C	1.95166800	2.88536900	-1.45961000
C	2.35765500	4.21314400	-1.28362900
H	1.97325800	6.08460100	-0.26206600
H	2.53183800	2.24531900	-2.12850700
H	3.22877900	4.59330900	-1.82345800
N	-1.25662400	1.41747800	0.77219600
P	-1.27035200	-1.42098800	-0.24165500
C	-2.24452800	-2.17447900	-1.70645200
C	-0.46549900	-2.72655000	0.88675200
C	-1.43700700	-3.61433200	1.67644400
H	-0.84868800	-4.32264400	2.28313200
H	-2.05447500	-3.02786300	2.36960900
H	-2.09846600	-4.20735300	1.03177300
C	0.37803600	-1.91057500	1.88606700
H	1.13616000	-1.29955600	1.37711600
H	-0.24589600	-1.24406000	2.49909800
H	0.89790700	-2.60685300	2.56419000
C	0.47179000	-3.59713500	0.03404200
H	1.09695700	-4.20790400	0.70571000
H	-0.08186000	-4.28863400	-0.61689400
H	1.14155200	-2.98428200	-0.58649800
C	-3.04064300	-3.45653500	-1.42881800
H	-3.55755300	-3.75888400	-2.35488300
H	-2.39285000	-4.29241800	-1.13222000
H	-3.81310000	-3.32193900	-0.66059300
C	-3.19178100	-1.06687000	-2.20266300
H	-4.00887200	-0.86987100	-1.49618300
H	-2.64952400	-0.12498600	-2.37660400

H	-3.63603700	-1.38282000	-3.16050900
C	-1.21527400	-2.45395300	-2.81909400
H	-0.49807000	-3.23840400	-2.54499400
H	-1.75135100	-2.78861400	-3.72236700
H	-0.64783900	-1.54565800	-3.06619700
Au	0.16972200	0.36285200	-0.85282900
C	5.22940400	-2.53624900	1.71680200
C	4.40428700	-2.36848400	0.60843600
C	4.08584500	-1.07253000	0.13745400
C	4.62956900	0.04300200	0.81919600
C	5.45523600	-0.13887500	1.92453100
C	5.76069800	-1.42566000	2.38109400
H	5.46499700	-3.54419300	2.06451400
H	3.99818900	-3.23566500	0.08545100
H	4.38341400	1.04589600	0.46757900
H	5.86316800	0.73367700	2.43867900
H	6.40937500	-1.56205700	3.24793300
C	3.25324400	-0.88501100	-0.98993400
C	2.41149900	-0.63248000	-1.85479200
H	2.05357900	-0.53010800	-2.86171400

III

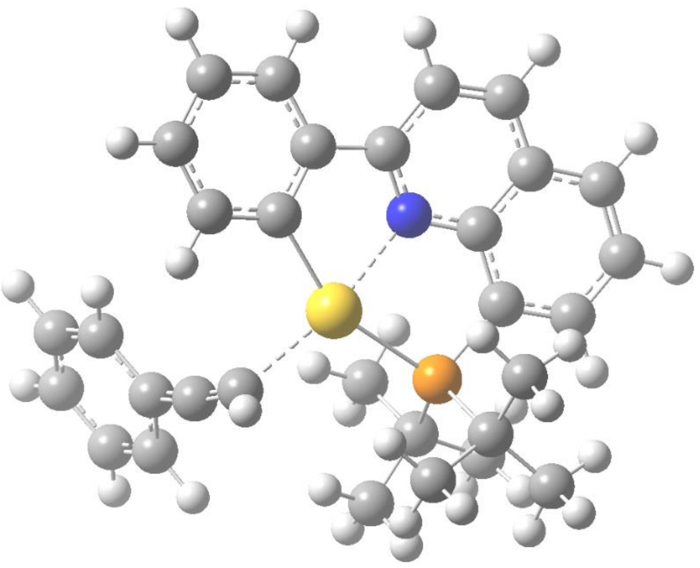

Electronic Energy (EE) = -1731.6697 Hartree
Zero-point Energy Correction = 0.533827 Hartree
Thermal Correction to Energy = 0.569192 Hartree
Thermal Correction to Enthalpy = 0.570136 Hartree
Thermal Correction to Free Energy = 0.465605 Hartree
EE + Zero-point Energy = -1731.1359 Hartree
EE + Thermal Energy Correction = -1731.1006 Hartree
EE + Thermal Enthalpy Correction = -1731.0996 Hartree
EE + Thermal Free Energy Correction = -1731.2041 Hartree

C	3.74499800	-1.10632600	-1.27890500
C	2.56586400	-0.52853200	-0.81540600

C	2.36133500	0.86646500	-1.07812700
C	3.38305200	1.64684000	-1.70846400
C	4.55634500	1.00371800	-2.16059100
C	4.72188000	-0.35599800	-1.96753300
C	3.15307700	3.04964600	-1.80600200
C	2.00832900	3.60791400	-1.28329600
C	1.00597100	2.76927100	-0.71838000
H	3.94801900	-2.15964200	-1.09039300
H	5.33747300	1.59634300	-2.64323400
H	5.62987000	-0.85289200	-2.31295400
H	3.91629800	3.67952100	-2.26880600
H	1.86465700	4.68819200	-1.30820200
C	-0.23669100	3.23736000	-0.08189400
C	-0.69318700	4.55923300	-0.25584500
C	-0.95287500	2.33675800	0.76892100
C	-1.82598800	5.01395700	0.41186300
H	-0.17198800	5.23547200	-0.93759600
C	-2.09953600	2.82116500	1.40860500
C	-2.52603700	4.14461400	1.25480600
H	-2.16921500	6.04020400	0.26870600
H	-2.67687300	2.14936300	2.04840100
H	-3.40950700	4.49931200	1.79154300
N	1.19637200	1.44445300	-0.70252400
P	1.32151900	-1.39829800	0.25985500
C	2.29897600	-1.97728800	1.79629800
C	0.63523100	-2.81970200	-0.80158300
C	1.67345000	-3.69094600	-1.52239400
H	1.13870500	-4.47905200	-2.07803600
H	2.25243200	-3.11429100	-2.25545100
H	2.36917700	-4.19039700	-0.83580200
C	-0.23852800	-2.12072200	-1.86223700
H	-1.05499200	-1.54377900	-1.40692200
H	0.35318300	-1.44485800	-2.49699700
H	-0.68807800	-2.88751700	-2.51371100
C	-0.25848200	-3.69802400	0.08976500
H	-0.83336100	-4.38397900	-0.55346700
H	0.32901800	-4.31604700	0.78340400
H	-0.97496100	-3.09667400	0.66773200
C	3.20842800	-3.19702200	1.59849500
H	3.73659100	-3.40138300	2.54472200
H	2.64151400	-4.10204900	1.34196700
H	3.97629500	-3.03277200	0.83098500
C	3.14085200	-0.76903700	2.24624300
H	3.95956200	-0.54995300	1.54806800
H	2.52228300	0.13534300	2.35150100
H	3.58187700	-0.99256500	3.23109000
C	1.26983200	-2.28221700	2.90119600
H	0.61817500	-3.12959600	2.65294400
H	1.80998900	-2.53417500	3.82840500
H	0.63295400	-1.40945800	3.09965700
Au	-0.25862700	0.35400700	0.73834400
C	-4.97514300	-2.71130500	-1.71446700
C	-4.06407300	-2.48491900	-0.68924800
C	-3.89029300	-1.18358300	-0.14091800
C	-4.68706800	-0.13182300	-0.67573700

C	-5.59511700	-0.37672900	-1.69825400
C	-5.74917100	-1.66407400	-2.22915500
H	-5.08940400	-3.72067300	-2.11618000
H	-3.47677400	-3.31021500	-0.28381600
H	-4.56550700	0.87431400	-0.27185700
H	-6.19138400	0.44920500	-2.09227500
H	-6.46510900	-1.84888900	-3.03136000
C	-2.99545100	-0.93862600	0.90933000
C	-2.05924500	-0.58427800	1.67735700
H	-1.90119100	-0.56441700	2.75192100

TS$_{III-IV}$

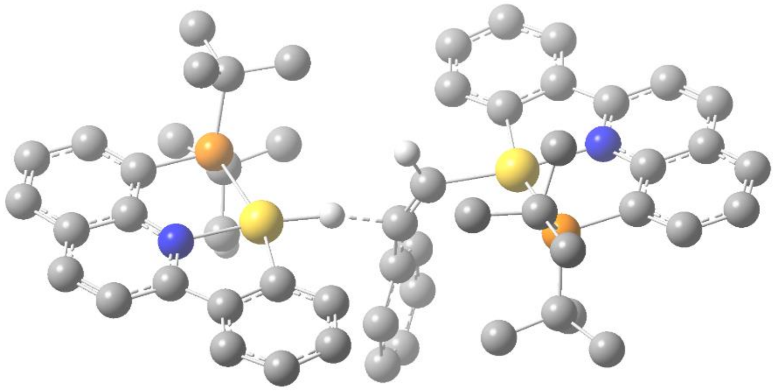

Electronic Energy (EE) = -3155.6786 Hartree
Zero-point Energy Correction = 0.97214 Hartree
Thermal Correction to Energy = 1.033239 Hartree
Thermal Correction to Enthalpy = 1.034183 Hartree
Thermal Correction to Free Energy = 0.875407 Hartree
EE + Zero-point Energy = -3154.7065 Hartree
EE + Thermal Energy Correction = -3154.6454 Hartree
EE + Thermal Enthalpy Correction = -3154.6444 Hartree
EE + Thermal Free Energy Correction = -3154.8032 Hartree

C	0.89052300	-0.19532200	-0.25769400
H	0.49082000	-0.22013500	-1.27344300
C	0.15125200	-0.08766000	0.80653600
C	-0.01635000	-0.15834200	2.21328600
C	-0.45719200	0.95577400	2.97387900
C	0.31861300	-1.37032500	2.87763700
C	-0.52806300	0.86565200	4.35552500
H	-0.75130800	1.87381200	2.46360500
C	0.26216600	-1.43738200	4.26376000
H	0.65576100	-2.22639000	2.29232600
C	-0.16573500	-0.32684300	5.00296600
H	-0.86610600	1.72117500	4.94153300
H	0.54699400	-2.35760200	4.77561800
H	-0.22365700	-0.39029000	6.09103500
C	6.80433900	1.96068800	-0.46462600
C	5.70956300	1.10304200	-0.37621900
C	5.95181800	-0.29661500	-0.36486800
C	7.29162100	-0.79908800	-0.42549800

C	8.36717500	0.11634800	-0.50664400
C	8.12607900	1.47452200	-0.53075000
C	7.47880000	-2.20396800	-0.40745000
C	6.39965200	-3.05273700	-0.34519600
C	5.08731800	-2.52732900	-0.30030400
H	6.65394900	3.03862600	-0.48411900
H	9.38676400	-0.27082600	-0.55326300
H	8.95380800	2.18096200	-0.59942400
H	8.49368300	-2.60486800	-0.44876900
H	6.54549500	-4.13134300	-0.33655600
C	3.86495800	-3.32065300	-0.26565800
C	3.86027600	-4.72626600	-0.27772400
C	2.63208800	-2.61795100	-0.24405200
C	2.65655900	-5.42686000	-0.27826700
H	4.79822100	-5.28299200	-0.29266800
C	1.43754200	-3.32544600	-0.25515300
C	1.44873800	-4.72927400	-0.27292900
H	2.66273500	-6.51707600	-0.29087800
H	0.48056400	-2.80277700	-0.25946900
H	0.50516200	-5.27816500	-0.28629000
N	4.90923100	-1.18618200	-0.30359600
P	3.96590300	1.67206900	-0.29623600
C	3.60656000	2.61325400	-1.91018800
C	3.78905900	2.63653900	1.33085500
C	4.87512600	3.70352900	1.54143600
H	4.64683100	4.23750000	2.47707300
H	5.87210700	3.26255800	1.66264300
H	4.90819800	4.45331000	0.74159100
C	3.90313000	1.58324300	2.44972100
H	3.09026200	0.84263300	2.41129300
H	4.86567000	1.05277200	2.42549200
H	3.84013800	2.09697700	3.42141300
C	2.40102700	3.29277200	1.37446300
H	2.23777600	3.69692900	2.38522500
H	2.31859400	4.13196000	0.67102800
H	1.59735600	2.57210100	1.16761400
C	4.27402400	3.99614200	-1.97080200
H	4.04315100	4.44384700	-2.94995800
H	3.89437200	4.68339600	-1.20370600
H	5.36743000	3.94719000	-1.89747300
C	4.13754900	1.72196000	-3.04881000
H	5.22995200	1.61953800	-3.02897200
H	3.69030300	0.71592700	-3.03724700
H	3.86563800	2.18794800	-4.00811400
C	2.08566600	2.77481100	-2.07250100
H	1.64156200	3.41727400	-1.30285300
H	1.89249100	3.24902000	-3.04659000
H	1.56475800	1.80801100	-2.06857300
Au	2.93614500	-0.55343200	-0.23312900
C	-6.87404500	-1.80830800	-0.62938100
C	-5.75659000	-0.99289500	-0.46381600
C	-5.95340100	0.41537800	-0.44310100
C	-7.26845800	0.96885800	-0.56862200
C	-8.36860300	0.09276800	-0.72257000
C	-8.17266000	-1.27270500	-0.75685900

C	-7.40169400	2.38040000	-0.53587900
C	-6.29795500	3.18843500	-0.39581300
C	-5.00980900	2.61297100	-0.28431800
H	-6.76080800	-2.89069200	-0.66531500
H	-9.37065400	0.51541700	-0.81744800
H	-9.01965100	-1.94775700	-0.88274300
H	-8.39709800	2.82050900	-0.62528200
H	-6.40540500	4.27150500	-0.37110000
C	-3.75627500	3.34795100	-0.14990400
C	-3.68734100	4.75134000	-0.15256000
C	-2.56098100	2.58566500	-0.04362700
C	-2.45565300	5.39568000	-0.05994200
H	-4.59510600	5.35068800	-0.23573500
C	-1.33768100	3.24015600	0.02997400
C	-1.28431400	4.64312500	0.02360500
H	-2.41134300	6.48506900	-0.06211400
H	-0.40878200	2.67176000	0.08154300
H	-0.31912700	5.14966600	0.08504500
N	-4.89058200	1.26796600	-0.31186200
P	-4.03323900	-1.61832500	-0.32287500
C	-3.61351900	-2.44562300	-1.98193100
C	-3.94933200	-2.70777600	1.22748200
C	-5.06441900	-3.76171800	1.31931100
H	-4.88298900	-4.37008700	2.21901800
H	-6.05472500	-3.30562900	1.43933100
H	-5.08557000	-4.44846400	0.46406400
C	-4.08221000	-1.73620000	2.41623800
H	-3.25085200	-1.01644000	2.46058100
H	-5.02947400	-1.17862200	2.39342500
H	-4.07039000	-2.31964800	3.34978200
C	-2.57711300	-3.39870400	1.27254800
H	-2.45945300	-3.87444900	2.25817800
H	-2.48695700	-4.19003000	0.51656900
H	-1.74889000	-2.68634900	1.14526200
C	-4.27697600	-3.81861900	-2.16515600
H	-4.01598300	-4.19740000	-3.16563100
H	-3.92530300	-4.56013900	-1.43612800
H	-5.37251600	-3.76970600	-2.12262100
C	-4.09526400	-1.47915300	-3.08041600
H	-5.18794100	-1.38229700	-3.10530200
H	-3.65577200	-0.47489800	-2.97754500
H	-3.77564300	-1.87699300	-4.05558400
C	-2.08522600	-2.58827500	-2.08620500
H	-1.67452900	-3.28550200	-1.34588900
H	-1.83915500	-2.98315600	-3.08354100
H	-1.57974200	-1.61711800	-1.97810100
Au	-2.95083500	0.54835600	-0.08305800
H	-1.36175400	0.12831300	0.22908500

IV

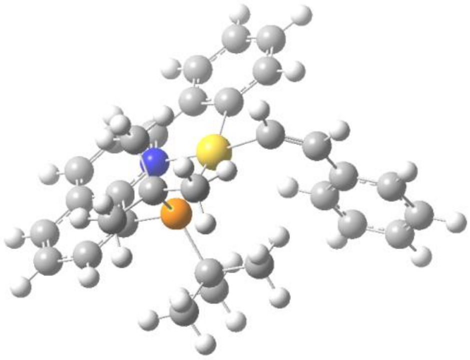

Electronic Energy (EE) = -1732.145 Hartree
Zero-point Energy Correction = 0.550539 Hartree
Thermal Correction to Energy = 0.584771 Hartree
Thermal Correction to Enthalpy = 0.585716 Hartree
Thermal Correction to Free Energy = 0.485031 Hartree
EE + Zero-point Energy = -1731.5944 Hartree
EE + Thermal Energy Correction = -1731.5602 Hartree
EE + Thermal Enthalpy Correction = -1731.5592 Hartree
EE + Thermal Free Energy Correction = -1731.6599 Hartree

Au	0.32008300	0.29512300	0.59212000
P	-1.42537500	-1.34885500	0.21651000
N	-1.06609600	1.59127700	-0.31700100
C	-0.65821800	2.87003000	-0.42634400
C	-1.52621800	3.82284300	-1.02135400
H	-1.20586800	4.85874300	-1.11726200
C	-2.76427200	3.43231100	-1.47074900
H	-3.43723400	4.15872300	-1.93128900
C	-4.45664800	1.62576300	-1.78195900
H	-5.14550400	2.33372900	-2.24680600
C	-4.80755600	0.30037700	-1.62186900
H	-5.78096900	-0.05574100	-1.96066200
C	-3.91299400	-0.60751900	-1.01629000
H	-4.23009600	-1.64311300	-0.90549500
C	-2.65713000	-0.21148400	-0.56523800
C	-2.29006700	1.15688300	-0.73607400
C	-3.19367800	2.08433400	-1.34278600
C	0.67618900	3.14606400	0.10470200
C	1.39705100	2.06210800	0.68061300
C	2.66535400	2.28237500	1.20440800
H	3.22580800	1.45671700	1.64369200
C	3.23101200	3.56444100	1.16468300
H	4.22849100	3.72522000	1.57785500
C	2.53003900	4.63243500	0.60021200
H	2.97530300	5.62748000	0.57146000
C	1.25877000	4.42613600	0.07181800
H	0.72112500	5.26841000	-0.36668800
C	-1.01471600	-2.68612200	-1.07101500
C	0.25349300	-3.43238900	-0.62001100
H	1.09394500	-2.75252200	-0.43555000

H	0.54922900	-4.11910700	-1.42825200
H	0.09145000	-4.03600400	0.28082200
C	-0.70768000	-1.93036900	-2.37747900
H	-1.58909700	-1.41598800	-2.78305300
H	-0.36956200	-2.66182900	-3.12740400
H	0.10345400	-1.19878500	-2.24898600
C	-2.14518500	-3.69794500	-1.30779600
H	-2.36653000	-4.29475600	-0.41353500
H	-1.81638200	-4.39931300	-2.09084800
H	-3.07210200	-3.23189200	-1.66621700
C	-2.20204500	-1.95898700	1.83970000
C	-1.28883700	-3.03769200	2.44665400
H	-1.35711700	-3.98883000	1.90252900
H	-1.61554100	-3.22819100	3.48067200
H	-0.23479400	-2.72786300	2.47946700
C	-2.23864200	-0.72974100	2.76847900
H	-1.23331300	-0.34873300	2.99606100
H	-2.70687700	-1.02493700	3.72037800
H	-2.83734800	0.09119100	2.34763200
C	-3.63090000	-2.50461900	1.69435100
H	-4.34129500	-1.72585500	1.38838300
H	-3.95732100	-2.86908500	2.68114000
H	-3.70336600	-3.34847900	0.99709500
C	1.69947200	-0.79022600	1.60857500
H	1.42648700	-0.86448200	2.66728300
C	2.85115200	-1.36596200	1.21767600
H	3.39480500	-1.89386400	2.01316300
C	3.51921600	-1.44309000	-0.08584000
C	4.64121100	-2.28885400	-0.20227900
H	4.97459600	-2.84887300	0.67482500
C	5.32626400	-2.42236400	-1.40829600
H	6.19033600	-3.08581600	-1.47045800
C	4.91123300	-1.70403400	-2.53189800
H	5.44813100	-1.80141700	-3.47666100
C	3.80793700	-0.85051000	-2.43408700
H	3.48736200	-0.27369200	-3.30372100
C	3.12128700	-0.72253900	-1.22859000
H	2.27719000	-0.03177400	-1.16717200

Structures for the hydridic pathway

hydr-II

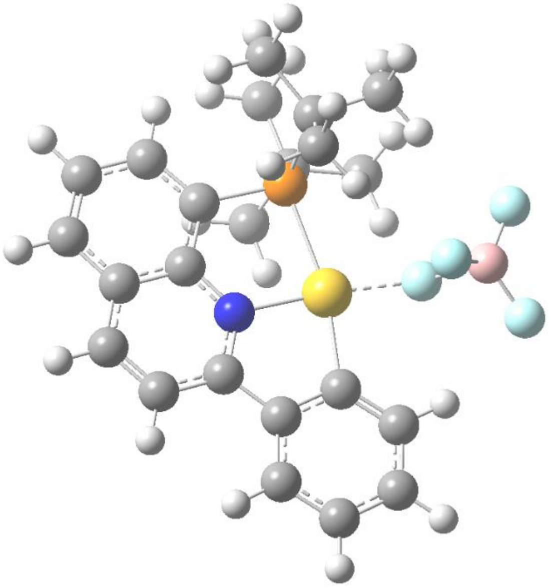

Electronic Energy (EE) = -1847.5823 Hartree
Zero-point Energy Correction = 0.445373 Hartree
Thermal Correction to Energy = 0.47839 Hartree
Thermal Correction to Enthalpy = 0.479334 Hartree
Thermal Correction to Free Energy = 0.381589 Hartree
EE + Zero-point Energy = -1847.1369 Hartree
EE + Thermal Energy Correction = -1847.1039 Hartree
EE + Thermal Enthalpy Correction = -1847.103 Hartree
EE + Thermal Free Energy Correction = -1847.2007 Hartree

C	3.48098000	1.71832900	0.20016600
C	2.18056900	1.22668800	0.11611400
C	1.10837100	2.15755500	0.20478400
C	1.36894300	3.55066900	0.40036600
C	2.70954500	3.99055100	0.48997400
C	3.74929700	3.08946400	0.38394300
C	0.26284300	4.43590800	0.48671900
C	-1.02210500	3.96901500	0.36824000
C	-1.26202700	2.58997900	0.15533200
H	4.32300300	1.03179700	0.12470600
H	2.90414400	5.05427700	0.63840300
H	4.78307400	3.43055900	0.44485900
H	0.45029600	5.50012600	0.64319700
H	-1.87117600	4.64773400	0.42708400
C	-2.56100200	1.97107700	-0.02277900
C	-3.78703200	2.66099300	0.00685700
C	-2.55840500	0.57379200	-0.25158100
C	-4.97404200	1.96114000	-0.19102900
H	-3.81876600	3.73743600	0.18390400
C	-3.73619300	-0.12423700	-0.45078200

C	-4.95073300	0.58165500	-0.41902800
H	-5.92596400	2.49237900	-0.16708900
H	-3.73388000	-1.20185200	-0.61962000
H	-5.88546900	0.03929200	-0.57178800
N	-0.19957100	1.74478000	0.09276000
P	1.75985800	-0.55143300	-0.11100700
C	2.34792100	-1.46464300	1.44377300
C	2.47692700	-1.12527800	-1.76990900
C	3.99702000	-1.33949900	-1.74273300
H	4.31800200	-1.64455300	-2.75115400
H	4.55376300	-0.42627200	-1.49267800
H	4.29488700	-2.13973000	-1.05327200
C	2.11796400	-0.03470100	-2.79607100
H	1.03396700	0.14938800	-2.84494000
H	2.62853700	0.91714000	-2.59742000
H	2.43204000	-0.38231200	-3.79209200
C	1.76947900	-2.43646300	-2.16526600
H	2.11075800	-2.71422000	-3.17443700
H	2.01142100	-3.26907800	-1.49467400
H	0.67684500	-2.33276400	-2.19669700
C	3.80233800	-1.14386600	1.82244900
H	4.06273600	-1.75956800	2.69752200
H	4.52079000	-1.38859200	1.02977600
H	3.93449800	-0.09481400	2.11765100
C	1.41655500	-0.99317000	2.57734100
H	1.45842200	0.09639300	2.72537100
H	0.37379100	-1.29847600	2.41527400
H	1.75272700	-1.46143000	3.51549700
C	2.18233600	-2.97856400	1.22936900
H	2.92101600	-3.37866600	0.52216900
H	2.35452100	-3.47751300	2.19552600
H	1.17583300	-3.26059100	0.89382600
Au	-0.63842700	-0.17981300	-0.22157600
B	-1.87877500	-3.17277400	0.66648900
F	-0.99795100	-4.18949800	0.56049100
F	-1.74388000	-2.35201900	1.74856100
F	-1.21372000	-2.07079500	-0.62150000
F	-3.12537900	-3.36072400	0.19149700

hydr-TS$_{II\text{-}III}$

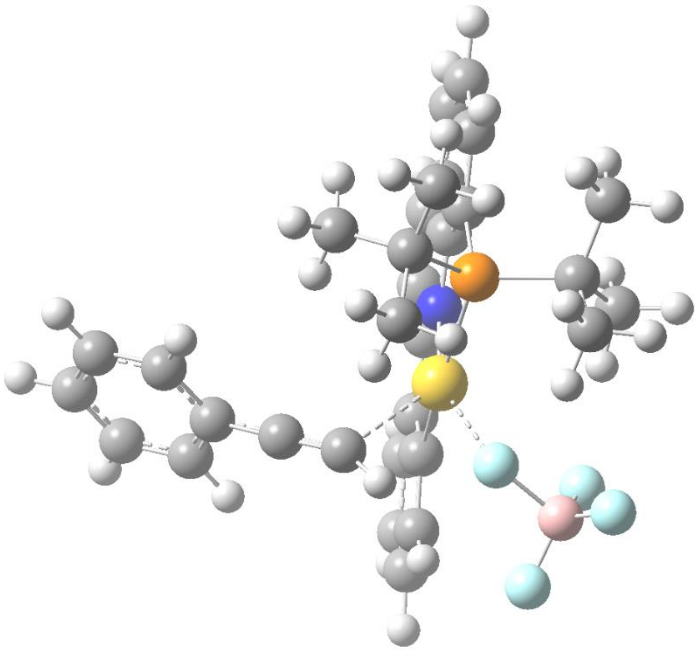

Electronic Energy (EE) = -2155.6782 Hartree
Zero-point Energy Correction = 0.551966 Hartree
Thermal Correction to Energy = 0.592536 Hartree
Thermal Correction to Enthalpy = 0.59348 Hartree
Thermal Correction to Free Energy = 0.478052 Hartree
EE + Zero-point Energy = -2155.1262 Hartree
EE + Thermal Energy Correction = -2155.0856 Hartree
EE + Thermal Enthalpy Correction = -2155.0847 Hartree
EE + Thermal Free Energy Correction = -2155.2001 Hartree

C	2.80765500	-2.84257000	1.13026200
C	1.92114500	-1.79633900	0.89032000
C	0.87410200	-1.58297400	1.83006400
C	0.76079300	-2.40788300	2.99451900
C	1.69293300	-3.45302400	3.18668500
C	2.69590100	-3.67156400	2.26464200
C	-0.29199900	-2.13767000	3.90783200
C	-1.17999900	-1.11861900	3.67311900
C	-1.05877100	-0.32460400	2.50520000
H	3.61987100	-3.03274900	0.43037800
H	1.60221700	-4.08156300	4.07455300
H	3.41302900	-4.47979200	2.41090200
H	-0.38585300	-2.75653200	4.80243900
H	-1.98906200	-0.91043400	4.37127700
C	-1.93123400	0.76915000	2.12763300
C	-2.99189900	1.24992100	2.92022400
C	-1.65439900	1.40428400	0.89255400
C	-3.73830100	2.34114700	2.48944300
H	-3.22070700	0.78433400	3.88049600
C	-2.38706900	2.49834300	0.46662300
C	-3.43286800	2.96754000	1.27624600

H	-4.55238200	2.71995000	3.10826200
H	-2.14158400	3.02197700	-0.45792900
H	-4.00301400	3.84024700	0.95273200
N	-0.04558400	-0.58156100	1.64391100
P	2.04431900	-0.64875700	-0.54883100
C	2.08747200	-1.69326400	-2.13672400
C	3.58806600	0.41439100	-0.22305900
C	4.84123200	-0.42192200	0.08065700
H	5.67543500	0.27986800	0.23702200
H	4.73855900	-1.00670300	1.00443400
H	5.13116000	-1.09132200	-0.73788400
C	3.26022700	1.27139800	1.01435800
H	2.42386000	1.96345800	0.85229500
H	3.05424400	0.65406100	1.90163900
H	4.14482100	1.88533100	1.24458700
C	3.83799300	1.32303300	-1.43914800
H	4.62988000	2.03867600	-1.17007700
H	4.19361600	0.75781600	-2.31140900
H	2.95522000	1.91295300	-1.71661900
C	3.44120200	-2.35868200	-2.42919800
H	3.32958700	-2.96752200	-3.34037600
H	4.23275300	-1.62507500	-2.62659000
H	3.77537300	-3.03529500	-1.63231000
C	0.99672200	-2.76849100	-1.98616400
H	1.24684900	-3.51591300	-1.22167400
H	0.01891000	-2.32956100	-1.73936500
H	0.89325500	-3.29415200	-2.94833000
C	1.72236500	-0.76702100	-3.31264200
H	2.43568600	0.05499400	-3.44565700
H	1.72892300	-1.36628600	-4.23646700
H	0.71778700	-0.34193200	-3.19872300
Au	-0.02330500	0.55833700	-0.05686200
B	0.74185700	3.81001800	-0.61976700
F	-0.36823600	4.49206700	-1.06618100
F	0.59854200	2.36765300	-1.25786200
F	0.73637500	3.57068700	0.75131500
F	1.94114400	4.26871900	-1.10051500
C	-5.34582400	-3.09544500	-1.73743500
C	-5.46807100	-1.85017500	-1.10943500
C	-4.44481800	-0.91545600	-1.20834000
C	-3.27397300	-1.22655800	-1.94221300
C	-3.16101200	-2.48857400	-2.57368500
C	-4.19455500	-3.41136700	-2.46846100
H	-6.15631400	-3.82215300	-1.66255000
H	-6.37152000	-1.60812000	-0.54830900
H	-4.52975100	0.06212000	-0.73238900
H	-2.26608000	-2.71802300	-3.15299300
H	-4.11052400	-4.37982800	-2.96304500
C	-2.25174900	-0.26665100	-2.06695500
C	-1.34656000	0.57774900	-2.09904800
H	-0.86685500	1.42999500	-2.56036000

hydr-III

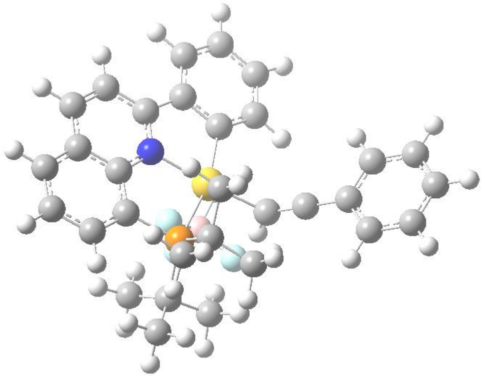

Electronic Energy (EE) = -2155.711 Hartree
Zero-point Energy Correction = 0.552312 Hartree
Thermal Correction to Energy = 0.59458 Hartree
Thermal Correction to Enthalpy = 0.595524 Hartree
Thermal Correction to Free Energy = 0.471738 Hartree
EE + Zero-point Energy = -2155.1587 Hartree
EE + Thermal Energy Correction = -2155.1165 Hartree
EE + Thermal Enthalpy Correction = -2155.1155 Hartree

C	1.88167100	0.21507700	0.76775800
H	1.75201200	0.03951200	1.90536500
C	2.94541000	0.58318600	0.18079200
C	4.14572900	0.94698700	-0.43619000
C	5.01848900	-0.06588400	-0.93957800
C	4.51904200	2.32069200	-0.54971100
C	6.22010600	0.29363200	-1.54472400
H	4.72426600	-1.11485700	-0.84620200
C	5.73007800	2.66170000	-1.14659700
H	3.85272300	3.08943100	-0.14898700
C	6.57591500	1.65213300	-1.64488800
H	6.88997300	-0.47801800	-1.93433300
H	6.02563800	3.71160000	-1.22650800
H	7.52599200	1.92759100	-2.11352200
C	-3.98963300	1.71706300	-0.66683700
C	-2.79519900	0.99782900	-0.56455900
C	-2.80580500	-0.38073500	-0.93818800
C	-4.01004000	-0.99398200	-1.42428100
C	-5.19375300	-0.21639600	-1.50990500
C	-5.18452900	1.11749800	-1.13242100
C	-3.95566700	-2.36887500	-1.79243800
C	-2.78076000	-3.08153400	-1.68242900
C	-1.60629100	-2.44750600	-1.18850200
H	-4.01554800	2.77052200	-0.37820900
H	-6.11122500	-0.68993900	-1.87359400
H	-6.09835000	1.71506700	-1.19161700
H	-4.86445600	-2.85507500	-2.16184100
H	-2.74338100	-4.13699900	-1.95809100
C	-0.31103700	-3.08905500	-0.99887700
C	-0.05761900	-4.44054900	-1.32401900

C	0.72736200	-2.29803600	-0.42779200
C	1.19908800	-4.99648100	-1.07581300
H	-0.84226100	-5.06234400	-1.76612200
C	1.97113700	-2.86680800	-0.16303700
C	2.20649100	-4.21496000	-0.49021000
H	1.38985200	-6.04427000	-1.32414800
H	2.75045700	-2.28899400	0.33845100
H	3.18187600	-4.65930400	-0.26723400
N	-1.66210400	-1.13721100	-0.83781900
P	-1.20124900	1.72128000	0.03466700
C	-1.52204200	2.56764300	1.72669900
C	-0.62176300	2.89262900	-1.36801500
C	-1.70315100	3.89112600	-1.82536000
H	-1.27503800	4.52010800	-2.62717600
H	-2.58255900	3.37976500	-2.24921700
H	-2.03631100	4.56625700	-1.02310400
C	-0.25311100	1.98700100	-2.56457200
H	0.58122000	1.30353600	-2.33093700
H	-1.11065700	1.38347900	-2.90889300
H	0.06101300	2.62698700	-3.40916100
C	0.63535500	3.64674000	-0.88932400
H	1.06087900	4.20351900	-1.74402400
H	0.41106800	4.37926000	-0.09725200
H	1.40885800	2.95401300	-0.51663600
C	-2.12740900	3.97985100	1.59837800
H	-2.31448900	4.35594100	2.62045800
H	-1.44767900	4.69628200	1.10939200
H	-3.09575500	3.99352800	1.07185600
C	-2.47664200	1.65347000	2.52447000
H	-3.48740900	1.61529700	2.08670900
H	-2.07953700	0.63063600	2.62413700
H	-2.56910400	2.07311900	3.54262300
C	-0.16981000	2.64887400	2.46962000
H	0.59905400	3.20314200	1.90605900
H	-0.33615900	3.19369000	3.41666200
H	0.21636600	1.65244400	2.73223600
Au	0.08170700	-0.36566400	-0.03451900
B	0.34270300	-1.41889200	3.28058200
F	1.22173000	-0.21814600	3.33679100
F	-0.75276500	-1.03666300	2.40124300
F	-0.11725900	-1.68960200	4.53548000
F	1.06986300	-2.43399900	2.67481600

hydr-TS$_{III-IV}$

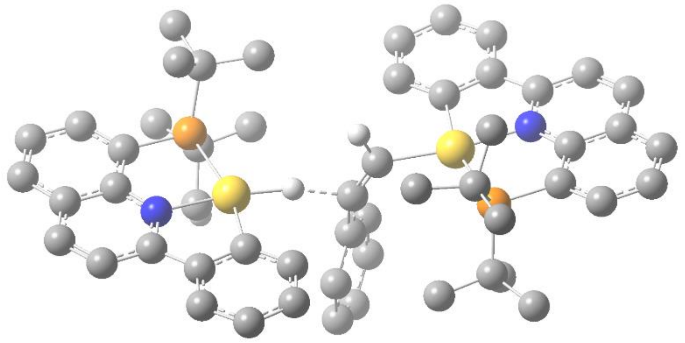

Electronic Energy (EE) = -3155.0643 Hartree
Zero-point Energy Correction = 0.975739 Hartree
Thermal Correction to Energy = 1.038566 Hartree
Thermal Correction to Enthalpy = 1.03951 Hartree
Thermal Correction to Free Energy = 0.876915 Hartree
EE + Zero-point Energy = -3154.0886 Hartree
EE + Thermal Energy Correction = -3154.0257 Hartree
EE + Thermal Enthalpy Correction = -3154.0248 Hartree
EE + Thermal Free Energy Correction = -3154.1874 Hartree

C	0.89052300	-0.19532200	-0.25769400
H	0.49082000	-0.22013500	-1.27344300
C	0.15125200	-0.08766000	0.80653600
C	-0.01635000	-0.15834200	2.21328600
C	-0.45719200	0.95577400	2.97387900
C	0.31861300	-1.37032500	2.87763700
C	-0.52806300	0.86565200	4.35552500
H	-0.75130800	1.87381200	2.46360500
C	0.26216600	-1.43738200	4.26376000
H	0.65576100	-2.22639000	2.29232600
C	-0.16573500	-0.32684300	5.00296600
H	-0.86610600	1.72117500	4.94153300
H	0.54699400	-2.35760200	4.77561800
H	-0.22365700	-0.39029000	6.09103500
C	6.80433900	1.96068800	-0.46462600
C	5.70956300	1.10304200	-0.37621900
C	5.95181800	-0.29661500	-0.36486800
C	7.29162100	-0.79908800	-0.42549800
C	8.36717500	0.11634800	-0.50664400
C	8.12607900	1.47452200	-0.53075000
C	7.47880000	-2.20396800	-0.40745000
C	6.39965200	-3.05273700	-0.34519600
C	5.08731800	-2.52732900	-0.30030400
H	6.65394900	3.03862600	-0.48411900
H	9.38676400	-0.27082600	-0.55326300
H	8.95380800	2.18096200	-0.59942400
H	8.49368300	-2.60486800	-0.44876900
H	6.54549500	-4.13134300	-0.33655600
C	3.86495800	-3.32065300	-0.26565800
C	3.86027600	-4.72626600	-0.27772400
C	2.63208800	-2.61795100	-0.24405200

C	2.65655900	-5.42686000	-0.27826700
H	4.79822100	-5.28299200	-0.29266800
C	1.43754200	-3.32544600	-0.25515300
C	1.44873800	-4.72927400	-0.27292900
H	2.66273500	-6.51707600	-0.29087800
H	0.48056400	-2.80277700	-0.25946900
H	0.50516200	-5.27816500	-0.28629000
N	4.90923100	-1.18618200	-0.30359600
P	3.96590300	1.67206900	-0.29623600
C	3.60656000	2.61325400	-1.91018800
C	3.78905900	2.63653900	1.33085500
C	4.87512600	3.70352900	1.54143600
H	4.64683100	4.23750000	2.47707300
H	5.87210700	3.26255800	1.66264300
H	4.90819800	4.45331000	0.74159100
C	3.90313000	1.58324300	2.44972100
H	3.09026200	0.84263300	2.41129300
H	4.86567000	1.05277200	2.42549200
H	3.84013800	2.09697700	3.42141300
C	2.40102700	3.29277200	1.37446300
H	2.23777600	3.69692900	2.38522500
H	2.31859400	4.13196000	0.67102800
H	1.59735600	2.57210100	1.16761400
C	4.27402400	3.99614200	-1.97080200
H	4.04315100	4.44384700	-2.94995800
H	3.89437200	4.68339600	-1.20370600
H	5.36743000	3.94719000	-1.89747300
C	4.13754900	1.72196000	-3.04881000
H	5.22995200	1.61953800	-3.02897200
H	3.69030300	0.71592700	-3.03724700
H	3.86563800	2.18794800	-4.00811400
C	2.08566600	2.77481100	-2.07250100
H	1.64156200	3.41727400	-1.30285300
H	1.89249100	3.24902000	-3.04659000
H	1.56475800	1.80801100	-2.06857300
Au	2.93614500	-0.55343200	-0.23312900
C	-6.87404500	-1.80830800	-0.62938100
C	-5.75659000	-0.99289500	-0.46381600
C	-5.95340100	0.41537800	-0.44310100
C	-7.26845800	0.96885800	-0.56862200
C	-8.36860300	0.09276800	-0.72257000
C	-8.17266000	-1.27270500	-0.75685900
C	-7.40169400	2.38040000	-0.53587900
C	-6.29795500	3.18843500	-0.39581300
C	-5.00980900	2.61297100	-0.28431800
H	-6.76080800	-2.89069200	-0.66531500
H	-9.37065400	0.51541700	-0.81744800
H	-9.01965100	-1.94775700	-0.88274300
H	-8.39709800	2.82050900	-0.62528200
H	-6.40540500	4.27150500	-0.37110000
C	-3.75627500	3.34795100	-0.14990400
C	-3.68734100	4.75134000	-0.15256000
C	-2.56098100	2.58566500	-0.04362700
C	-2.45565300	5.39568000	-0.05994200
H	-4.59510600	5.35068800	-0.23573500

C	-1.33768100	3.24015600	0.02997400
C	-1.28431400	4.64312500	0.02360500
H	-2.41134300	6.48506900	-0.06211400
H	-0.40878200	2.67176000	0.08154300
H	-0.31912700	5.14966600	0.08504500
N	-4.89058200	1.26796600	-0.31186200
P	-4.03323900	-1.61832500	-0.32287500
C	-3.61351900	-2.44562300	-1.98193100
C	-3.94933200	-2.70777600	1.22748200
C	-5.06441900	-3.76171800	1.31931100
H	-4.88298900	-4.37008700	2.21901800
H	-6.05472500	-3.30562900	1.43933100
H	-5.08557000	-4.44846400	0.46406400
C	-4.08221000	-1.73620000	2.41623800
H	-3.25085200	-1.01644000	2.46058100
H	-5.02947400	-1.17862200	2.39342500
H	-4.07039000	-2.31964800	3.34978200
C	-2.57711300	-3.39870400	1.27254800
H	-2.45945300	-3.87444900	2.25817800
H	-2.48695700	-4.19003000	0.51656900
H	-1.74889000	-2.68634900	1.14526200
C	-4.27697600	-3.81861900	-2.16515600
H	-4.01598300	-4.19740000	-3.16563100
H	-3.92530300	-4.56013900	-1.43612800
H	-5.37251600	-3.76970600	-2.12262100
C	-4.09526400	-1.47915300	-3.08041600
H	-5.18794100	-1.38229700	-3.10530200
H	-3.65577200	-0.47489800	-2.97754500
H	-3.77564300	-1.87699300	-4.05558400
C	-2.08522600	-2.58827500	-2.08620500
H	-1.67452900	-3.28550200	-1.34588900
H	-1.83915500	-2.98315600	-3.08354100
H	-1.57974200	-1.61711800	-1.97810100
Au	-2.95083500	0.54835600	-0.08305800
H	-1.36175400	0.12831300	0.22908500

Structures for the deprotonation pathway

TS$_{deprot}$

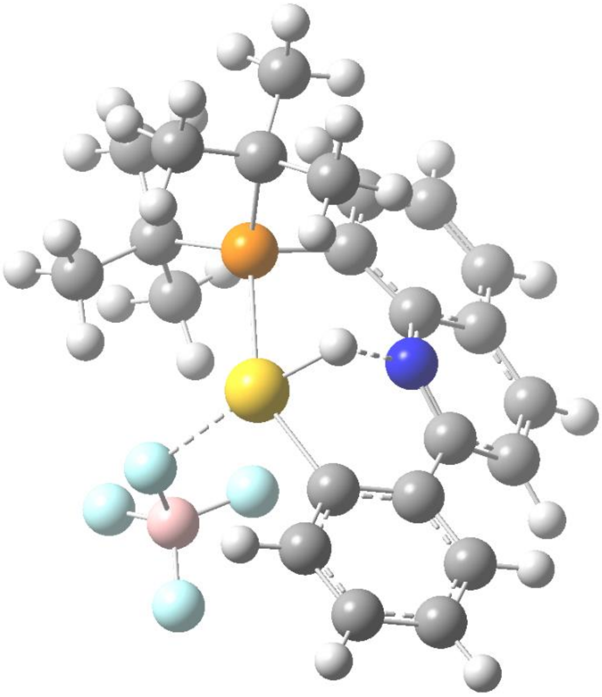

Electronic Energy (EE) = -1848.3455 Hartree
Zero-point Energy Correction = 0.450156 Hartree
Thermal Correction to Energy = 0.483387 Hartree
Thermal Correction to Enthalpy = 0.484331 Hartree
Thermal Correction to Free Energy = 0.385994 Hartree
EE + Zero-point Energy = -1847.8953 Hartree
EE + Thermal Energy Correction = -1847.8621 Hartree
EE + Thermal Enthalpy Correction = -1847.8611 Hartree
EE + Thermal Free Energy Correction = -1847.9595 Hartree

C	2.93003100	2.08231400	0.83083500
C	1.82520600	1.50570500	0.21288500
C	0.59474500	2.25165900	0.26929200
C	0.47816800	3.42650000	1.07730300
C	1.64157400	3.96480100	1.67265500
C	2.85179200	3.31719300	1.51540200
C	-0.83882700	3.93653300	1.26497100
C	-1.93588700	3.28143000	0.74796100
C	-1.72851400	2.14790100	-0.09405700
H	3.88872200	1.56483300	0.82707900
H	1.56994200	4.87671600	2.27007400
H	3.75511600	3.72723800	1.96959100
H	-0.97313400	4.82357100	1.88869300
H	-2.94374400	3.61050000	1.00008000
C	-2.80244300	1.24732100	-0.60011200
C	-4.11563400	1.73594500	-0.72897600
C	-2.54296400	-0.12710800	-0.88401600
C	-5.15717700	0.90443900	-1.13198600
H	-4.32049700	2.79011500	-0.52980100

C	-3.59901800	-0.94308700	-1.29200900
C	-4.90001800	-0.43934500	-1.40565500
H	-6.16755700	1.30461800	-1.22994300
H	-3.41962700	-1.99905600	-1.49807900
H	-5.71336200	-1.10411200	-1.70399700
N	-0.48711700	1.79425000	-0.38262900
P	1.84487800	-0.25837500	-0.41475300
C	2.81295200	-1.31454500	0.85372400
C	2.70786100	-0.14305600	-2.11300700
C	4.04950900	0.60530400	-2.06419700
H	4.46513500	0.64378100	-3.08421800
H	3.92349500	1.64161900	-1.72247600
H	4.79246800	0.11373900	-1.42584300
C	1.76488800	0.63466800	-3.04975000
H	0.83070000	0.08807900	-3.23811900
H	1.51704700	1.63156000	-2.65760000
H	2.27122800	0.77420900	-4.01841000
C	2.90271300	-1.56082400	-2.67338300
H	3.25212300	-1.48743600	-3.71577100
H	3.65432600	-2.13359600	-2.11428300
H	1.96163300	-2.13018600	-2.67458700
C	4.34603700	-1.25806300	0.77312100
H	4.74845800	-1.88974000	1.58158100
H	4.73503900	-1.65891300	-0.17254400
H	4.75683000	-0.25077200	0.92128200
C	2.34759700	-0.88168800	2.25596900
H	2.77263100	0.08580300	2.55340500
H	1.25432100	-0.82378000	2.34456600
H	2.68883300	-1.63983100	2.97809600
C	2.36085600	-2.77119900	0.61554800
H	2.61501000	-3.13414200	-0.38948300
H	2.88327300	-3.41346200	1.34242500
H	1.28292800	-2.89726600	0.77782800
Au	-0.57041800	-0.81012000	-0.61804200
H	-0.45871400	0.38633000	-1.56761100
B	-1.37184400	-1.88665000	2.33623900
F	-0.59674400	-2.57414100	3.25762800
F	-1.07907500	-0.50382400	2.33806800
F	-0.92638900	-2.38113900	0.96555300
F	-2.72272400	-2.13740300	2.44061500

Int-PyH

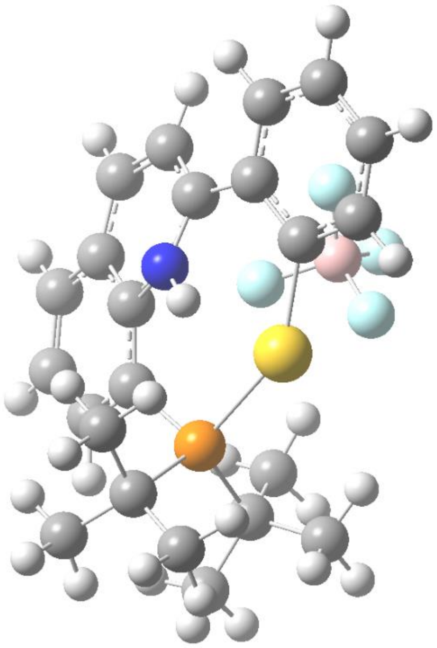

Electronic Energy (EE) = -1848.38 Hartree
Zero-point Energy Correction = 0.454756 Hartree
Thermal Correction to Energy = 0.488234 Hartree
Thermal Correction to Enthalpy = 0.489178 Hartree
Thermal Correction to Free Energy = 0.389795 Hartree
EE + Zero-point Energy = -1847.9253 Hartree
EE + Thermal Energy Correction = -1847.8918 Hartree
EE + Thermal Enthalpy Correction = -1847.8908 Hartree
EE + Thermal Free Energy Correction = -1847.9902 Hartree

C	-2.48601200	2.23700500	0.97085200
C	-1.55502500	1.19900800	0.93571400
C	-0.24413300	1.54590500	1.38146500
C	0.15774900	2.88185000	1.64947000
C	-0.83721500	3.88205800	1.66475900
C	-2.14590400	3.54690400	1.37099900
C	1.55785100	3.12522100	1.77491600
C	2.49197600	2.13325400	1.56664700
C	2.05811400	0.79769300	1.37039400
H	-3.50737000	2.05630100	0.63751400
H	-0.55693300	4.91567500	1.87475500
H	-2.92097600	4.31436200	1.37887200
H	1.88944700	4.15362700	1.93194900
H	3.54984800	2.37169700	1.48430200
C	2.85669300	-0.37282500	0.98299800
C	4.22369500	-0.38323300	1.32599400
C	2.28457500	-1.44874500	0.21294400
C	5.04784200	-1.43273100	0.94159700
H	4.63734800	0.43518800	1.91885800
C	3.15305600	-2.48946900	-0.14386200
C	4.50845100	-2.48728000	0.20112600
H	6.10370200	-1.42924000	1.21564500

H	2.76979800	-3.32216900	-0.73604800
H	5.14975400	-3.31139100	-0.11972100
N	0.73391300	0.60343200	1.48976900
P	-1.91193500	-0.42629800	0.05674000
C	-2.90376300	-0.02389300	-1.52862100
C	-2.90314300	-1.42075600	1.34655000
C	-4.11200300	-0.67351200	1.92779800
H	-4.58515800	-1.31066100	2.69276900
H	-3.81365900	0.26170600	2.42120200
H	-4.87509100	-0.44394200	1.17509500
C	-1.92273200	-1.72332000	2.49592600
H	-1.04967800	-2.29460600	2.14828300
H	-1.57364300	-0.80449500	2.99092500
H	-2.44332500	-2.32629100	3.25730300
C	-3.34385500	-2.74926300	0.71138500
H	-3.77625400	-3.39438400	1.49299100
H	-4.11179300	-2.61014000	-0.06140800
H	-2.49329300	-3.28527400	0.26508200
C	-4.41501000	0.18887100	-1.36179000
H	-4.83722600	0.41896900	-2.35334100
H	-4.93253100	-0.70564400	-0.98916600
H	-4.66430700	1.03376800	-0.70638800
C	-2.25863700	1.20652900	-2.18723400
H	-2.46913600	2.13689100	-1.64533100
H	-1.16817500	1.11055200	-2.28477900
H	-2.67977600	1.30502400	-3.20091600
C	-2.66776500	-1.23695600	-2.45428800
H	-3.04588500	-2.17923200	-2.03480700
H	-3.19731800	-1.05519700	-3.40309000
H	-1.59981700	-1.35849200	-2.68326100
Au	0.25645100	-1.36665200	-0.31373100
H	0.43480500	-0.38740500	1.37405800
B	1.57194300	1.91331500	-2.10562100
F	1.78159600	2.74372900	-3.20452600
F	0.62541100	2.51639900	-1.20542700
F	1.02806500	0.65701800	-2.52350500
F	2.77827400	1.68955100	-1.39347500

7. References

(1) Martín, J.; Schörgenhumer, J.; Biedrzycki, M.; Nevado, C. *Inorg. Chem.* **2024**, *63*, 8390-8396.

(2) Zhang, W.; Sprafke, J. K.; Ma, M.; Tsui, E. Y.; Sydlik, S. A.; Rutledge, G. C.; Swager, T. M. *J. Am. Chem. Soc.* **2009**, *131*, 8446-8454.

(3) Sankaralingam, M.; Lee, Y.-M.; Karmalkar, D. G.; Nam, W.; Fukuzumi, S. *J. Am. Chem. Soc.* **2018**, *140*, 12695-12699.

(4) Fulmer, G. R.; Miller, A. J. M.; Sherden, N. H.; Gottlieb, H. E.; Nudelman, A.; Stoltz, B. M.; Bercaw, J. E.; Goldberg, K. I. NMR Chemical Shifts of Trace Impurities: Common Laboratory Solvents, Organics, and Gases in Deuterated Solvents Relevant to the Organometallic Chemist. *Organometallics* **2010**, *29*, 2176-2179.

(5) Rigaku Oxford Diffraction, CrysAlisPro Software System, Version 1.171.41.105a, Rigaku Corporation, Wroclaw, Poland, 2021.

(6) Zhu, Z.; Fettinger, J. C.; Olmstead, M. M.; Power, P. P. *Organometallics* **2009**, *28*, 2091-2095.

(7) Cuesta-Galisteo, S.; Schörgenhumer, J.; Hervieu, C.; Nevado, C. *Angew. Chem., Int. Ed.* **2024**, *63*, e202313717.

(8) Gaussian 16, Revision C.01, Frisch, M. J.; Trucks, G. W.; Schlegel, H. B.; Scuseria, G. E.; Robb, M. A.; Cheeseman, J. R.; Scalmani, G.; Barone, V.; Petersson, G. A.; Nakatsuji, H.; Li, X.; Caricato, M.; Marenich, A. V.; Bloino, J.; Janesko, B. G.; Gomperts, R.; Mennucci, B.; Hratchian, H. P.; Ortiz, J. V.; Izmaylov, A. F.; Sonnenberg, J. L.; Williams-Young, D.; Ding, F.; Lipparini, F.; Egidi, F.; Goings, J.; Peng, B.; Petrone, A.; Henderson, T.; Ranasinghe, D.; Zakrzewski, V. G.; Gao, J.; Rega, N.; Zheng, G.; Liang, W.; Hada, M.; Ehara, M.; Toyota, K.; Fukuda, R.; Hasegawa, J.; Ishida, M.; Nakajima, T.; Honda, Y.; Kitao, O.; Nakai, H.; Vreven, T.; Throssell, K.; Montgomery Jr., J. A.; Peralta, J. E.; Ogliaro, F.; Bearpark, M. J.; Heyd, J. J.; Brothers, E. N.; Kudin, K. N.; Staroverov, V. N.; Keith, T. A.; Kobayashi, R.; Normand, J.; Raghavachari, K.; Rendell, A. P.; Burant, J. C.; Iyengar, S. S.; Tomasi, J.; Cossi, M.; Millam, J. M.; Klene, M.; Adamo, C.; Cammi, R.; Ochterski, J. W.; Martin, R. L.; Morokuma, K.; Farkas, O.; Foresman, J. B.; Fox, D. J. Gaussian, Inc., Wallingford CT, **2019**.

(9) (a) Perdew, J. P.; Burke, K.; Ernzerhof, M. *Phys. Rev. Lett.* **1996**, *77*, 3865-3868; (b) Perdew, J. P.; Burke, K.; Ernzerhof, M. *Phys. Rev. Lett.* **1997**, *78*, 1396.

(10) Weigend, F.; Ahlrichs, R. *Phys. Chem. Chem. Phys.*, **2005**, *7*, 3297-3305.

(11) Thom, A. J. W.; Sundstrom, E. J.; Head-Gordon, M. *Phys. Chem. Chem. Phys.* **2009**, *11*, 11297-11304.

(12) Lu, T.; Chen, F. W. *J. Comput. Chem.* **2012**, *33*, 580-592.
